# Dynamic Reactivity: Reversible Reaction of a Phosphinine–Borane Adduct With Water and Double Hydrophosphination

**DOI:** 10.1002/chem.202503493

**Published:** 2026-02-21

**Authors:** Samantha Frank, Ádám Horváth, Júlia Boglárka Horváth, Chiara Interdonato, Moritz J. Ernst, Priya Kumar, Zoltán Benkő, Christian Müller

**Affiliations:** ^1^ Institute of Chemistry and Biochemistry Freie Universität Berlin Berlin Germany; ^2^ Department of Inorganic and Analytical Chemistry, Faculty of Chemical Technology and Biotechnology Budapest University of Technology and Economics Budapest Hungary; ^3^ HUN‐REN‐BME Computation Driven Chemistry Research Group Budapest University of Technology Budapest Hungary

**Keywords:** DFT Calculations, hydrophosphination, Lewis Pairs, O─H‐bond activation, phosphorus heterocycles

## Abstract

An unprecedented reversible addition of water to a 3,5‐bis(SiMe_3_)phosphinine→B(C_6_F_5_)_3_ was observed, revealing the formation of novel 1,2‐ and 1,4‐dihydrophosphinine oxide–borane adducts. Experimental results, supported by DFT calculations, unveiled two plausible reaction pathways, enabled by the liberation of phosphinine upon water coordination to the borane. On the basis of our results, the water addition can be reversed under basic conditions, regenerating the aromatic phosphorus heterocycle. Furthermore, treating the mixture of the dihydrophosphinine oxides with the sterically demanding and weak base tris(*tert*‐butyl)phosphine induced a unique double hydrophosphination, yielding a bicyclic diphosphine oxide. These findings highlight a new mode of small molecule activation and dynamic structural interconversion by aromatic phosphorus heterocycles.

## Graphical abstract

A phosphinine–borane Lewis pair shows a dynamic mode of water addition, revealing reversible and regioselective transformations that expand the reactivity of aromatic phosphorus heterocycles and open pathways to stimuli‐responsive systems for catalysis, small‐molecule activation, and functional materials.

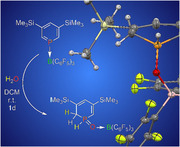

## 1 Introduction

Phosphinines, the higher homologs of pyridines, are currently undergoing a renaissance in organophosphorus chemistry. They provide new directions in the fields of coordination chemistry, homogeneous catalysis, activation of small molecules, and photoluminescent molecular materials [1, 2, 3, 4, 5, 6, 7, 8, 9, 10, 11, 12, 13, 14, 15, 16, 17, 18, 19, 20].

**FIGURE 1:**
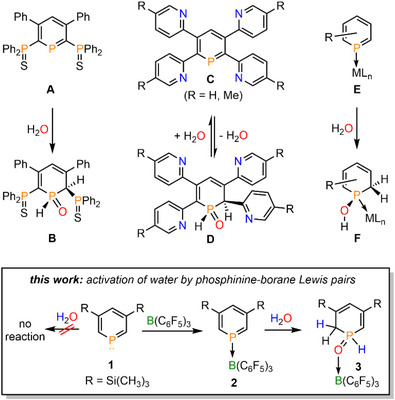
Reaction of donor‐functionalized phosphinines and phosphinine metal complexes with water and a brief summary of this work.

The typically highly reactive P═C double bond, as present for instances in phosphaalkenes (R‐P═CR_2_), usually requires kinetic stabilization by bulky substituents. If these groups are sterically sufficiently demanding (e.g. mesityl or fluorenyl groups) the P═C double bond becomes resistant even toward water [21, 22, 23]. Alternatively, the integration of a reactive P═C moiety into an aromatic system leads to the general inertness of phosphinines toward water or alcohols. However, two exceptions have been reported in the literature so far. In 2003, Le Floch and coworkers prepared the donor‐functionalized, SPS‐pincer type λ^3^‐phosphinine **A**, which, upon reaction with water, yielded the corresponding 1,2‐dihydrophosphinine oxide **B** (Figure 1) [24]. As a second example, we have recently shown that the tetrapyridyl‐substituted phosphinines **C** (Figure 1, R═H, Me) react with water to form the 1,2‐dihydrophosphinine oxides **D** selectively [25]. Most strikingly, this conversion is fully reversible in contrast to the analogous reaction **A** + H_2_O→**B**. Mechanistic studies suggest that the water addition to the P═C double bond of **C** occurs *via* P,N‐cooperativity. In fact, the complementary participation of the formally electrophilic (Lewis acidic) phosphorus center and the nucleophilic (Lewis basic) pyridyl‐nitrogen center resembles the previously reported modes of H_2_O activation by intramolecular Frustrated Lewis Pairs (FLPs). In the backward direction, the rearomatization of the heterocycle under the elimination of water maintains the reversibility.

Apart from these two examples, transition metal complexes containing phosphinine ligands (**E**, Figure 1) readily react with water and alcohols, due to a significant disruption of the aromaticity within the phosphorus heterocycle [26, 27]. Hence, the phosphorus atom becomes prone to nucleophilic attack, leading ultimately to the addition of the RO─H moiety to the P═C double bond under the formation of the phosphinous acid complexes **F**. As the lone pair of the phosphorus atom is occupied by the metal fragment, the isomerization to the corresponding secondary phosphine oxide is no longer possible.

During our recent investigations on the synthesis of novel phosphinine‐borane Lewis pairs and their reactivity toward unsaturated substrates, we have shown that **2** can mimic frustrated Lewis pair (FLP) type reactivity [28, 29]. Herein, we now demonstrate that the Lewis pair **2** readily reacts with H_2_O, leading to hitherto unknown borane adducts of 1,2‐ and 1,4‐dihydrophosphinine oxide derivatives (Figure 1). We also show that these species exhibit intriguing dynamic reactivities toward different bases.

## 2 Results and Discussion

**FIGURE 2:**
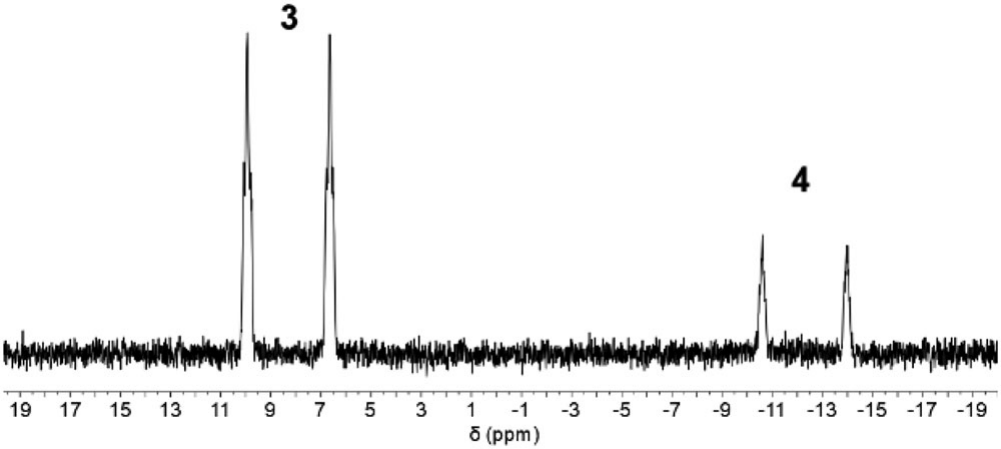
^31^P NMR spectrum for the reaction of 2 with H_2_O.

**SCHEME 1:**
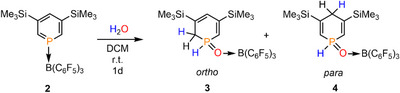
Reaction of Lewis pair 3,5‐bis(TMS) phosphinine→B(C_6_F_5_)_3_ (**2**) with H_2_O in CH_2_Cl_2_ at room temperature under formation of 1,2‐ (*ortho*) and 1,4‐ (*para*) dihydrophosphinine oxide derivatives **3** and **4**.

As already anticipated for phosphinines without additional substituents (e.g. P(S)Ph_2_ or pyridyl groups) in *ortho* position, the 3,5‐bis(trimethylsilyl)‐substituted phosphinine **1** shows no reaction with H_2_O at room or elevated temperatures, as demonstrated by ^31^P{^1^H} NMR spectroscopic studies. After establishing the inertness of **1** toward water, we added a large excess of H_2_O to a solution of **2** in CH_2_Cl_2_, which led to complete dissociation of **2** (and formation of free phosphinine **1**) within five minutes according to the ^31^P NMR spectra. Apparently, the weakly Lewis basic character of the low‐coordinated phosphorus atom in **1** allows the full displacement of the borane from the Lewis pair **2** by a more nucleophilic H_2_O molecule. Furthermore, the ^19^F and ^11^B NMR spectra also indicate the formation of borane‐water adducts with the general formula of [H_2_O→B(C_6_F_5_)_3_] or [H_2_O→B(C_6_F_5_)_3_]·(H_2_O)_n_ (where n = 2,3) [30, 31]. Much to our surprise, after 1 day, we detected the formation of two new species in the reaction mixture, which showed resonances in the ^31^P NMR spectrum at δ(ppm) = 7.7 and ‐12.9, with large coupling constants of ^1^ *J*_P‐H_ = 533.8 Hz and 545.5 Hz, indicating the formation of a P─H bond (corresponding to **3** and **4**, respectively, see Figure 2 and Scheme 1). In order to achieve a more controlled addition of water, a stock solution of H_2_O in CH_2_Cl_2_ was prepared (see the Supporting Information), which enabled the addition of one equivalent of H_2_O to a CH_2_Cl_2_ solution of **2**. Again, after the addition of H_2_O, the dissociation of **2** and the formation of [H_2_O→B(C_6_F_5_)_3_] or [H_2_O→B(C_6_F_5_)_3_]·(H_2_O)_n_ adducts were confirmed by ^31^P{^1^H}, ^19^F, and ^11^B NMR spectroscopy. Ultimately, this modified procedure enabled the nearly complete conversion of phosphinine **1** after 1 day of reaction time, along with the formation of **3** and **4** in an approx. 4:1 ratio (Figure 2). Further experiments with varied **2**:H_2_O ratios indicate that (see Table S1) the final product distribution as well as the conversion of phosphinine depend on the added equivalents of H_2_O.

By comparing the NMR spectroscopic data with those of the water‐addition product **D** (Figure 1) [25] and also with computed ^31^P NMR chemical shifts of reference compounds, we assigned the signal of the major product at δ(ppm) = 7.7 (δ(ppm)_calc_ = 9.0) to the 1,2‐dihydrophosphinine (*ortho*) derivative **3** (Scheme 1). Likewise, the resonance of the minor product at δ(ppm) = −12.9 (δ(ppm)_calc_ = −8.1) was assigned to the 1,4‐dihydrophosphinine (*para*) derivative **4** (see Figure 2 and Scheme 1).

**FIGURE 3:**
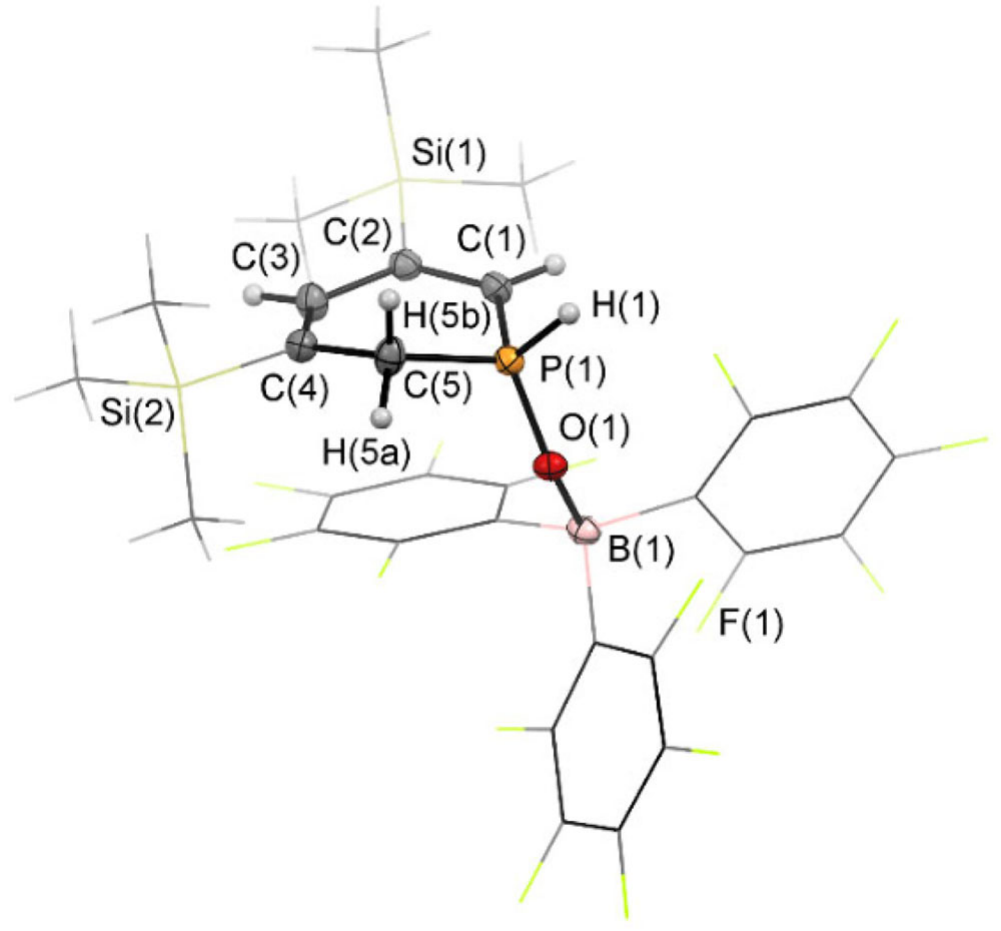
Molecular structure of **3** in the crystal. Thermal ellipsoids are given at 50% probability. Selected bond lengths (Å) and angles (°): P(1)‐O(1): 1.5325(15), O(1)‐B(1): 1.539(3), P(1)‐C(1): 1.760(2), P(1)‐C(5): 1.782(2), C(1)‐C(2): 1.361(3), C(2)‐C(3): 1.471(3), C(3)‐C(4): 1.357(3), C(4)‐C(5): 1.504(3), P(1)‐O(1)‐B(1): 135.71(13).

Additionally, single crystals of the major product **3** were obtained from a toluene solution of the reaction mixture, layered with pentane. The crystallographic characterization of **3** confirms the structure of the *ortho* isomer (Figure 3). The P(1)‐O(1) distance of 1.533 Å in **3** is slightly elongated compared to the reported tetrapyridyl‐substituted 1,2‐dihydrophosphinine oxide **D** (1.482 Å) [25]. The elongation can be attributed to the coordination of the oxygen atom to the Lewis acid B(C_6_F_5_)_3_. The phosphorus heterocycles **3** and **4** are phosphacyclohexadienes with two conjugated (**3**) or isolated (**4**) C═C double bonds, respectively.

To elucidate the mechanistic aspects and to propose possible reaction pathways for the reaction between **2** and H_2_O, yielding the regioisomers **3** and **4**, we carried out DFT calculations at the ωB97X‐D/6‐311+G**(PCM═DCM) level of theory, including an implicit solvent model for dichloromethane. The applied method has recently been tested on similar systems using high‐level *ab initio* calculations [29].

**SCHEME 2:**
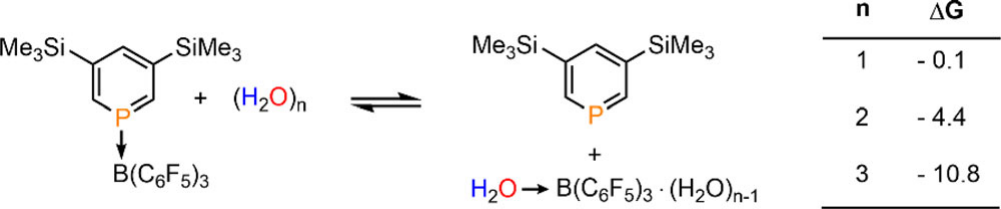
Reaction Gibbs free energies (ΔG, in kcal mol^−1^) between the Lewis pair **2** and hydrogen‐bonded water clusters (H_2_O)_n_ (n = 1–3) forming the separated *free* phosphinine **1** + [H_2_O→B(C_6_F_5_)_3_] or [H_2_O→B(C_6_F_5_)_3_]·(H_2_O)_n‐1_.

As a starting point, based on the observed dissociation of the adduct **2** (see above), we assessed the thermodynamic data for the exchange reactions between the Lewis pair **2** and hydrogen‐bonded water clusters (H_2_O)_n_ (n = 1–3), leading to the free phosphinine **1** and [H_2_O→B(C_6_F_5_)_3_] or [H_2_O→B(C_6_F_5_)_3_]·(H_2_O)_n‐1_ adducts (see Scheme 2).

**SCHEME 3:**
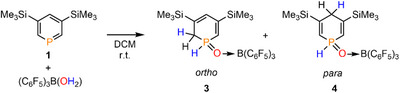
Control reaction of 3,5‐bis(TMS) phosphinine **1** with the adducts [H_2_O→B(C_6_F_5_)_3_] or [H_2_O→B(C_6_F_5_)_3_]·(H_2_O)_n‐1_ in CH_2_Cl_2_ at room temperature, yielding 1,2‐(*ortho*) and 1,4‐(*para*) dihydrophosphinine oxide derivatives **3** and **4**.

We found that an ideal equilibrium scenario can be concluded in the case of a single H_2_O molecule (n = 1), as depicted in Scheme 3 (ΔG = −0.1 kcal·mol^−1^). However, the addition of a second (∆G = −4.4 kcal·mol^−1^) and a third (∆G = −10.8 kcal·mol^−1^) H_2_O molecule gradually shifts the equilibrium toward the liberation of the phosphinine **1** and the formation of the corresponding [H_2_O→B(C_6_F_5_)_3_]·(H_2_O)_n‐1_ (n = 2, 3) species. These computed results are in line with the experimental observations that phosphinine **1** can be released from **2** by water; indicating a complex dynamic equilibrium between free B(C_6_F_5_)_3_, adduct **2** and [H_2_O→B(C_6_F_5_)_3_] or [H_2_O→B(C_6_F_5_)_3_]·(H_2_O)_n‐1_ adducts.

To further investigate the above equilibrium, we prepared an equimolar CH_2_Cl_2_ solution of the [H_2_O→B(C_6_F_5_)_3_] or [H_2_O→B(C_6_F_5_)_3_]·(H_2_O)_n‐1_ adduct according to Beringhelli et al. [30, 31], and added it to a CH_2_Cl_2_ solution of **1** at room temperature. Similarly to the results above, the slow consumption of phosphinine **1**, accompanied by the formation of **3** and **4**, was indeed observed by ^31^P NMR spectroscopy (Scheme 3).

In order to propose a viable mechanism for the formation of **3** and **4**, we performed DFT computations considering several possibilities. As both O─H bonds of H_2_O are cleaved during the reactions in Schemes 1, 2, it is evident that the reactions must follow stepwise mechanisms. It is reasonable to assume that an OH─group is placed on the phosphorus atom of the phosphinine in a first step, which is followed by tautomerization. Knowing that the reactions require the presence of the borane (see above), the first reaction step was modelled using both H_2_O→B(C_6_F_5_)_3_ and H_2_O→B(C_6_F_5_)_3_]·(H_2_O) adducts. In accordance with the literature, the presence of the borane in H_2_O→B(C_6_F_5_)_3_ induces the polarization of the O─H bond, making the boron‐bound water molecule in this compound significantly more acidic [32]. In contrast, in the second hydrogen‐bonded water molecule in [H_2_O→B(C_6_F_5_)_3_]·(H_2_O) this polarization is slightly decreased (Figure S48). Nevertheless, the presence of an additional water molecule was shown to significantly reduce the activation barrier of the second, tautomerization process *via* a network of hydrogen bonds [33].

**FIGURE 4:**
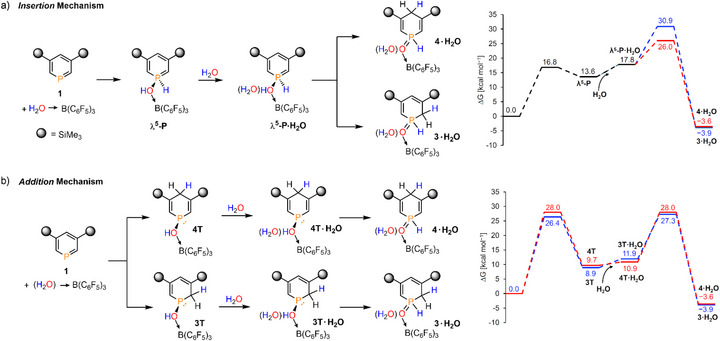
Proposed reaction pathways for the reaction between phosphinine **1** and the [H_2_O→B(C_6_F_5_)_3_]·(H_2_O) adduct *via* a) formal phosphorus‐atom‐insertion into the O‐H bond and b) 1,2‐ or 1,4‐addition of the O‐H bond to the phosphorus heterocycle. Right side: relative Gibbs free energy profiles for the corresponding reactions (ΔG, in kcal mol^−1^). The *zero point* for each Gibbs free energy profile was chosen as the reactant complex from the Intrinsic reaction coordinate calculations. Labels “**T**” and “·H2O” stand for Tautomer and a coordinating H_2_O molecule *via* hydrogen‐bonding.

Building on the increased acidity of the water molecule in H_2_O→B(C_6_F_5_)_3_ adduct, we hypothesized that this species may be acidic enough to initially protonate the free phosphinine **1**. However, all efforts to locate a contact ion pair of protonated phosphinine and the remaining [HO→B(C_6_F_5_)_3_]^−^ borate anion from a direct protonation transition state remained unsuccessful. Considering these results, we have investigated different possibilities (for details, see Supporting Information), and Figure 4 presents the proposed pathways. The key distinction between these two mechanisms lies in the position where the H atom is placed in the first step (parallelly with the formation of the P─OH moiety).

First, we assume a formal insertion of the phosphorus atom of **1** into the O─H bond of H_2_O→B(C_6_F_5_)_3_, followed by H‐migrations onto either the *ortho*‐ or *para*‐C centers of the phosphinine backbone (*insertion* mechanism, see Figure 4a). According to our calculations, the insertion proceeds *via* an activation barrier of ΔG^‡^ = 16.8 kcal mol^−1^ and is endergonic by ΔG = 13.6 kcal mol^−1^. This step leads to the intermediate ***λ^5^*‐P**, exhibiting a >P(H)─OH functionality. Following the addition of a second H_2_O molecule, the intermediate ***λ^5^*‐P·H_2_O** is the starting point toward the formation of the final products **3·H_2_O** and **4·H_2_O**, which are accessed by hydrogen‐shift from the P(H)─OH group to either the *ortho*‐, or the *para*‐position of the ring (see Figure 4a). The barrier leading to the *para* isomer **4·H_2_O** is lower than the barrier for the formation of the *ortho* congener **3·H_2_O** (ΔΔG^‡^ = 4.9 kcal mol^−1^, see Figure 4a, right side). The whole reaction sequences are moderately exergonic by ΔG = ‐3.9 and ‐3.6 kcal mol^−1^, respectively, offering the thermodynamic driving forces of the reactions.

Second, we examined the 1,2‐ or 1,4‐addition of the O─H bond of (H_2_O)→B(C_6_F_5_)_3_ to phosphinine **1**, followed by tautomerization steps to afford the two final products (*addition* mechanism, Figure 4b). In contrast to the *insertion* mechanism, which proceeds through a common intermediate, two different cyclic hydroxyphosphine intermediates **3T** and **4T** are formed in this case, exhibiting similar relative Gibbs free energies (ΔG = 8.9 and 9.7 kcal mol^−1^, respectively). Consistently, the activation barriers leading to these species are also rather similar (ΔG^‡^ = 26.4 and 28.0 kcal mol^−1^, respectively). In the second step, which proceeds with an additional water molecule, **3T·H_2_O** and **4T·H_2_O**, undergo a >P─(OH) to >P(═O)─H tautomerization (Figure 4b), yielding the final products **3·H_2_O** and **4·H_2_O**, respectively. The transition states of these tautomerization reactions are located at comparable relative Gibbs free energies to those corresponding to the 1,2‐/1,4‐addition. As the final products of this sequence are the same as those presented in Figure 4a, the thermodynamic driving forces are also identical (Figure 4b, right side).

Importantly, despite the fundamentally different outcome of the first reaction steps, the corresponding transition states exhibit similar features. By inspecting the transition structures, these reactions can be considered as protonations at the phosphorus (a) and carbon atoms (b), respectively (see Figures S36–S39, S42–S45). However, the recombination of the opposite charges happens within the same concerted reaction step. This statement is in line with the increased Brønsted‐acidity of the water moiety in H_2_O→B(C_6_F_5_)_3_ [32].

Finally, we compared the *insertion* and *addition* mechanisms (Figures 4a,b, respectively) with our experimental observations. The rate‐determining barriers encompass a relatively narrow range between ΔG^‡^ = 26.0–30.9 kcal mol^−1^. Therefore, based solely on these computational data, it is difficult to unambiguously exclude any of these pathways. This is further supported by the observation that the product ratio depends on the relative equivalents of starting materials (Lewis pair and water), and plausibly these pathways compete with each other.

**SCHEME 4:**
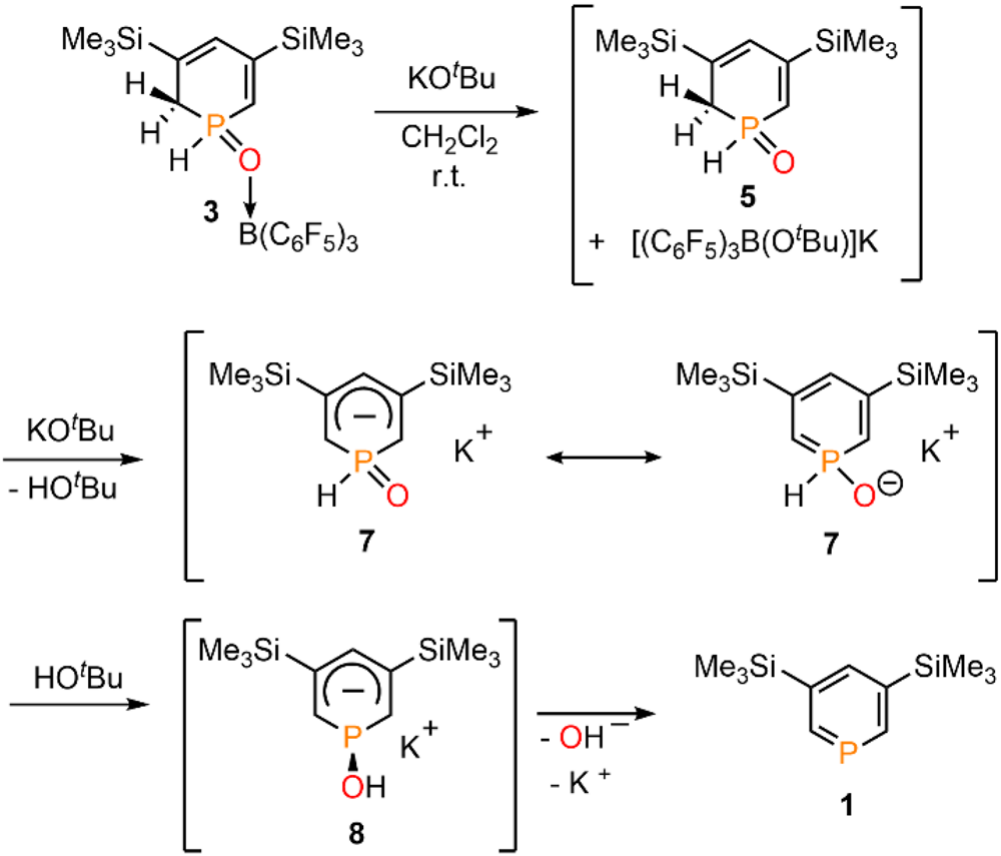
Reaction of **3** with KO*^t^* Bu leading to the formation of phosphinine **1** with the proposed reaction steps.

Having revealed a plausible reaction mechanism for the H_2_O‐activation reaction, we subsequently explored the reactivity of the mixture of **3** and **4**, toward bases from different regions on the basicity scale. We started with KO*^t^* Bu (proton affinity of *^t^* BuO^−^: 331.7 kcal mol^−1^, p*K*_a_ of *^t^* BuOH in DMSO: 32.2) [34], a strong base with significant nucleophilicity. We hypothesized that the *^t^* BuO^−^ anion might bind to B(C_6_F_5_)_3_, liberating the corresponding secondary phosphine oxide. Therefore, KO*^t^* Bu was added to a freshly prepared solution of **3/4** in CH_2_Cl_2_ at room temperature. However, in contrast to our assumption, we observed the instantaneous and quantitative formation of free phosphinine **1** alongside the [*^t^* BuO→B(C_6_F_5_)_3_]^−^ anion, which could be identified by means of mass spectrometry (Scheme 4). This reactivity is perfectly in line with our observation that the reaction of the pyridyl‐functionalized phosphinine **C** (Figure 1) undergoes a reversible H_2_O addition toward **D**, while the rearomatization to the corresponding phosphinine is the driving force for the elimination of H_2_O [25].

To propose a possible mechanism for this base‐assisted reversibility, we computationally explored the decoordination of B(C_6_F_5_)_3_ from **3**/**4** as well as the deprotonation either at the *ortho*/*para* C(sp^3^)─H group, or at the >P(O)H moiety enabled by the *^t^* BuO^−^ anion. For simplicity reasons, we only focus here on the *ortho* isomer **3**, but we anticipate that the transformation similarly proceeds with **4** as well. In its reaction with **3**, the *^t^* BuO^−^ anion can either act as a base, abstracting a proton from the C(sp^3^) center (ΔG = −37.8 kcal mol^−1^) or as a nucleophile, inducing the decoordination of the borane to form **5** and the [*^t^* BuO→B(C_6_F_5_)_3_]^−^ anion (ΔG = −30.6 kcal mol^−1^). Next, **5** can be deprotonated by a second *^t^* BuO^−^ anion at the C(sp^3^) center (ΔG = −20.1 kcal mol^−1^) to form **7** and *^t^* BuOH. Alternatively, the deprotonation may happen at the P(O)─H moiety, however, this is thermodynamically less favored (ΔG = −13.0 kcal mol^−1^.) Subsequently, the formed *^t^* BuOH assists the tautomerization of the >P(O)─H moiety of **7** to P─OH leading to **8** (ΔG = 10.8 kcal·mol^−1^). This reaction has an activation barrier of ΔG^‡^ = 30.0 kcal·mol^−1^, which seems realistic to overcome, considering the large imaginary frequency of 1048*i* cm^−1^ (tunneling effect). In the last reaction step, **8** dissociates into phosphinine **1** and an OH^−^ anion via a low activation barrier (ΔG^‡^ = 12.8 kcal·mol^−1^). This step is slightly exergonic (ΔG = −0.6 kcal·mol^−1^), implying an equilibrium, and the formed free hydroxide ion may further react e.g. with a free borane to form the [HO→B(C_6_F_5_)_3_]^−^ anion, contributing to the thermodynamic sink of the process.

**SCHEME 5:**
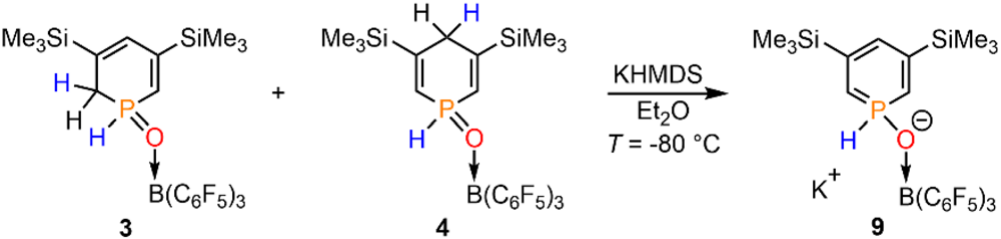
Deprotonation reaction of **3** and **4** with KHMDS.

In order to achieve the *ortho*/*para*‐C(sp^3^)H deprotonation, we turned our attention to the even stronger and less nucleophilic base, KHDMS, potassium hexamethyldisilazanide (proton affinity of [N(TMS)_2_]^−^: 334.2 kcal mol^−1^, p*K*_a_ of HN(TMS)_2_ in DMSO: 30.0) [35]. Thus, one equivalent of KHMDS was added to a freshly prepared mixture of **3** and **4** in Et_2_O at *T* = −80°C to prevent decomposition or side‐reactions. Instantaneously, the color of the solution changed from colorless to yellow. At the same time, the ^31^P NMR spectrum indicated a nearly complete consumption of **3** and **4**, and the appearance of a single doublet resonance at δ(ppm) = 1.5 with ^1^ *J*_PH_ = 547 Hz coupling constant. This ^31^P chemical shift and its large coupling constant are in line with the formation of a borane‐coordinated λ^5^‐phosphinine (**9**), generated by the deprotonation at the C(sp^3^)H‐positions in **3**/**4** (Scheme 5). Compound **9** turned out to be highly air‐, moisture‐, and temperature‐sensitive, and was only characterized by means of multinuclear NMR spectroscopy. Attempts to isolate and characterize it by single crystal X‐ray diffraction have failed so far.

In fact, anion **9** appeared to decompose selectively into the free phosphinine **1** at room temperature, indicating that the reaction with KHMDS ultimately reverses the water‐addition reaction as well. According to our calculations, the reaction with [HMDS]^−^ anion follows a pathway similar to that presented above for *^t^* BuO^−^ (see also Supporting Information for details).

**SCHEME 6:**
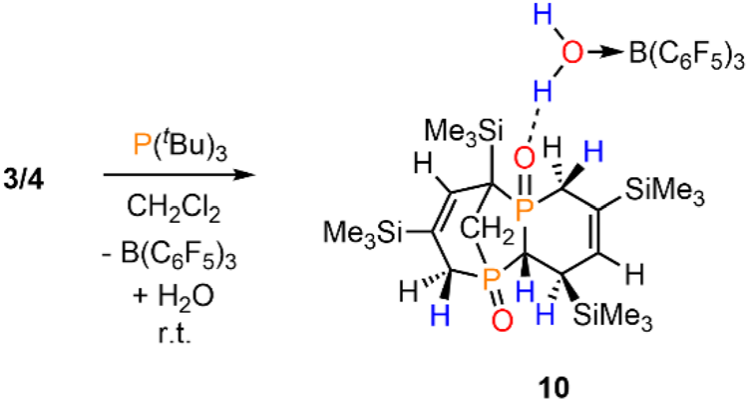
Double hydrophosphination of **3** and **4** in the presence of P(*^t^* Bu)_3_ and formation of **10**.

Finally, we explored the reaction of **3** and **4** with the weak, sterically hindered and nonnucleophilic base, P(*^t^* Bu)_3_ (proton affinity of P*^t^* Bu_3_: 282.8 kcal·mol^−1^, p*K*_a_ of [HP*^t^* Bu_3_]^+^: 11.4) [36]. Upon addition of one equivalent of P(*^t^* Bu)_3_ to a solution of **3**/**4** in CH_2_Cl_2_, protonation of the P(*^t^* Bu)_3_ was observed as the major resonance in ^31^P NMR spectra, alongside unreacted P(*^t^* Bu)_3_. However, after several days, the ^31^P{^1^H} NMR spectrum indicated the formation of free phosphinine **1** and a new main product **10** (Scheme 6), which exhibits two doublet resonances at δ(ppm) = 50.5 and 43.7 with identical coupling constants of *J*_P‐p_ = 53.2 Hz.

**FIGURE 5:**
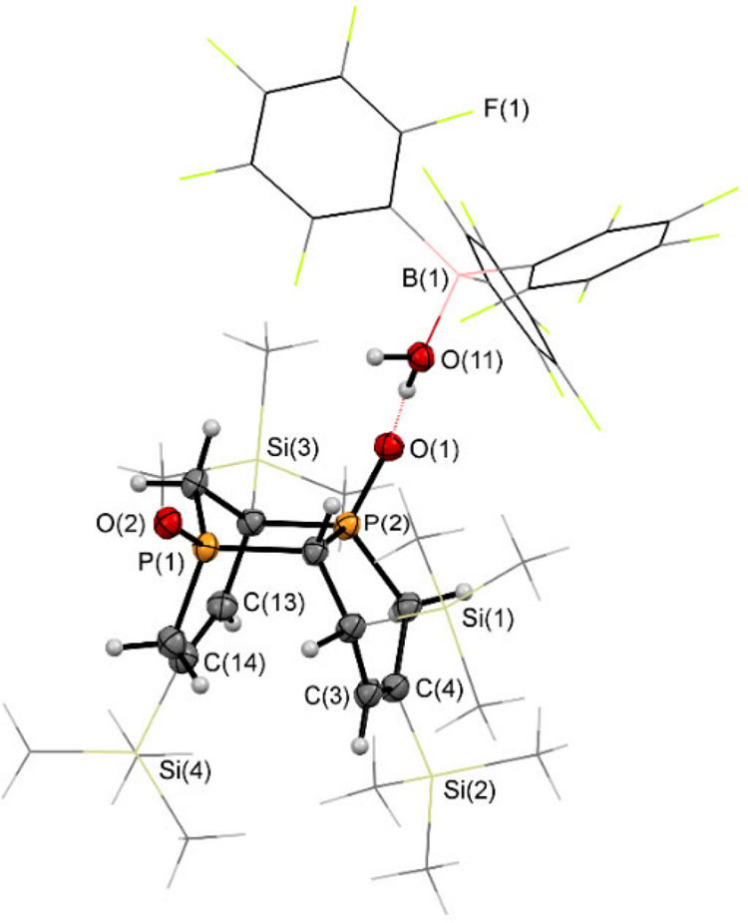
Molecular structure of **10** in the crystal. Displacement ellipsoids are shown at the 50% probability level. Selected bond lengths (Å) and angles (°): P(1)‐O(1): 1.503(2); P(2)‐O(2): 1.493(2); B(1)‐O(11): 1.549(4); P(2)‐C(11): 1.814(3); C(11)‐C(12): 1.557(4); C(12)‐P(1): 1.829(3); P(1)‐C(1): 1.828(3); C(14)‐C(13): 1.342(4); C(3)‐C(4): 1.335(4).

Gratifyingly, we were able to obtain crystals of **10** suitable for a single crystal X‐ray diffraction analysis from a CH_2_Cl_2_ solution layered with pentane at *T* = ‐20°C, giving an isolated yield of 33%. The molecular structure of **10** in the crystal, along with selected bond lengths and angles, is depicted in Figure 5.

Remarkably, the crystallographic characterization reveals the presence of a bicyclic compound that contains two phosphorus atoms (Figure 5). Moreover, the solid‐state structure involves an H_2_O→B(C_6_F_5_)_3_ adduct forming a hydrogen bond with one of the P═O moieties. A closer look at the crystallographic characterization of **10** reveals that the P─H bonds are no longer present, while a total of two C═C double bonds have disappeared, in comparison to the starting materials **3** and **4**.

**SCHEME 7:**
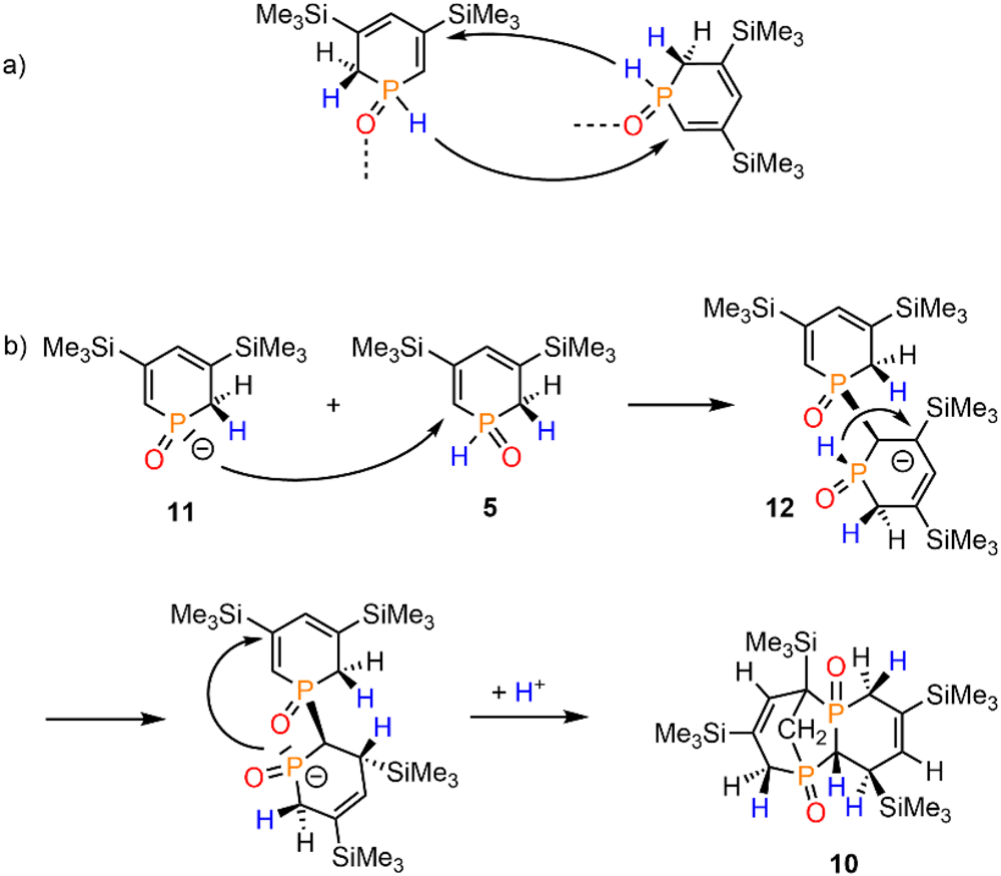
(a) The schematic representation of the hydrophosphination reaction forming compound **10**, and (b) a possible mechanism of this reaction starting from anion **11** and **5**.

Apparently, compound **10** is formally generated by a twofold intermolecular hydrophosphination reaction, in which the P─H and the adjacent C═C double bond of the second phosphorus heterocycle are involved (Scheme 7a). In fact, secondary phosphine oxides are known to react with C═C double or C≡C triple bonds using acidic or basic reaction conditions, radical initiators or even metal catalysts [37, 38, 39, 40, 41, 42]. Notably, P(*^t^* Bu)_3_ also seems to react slowly with the solvent CH_2_Cl_2_, as [ClP(*^t^* Bu)_3_]^+^ and [CH_3_P(tBu)_3_]^+^ could be identified as by‐products of the reaction by means of ^31^P NMR‐spectroscopy. Furthermore, compound **10** resembles the hydrocarbon C_12_H_16_, which was obtained by the cobalt‐catalyzed cycloaddition of bicyclo[3.2.1]‐octa‐2,6‐diene with butadiene [43].

In order to propose a plausible pathway for the formation of **10**, we investigated the hydrophosphination of **3**/**4** computationally at the ωB97X‐D/6‐311+G(d,p)/ωB97X‐D/6‐31+G(d)(PCM═DCM) level of theory. Knowing that B(C_6_F_5_)_3_ is not attached to the P═O unit in **10** anymore, as well as to decrease computational demand, phosphinine oxide **5** was taken as the starting point of this investigation (Scheme 7b). In accordance with experimental findings, P(*^t^* Bu)_3_ acts as a base and deprotonates **5** at the phosphorus atom, leading to **11**. While the *ortho* C(sp^3^)‐H_2_ deprotonation, leading to anion **7**, is thermodynamically more favored (see above), the P─H deprotonation by P(*^t^* Bu)_3_ is kinetically favored with a Gibbs free energy barrier of ΔG^‡^ = 23.8 kcal mol^−1^, which is similar to that of C(sp^3^)─H deprotonation. Based on these results, the coexistence of anions **7** and **11** cannot be excluded, but we propose that the main reactive species for the formation of **10** is anion **11**, in line with previous reports on base‐catalyzed hydrophosphination reactions [37, 38, 39, 40, 41, 42]. Therefore, in the first step, **11** attacks the *ortho*‐C(sp^2^)H carbon of an intact **5** and forms the dimeric anion **12** (Scheme 7, ΔG = 0.6 kcal mol^−1^). The new P─C bond is located on the same side of the ring of **5** as the P─H bond (see Scheme 7). In the following step, an intramolecular H‐transfer occurs from the P(O)─H moiety to the C(3) carbon (ΔG = −13.3 kcal mol^−1^) *via* a Gibbs free energy barrier of ΔG^‡^ = 26.5 kcal mol^−1^. Due to the fixed stereochemistry of the P(O)H function, this H‐transfer is stereospecific, which configuration nicely agrees with the crystallographic data (see Scheme 7 and Figure 5). The newly established P(O)^−^ moiety can now close the bicyclic ring *via* an attack on the *ortho*‐C(sp^2^) center of the second heterocycle (ΔG = −14.3 kcal mol^−1^), through a small activation barrier of ΔG^‡^ = 13.3 kcal mol^−1^. In the final step, the bicyclic anion **12** regains a proton from the [HP(***^t^*** Bu)_3_]^+^ cation, leading to **10** and regenerating the catalyst P(***^t^*** Bu)_3_ (in a thermodynamically highly favored step, ΔG = −59.2 kcal mol^−1^). Although we cannot rule out further possibilities, the presented mechanistic model is both kinetically and thermodynamically feasible.

## 3 Conclusion

In this work, we have described a unique mode of water addition to a phosphinine‐borane Lewis pair, leading to the borane adducts of 1,2‐ and 1,4‐dihydrophosphinine oxide **3** and **4** in an isomeric mixture. The 3,5‐bis(TMS)‐phosphinine **1**, which is chemically inert toward water, undergoes water addition, enabled by the strong Lewis acid B(C_6_F_5_)_3_ and traced back to highly dynamic equilibria, involving phosphinine, borane, and water. Detailed DFT calculations reveal two plausible and concurrent reaction mechanisms, starting either with a formal P insertion into the O─H bond or with the addition of the O─H bond to the phosphinine ring. These starting reaction steps (which can be described as protonations by the rather acidic H_2_O→B(C_6_F_5_)_3_ adduct) are followed by water‐mediated tautomerizations, yielding the observed products. Our further experimental investigations showcase novel distinctive and dynamic reactivities of these dihydrophosphinine oxide—borane adducts with different bases, depending on their relative nucleophilicity and basicity. As such, we could demonstrate the nearly complete regeneration of the free phosphinine by deprotonation reactions, as well as the stereoselective generation of a novel bicyclic bis(phosphine oxide).

The observed dynamic behavior underscores the potential of phosphinine‐based systems in reversible small‐molecule transformations. Moreover, it exemplifies their multifaceted reactivity toward bases, which could be exploited to novel phosphorus heterocycles.

Our findings expand the fundamental understanding of aromatic phosphorus heterocycles by introducing a rare, dynamic water activation pathway mediated by a main‐group Lewis acid. Thus, the reactions described here may open new directions for the design of stimuli‐responsive phosphorus‐based systems with applications in catalysis, small‐molecule activation, and functional materials.

## 4 Experimental Section

All reactions and workups were performed following standard schlenk techniques or using an argon filled *Unilab* glovebox by *MBraun* (H_2_O < 0.1 ppm, O_2_< 0.1 ppm). Room temperature refers to *T*  =  23°C, while elevated reaction temperatures are referred to as the respective oil bath's temperature. Solvents and reagents were degassed using the *freeze‐pump‐thaw* method (three cycles) or by purging with argon. Dry toluene, dichloromethane, and *n*‐pentane were collected from a solvent purification system, *MB SPS‐800* by *MBraun*, and additionally stored over 3 Å molecular sieves prior to use. Dry diethyl ether was stirred over Na, distilled, and degassed. CD_2_Cl_2_ and triethylamine were dried over CaH_2_, distilled, and degassed. PCl_3_ was refluxed and distilled prior to use. All other commercially available chemicals were used without further purification. Preparative, inert filtration over dried silica gel (particle size: 0.040‐0.063 mm, surface area: 500 m^2^/g) by *Sigma‐Aldrich* as the stationary phase under an argon atmosphere. ^1^H, ^1^H{^31^P}, ^13^C{^1^H}, ^31^P, ^31^P{^1^H}, ^19^F, and ^11^B NMR spectra were recorded on *JEOL* (ECX 400, Lamba 400, ECP 500, ECZ 600), and *Bruker* (AVANCE 500, AVANCE 700) spectrometers. NMR chemical shifts are referenced to the IUPAC standards [45]. Quantitative ^31^P NMR spectroscopic measurements were carried out using triphenylphosphine as an internal standard. Mass spectra were measured with an Agilent 6210 ESI‐TOF Agilent Technologies, Santa Clara, CA, USA. The flow rate and the spray voltage were set at 4 µL · min^−1^ and at 4 kV, respectively. The desolvation gas was set at 15 psi (1 bar). All other parameters were optimized for a maximum abundance of the respective [M+H]^+^. EI mass spectra were measured on a MAT 711, Varian MAT, Bremen. Electron energy for EI was set to 80 eV. NMR and mass spectra were analyzed and displayed with the software MestReNova 7.1.2 developed by Mestrelab Research. Single crystal X‐ray diffraction data was collected on a *Bruker D8 Venture* fitted with a Photon II CMOS Detector with Mo *K*α radiation (λ  = 0.71073 Å) or Cu *K*α radiation (λ  = 1.54178 Å) from an *IµS* micro‐source, performing φ‐and ω‐scans. Deposition Numbers 2492243 (for **3**), 2492244 (for **10**) contain the supplementary crystallographic data for this paper. These data are provided free of charge by the joint Cambridge Crystallographic Data Centre and Fachinformationszentrum Karlsruhe http://www.ccdc.cam.ac.uk/structures.

### 4.1 Synthesis of Dihydrophosphinine Oxide (**3**/**4**)

Water (in CH_2_Cl_2_, *c* = 0.078 mmol/mL, 1.64 mL, 1.0 eq) was added to a solution of 3,5‐bis(TMS) phosphinine (**1**) (0.125 mmol, 30 mg, 1.0 eq) and tris(pentafluorophenyl)borane (0.125 mmol, 64 mg, 1.0 eq) in CH_2_Cl_2_ (1 mL). After stirring at rt for 24 h, the solvent was removed in high vacuum at *T* = 50°C. Dihydrophosphinine oxide **3/4** was obtained as a colorless oil (96 mg, quant.). Crystals suitable for X‐ray diffraction were obtained from a toluene solution, layered with pentane at rt. Spectral data for **3**: **^1^H NMR** (600 MHz, CD_2_Cl_2_, 25°C): δ= 7.54 and 6.66 (d, ^1^ *J_PH_* = 533.0 Hz, 1H, *P*─*H*), 6.68 (dd, *J* = 5.5, 2.4 Hz, 1H, *CH‐para*), 6.36 (d, *J_PH_* = 23.5 Hz, 1H, *CH‐ortho*), 3.22 (t, *J_PH_* = 20.1 Hz, 1H, *CH_2_*), 2.70‐2.65 (m, 1H, *CH_2_*), 0.18 (s, 9H, *TMS*), 0.17 (s, 9H, *TMS*) ppm. **^13^C{^1^H} NMR** (150 MHz, CD_2_Cl_2_, 25°C): δ= 168.2 (d, *^2^J_CP_* = 5.8 Hz, *C‐TMS*), 149.0 (d, *^1^J_CF_* = 239.8 Hz, *CF‐ortho*), 141.0 (d, *^1^J_CF_* = 245.5 Hz, *CF‐para*), 139.7 (d, *^2^J_CP_* = 14.6 Hz, *C‐TMS*), 138.2 (d, *^1^J_CF_* = 247.8 Hz, *CF‐meta*), 133.0 (d, *^3^J_CP_* = 36.81 Hz, *CH‐para*), 113.9 (d, *^1^J_CP_* = 88.2 Hz, *CH‐ortho*), 24.1 (d, *^1^J_CP_* = 75.1 Hz, *CH_2_*), 1.1 (s, *Si(CH_3_)_3_*), 0.8 (s, *Si(CH_3_)_3_*) ppm. **^31^P NMR** (162 MHz, CD_2_Cl_2_, 19°C): δ= 7.7 (d, *^1^J_PH_* = 528.4 Hz) ppm. **^31^P{^1^H} NMR** (162 MHz, CD_2_Cl_2_, 19°C): δ= 7.7 (s) ppm. **^19^F NMR** (376 MHz, CD_2_Cl_2_, 19°C): δ= ‐136.6 (d, *J_FF_* = 19.6 Hz, 2F, *o‐F*), ‐160.6 (td, *J_FF_* = (20.3, 5.5) Hz, 1F, *p‐F*), ‐166.5 (m, 2F, *m‐F*) ppm. **^11^B NMR** (128 MHz, CD_2_Cl_2_, 20°C): δ= ‐4.0 (s, br) ppm. **IR** (ATR, rt, 4 cm^−1^): $\tilde{\nu }=$ 2959 (w, br), 2903 (vw, br), 2361 (w, br, $\nu_{PH}$) 2343 (vw, br, $\nu_{PH}$), 1644 (m, $\nu_{C=C}$), 1515 (s, $\nu_{C=C}$), 1462 (vs, $\nu_{C=C}$), 1374 (w, $\nu_{C=C}$), 1283 (m, $\delta_{TMS}$), 1253 (m, $\delta_{TMS}$), 1095 (s, $\delta_{PH}$), 971 (s, $\delta_{PH},\;\delta_{BC}$), 837 (vs, $\delta_{TMS}$) cm^−1^. **ESI‐MS** (m/z) calculated for [C_11_H_24_PSi_2_O]^+^ [M+H]^+^ 259.1103; found: 259.1130. Calculated for [C_11_H_23_PSi_2_ONa]^+^ [M+Na]^+^ 281.0923; found: 281.0943. Spectral data for **4**: **^1^H NMR** (600 MHz, CD_2_Cl_2_, 25°C): δ= 7.76 and 6.85 (d, *^1^J_PH_* = 544.0 Hz, 1H, *P*─*H*), 6.47 (td, *J_PH_* = 17.3 Hz, *J_HH_* = 1.8 Hz, 2H, *CH‐ortho*), 3.36 (ddt, *J* = (7.1, 3.4, 1.8) Hz, 1H, *CH_2_‐para*), 3.32 (ddt, *J* = (7.0, 3.5, 1.8) Hz, 1H, *CH_2_‐para*), 3.20‐3.13 (m, 1H, *CH_2_‐para*), 0.19 (s, 18H, *TMS*) ppm. **^13^C{^1^H} NMR** (150 MHz, CD_2_Cl_2_, 25°C): δ= 176.9 (d, *^2^J_CP_* = 5.3 Hz, *C‐TMS*), 149.0 (d, *^1^J_CF_* = 239.8 Hz, *CF‐ortho*), 141.0 (d, *^1^J_CF_* = 245.5 Hz, *CF‐para*), 138.2 (d, *^1^J_CF_* = 247.8 Hz, *CF‐meta*), 116.9 (d, *^2^J_CP_* = 91.2 Hz, *CH‐ortho*), 34.9 (d, *^3^J_CP_* = 39.4 Hz, *CH_2_‐para*) ppm. **^31^P NMR** (162 MHz, CD_2_Cl_2_, 19°C): δ= ‐12.9 (d, *^1^J_PH_* = 544.5 Hz) ppm. **^31^P{^1^H} NMR** (162 MHz, CD_2_Cl_2_, 19°C): δ= ‐12.9 (s) ppm. **^19^F NMR** (376 MHz, CD_2_Cl_2_, 19°C): δ= ‐136.4 (dd, *J_FF_* = (23.8, 8.2) Hz, 2F, *o‐F*), ‐160.6 (td, *J_FF_* = (20.4, 5.3) Hz, 2F, *p‐F*), ‐166.6 (m, 2F, *m‐F*) ppm. **^11^B NMR** (128 MHz, CD_2_Cl_2_, 20°C): δ= ‐4.0 (s, br) ppm.

### 4.2 Synthesis of Borane‐Coordinated λ^5^‐Phosphinine (**9**)

KHMDS (10.0 mg, 0.05 mmol, 1.0 eq) in diethyl ether (0.4 mL) was added to a solution of dihydrophosphinine oxide **3/4** (38.5 mg, 0.05 mmol, 1.0 eq) in diethyl ether (1.5 mL) at *T =* ‐78°C. After 2 min, the solvent was removed. No product was isolated. Spectral data for **9**: **^31^P NMR** (162 MHz, CD_2_Cl_2_, 19°C): δ= 1.4 (d, *^1^J_PH_* = 549.2 Hz) ppm. **^31^P{^1^H} NMR** (162 MHz, CD_2_Cl_2_, 19°C): δ= 1.4 (s) ppm. **^19^F NMR** (376 MHz, CD_2_Cl_2_, 19°C): δ= ‐137.1 (d, *J_FF_* = 20.8 Hz), ‐160.6 (t, *J_FF_* = 20.3 Hz), ‐166.4 (m) ppm. **^11^B NMR** (128 MHz, CD_2_Cl_2_, 19°C): δ= ‐6.6 (s, br) ppm.

### 4.3 Synthesis of Hydrophosphination Product (**10**)

Tri‐*tert*‐butyl phosphine (8 mg, 0.04 mmol, 1.0 eq) was added to a J. Young tube containing dihydrophosphinine oxide **3/4** (31 mg, 0.04 mmol, 1.0 eq) in CH_2_Cl_2_ (0.55 mL). After 4 weeks at rt, the solution was transferred into a reaction flask with CH_2_Cl_2_ (3×0.3 mL). Single crystals suitable for X‐ray diffraction were obtained from a CH_2_Cl_2_ solution, layered with pentane at *T* = –20°C. Spectral data for **10**: **^1^H NMR** (700 MHz, THF‐d8, 25°C): δ= 11.36 (br, s, *H_2_O*), 6.13 (dtd, ^3^ *J_PH_* = 6.0 Hz, *J_HH_* = (2.9, 1.2) Hz, 1H, *H‐1*), 5.66 (q, ^3^ *J_HH_* = 2.3 Hz, 1H, *H‐2*), 2.66 (ddd, ^2^ *J_PH_* = (18.0, 11.2) Hz, *J_HH_* = 1.2 Hz, 1H, *H‐3*), 2.55 (m, 2H, *H‐4, H‐5*), 2.42 (m, 3H, *H‐4’, H‐7, H‐7’*), 2.25 (m, 1H, *H‐6’*), 2.12 (ddd, ^2^ *J_PH_* = 31.1 Hz, *J_HH_* = (14.0, 8.8) Hz, 1H, *H‐6*), 0.22 (s, 9H, *H‐8*), 0.12 (s, 9H, *H‐9*), 0.09 (s, 9H, *H‐11*), 0.08 (s, 9H, *H‐10*) ppm. **^13^C{^1^H} NMR** (150 MHz, THF‐d8, 25°C): δ= 190.1 (s, *C‐TMS‐9*), 149.9 (d, *^1^J_CF_* = 246.2 Hz, *CF‐ortho*), 143.7 (s, *C‐H5*), 141.6 (d, *^1^J_CF_* = 246.2 Hz, *CF‐para*), 138.8 (d, *^1^J_CF_* = 247.2 Hz, *CF‐meta*), 138.2 (d, *^2^J_CP_* = 20.7), 134.7 (d, *^3^J_CP_* = 17.2 Hz), 131.4 (d, *^3^J_CP_* = 10.2 Hz), 120.4 (s), 40.4 (d, *^3^J_CP_* = 43.2 Hz), 30.8 (m, *C‐H3*), 29.3 (m), 27.7 (d, *^3^J_CP_* = 52.9 Hz), –1.9 (d, *^3^J_CP_* = 20.7 Hz), –2.7 (d, *^3^J_CP_* = 6.7 Hz) ppm. **^31^P NMR** (162 MHz, CD_2_Cl_2_, 19°C): δ= 50.1 (br, s), 43.2 (br, s) ppm. **^31^P{^1^H} NMR** (162 MHz, CD_2_Cl_2_, 19°C): δ= 50.6 (d, *^2^J_PP_* = 50.3 Hz), 43.2 (d, *^2^J_PP_* = 50.3 Hz) ppm. **^19^F NMR** (376 MHz, CD_2_Cl_2_, 19°C): δ= –137.5 (dd, *J_FF_* = (24.3, 7.3) Hz, 2F, *o‐F*), –162.3 (br, s, 1F, *p‐F*), –168.6 (m, 2F, *m‐F*) ppm. **^11^B NMR** (128 MHz, CD_2_Cl_2_, 20°C): δ= –4.1 (s, br) ppm. **EI‐MS** (m/z) calculated for [C_11_H_24_PSi_2_O]^+^ [M+H]^+^ 516.2051; found: 516.2101

### 4.4 Computational Section

The DFT calculations were performed using Gaussian 16 (Rev. B.01) [44]. The geometries were optimized, and subsequent harmonic vibrational analyses were carried out at the ωB97X‐D/6‐311+G(d,p) level of theory using the default settings of Gaussian16 and utilizing the polarizable continuum implicit solvent model (PCM) with dichloromethane as solvent. The stationary points of the hydrophosphination (dimerization) reaction were optimized, and harmonic vibrational analyses were carried out at the ωB97X‐D/6‐31+G(d)(PCM = DCM) level of theory with subsequent single‐point calculations at the ωB97X‐D/6‐311+G(d,p)(PCM = DCM) level of theory. The results of the vibrational analyses were used to determine whether the found stationary point corresponds to a local minimum (no imaginary frequency) or a saddle point (one imaginary frequency, transition state) of the potential energy surface. The transition states were optimized using the force constants from a previous calculation. For every optimized transition state, intrinsic reaction coordinate calculations were carried out to identify the two corresponding minima that the transition state connects. The proton affinity values presented in the paper were also obtained at the ωB97X‐D/6‐311+G(d,p)(PCM = DCM) level of theory.

During the early phases of this study, the CREST (version 3.0) [46] program was used in combination with the GFN2‐XTB semiempirical method [45] for generating conformer ensembles with an aim to find minimum energy conformers for stationary points.

## Conflicts of Interest

The authors declare no conflicts of interest.

## Supporting information



The authors have cited additional references within the Supporting Information [47–66].

**Supporting Information File 1**: chem70781‐sup‐0002‐DataSet.zip

## Data Availability

The data that support the findings of this study are available in the supplementary material of this article.
