# Supplementary material for: Dynamic Reactivity: Reversible Reaction of a Phosphinine–Borane Adduct With Water and Double Hydrophosphination
Source: Chemistry. 2026 Feb 21;32(19):e03493. doi: 10.1002/chem.202503493 (PMC13206177; doi:10.1002/chem.202503493)
Supplement: Supplementary file 1 — The authors have cited additional references within the Supporting Information [47–66]. [file CHEM-32-e03493-s001.pdf]

Supporting Information

Dynamic Reactivity: Reversible Reaction of a Phosphinine–Borane Adduct with Water and Double Hydrophosphination

Samantha Frank,‡[a] Ádám Horváth,‡[b] Júlia Boglárka Horváth,[b] Chiara Interdonato,[a] Moritz J. Ernst,[a] Priya Kumar,[a] Zoltán Benkő,*[b,c] Christian Müller*[a]

[a] M.Sc. S. Frank, M. Sc. C. Interdonato, M.Sc. M. J. Ernst, M.Sc. P. Kumar, Prof. Dr. Dr. h.c. C. Müller, Institute of Chemistry and Biochemistry, Freie Universität Berlin, Fabeckstr. 34/36, 14195 Berlin, Germany. E-mail: c.mueller@fu-berlin.de.

[b] M. Sc. Á. Horváth, J.B. Horváth, Prof. Dr. Z. Benkő, Department of Inorganic and Analytical Chemistry, Faculty of Chemical Technology and Biotechnology, Budapest University of Technology and Economics, Műegyetem rkp. 3, H-1111, Budapest, Hungary. E-mail: benko.zoltan@vbk.bme.hu.

[c] Prof. Dr. Z. Benkő, HUN-REN-BME Computation Driven Chemistry Research Group, Műegyetem rkp. 3, H-1111, Budapest, Hungary.

‡ These authors have contributed equally.

Table of contents

1. **Experimental procedures** ... 4

 1.1 **General remarks** ... 4

 1.2 **Synthesis of starting material** ... 5

 1.3 **Synthesis of dihydrophosphinineoxide (3/4)** ... 7

 1.4 **Reactivity of dihydrophosphinineoxide (3/4)** ... 9

  1.4.1 **Deprotonation to phosphinineoxide 9** ... 9

  1.4.2 **Hydrophosphination to compound 10** ... 10

  1.4.3 **Reversibility with ^{t}BuOK** ... 11

  1.4.4 **Control experiments** ... 12

 1.5 **Experimental spectra** ... 12

  1.5.1 **Dihydrophosphinineoxide 3/4** ... 12

  1.5.2 **High temperature NMR spectra** ... 20

  1.5.3 **Deprotonation of 3/4 to species 9** ... 24

  1.5.4 **Hydrophosphination to compound 10** ... 26

  1.5.5 **Reversibility with ^{t}BuOK** ... 30

2. **Crystallographic Data** ... 33

3. **DFT calculations** ... 36

 3.1 **Computed NMR chemical shifts** ... 36

 3.2 **Formal insertion of the P atom into the H-O bond of H_2O** ... 37

 3.3 **1,2- and 1,4-additions** ... 40

 3.4 **Investigation on the hydrophosphination reaction** ... 45

 3.5 **Optimized geometries (in xyz format) and electronic energies (in a. u.)** ... 46

  3.5.1 **General** ... 46

  3.5.2 **NMR chemical shift calculations** ... 54

  3.5.3 **Formal insertion of the P atom into the H-O bond of H_2O** ... 57

  3.5.4 **1,2- and 1,4-additions** ... 80

3.5.5 Deprotonation 108
3.5.6 Hydrophosphination reaction 116
4. References 130

1. Experimental procedures

1.1 General remarks

Unless otherwise stated, all reactions and workups were performed following standard SCHLENK techniques or using an argon filled *Unilab* glovebox by *MBraun* (H_2O < 0.1 ppm, O_2 < 0.1 ppm). Room temperature refers to T = 23 °C, while elevated reaction temperatures are referred to the respective oil bath's temperature. Solvents and reagents were degassed using the *freeze-pump-thaw* method (three cycles) or by purging with argon. Dry toluene, dichloromethane and *n*-pentane were collected from a solvent purification system, *MB SPS-800* by *MBraun*, and additionally stored over 3 Å molecular sieves prior to use. Dry diethyl ether was stirred over Na, distilled and degassed. CD_2Cl_2 and tiethylamine were dried over CaH_2, distilled, and degassed. PCl_3 was refluxed and distilled prior to use. A stock solution of H_2O in CH_2Cl_2 was prepared by adding 0.05 mL H_2O (degassed) to 12 mL CH_2Cl_2. All other commercially available chemicals were used without further purification. Preparative, inert filtration over dried silica gel (particle size: 0.040-0.063 mm, surface area: 500 m^2/g) by *Sigma-Aldrich* as the stationary phase under an argon atmosphere. ^1H, $^{13}C\{^1H\}$, ^{31}P, $^{31}P\{^1H\}$, ^{19}F and ^{11}B NMR spectra were recorded on *JEOL* (ECX 400, Lamba 400, ECP 500, ECZ 600) or *Bruker* (AVANCE 500, AVANCE 700) spectrometers. NMR chemical shifts are referenced to the IUPAC standards.[47,48] Quantitative ^{31}P NMR spectroscopic measurements were carried out using an internal triphenylphosphine standard. ESI mass spectra were measured with an Agilent 6210 ESI-TOF, Agilent Technologies, Santa Clara, CA, USA. The flow rate was set at 4 µL · min^{-1} and the spray voltage at 4 kV. The desolvation gas was set at 15 psi (1 bar). All other parameters were optimized for a maximum abundance of the respective $[M+H]^+$. EI mass spectra were measured on a MAT 711, Varian MAT, Bremen. Electron Energy for EI was set to 80 eV. NMR and mass spectra were analyzed and displayed with the software MestReNova 7.1.2 by Mestrelab Research. IR spectra were measured at rt under ambient atmosphere with a Nicolet iS 10 FT-IR spectrometer and displayed with the software OriginPro 2022b.

1.2 Synthesis of starting material

4,6-Di-*tert*-butyl-1,3,2-diazaphosphinine

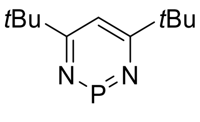

C

$C_{11}H_{19}N_2P$
M = 210.26 g/mol

Diazaphosphinine was syntheseized according to a modified literature procedure. Pivalonitrile (6.3 mL, 56.8 mmol, 3.0 eq) was added to a solution of diemethyltitanocene (0.47 M in toluene, 40 mL, 18.9 mmol) at rt. The reaction mixture was stirred at T = 80 °C for 24 h. The dark red suspension was filtrated over Celite with toluene (3 x 3 mL). Quantitative conversion was assumed. To the solution, triethylamine (7.9 mL, 56.8 mmol, 3.0 eq) was added at T = –20 °C. Subsequently, PCl_3 (1.7 mL, 18.9 mmoL, 1.0 eq) was added dropwise into the solution. The dark yellow suspension was stirred at rt for 1 h and filtrated over Celite with toluene (3 x 3 mL). Diazaphosphinine was obtained as a dark yellow solution in toluene (0.09 M, 58 mL, 9.28 mmol, 59%).

^{31}P NMR (162 MHz, toluene): δ = 267.8 ppm.

The spectroscopic data is consistent with the literature.[49]

2,3,5,6-Tetrakis(trimethylsilyl)-phosphinine

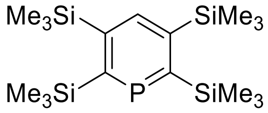

$C_{17}H_{37}PSi_4$
M = 384.7968 g/mol

Diazaphosphinine (0.09 M, 9.28 mmol, 1.0 eq) and bis(trimethylsilyl)acetylene (6.3 mL, 27.8 mmol, 3.0 eq) were warmed up to T = 130 °C in a pressure tube for three days. After one day, additional bis(trimethylsilyl)acetylene (4.2 mL, 18.6 mmol, 2.0 eq) was added to the reaction solution. After three days, the solvent was removed at T = 40 °C, the residue was suspended in pentane (10 mL) and filtrated over dry silica (7 cm) with pentane (250 mL). The solvent of the colorless filtrate was removed in high vacuum. 2,3,5,6-Tetrakis(trimethylsilyl)-phosphinine was isolated as a colourless, crystalline solid (2.2 g, 5.6 mmol, 60%).

^{1}**H NMR** (400 MHz, CD_2Cl_2, 19 °C): δ = 8.12 (d, $^4J_{PH}$ = 1.8 Hz, 1H, ***p*-H**), 0.46 (d, $^4J_{PH}$ = 1.9 Hz, 18H, ***o*-SiMe₃**), 0.40 (s, 18H, ***m*-SiMe₃**) ppm.

^{31}P{^{1}H} NMR (162 MHz, CD_2Cl_2, 21 °C): δ = 266.3 (s) ppm.

3,5-Bis(trimethylsilyl)-phosphinine (1)

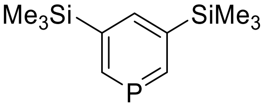

$C_{11}H_{21}PSi_2$
M = 240.4328 g/mol

HCl in diethyl ether (2 M in Et_2O, 8.9 mL, 18 mmol, 4.8 eq) was added to a solution of 2,3,5,6-tetrakis(trimethylsilyl)-phosphinine (1.4 g, 3.7 mmol, 1.0 eq) in diethyl ether (37 mL). The bright yellow solution was stirred at rt for 16 h. The solvent was removed in high vacuum and the yellow oil was diluted in pentane and filtrated over dry silica (4 cm) with pentane (200 mL). The solvent of the colourless filtrate was removed in static vacuum and 3,5-bis(trimethylsilyl)-phosphinine was obtained as a colourless oil (721 mg, 3.0 mmol, 81%).

^{1}**H NMR** (400 MHz, CD_2Cl_2, 19 °C): δ = 9.04 (dd, $^2J_{PH}$ = 38.4 Hz, $^4J_{HH}$ = 0.7 Hz, 2H, ***o*-H**), 7.49 (dt, $^4J_{PH}$ = 3.7 Hz, $^4J_{HH}$ = 0.7 Hz, 1H, ***p*-H**), 0.34 (s, 18H, ***SiMe₃***) ppm.

^{31}P{^{1}H} NMR (162 MHz, CD_2Cl_2, 21 °C): δ = 200.6 (s) ppm.

^{31}P NMR (162 MHz, CD_2Cl_2, 21 °C): δ = 200.6 (dt, $^2J_{PH}$ = 38.4 Hz, $^4J_{PH}$ = 3.7 Hz) ppm.

3,5-Bis(trimethylsilyl)phosphinine-B(C_6F_5)$_3$ Lewis pair (2)

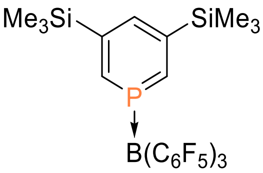

$C_{29}H_{21}BF_{15}PSi_2$
M = 752.4168 g/mol

Tris(pentafluorophenyl)borane (21 mg, 0.0416 mmol, 1.0 eq) was added to a solution of 3,5-bis(trimethylsilyl)phosphinine (10 mg, 0.0416 mmol, 1.0 eq) in CH_2Cl_2. Quantitative conversion to the Lewis pair occurred immediately.

^{1}**H** (400 MHz, CD_2Cl_2, 19 °C): δ = 8.37 (m, 2H, ***o*-H**), 8.03 (d, $^{4}J_{PH}$ = 9.22 Hz, 1H, ***p*-H**), 0.33 (s, 18H, ***SiMe₃***) ppm.

^{31}P{^{1}H} NMR (162 MHz, CD_2Cl_2, 21 °C): δ = 171.5 (s) ppm.

^{31}P NMR (162 MHz, CD_2Cl_2, 21 °C): δ = 171.5 (br, s) ppm.

^{19}F NMR (376 MHz, CD_2Cl_2, 23 °C): δ = −129.3 (d, J_{FF} = 15.3 Hz, 6F, ***o-F)***, −156.1 (t, J_{FF} = 20.2 Hz, 3F, ***p-F)***, −163.7 (ddd, J_{FF} = (23.9, 20.2, 8.44) Hz, 6F, ***m-F)***, ppm.

^{11}B NMR (128 MHz, CD_2Cl_2, 23 °C): δ = −10.4 (br, s) ppm.

1.3 Synthesis of dihydrophosphinineoxide (3/4)

Method A:

Water (in CH_2Cl_2, *c* = 0.078 mmol/mL, 1.64 mL, 1.0 eq) was added to a solution of 3,5-bis($SiMe_3$) phosphinine (**1**) (0.125 mmol, 30 mg, 1.0 eq) and tris(pentafluorophenyl)borane (0.125 mmol, 64 mg, 1.0 eq) in CH_2Cl_2 (1 mL). After stirring at rt for 24 hours, the solvent was removed in high vacuum at *T* = 50 °C. Dihydrophosphinineoxide **3/4** was obtained as a colourless oil (96 mg, quant.). Crystals suitable for X-ray diffraction were obtained from a toluene solution, layered with pentane at rt.

Table S1: Isomeric ratio depending on equivalents of H_2O for synthesis.

H_2O [eq]	*ortho* isomer 3	*para* isomer 4	conversion[a] [%]
1.0	80	20	quant
2.0	78	22	79
3.0	64	36	88
4.0	59	41	50
5.0	56	44	62

[a]determined with quantitative ^{31}P NMR spectroscopy of the reaction solution after 24 h

Method B:

Water (in CH_2Cl_2, c = 0.078 mmol/mL, 1.64 mL, 1.0 eq) was added to a solution of tris(pentafluorophenyl)borane (0.125 mmol, 64 mg, 1.0 eq) in CH_2Cl_2 (0.5 mL). A solution of 3,5-bis($SiMe_3$) phosphinine (**1**) (0.125 mmol, 30 mg, 1.0 eq) in pentane (0.3 mL) was added. dihydrophosphinineoxide **3/4** was not isolated (70:30 ortho/para ratio, 89% conversion).

ortho-1,2-Dihydrophosphinineoxide **3**

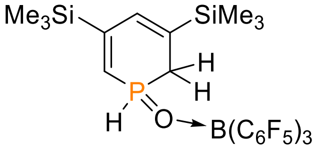

$C_{29}H_{23}BF_{15}OPSi_2$
M = 770,4318 g/mol

^{1}H NMR (600 MHz, CD_2Cl_2, 25 °C): δ = 7.54 and 6.66 (d, $^{1}J_{PH}$ = 533.0 Hz, 1H, ***P-H***), 6.68 (dd, J = 5.5, 2.4 Hz, 1H, ***CH-para***), 6.36 (d, J_{PH} = 23.5 Hz, 1H, ***CH-ortho***), 3.22 (t, J_{PH} = 20.1 Hz, 1H, ***CH₂***), 2.70-2.65 (m, 1H, ***CH₂***), 0.18 (s, 9H, ***SiMe₃***), 0.17 (s, 9H, ***SiMe₃***) ppm.

^{13}C{^{1}H} NMR (150 MHz, CD_2Cl_2, 25 °C): δ = 168.2 (d, $^{2}J_{CP}$ = 5.8 Hz, ***C-SiMe₃***), 149.0 (d, $^{1}J_{CF}$ = 239.8 Hz, ***CF-ortho***), 141.0 (d, $^{1}J_{CF}$ = 245.5 Hz, ***CF-para***), 139.7 (d, $^{2}J_{CP}$ = 14.6 Hz, ***C-SiMe₃***), 138.2 (d, $^{1}J_{CF}$ = 247.8 Hz, ***CF-meta***), 133.0 (d, $^{3}J_{CP}$ = 36.81 Hz, ***CH-para***), 113.9 (d, $^{1}J_{CP}$ = 88.2 Hz, ***CH-ortho***), 24.1 (d, $^{1}J_{CP}$ = 75.1 Hz, ***CH₂***), 1.1 (s, ***Si(CH₃)₃***), 0.8 (s, ***Si(CH₃)₃***) ppm.

^{31}P NMR (162 MHz, CD_2Cl_2, 19 °C): δ = 7.7 (d, $^{1}J_{PH}$ = 528.4 Hz) ppm.

^{31}P{^{1}H} NMR (162 MHz, CD_2Cl_2, 19 °C): δ = 7.7 (s) ppm.

^{19}F NMR (376 MHz, CD_2Cl_2, 19 °C): δ = –136.6 (d, J_{FF} = 19.6 Hz, 2F, ***o-F***), –160.6 (td, J_{FF} = (20.3, 5.5) Hz, 1F, ***p-F***), –166.5 (m, 2F, ***m-F***) ppm.

^{11}B NMR (128 MHz, CD_2Cl_2, 20 °C): δ = –4.0 (s, br) ppm.

IR (ATR, rt, 4 cm^{-1}): $\tilde{\nu}$ = 2959 (w, br), 2903 (vw, br), 2361 (w, br, ν_{PH}) 2343 (vw, br, ν_{PH}), 1644 (m, $\nu_{C=C}$), 1515 (s, $\nu_{C=C}$), 1462 (vs, $\nu_{C=C}$), 1374 (w, $\nu_{C=C}$), 1283 (m, δ_{TMS}), 1253 (m, δ_{TMS}), 1095 (s, δ_{PH}), 971 (s, δ_{PH}, δ_{BC}), 837 (vs, δ_{TMS}) cm^{-1}.

ESI-MS (m/z) calculated for $[C_{11}H_{24}PSi_2O]^+$ $[M+H]^+$ 259.1103; found: 259.1130. Calculated for $[C_{11}H_{23}PSi_2ONa]^+$ $[M+Na]^+$ 281.0923; found: 281.0943

Para-1,4-Dihydrophosphinineoxide **4**

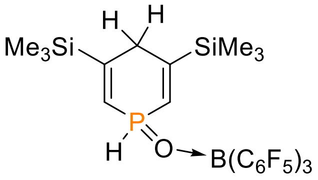

$C_{29}H_{23}BF_{15}OPSi_2$
M = 770,4318 g/mol

^{1}H NMR (600 MHz, CD_2Cl_2, 25 °C): δ = 7.76 and 6.85 (d, $^1J_{PH}$ = 544.0 Hz, 1H, ***P-H***), 6.47 (td, J_{PH} = 17.3 Hz, J_{HH} = 1.8 Hz, 2H, ***CH-ortho***), 3.36 (ddt, J = (7.1, 3.4, 1.8) Hz, 1H, ***CH₂-para***), 3.32 (ddt, J = (7.0, 3.5, 1.8) Hz, 1H, ***CH₂-para***), 3.20-3.13 (m, 1H, ***CH₂-para***), 0.19 (s, 18H, ***SiMe₃***) ppm.

^{13}C{^{1}H} NMR (150 MHz, CD_2Cl_2, 25 °C): δ = 176.9 (d, $^2J_{CP}$ = 5.3 Hz, ***C-SiMe₃***), 149.0 (d, $^1J_{CF}$ = 239.8 Hz, ***CF-ortho***), 141.0 (d, $^1J_{CF}$ = 245.5 Hz, ***CF-para***), 138.2 (d, $^1J_{CF}$ = 247.8 Hz, ***CF-meta***), 116.9 (d, $^2J_{CP}$ = 91.2 Hz, ***CH-ortho***), 34.9 (d, $^3J_{CP}$ = 39.4 Hz, ***CH₂-para***) ppm.

^{31}P NMR (162 MHz, CD_2Cl_2, 19 °C): δ = –12.9 (d, $^1J_{PH}$ = 544.5 Hz) ppm.

^{31}P{^{1}H} NMR (162 MHz, CD_2Cl_2, 19 °C): δ = –12.9 (s) ppm.

^{19}F NMR (376 MHz, CD_2Cl_2, 19 °C): δ = –136.4 (dd, J_{FF} = (23.8, 8.2) Hz, 2F, ***o-F***), –160.6 (td, J_{FF} = (20.4, 5.3) Hz, 2F, ***p-F***), –166.6 (m, 2F, ***m-F***) ppm.

^{11}B NMR (128 MHz, CD_2Cl_2, 20 °C): δ = –4.0 (s, br) ppm.

1.4 Reactivity of dihydrophosphinineoxide (3/4)

1.4.1 Deprotonation to phosphinineoxide 9

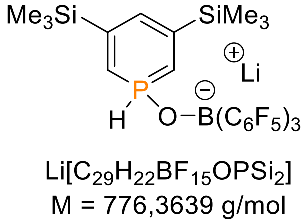

$Li[C_{29}H_{22}BF_{15}OPSi_2]$
M = 776,3639 g/mol

KHMDS (10.0 mg, 0.05 mmol, 1.0 eq) in diethylether (0.4 mL) was added to a solution of dihydrophosphinineoxide **3/4** (38.5 mg, 0.05 mmol, 1.0 eq) in diethylether (1.5 mL) at T = –78 °C. After 2 minutes, the solvent was removed. No product was isolated. NMR spectra were measured from the diethylether solution with the option of *no solvent*.

Table S2: Reaction conditions for the deprotonation of **3/4**.

**solvent**	***T* [°C]**	**base (1.0 eq)**	**result**
CH_2Cl_2	rt	KHMDS	side reactions
Et_2O	–78	KHMDS	deprotonation
Et_2O	–78	MeLi (c = 1.45 M)	decomposition
pentane	–78	MeLi (c = 0.3 M)	decomposition
toluene	rt	NEt_3	side reaction
toluene	–78	KHMDS	deprotonation
Et_2O	–78→rt	KH	decomposition
Et_2O	–78	LDA (c = 2 M)	free phosphinine

^{31}P NMR (162 MHz, Et_2O, 19 °C): δ = 1.4 (d, $^1J_{PH}$ = 547 Hz) ppm.

^{31}P{^{1}H} NMR (162 MHz, Et_2O, 19 °C): δ = 1.4 (s) ppm.

^{19}F NMR (376 MHz, Et_2O, 19 °C): δ = –137.1 (d, J_{FF} = 20.8 Hz), –160.6 (t, J_{FF} = 20.3 Hz), –166.4 (m) ppm.

^{11}B NMR (128 MHz, Et_2O, 19 °C): δ = –6.6 (s, br) ppm.

1.4.2 Hydrophosphination to compound 10

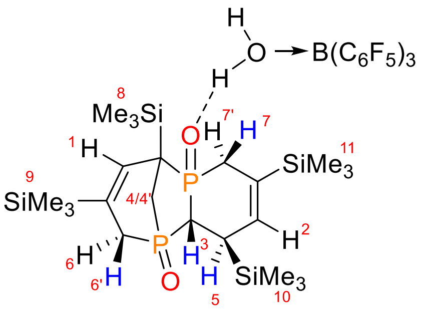

$C_{40}H_{48}O_3P_2Si_4BF_{15}$
M = 1046,8945 g/mol

Tri-*tert*-butyl phosphine (8 mg, 0.04 mmol, 1.0 eq) was added to a J. Young tube containing dihydrophosphinine oxide **3/4** (31 mg, 0.04 mmol, 1.0 eq) in CH_2Cl_2 (0.55 mL). After 4 weeks at rt, the solution was transferred into a reaction flask with CH_2Cl_2 (3 x 0.3 mL). Single crystals

suitable for X-ray diffraction were obtained from a CH_2Cl_2 solution, layered with pentane at T = –20 °C (isolated yield as single crystals: 0.013 mmol, 14 mg, 33%).

^{1}H NMR (700 MHz, THF-d8, 25 °C): δ = 11.36 (br, s, ***H₂O***), 6.13 (dtd, $^3J_{PH}$ = 6.0 Hz, J_{HH} = (2.9, 1.2) Hz, 1H, ***H-1***), 5.66 (q, $^3J_{HH}$ = 2.3 Hz, 1H, ***H-2***), 2.66 (ddd, $^2J_{PH}$ = (18.0, 11.2) Hz, J_{HH} = 1.2 Hz, 1H, ***H-3***), 2.55 (m, 2H, ***H-4, H-5***), 2.42 (m, 3H, ***H-4', H-7, H-7'***), 2.25 (m, 1H, ***H-6'***), 2.12 (ddd, $^2J_{PH}$ = 31.1 Hz, J_{HH} = (14.0, 8.8) Hz, 1H, ***H-6***), 0.22 (s, 9H, ***H-8***), 0.12 (s, 9H, ***H-9***), 0.09 (s, 9H, ***H-11***), 0.08 (s, 9H, ***H-10***) ppm.

^{13}C{^{1}H} NMR (150 MHz, THF-d8, 25 °C): δ = 190.1 (s, ***C-SiMe₃-9***), 149.9 (d, $^1J_{CF}$ = 246.2 Hz, ***CF-ortho***), 143.7 (s, ***C-H5***), 141.6 (d, $^1J_{CF}$ = 246.2 Hz, ***CF-para***), 138.8 (d, $^1J_{CF}$ = 247.2 Hz, ***CF-meta***), 138.2 (d, $^2J_{CP}$ = 20.7), 134.7 (d, $^3J_{CP}$ = 17.2 Hz), 131.4 (d, $^3J_{CP}$ = 10.2 Hz), 120.4 (s), 40.4 (d, $^3J_{CP}$ = 43.2 Hz), 30.8 (m, ***C-H3***), 29.3 (m), 27.7 (d, $^3J_{CP}$ = 52.9 Hz), –1.9 (d, $^3J_{CP}$ = 20.7 Hz), –2.7 (d, $^3J_{CP}$ = 6.7 Hz) ppm.

^{31}P NMR (162 MHz, CD_2Cl_2, 19 °C): δ = 50.1 (br, s), 43.2 (br, s) ppm.

^{31}P{^{1}H} NMR (162 MHz, CD_2Cl_2, 19 °C): δ = 50.6 (d, $^2J_{PP}$ = 50.3 Hz), 43.2 (d, $^2J_{PP}$ = 50.3 Hz) ppm.

^{19}F NMR (376 MHz, CD_2Cl_2, 19 °C): δ = –137.5 (dd, J_{FF} = (24.3, 7.3) Hz, 2F, ***o-F***), –162.3 (br, s, 1F, ***p-F***), –168.6 (m, 2F, ***m-F***) ppm.

^{11}B NMR (128 MHz, CD_2Cl_2, 20 °C): δ = –4.1 (s, br) ppm.

EI-MS (m/z) calculated for $[C_{11}H_{24}PSi_2O]^+$ $[M+H]^+$ 516.2051; found: 516.2101

1.4.3 Reversibility with ^{t}BuOK

1,2 Dihydrophosphinine oxide **3/4** (35 mg, 0.046 mmol, 1.0 eq) was dissolved in CH_2Cl_2 (1.5 mL). ^{t}BuOK (5 mg, 0.046 mmol, 1.0 eq) was added. After 5 minutes, the solvent was removed in high vacuum and the residue was filtrated over SiO_2 (1 cm) with pentane (15 mL). The solvent was removed in high vacuum and 3,5-bis($SiMe_3$)-phosphinine (**1**) was obtained as a pale yellow oil.

NMR spectra measured before work-up:

^{1}H NMR (400 MHz, CD_2Cl_2, 23 °C): δ = 9.06 (d, $^2J_{PH}$ = 38.2 Hz, 2H, ***o-H***), 7.91 (d, $^4J_{PH}$ = 3.5 Hz, 1H, ***p-H***), 1.30 (s, 9H, ***^{t}Bu***), 0.31 (s,18H, ***SiMe₃***) ppm.

^{31}P{^{1}H} NMR (162 MHz, CD_2Cl_2, 23 °C): δ = 200.4 (s) ppm.

^{31}P{^{1}H} NMR (162 MHz, CD_2Cl_2, 23 °C): δ = 200.4 (td, $^2{}_{PH}$ = 38.9 Hz, $^4J_{PH}$ = 3.5 Hz) ppm.

^{19}**F NMR** (376 MHz, CD_2Cl_2, 20 °C): δ = –140.3 (m, 2F, ***o-F***), –155.3 (t, J_{FF} = 21.6 Hz, 1F, ***p-F***), –163.5 (m, 2F, ***m-F***) ppm.

^{11}**B NMR** (128 MHz, CD_2Cl_2, 17 °C): δ = 15.8 (s, br) ppm.

ESI-MS (m/z) calculated for $[OH(B(C_6F_5)_3]^-$ $[M]^-$ 528.9881; found:528.9909. Calculated for $[^tBuOB(C_6F_5)_3]^-$ $[M]^-$ 585.0507; found: 585.0539.

1.4.4 Control experiments

Reaction of uncoordinated phosphinine 1 with water

Water (in CH_2Cl_2, *c* = 0.078 mmol/mL, 0.55 mL, 1.0 eq) was added to a solution of 3,5-bis($SiMe_3$) phosphinine (**1**) (0.0416 mmol, 10 mg, 1.0 eq) in CH_2Cl_2 (0.1 mL). No reaction was observed spectroscopically.

1.5 Experimental spectra

1.5.1 Dihydrophosphinineoxide 3/4

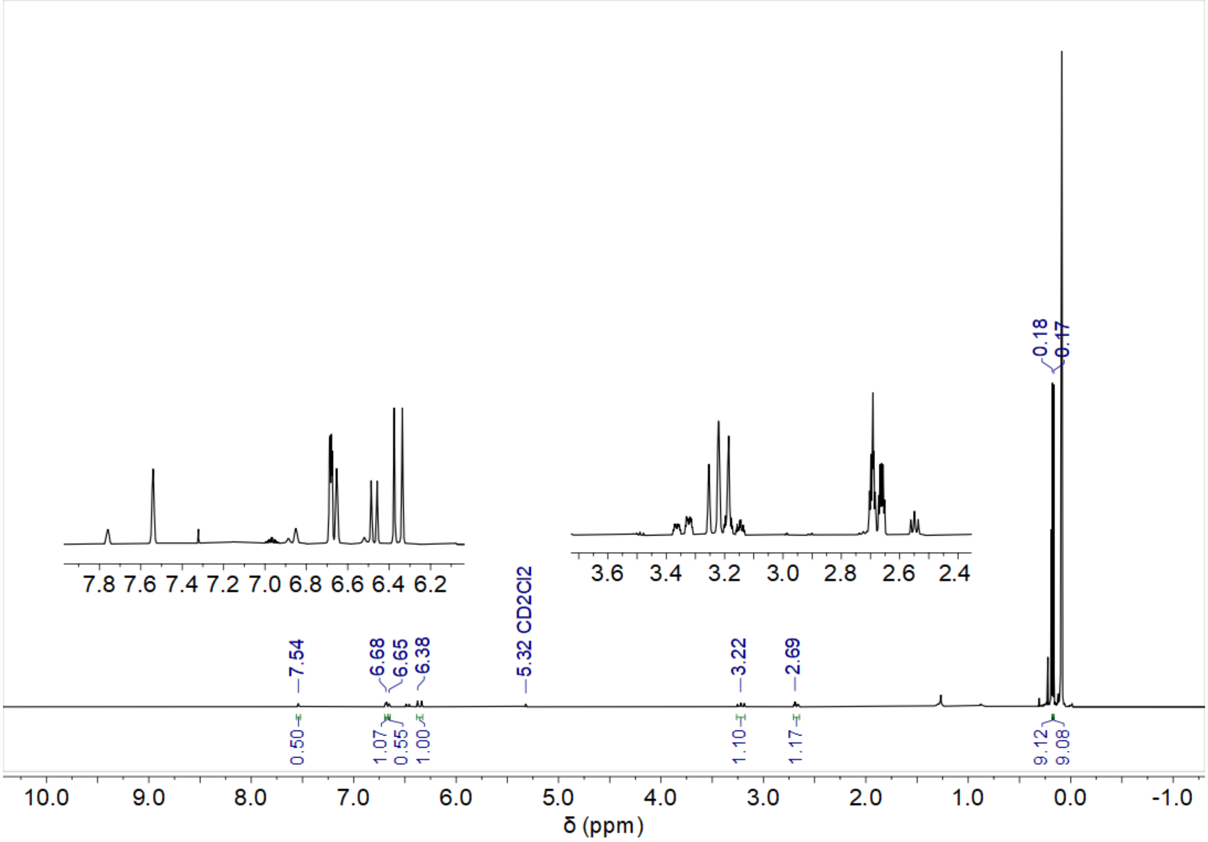

Figure S1: ^{1}H NMR spectrum containing integrated resonances of *ortho*-1,2-dihydrophosphinineoxide **3**.

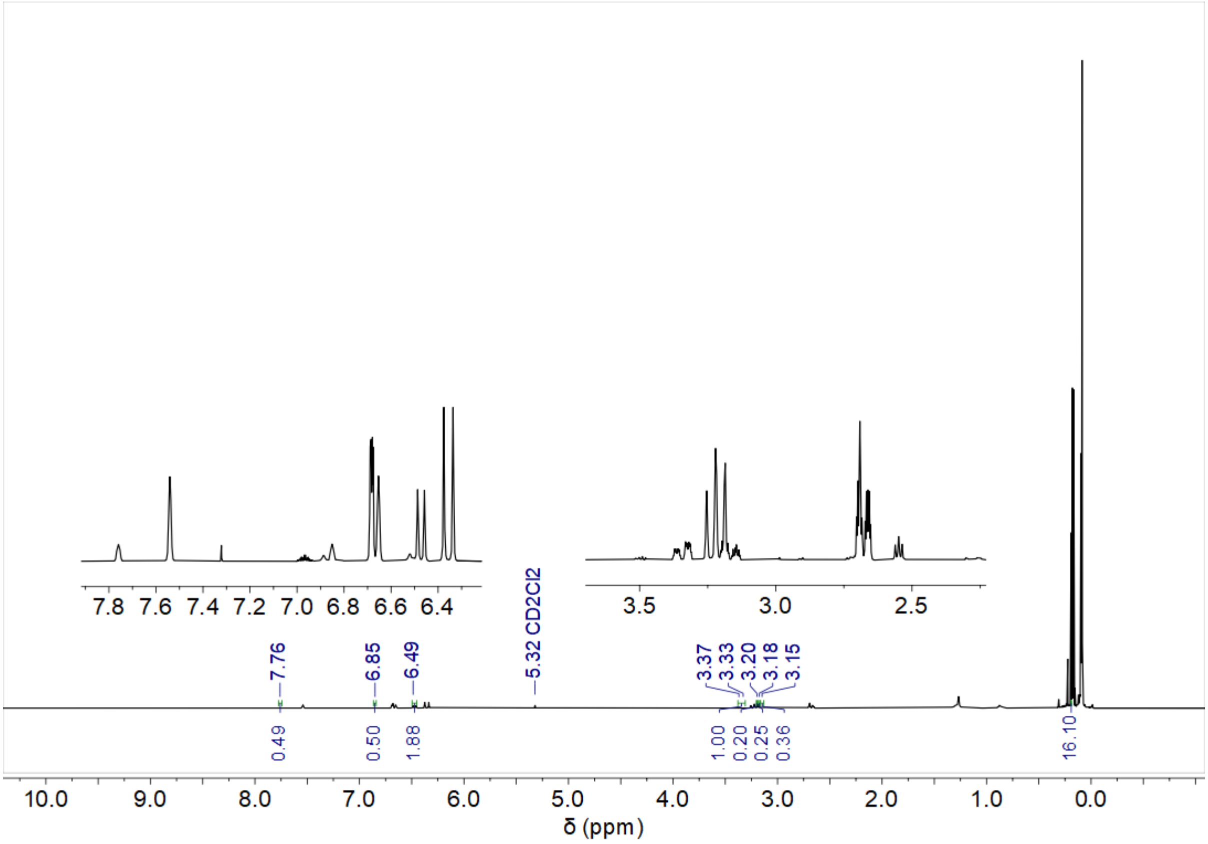

Figure S2: ^{1}H NMR spectrum containing integrated resonances for *para*-1,4-dihydrophosphinineoxide **4**.

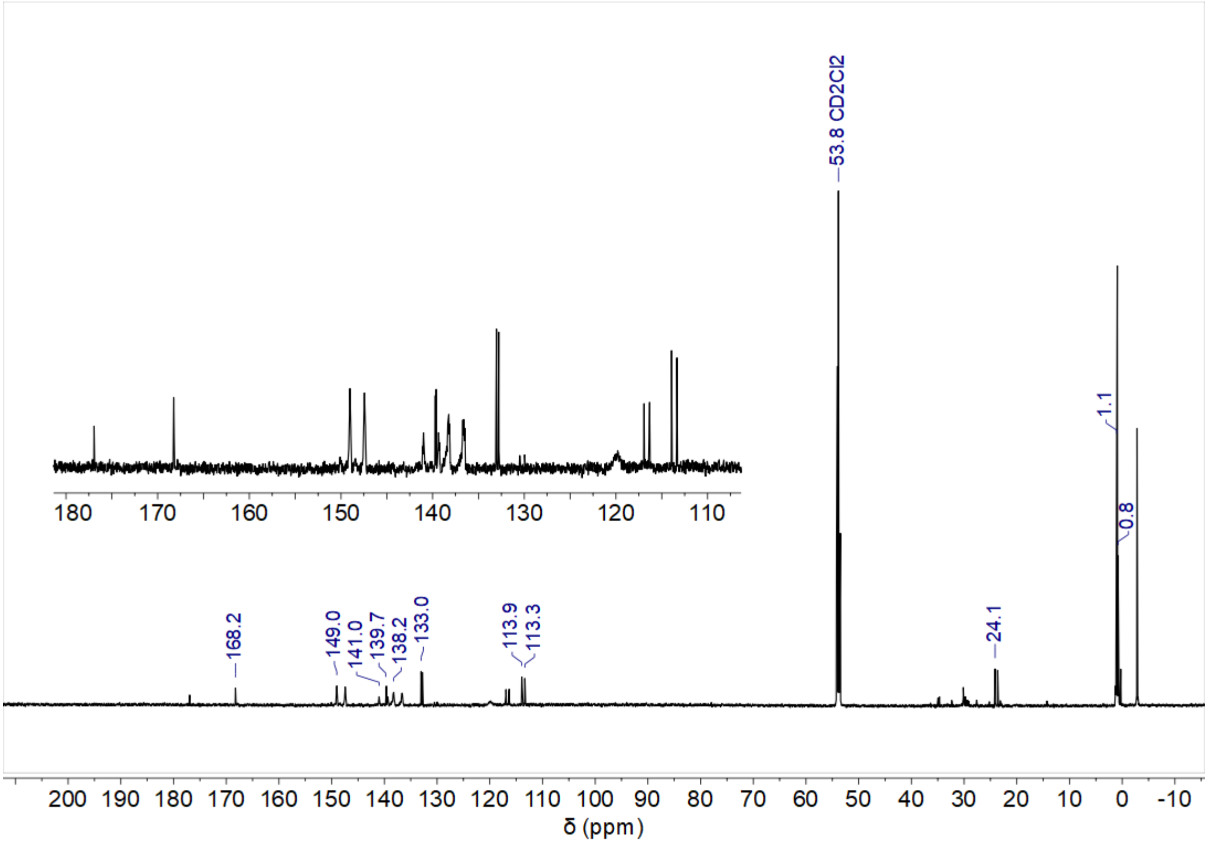

, **Figure S3:** $^{13}C\{^{1}H\}$ NMR spectrum containing marked resonances of *ortho*-1,2-dihydrophosphinineoxide **3**.

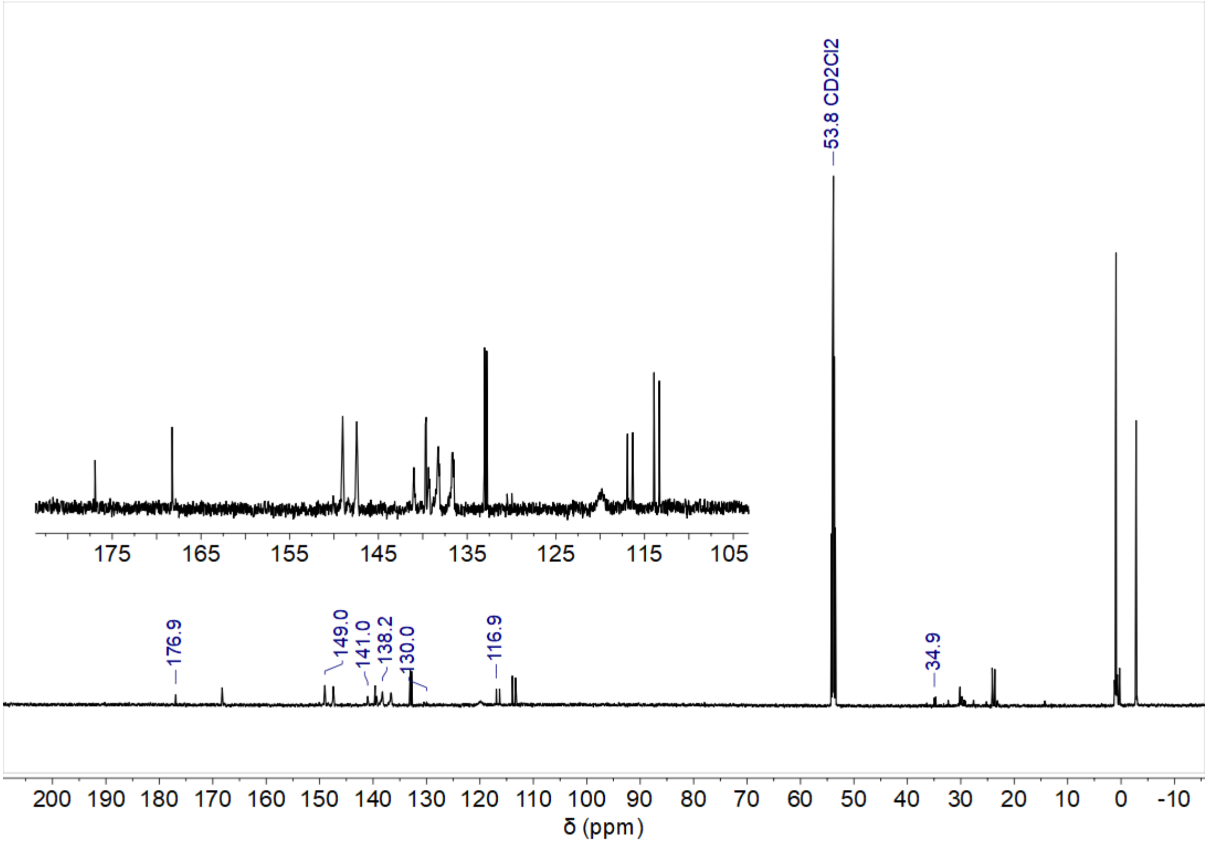

Figure S4: $^{13}C\{^{1}H\}$ NMR spectrum containing marked resonances of *para*-1,4-dihydrophosphinineoxide **4**.

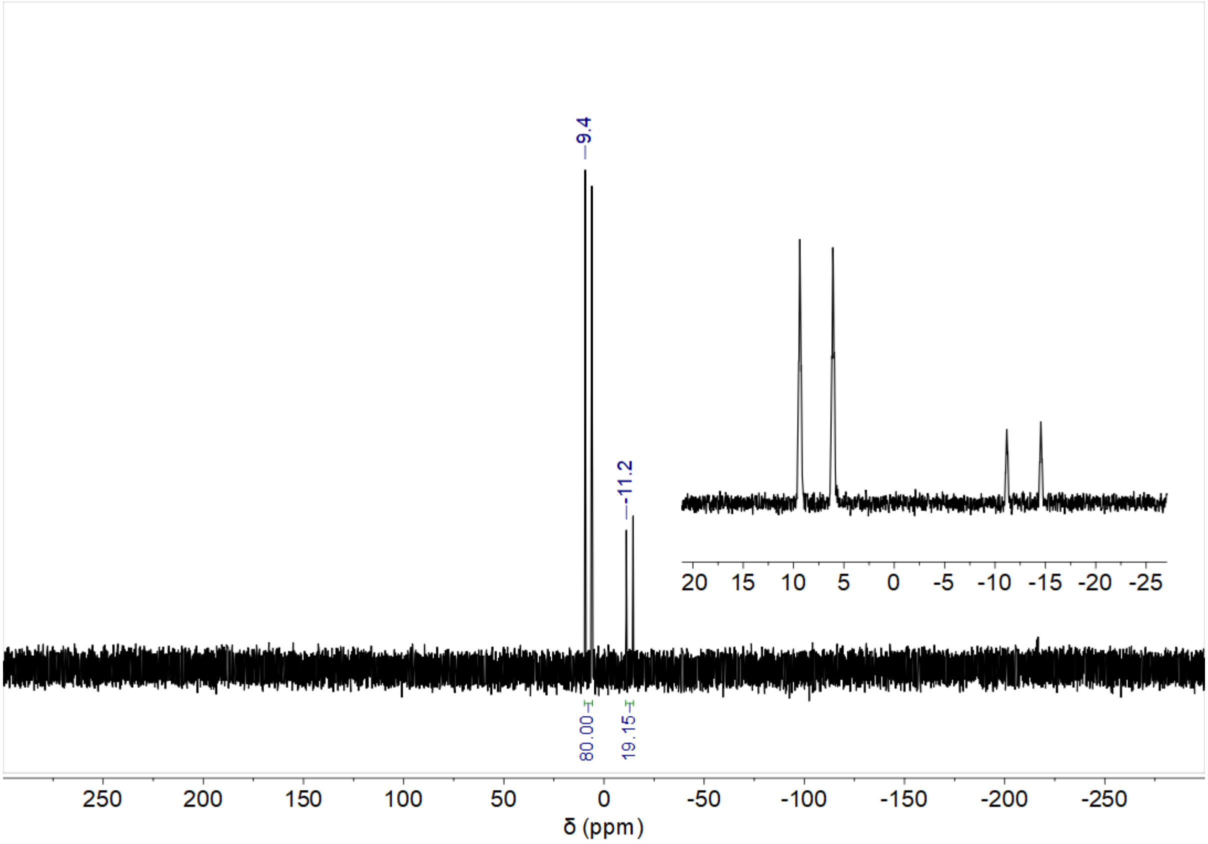

Figure S5: ^{31}P NMR spectrum of dihydrophosphinineoxide **3/4**.

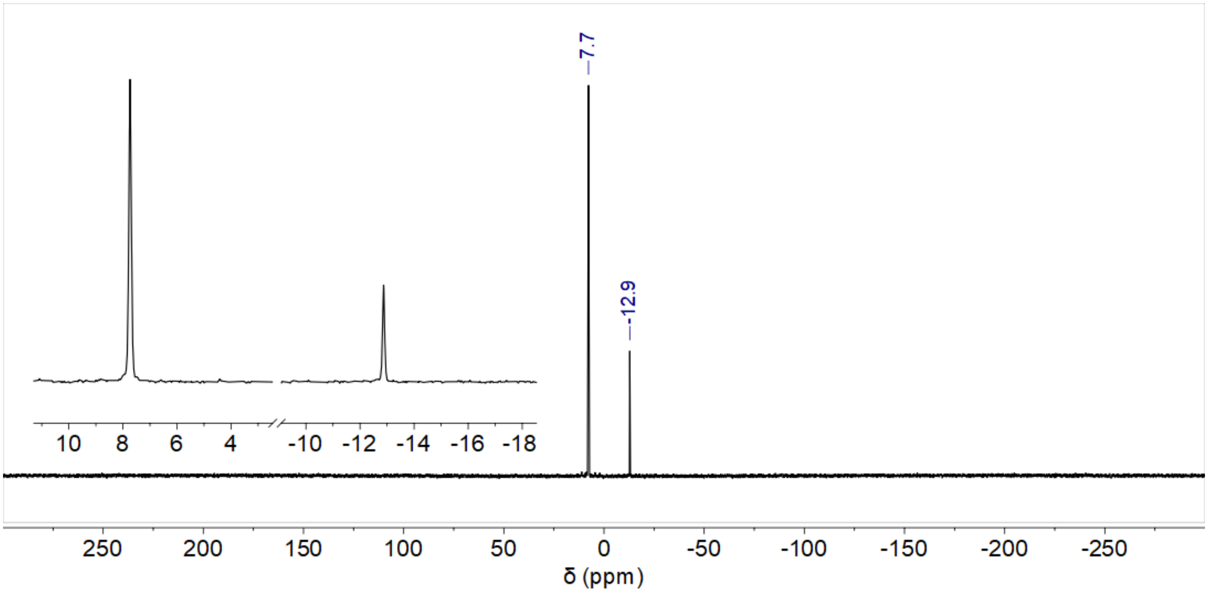

Figure S6: ^{31}P{^{1}H} NMR spectrum of dihydrophosphinineoxide **3/4**.

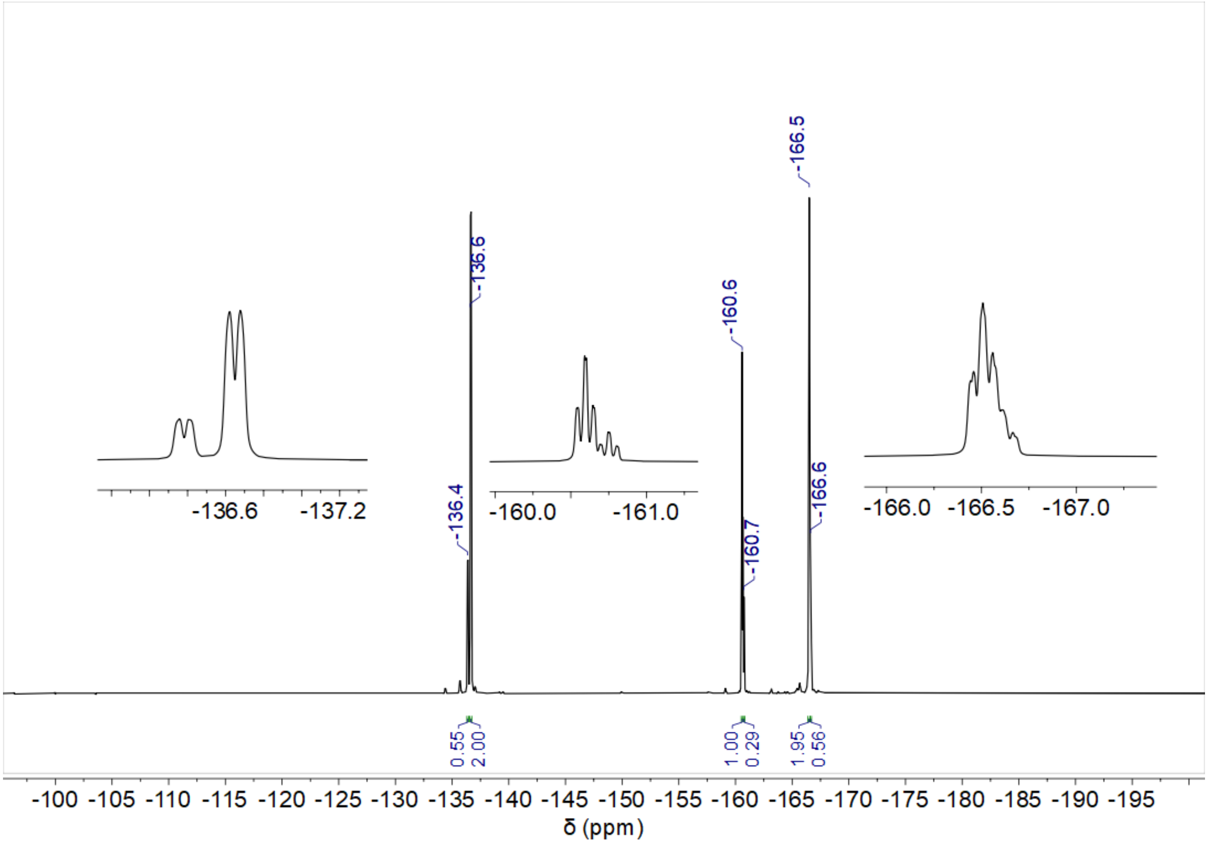

Figure S7: ^{19}F NMR spectrum of dihydrophosphinineoxide **3/4**.

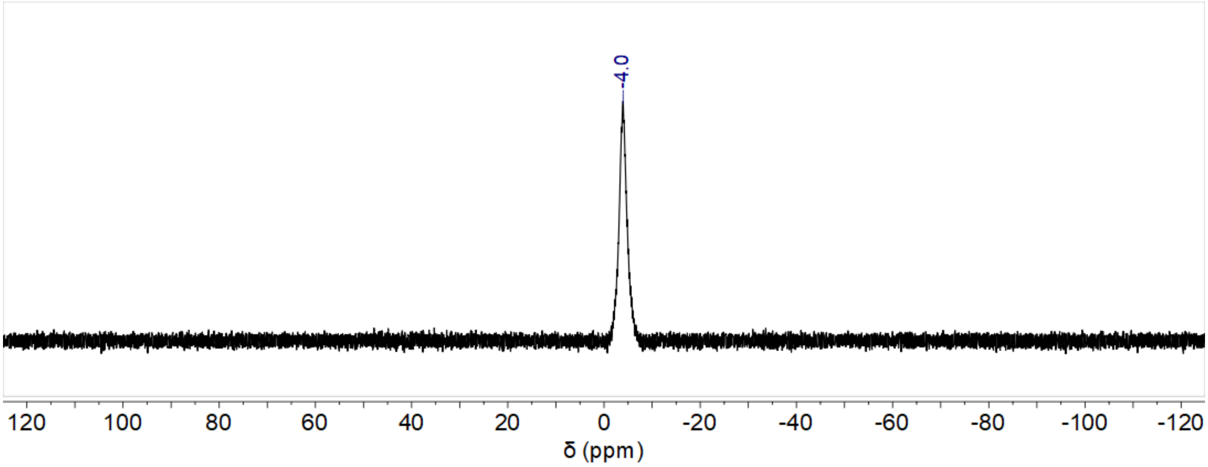

Figure S8: ^{11}B NMR spectrum of dihydrophosphinineoxide **3/4**.

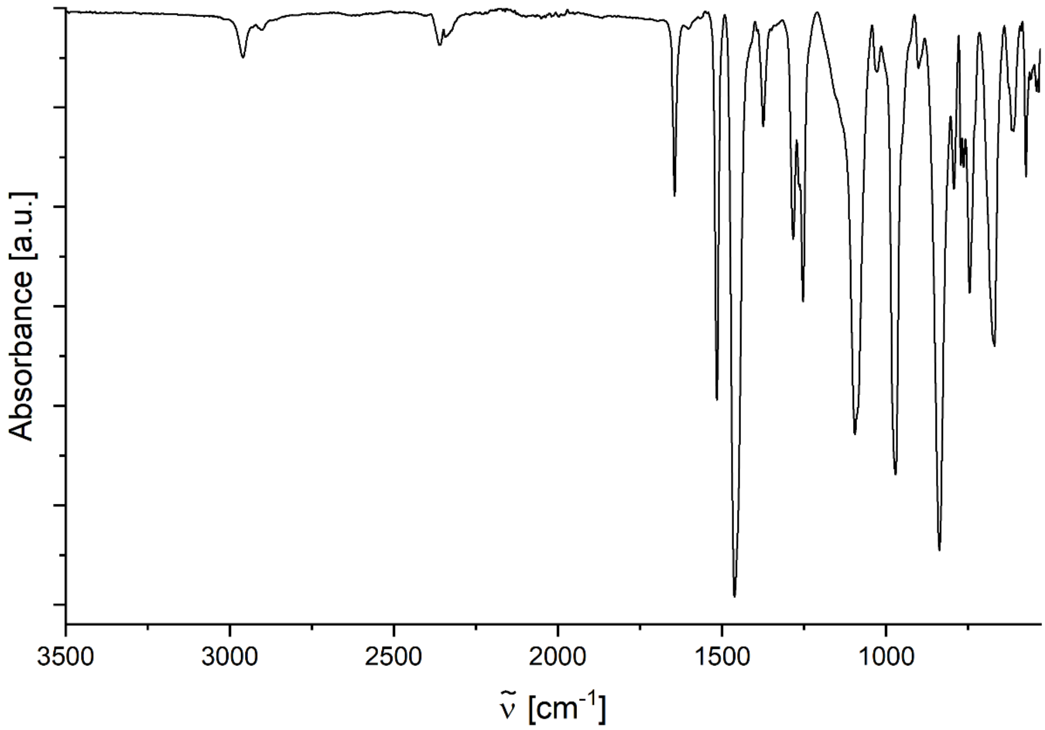

Figure S9: IR spectrum of dihydrophosphinineoxide **3/4**.

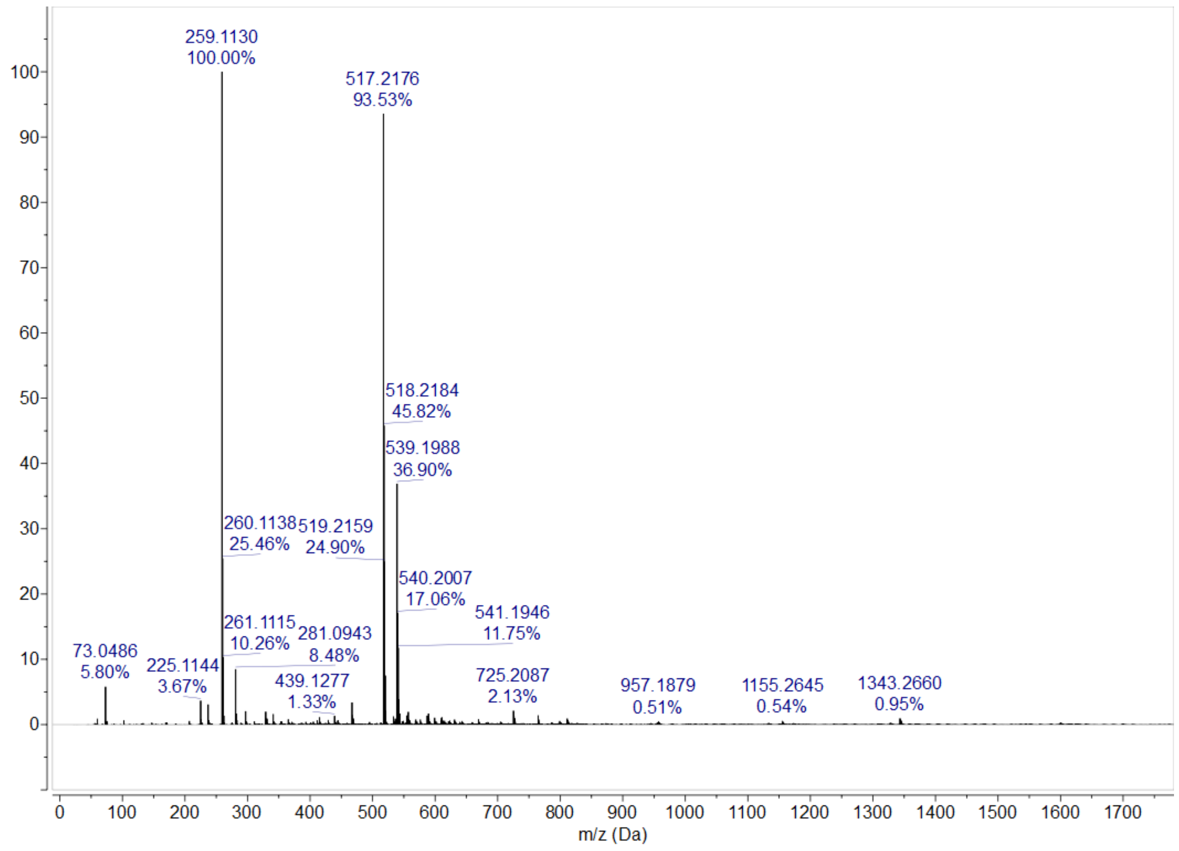

Figure S10: ESI+ (MeOH) of dihydrophosphinineoxide **3/4**.

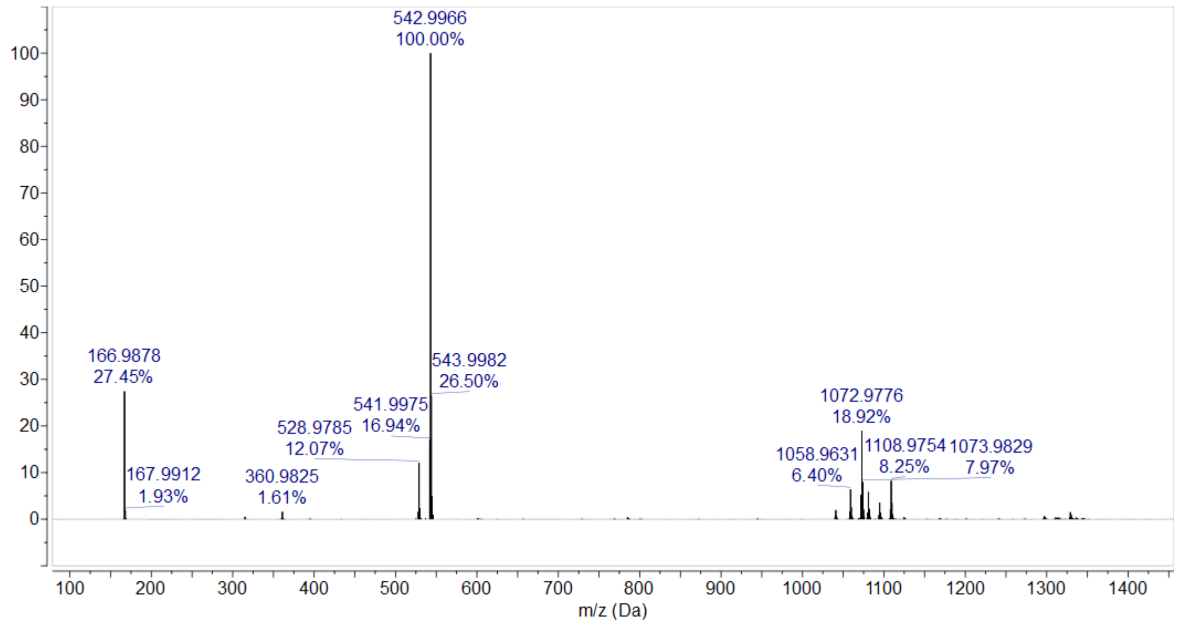

Figure S11: ESI– (MeOH) of dihydrophosphinineoxide **3/4**.

1.5.2 High temperature NMR spectra

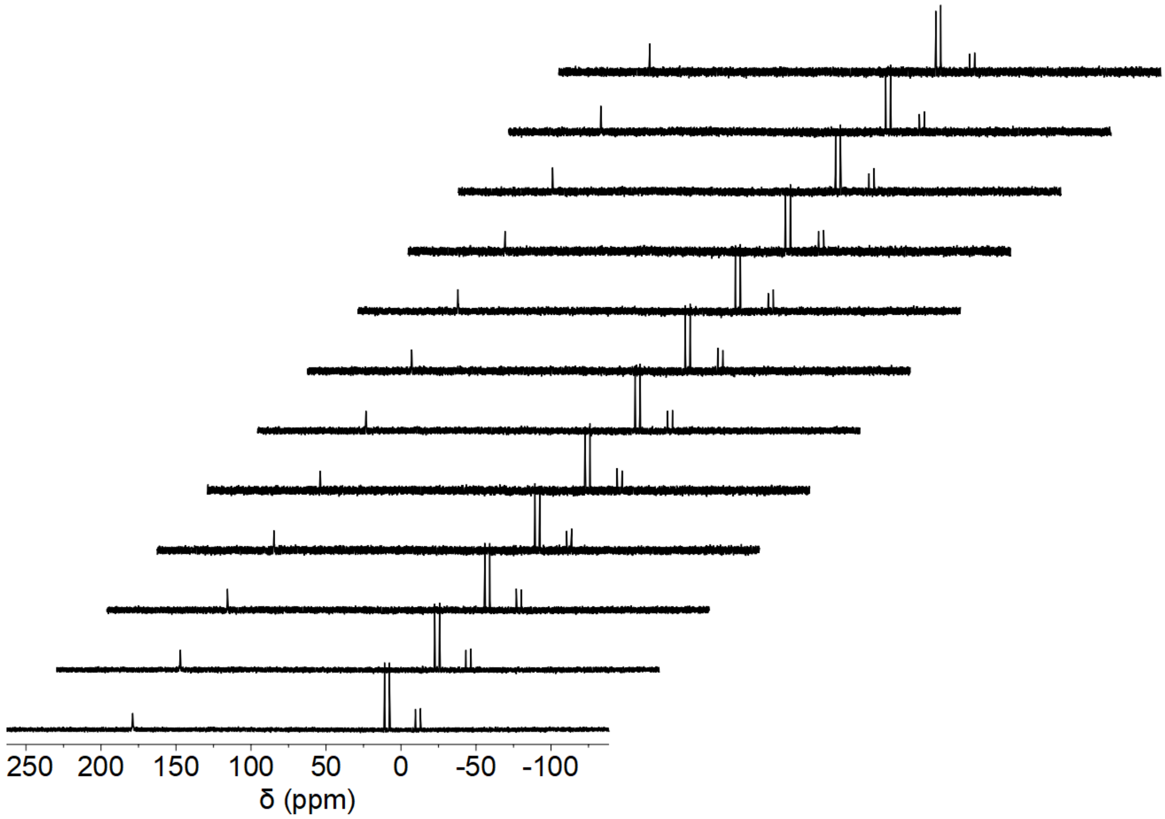

Figure S12: High temperature ^{31}P NMR spectra (1,2-dichlorobenzene-d_4) of dihydrophosphinine oxide **3/4** (and residual phosphinine/borane Lewis pair **2**) at *T* = 30 °C (bottom) in 10 °C steps.

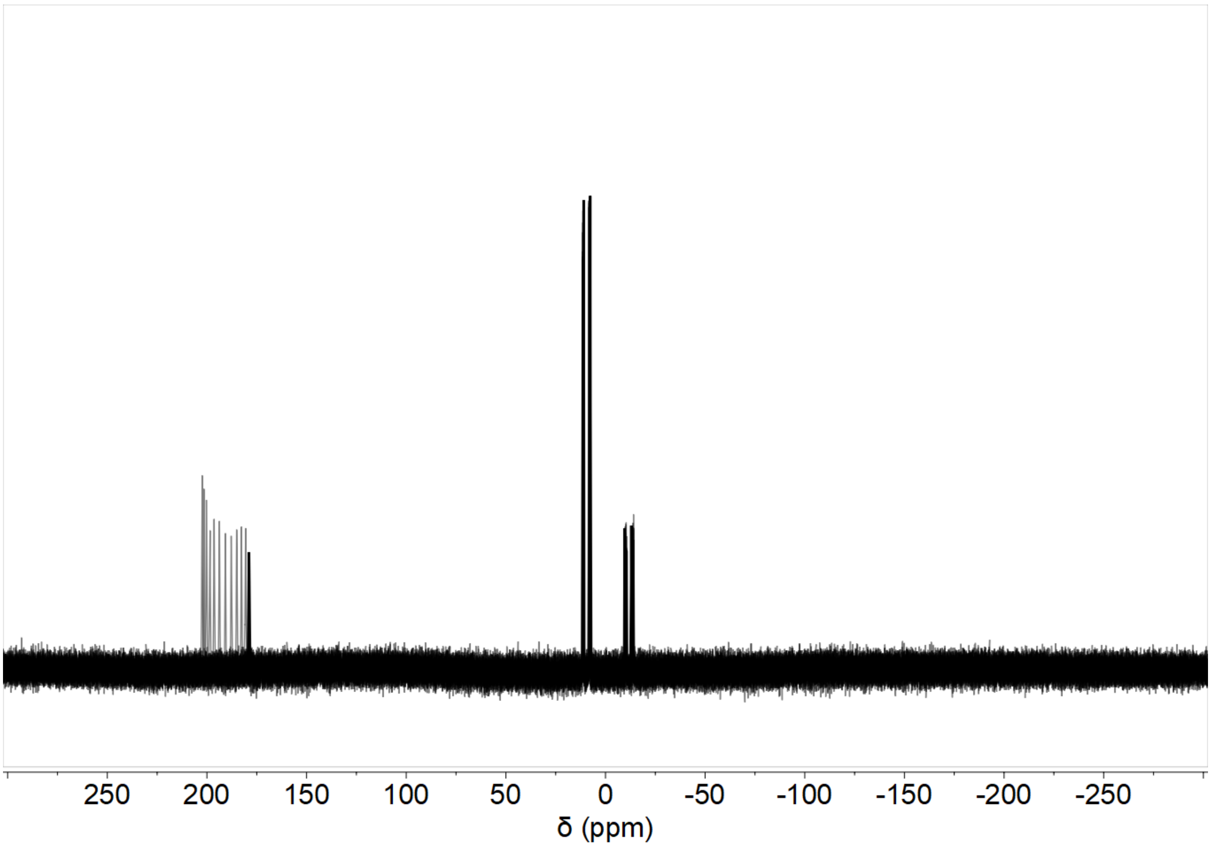

Figure S13: High temperature ^{31}P NMR spectra superimposed (1,2-dichlorobenzene-d$_4$) of dihydrophosphinine oxide **3/4** (and residual phosphinine/borane Lewis pair **2**) at T = 30 °C (black) in 10 °C steps (grey).

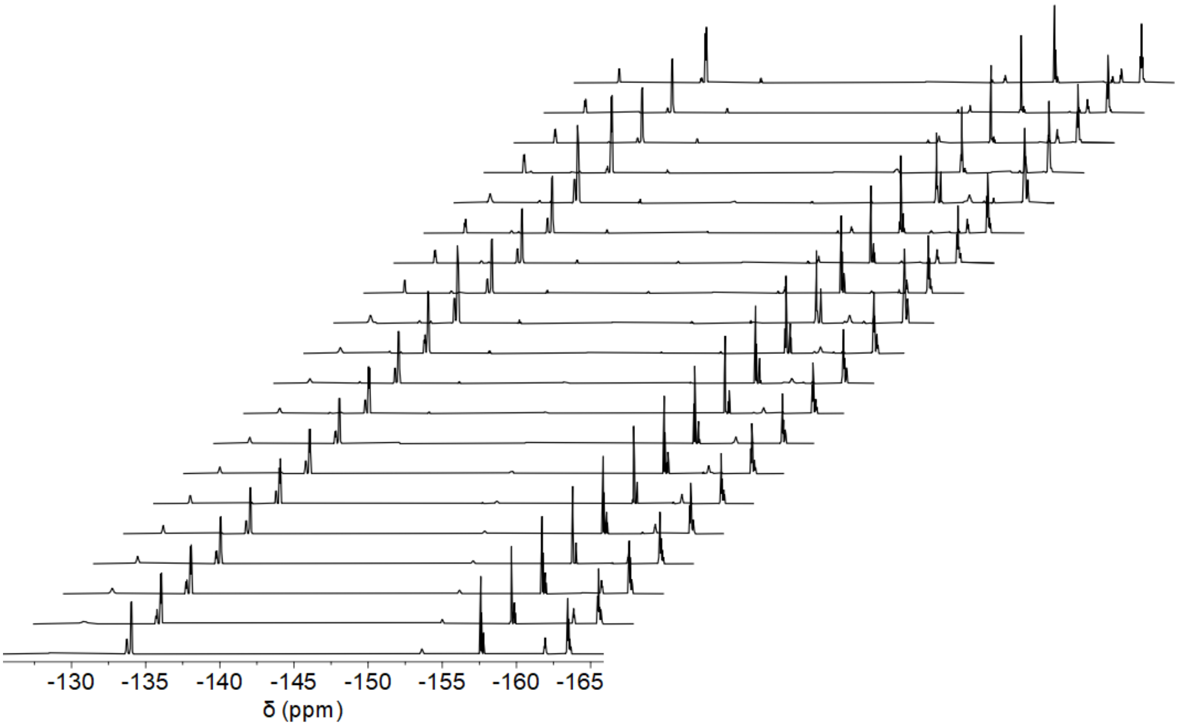

Figure S14: High temperature ^{19}F NMR spectra (1,2-dichlorobenzene-d_4) of dihydrophosphinine oxide **3/4** (and residual phosphinine/borane Lewis pair **2**) at *T* = 30 °C (bottom) in 10 °C steps.

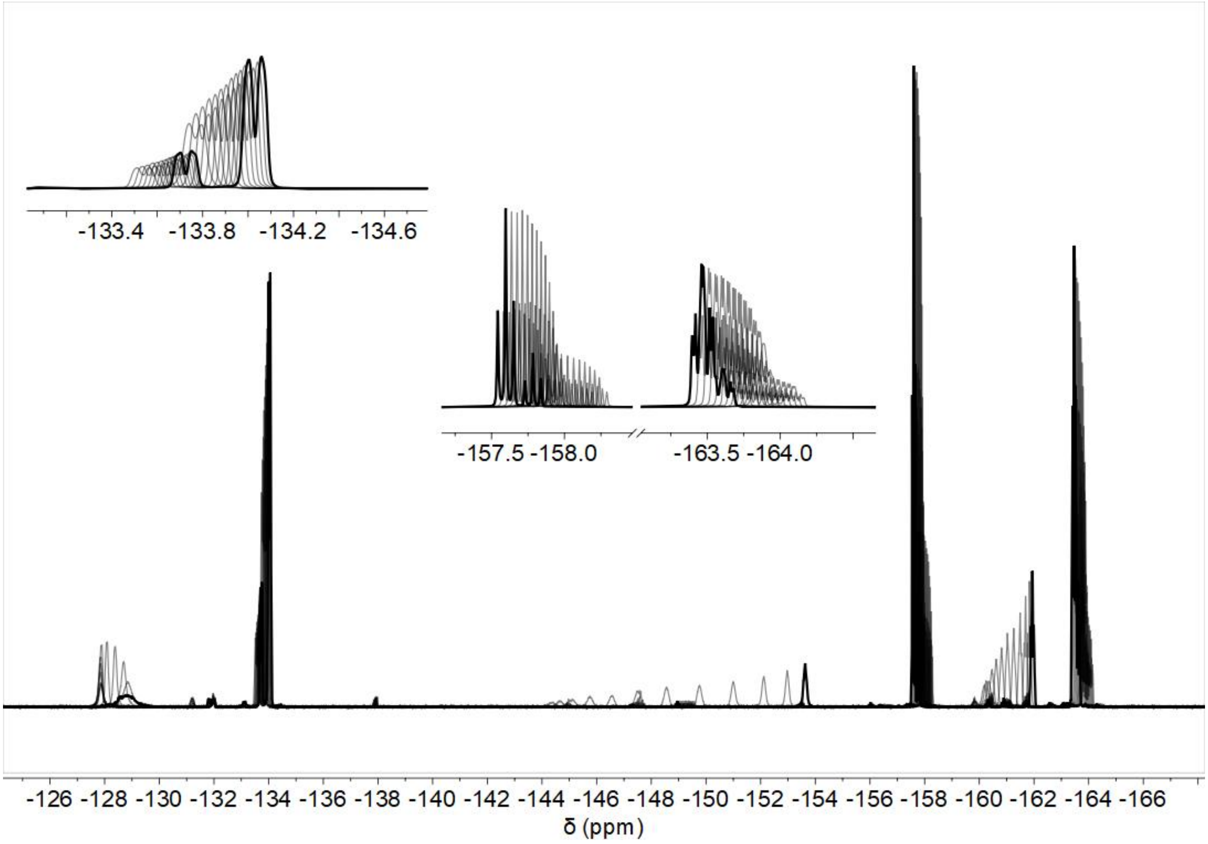

Figure S15: High temperature ^{19}F NMR spectra superimposed (1,2-dichlorobenzene-d_4) of dihydrophosphinine oxide **3/4** (and residual phosphinine/borane Lewis pair **2**) at *T* = 30 °C (black) in 10 °C steps (grey).

1.5.3 Deprotonation of 3/4 to species 9

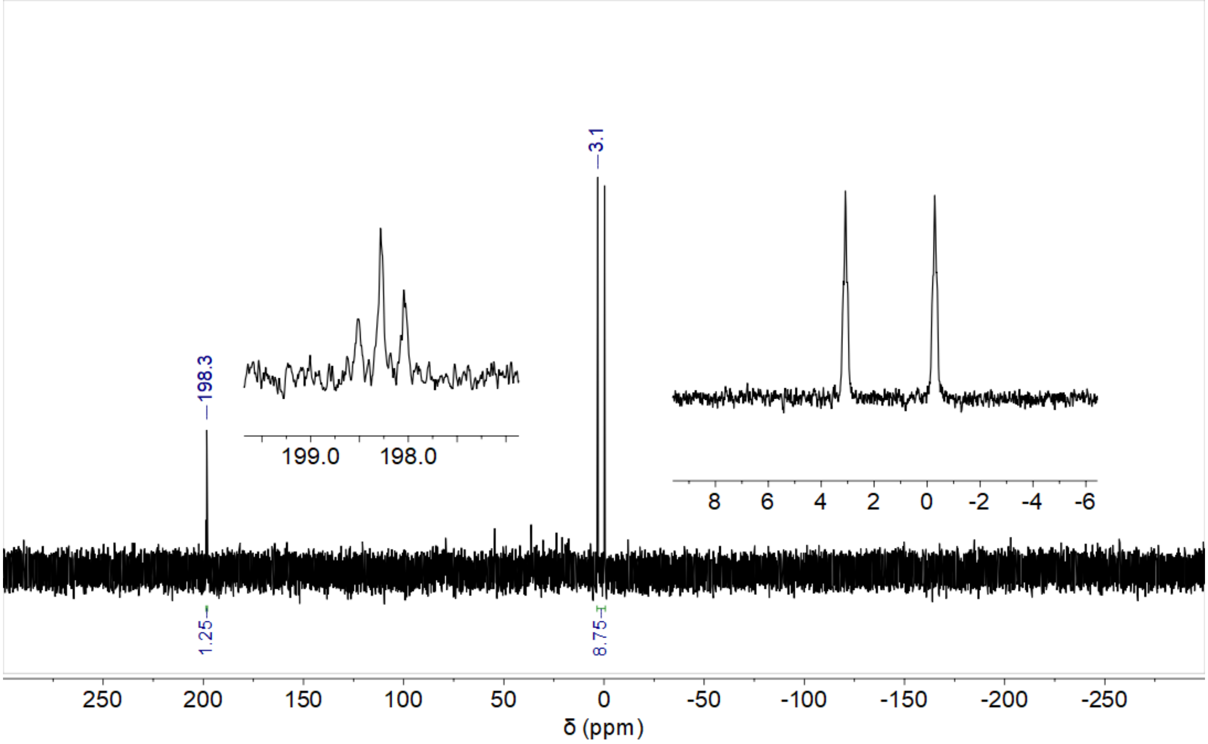

Figure S16: ^{31}P NMR spectrum (Et_2O) of deprotonated dihydrophosphinineoxide **9**.

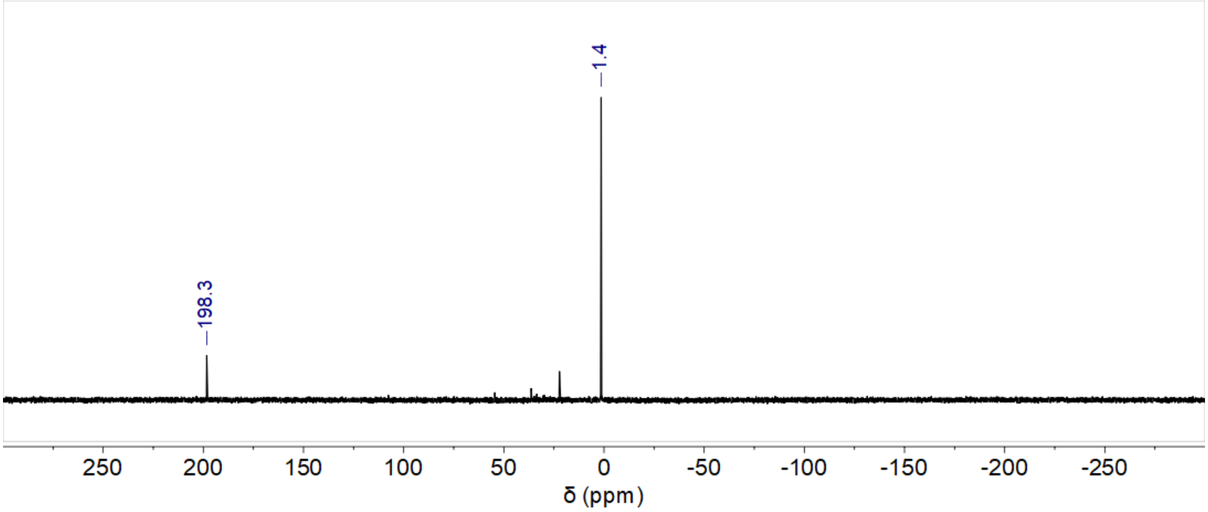

Figure S17: $^{31}P\{^1H\}$ NMR spectrum (Et_2O) of deprotonated dihydrophosphinineoxide **9**.

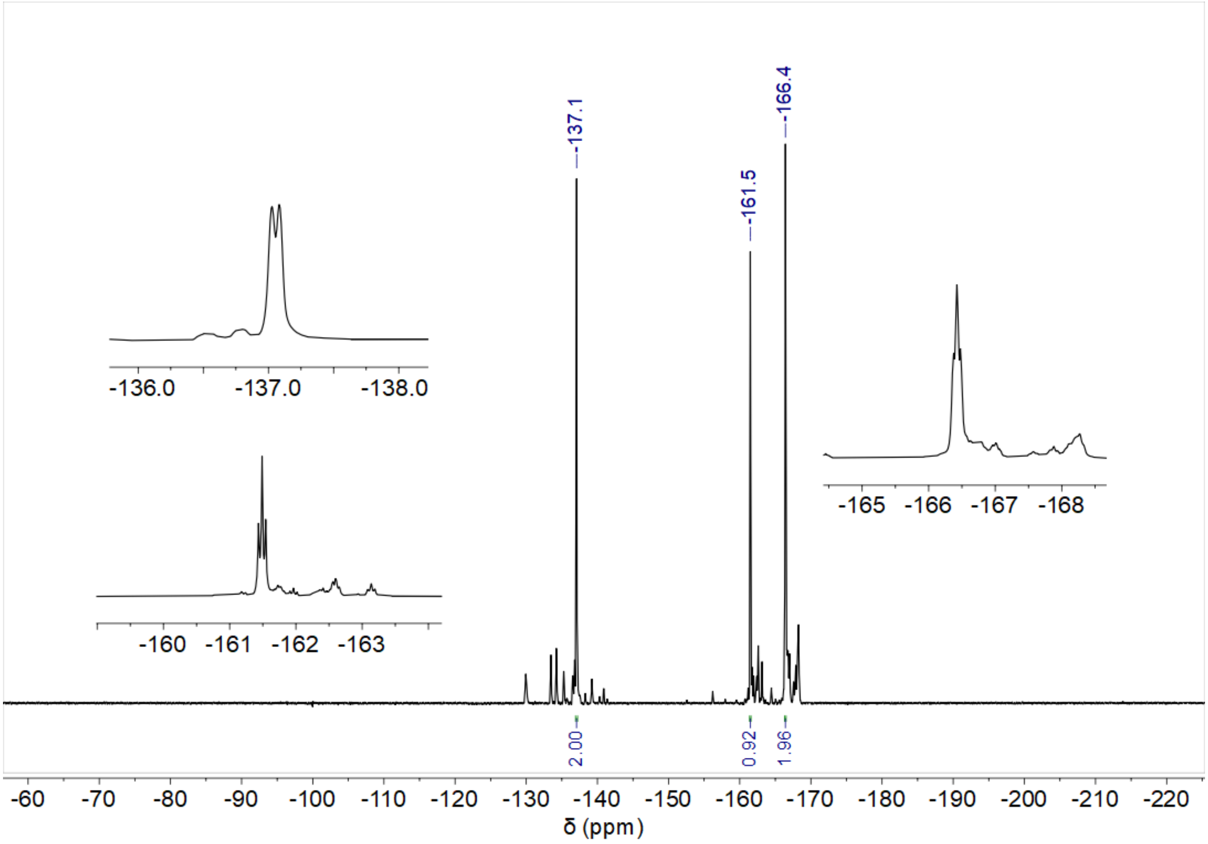

Figure S18: ^{19}F NMR spectrum (Et_2O) of deprotonated dihydrophosphinineoxide **9**.

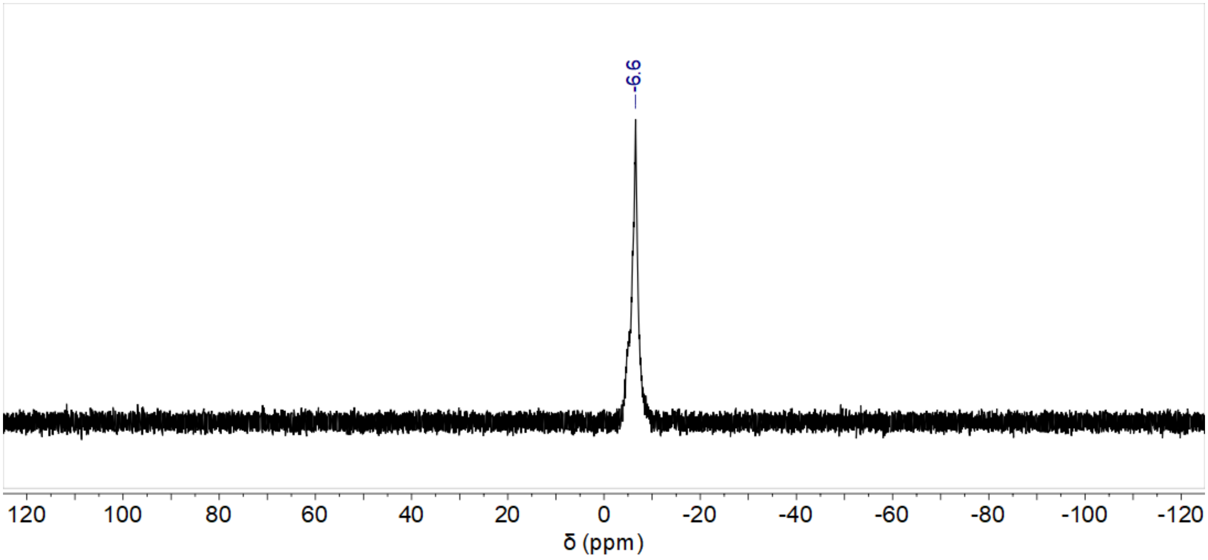

Figure S19: ^{11}B NMR spectrum (Et_2O) of deprotonated dihydrophosphinineoxide **9**.

1.5.4 Hydrophosphination to compound 10

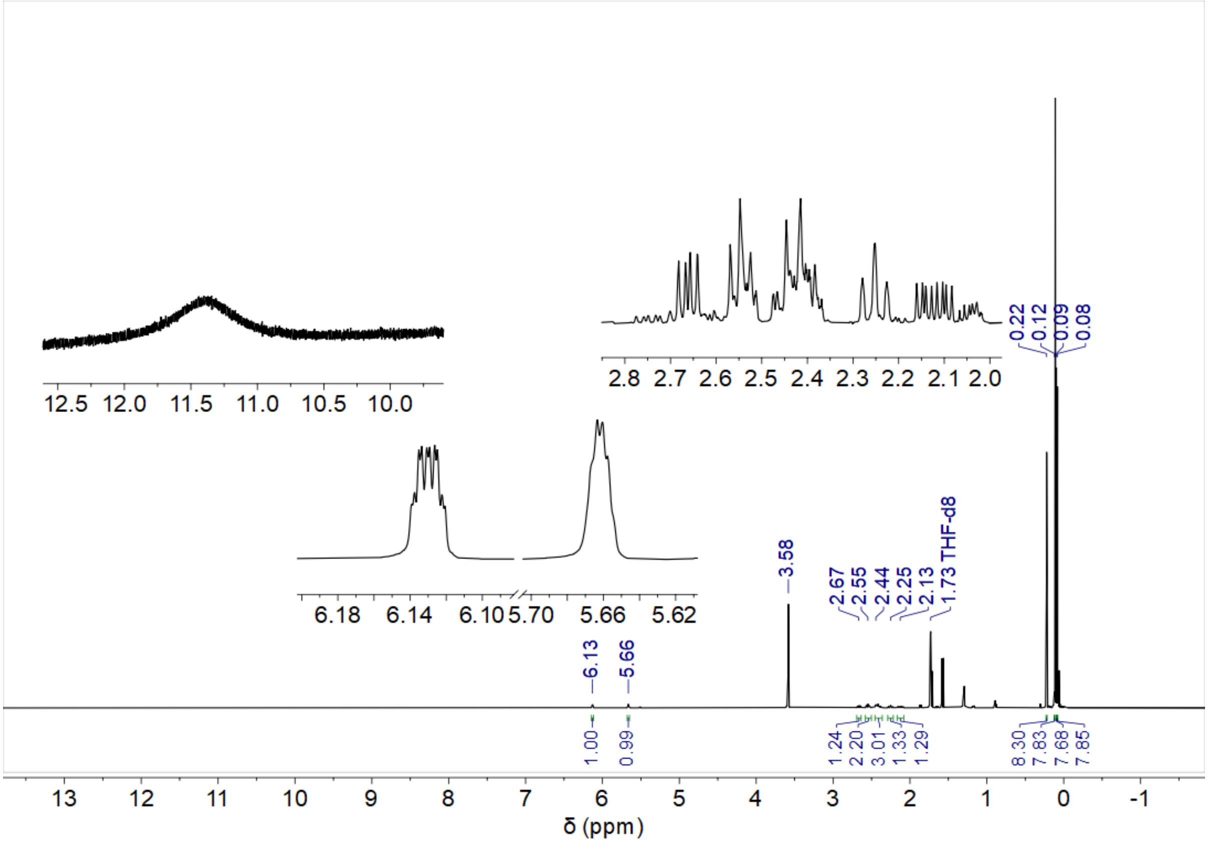

Figure S20: ^{1}H NMR spectrum of Hydrophosphination product **10**.

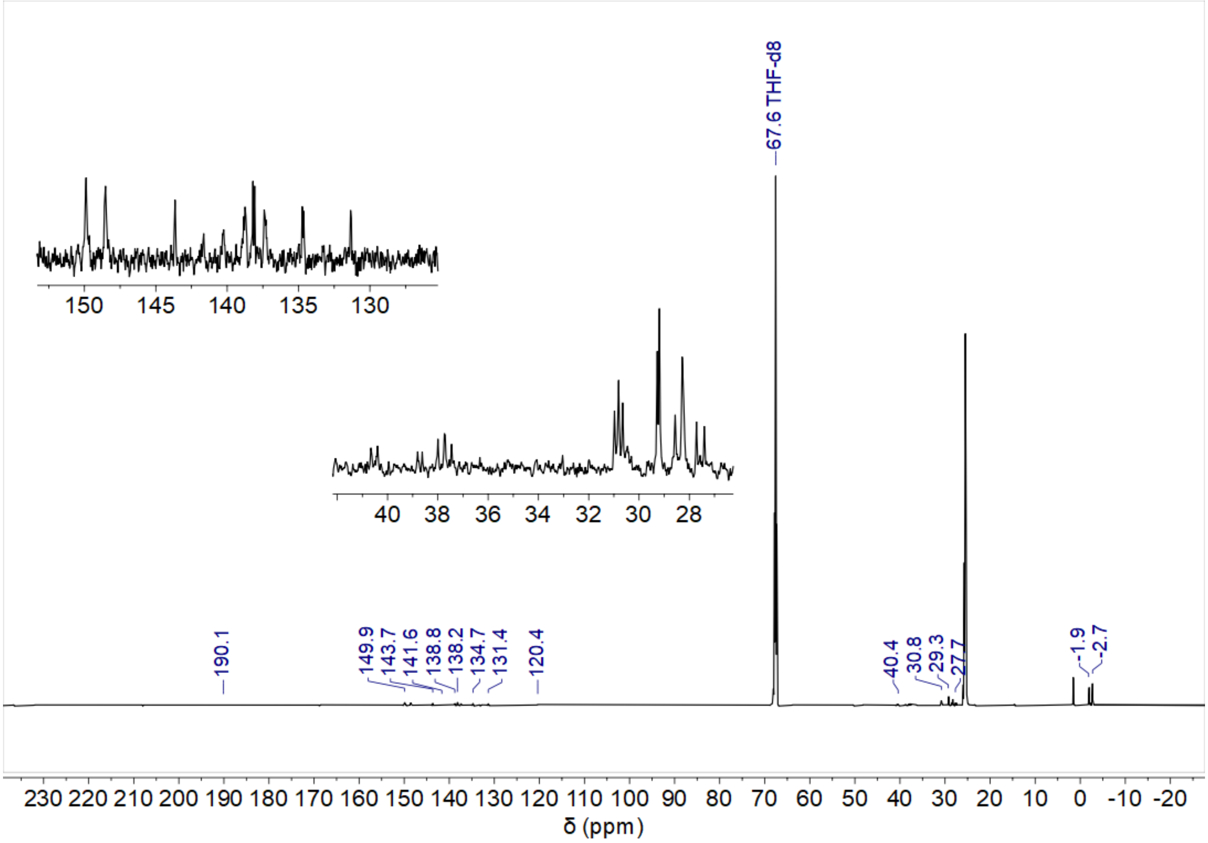

Figure S21: $^{13}C\{^{1}H\}$ NMR spectrum of Hydrophosphination product **10**.

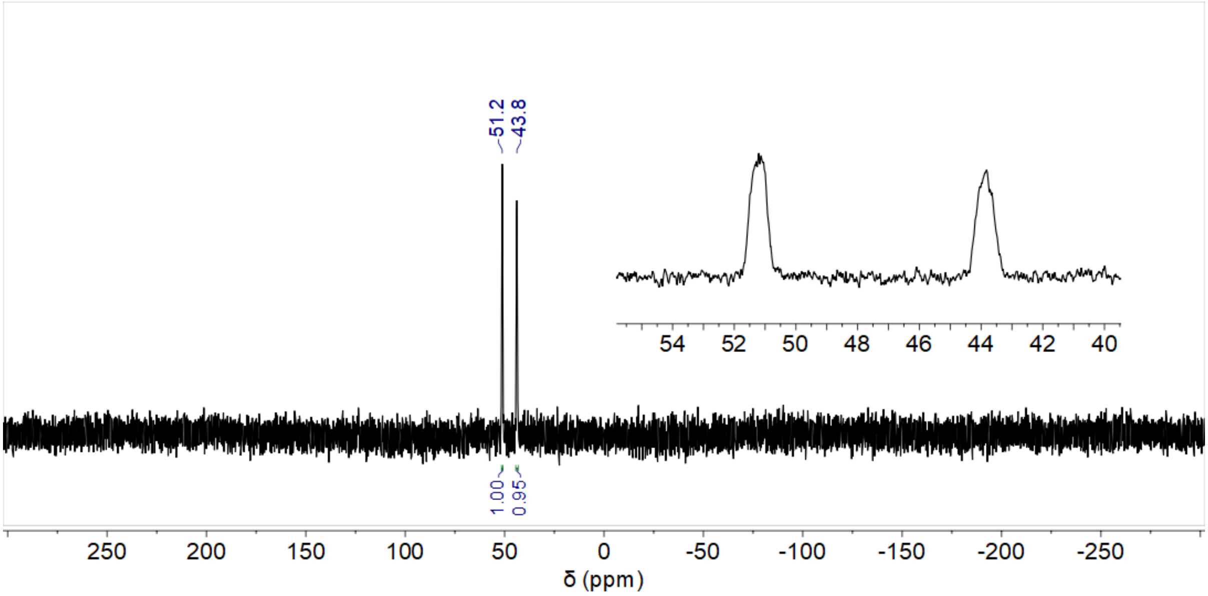

Figure S22: ^{31}P NMR spectrum of hydrophosphination product **10**.

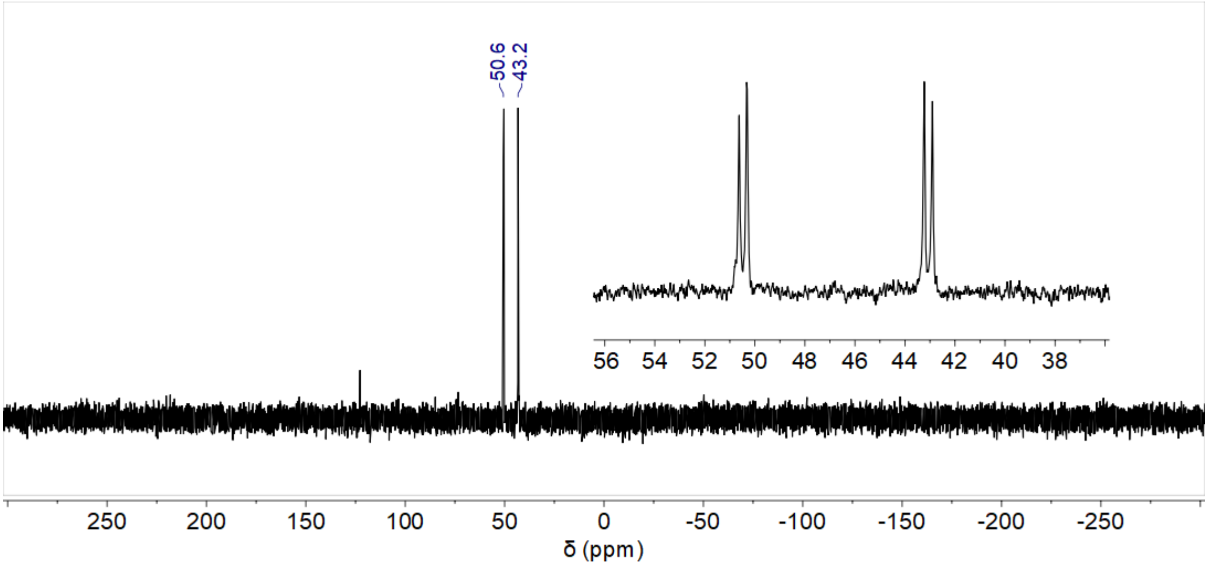

Figure S23: $^{31}P\{^1H\}$ NMR spectrum of hydrophosphination product **10**.

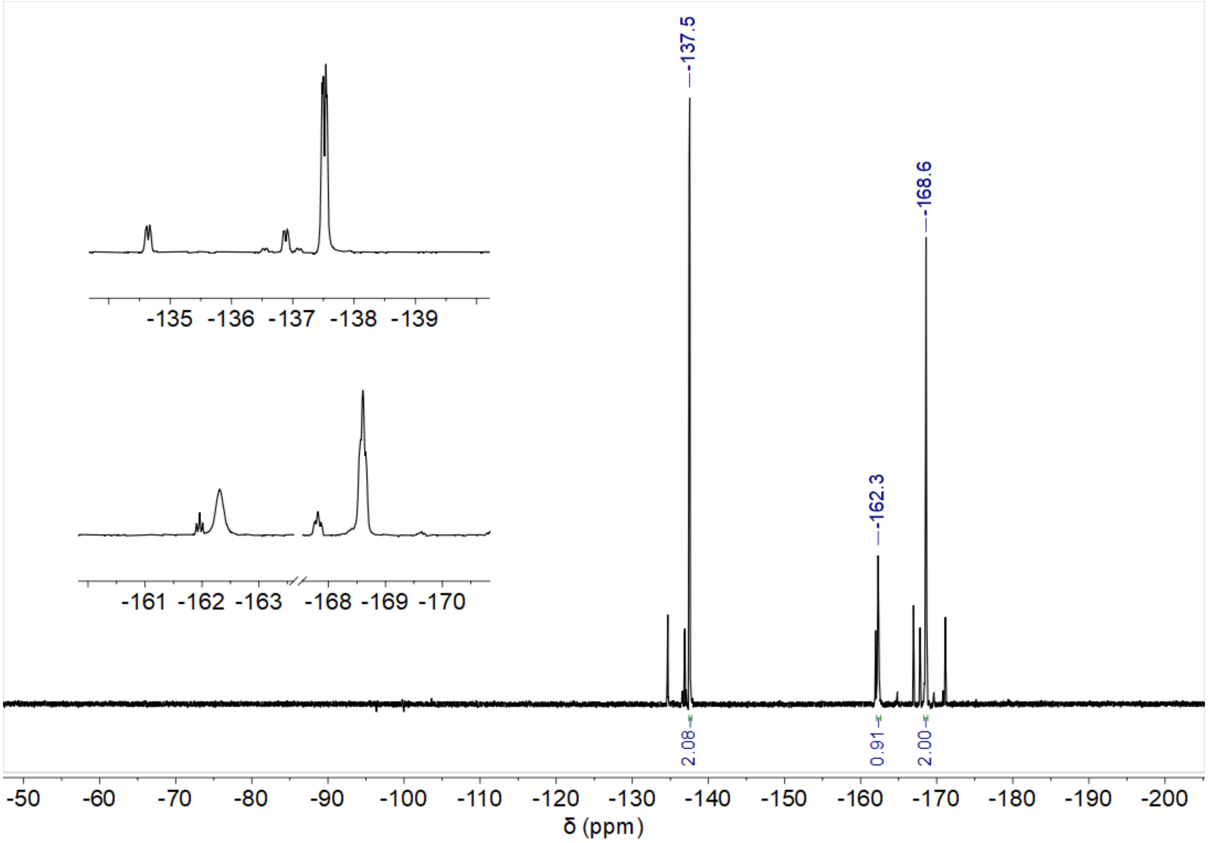

Figure S24: ^{19}F NMR spectrum of hydrophosphination product **10**.

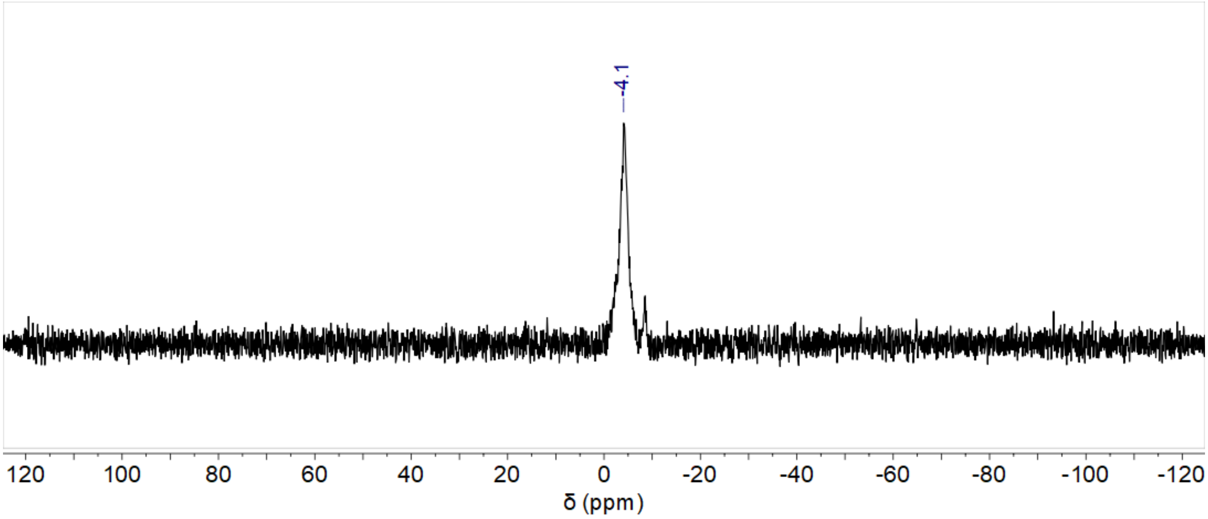

Figure S25: ^{11}B NMR spectrum of hydrophosphination product **10**.

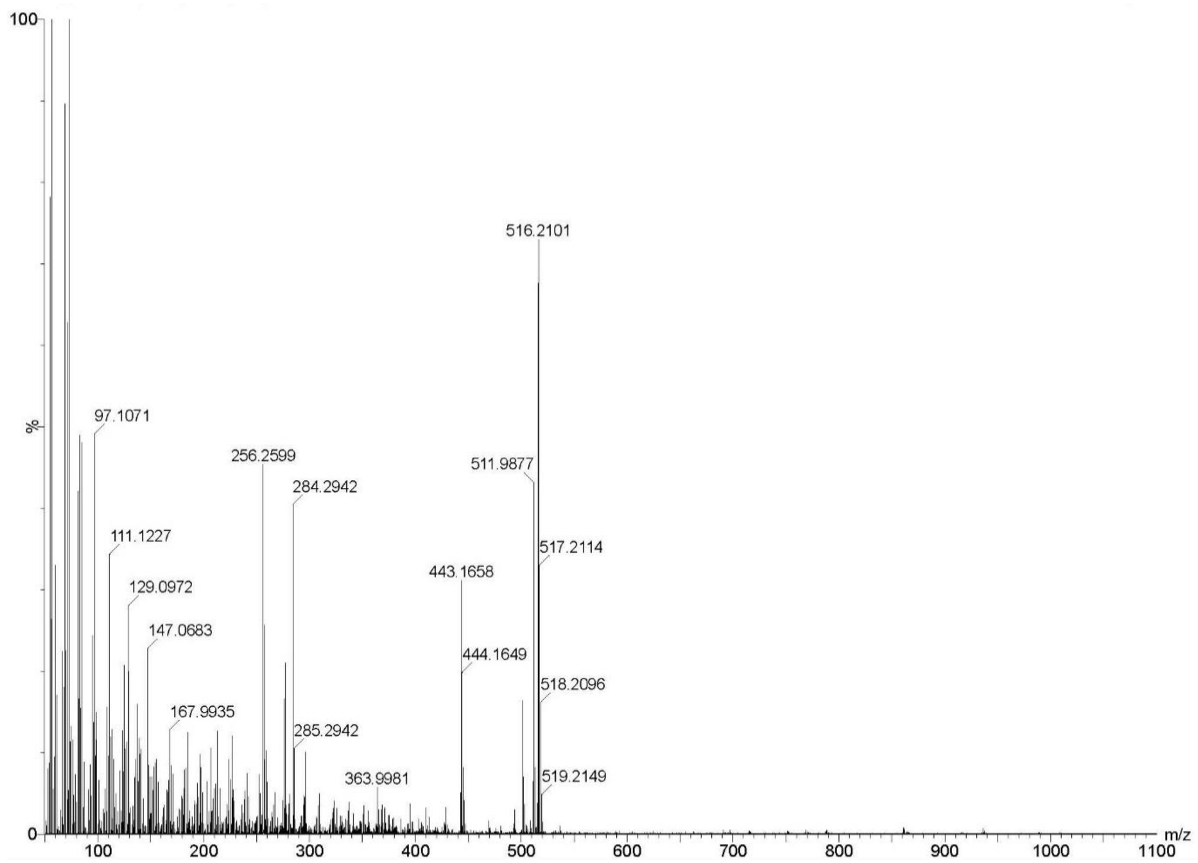

Figure S26: EI+ spectrum of hydrophosphination product **10**.

1.5.5 Reversibility with ${}^tBuOK$

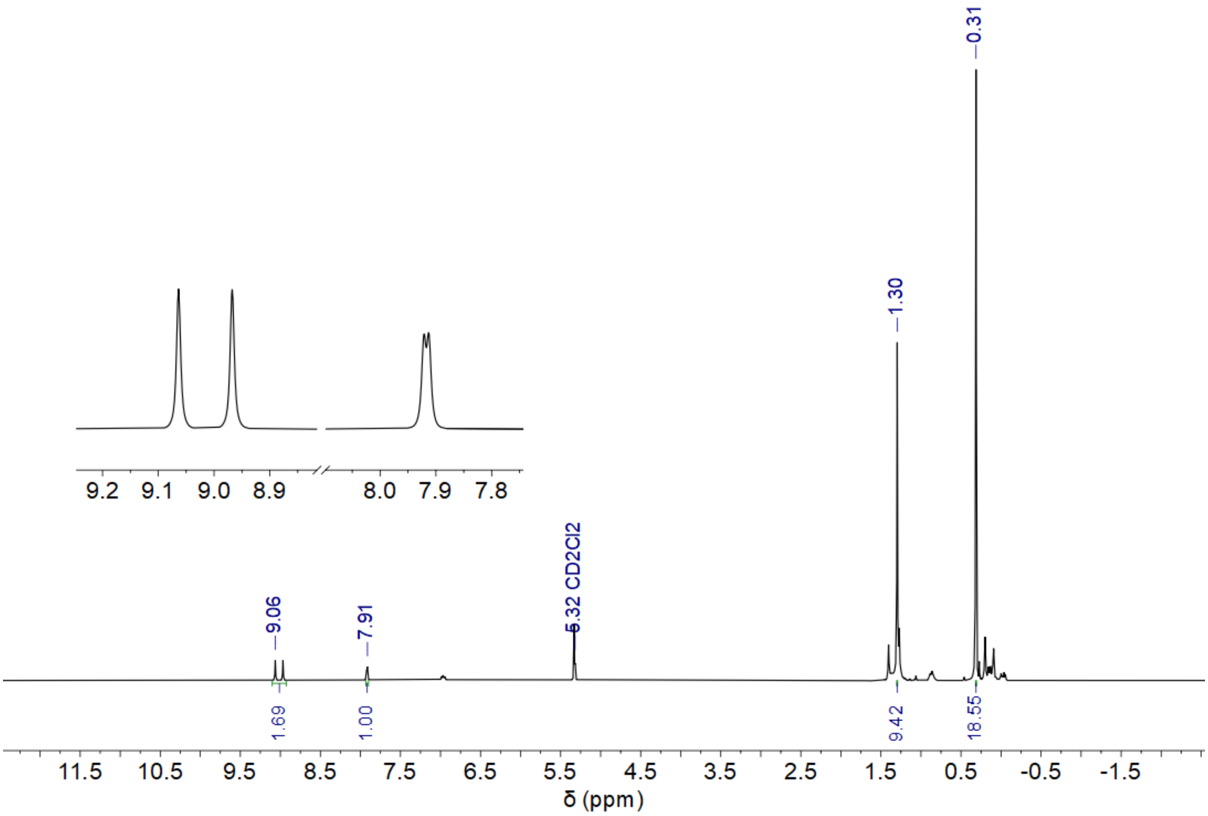

Figure S27: ${}^1H$ NMR spectrum of the reaction solution of dihydrophosphinine oxide **3/4** with KO^tBu.

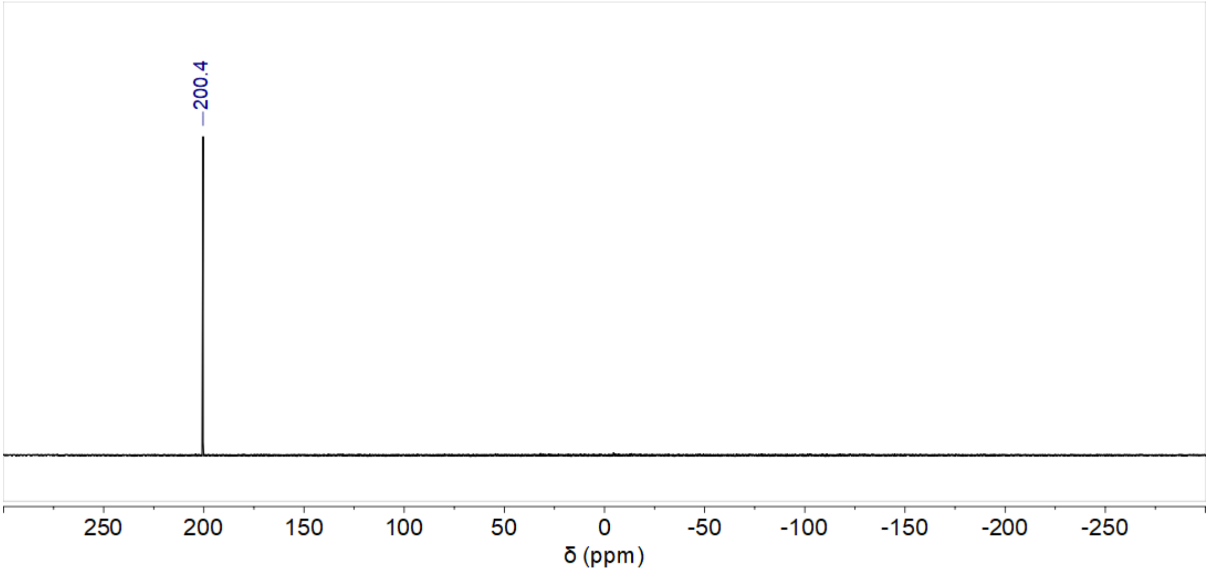

Figure S28: ${}^{31}P\{{}^1H\}$ NMR spectrum of the reaction solution of dihydrophosphinine oxide **3/4** with KO^tBu.

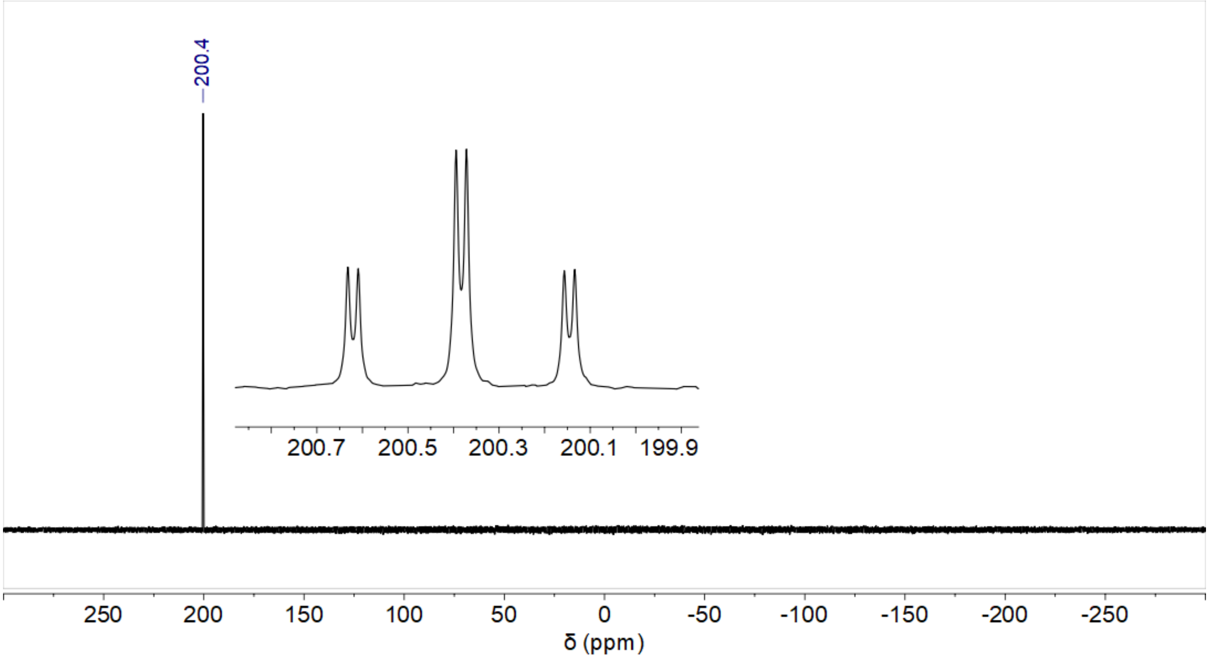

Figure S29: ^{31}P NMR spectrum of the reaction solution of dihydrophosphinine oxide **3/4** with KO^{t}Bu.

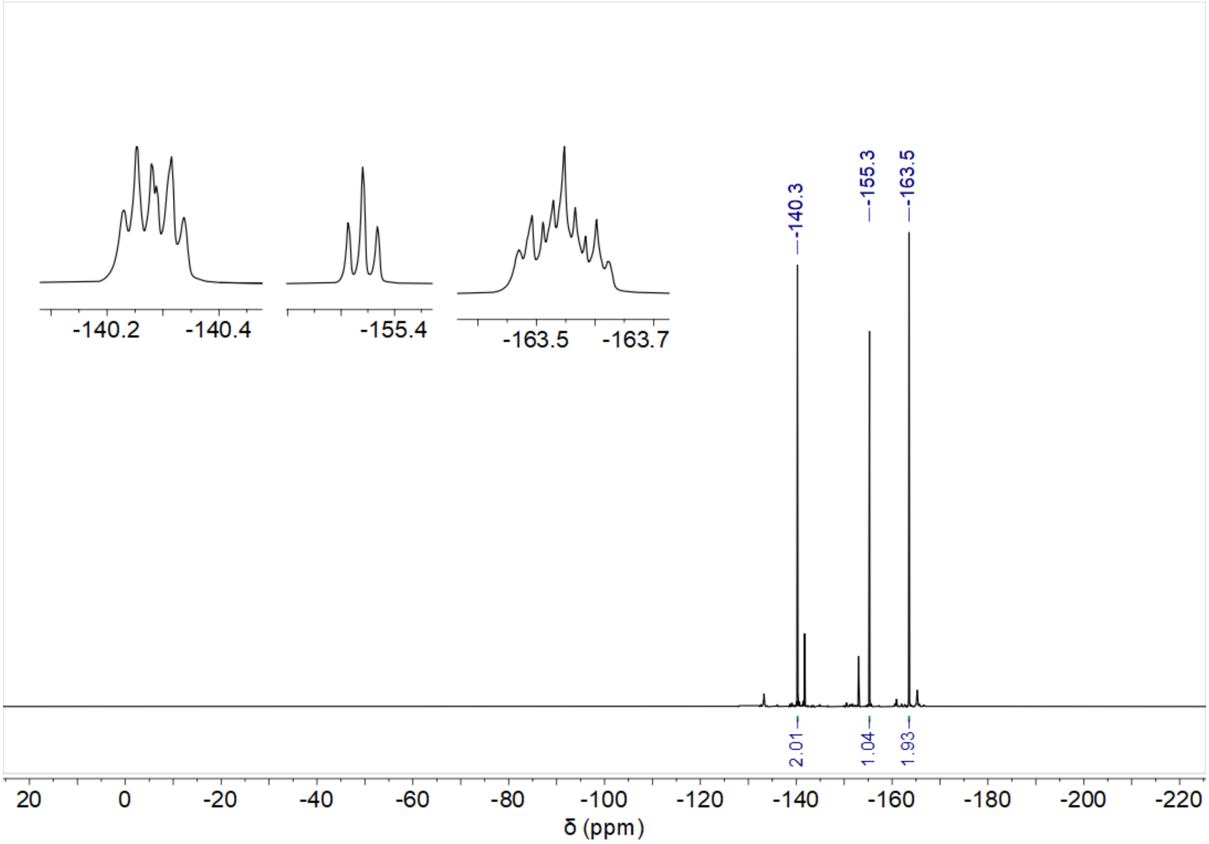

Figure S30: ^{19}F NMR spectrum of the reaction solution of dihydrophosphinine oxide **3/4** with KO^{t}Bu.

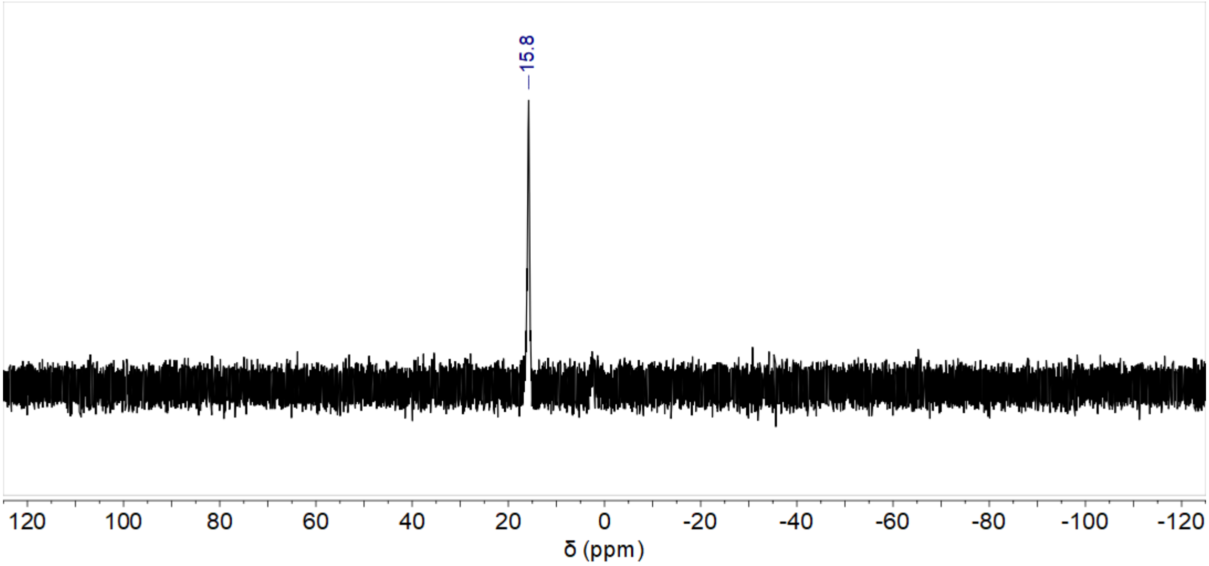

Figure S31: ^{11}B NMR spectrum of the reaction solution of dihydrophosphinine oxide **3/4** with KO^{t}Bu.

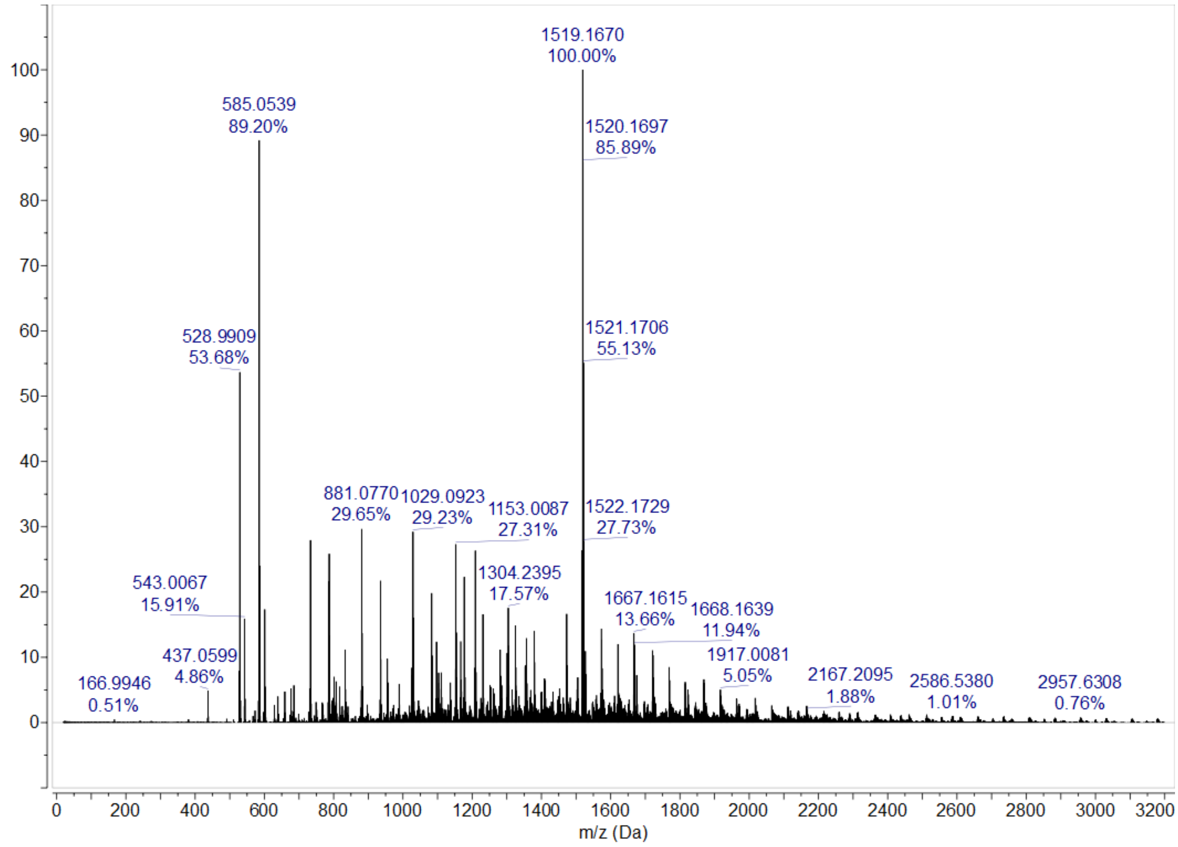

Figure S32: ESI– spectrum of the reaction solution of dihydrophosphinine oxide **3/4** with KO^{t}Bu.

2. Crystallographic Data

Single crystal x-ray diffraction data was collected on a Bruker D8 Venture fitted with a Photon II CMOS Detector with Mo *K*α radiation (λ = 0.71073 Å) from an *IμS* micro-source, performing φ-and ω-scans. Data collection and processing was handled using the *Bruker APEX3* and *Bruker APEX4* software packages.[50,51] Absorption corrections were carried out by the multiscan method.[52,53] Structures were solved by dual space iterative methods and refined in Olex2[54] with the SHELX program package.[55,56] and all non-hydrogen atoms refined by full-matrix least-squares on all unique F^2 values with anisotropic displacement parameters (SHELXL). All non-hydrogen atoms were refined anisotropically, all hydrogen atoms were included into the model at geometrically calculated positions and refined using a riding model. Structures were checked with checkCIF.[57] Selected crystallographic data can be found in Table S3 below. The CCDC entries 2492243 (**3**) and 2492244 (**10**) contain the supplementary crystallographic data for this article. These data can be obtained free of charge from The Cambridge Crystallographic Data Centre via www.ccdc.cam.ac.uk/structures.

Tabel S3 Crystal data and structure determination parameters.

	ortho-1,2-diydrophosphinine oxide	Hydrophosphination
Compound number	**3**	**10**
Empirical formula	$C_{29}H_{23}BF_{15}OPSi_2$	$C_{45}H_{60}BF_{15}O_3P_2Si_4$
Formula weight	770.43	1119.04
Temperature/K	100.00	100.00
Crystal system	monoclinic	monoclinic
Space group	$P2_1/n$	$P2_1/c$
a/Å	10.3863(4)	15.7103(5)
b/Å	26.7312(12)	28.6587(9)
c/Å	12.3645(6)	13.0839(4)
α/°	90	90
β/°	106.848(2)	113.0440(10)
γ/°	90	90
Volume/Å^3	3285.5(3)	5420.8(3)
Z	4	4
ρ_{calc}/g cm^{-3}	1.558	1.371
μ/mm^{-1}	0.267	0.258
F(000)	1552.0	2320.0
Crystal size/mm^3	0.198 × 0.121 × 0.111	0.757 × 0.27 × 0.112
Radiation	Mo K_α (λ = 0.71073)	Mo K_α (λ = 0.71073)
Θ range for data collection/°	4.372 to 51.42	4.418 to 56.606
Index ranges	$-12 \le h \le 12, -32 \le k \le 32, -15 \le l \le 15$	$-20 \le h \le 20, -35 \le k \le 38, -17 \le l \le 16$
Reflections collected	103197	85785
Independent reflections	6257 [R_{int} = 0.0442, R_{sigma} = 0.0155]	13228 [R_{int} = 0.0446, R_{sigma} = 0.0340]
Data/restraints/parameters	6257/0/453	13228/0/606
Goodness-of-fit on F^2	1.143	1.043
Final R indexes [I>=2σ (I)]	R_1 = 0.0383, wR_2 = 0.0827	R_1 = 0.0586, wR_2 = 0.1300
Final R indexes [all data]	R_1 = 0.0499, wR_2 = 0.0948	R_1 = 0.1034, wR_2 = 0.1757
Largest diff. peak/hole / e Å^{-3}	0.38/−0.42	0.63/−0.65
Diffractometer	Bruker D8 Venture	Bruker D8 Venture
CCDC access code	2492243	2492244

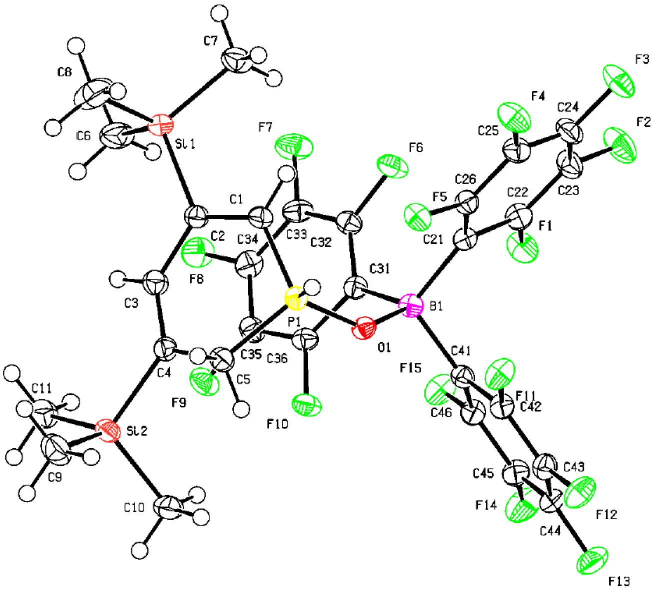

Figure S33: Ellipsoid representation of **3**. The thermal ellipsoids are set at a 50% probability level.

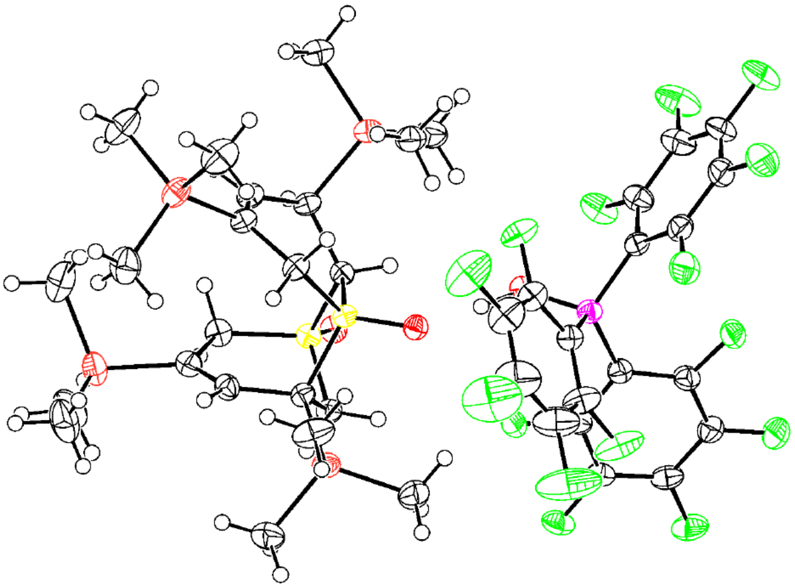

Figure S34: Ellipsoid representation of **10**. The thermal ellipsoids are set at a 50% probability level. A solvent mask was calculated, and 172 electrons were found in a volume of 892 Å^3 in 1 void per unit cell. This is consistent with the presence of one *n*-pentane molecule (C_5H_{12}) per formula unit which account for 168 electrons per unit cell.

3. DFT calculations

3.1 Computed NMR chemical shifts

DFT calculations on the ^{31}P NMR chemical shifts of the *ortho*-1,2-diydrophosphinine oxide **3** and *para*-1,2-diydrophosphinine oxide **4** with 2,3,5,6-tetra-pyridyl 1,2-dihydrophosphinine oxide as reference[25] were performed with Gaussian 16 (Revision C.02)(GAUSSIAN)[58] at the B3LYP-D3(BJ)/def2-TZVP[59–62] level of theory with the option of tight SCF convergence in the gas phase. Frequency calculations were performed at the same level and confirmed local minima without any imaginary frequency. Calculation were performed with the HPC cluster *Curta*.[63]

To determine the chemical shifts (δ) from the calculated chemical shielding (σ) the following equation was used:

$$\delta[^{n}M] = \sigma(\text{reference}) - \sigma(\text{compound}) + \delta(\text{reference})$$

While σ(reference) is the absolute shielding constant of the chosen reference 2,3,5,6-tetra-pyridyl 1,2-dihydrophosphinine oxide, calculated on the same level of theory. σ(compound) is the absolute shielding constant of the desired compound and δ(reference) is the experimental chemical shift of the reference compound, relative to the primary reference.[64-66]

2,3,5,6-tetra-pyridyl-1,2-dihydrophosphinine oxide was chosen as reference due to the similar chemical and electronic environment of the phosphorus atom in the here reported diydrophosphinine oxide **3** and **4**.

Table S4: Calculated structure of 2,3,5,6-tetra-pyridyl-1,2-dihydro-phosphinine oxide, *ortho* and *para* dihydrophosphinine oxide **3** and **4** and the corresponding experimentally chemical shifts as well the calculated chemical shielding *σ.*

**structure**	**$\sigma(^{31}P)$ cal. [ppm]**	**$\delta(^{31}P)$ cal. [ppm]**	**$\delta(^{31}P)$ exp. [ppm]**
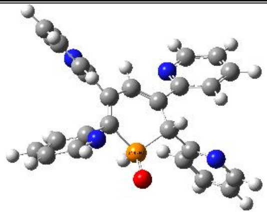	264.16	(reference)	21.1[22]

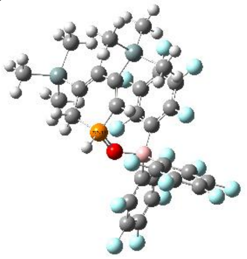 3	276.17	9.0	7.7
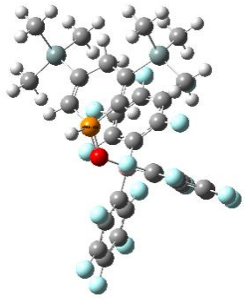 4	293.25	–8.1	–12.9

3.2 Formal insertion of the P atom into the H-O bond of H_2O

Without borane:

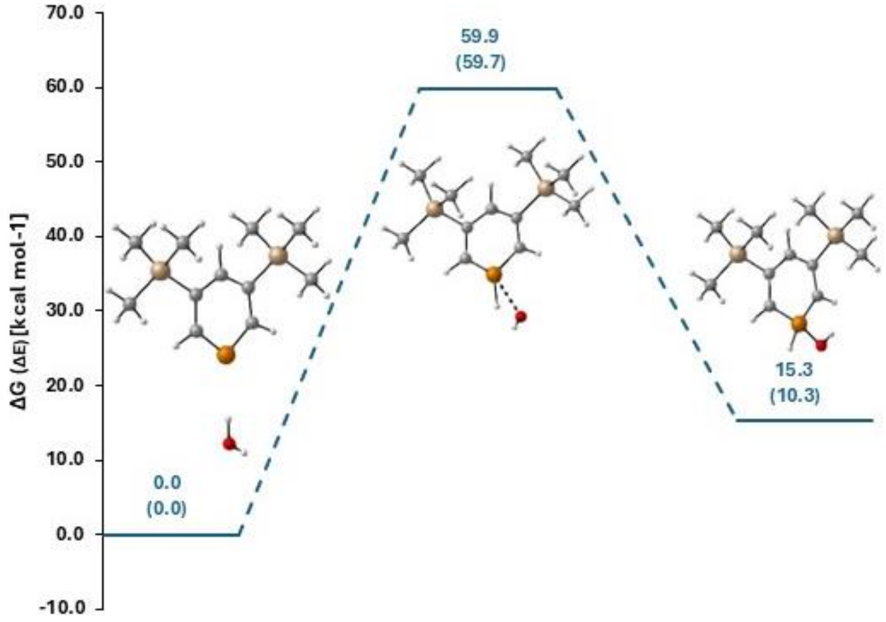

Figure S35 Direct reaction of **1** and H_2O.

Since the activation barrier in Figure S35 are enormously high, the subsequent tautomerization step was not studied in these cases.

With borane:

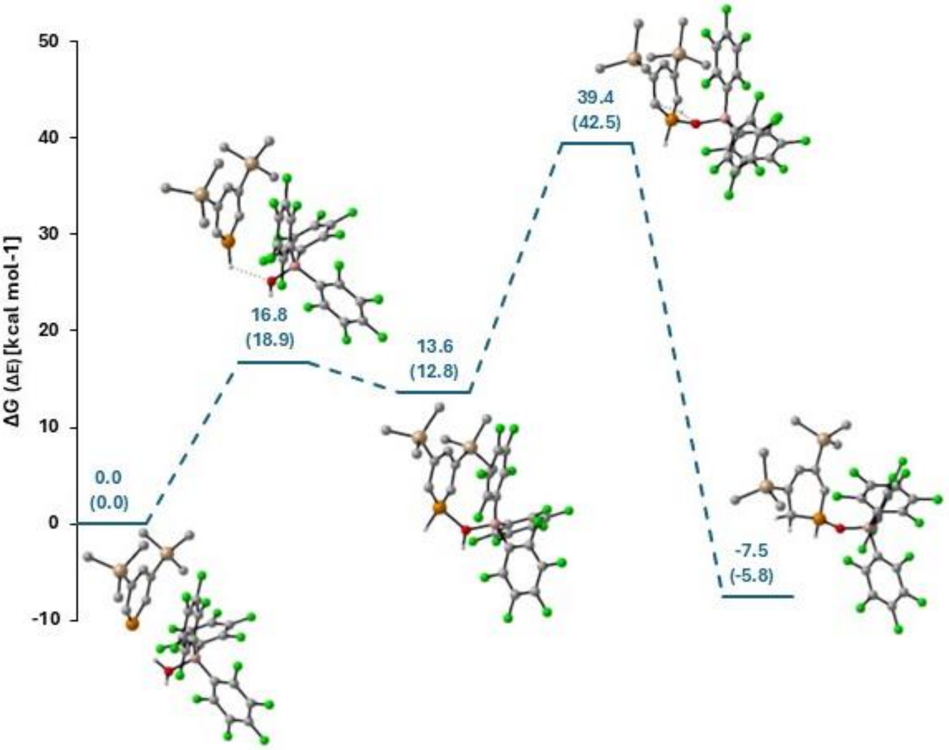

Figure S36 Reaction of **1** with [$H_2O \rightarrow B(C_6F_5)_3$] to **3** (*ortho*).

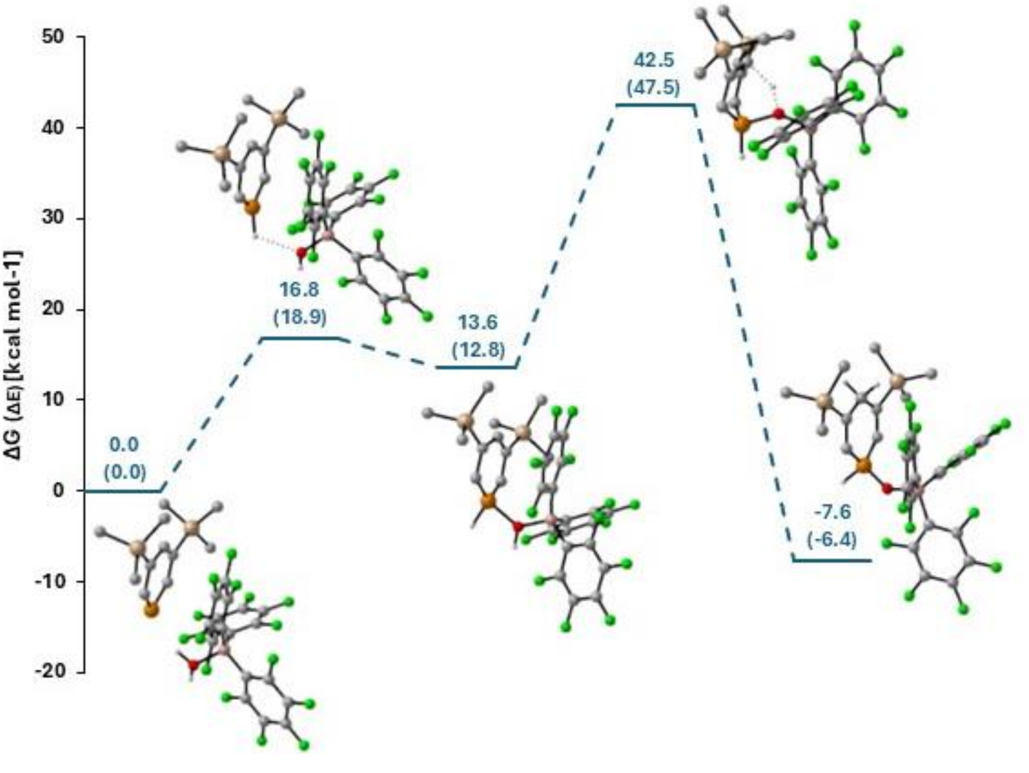

Figure S37 Reaction of **1** with [$H_2O \rightarrow B(C_6F_5)_3$] to **4** (*para*).

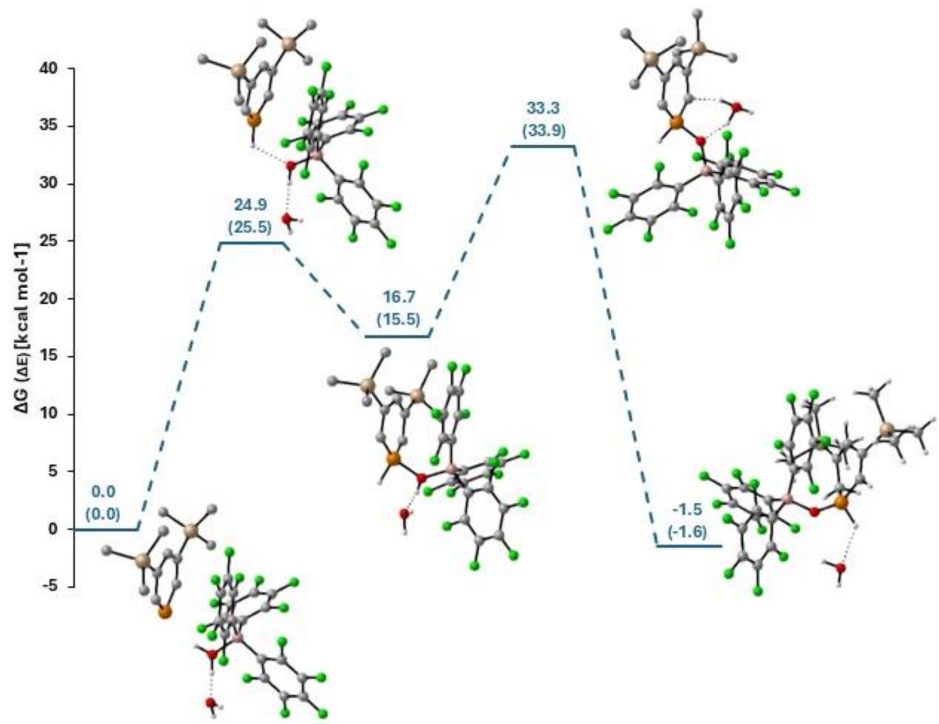

Figure S38 Reaction of **1** with $[H_2O{\rightarrow}B(C_6F_5)_3]{\cdot}(H_2O)$ to **3** (*ortho*)

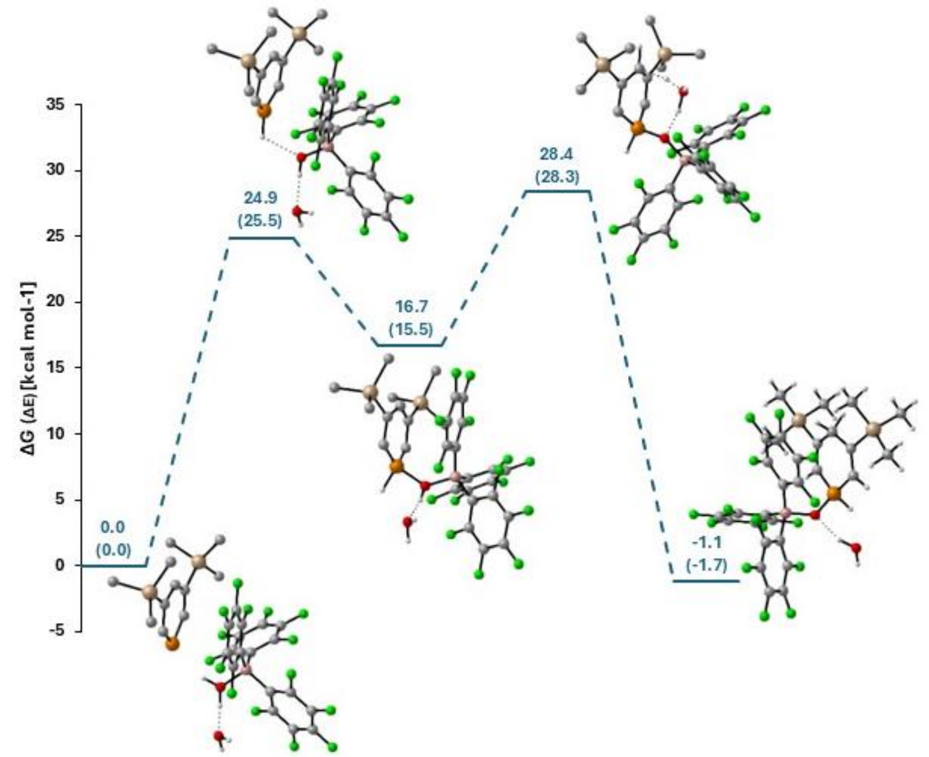

Figure S39 Reaction of **1** with $[H_2O{\rightarrow}B(C_6F_5)_3]{\cdot}(H_2O)$ to **4** (*para*)

3.3 1,2- and 1,4-additions

Without borane:

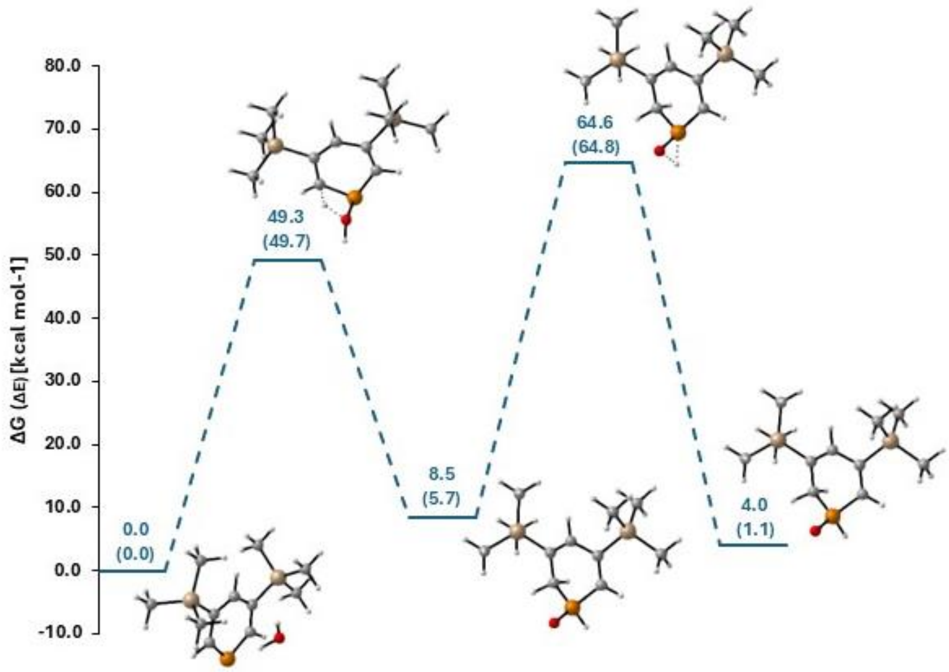

Figure S40 1,2-addition of H_2O to **1**.

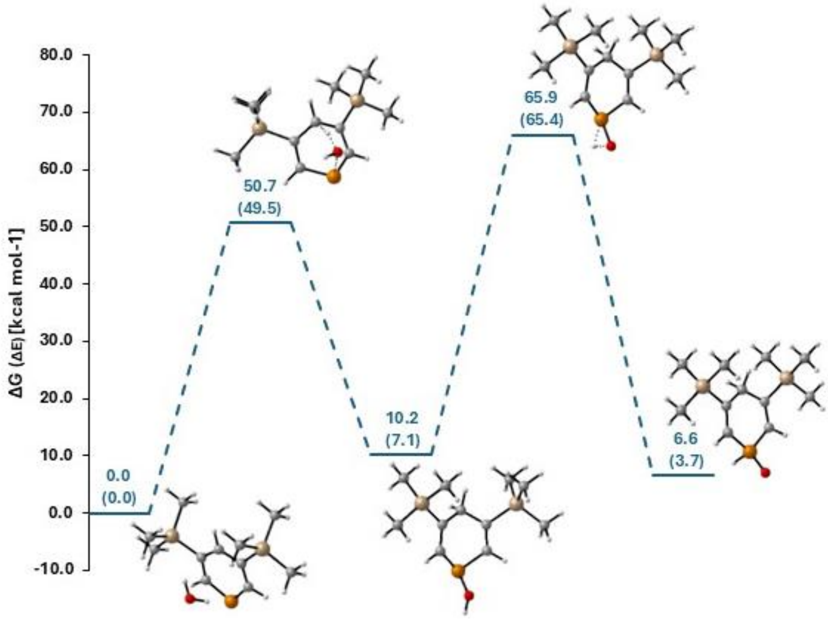

Figure S41 1,4-addition of H_2O to **1**.

With borane

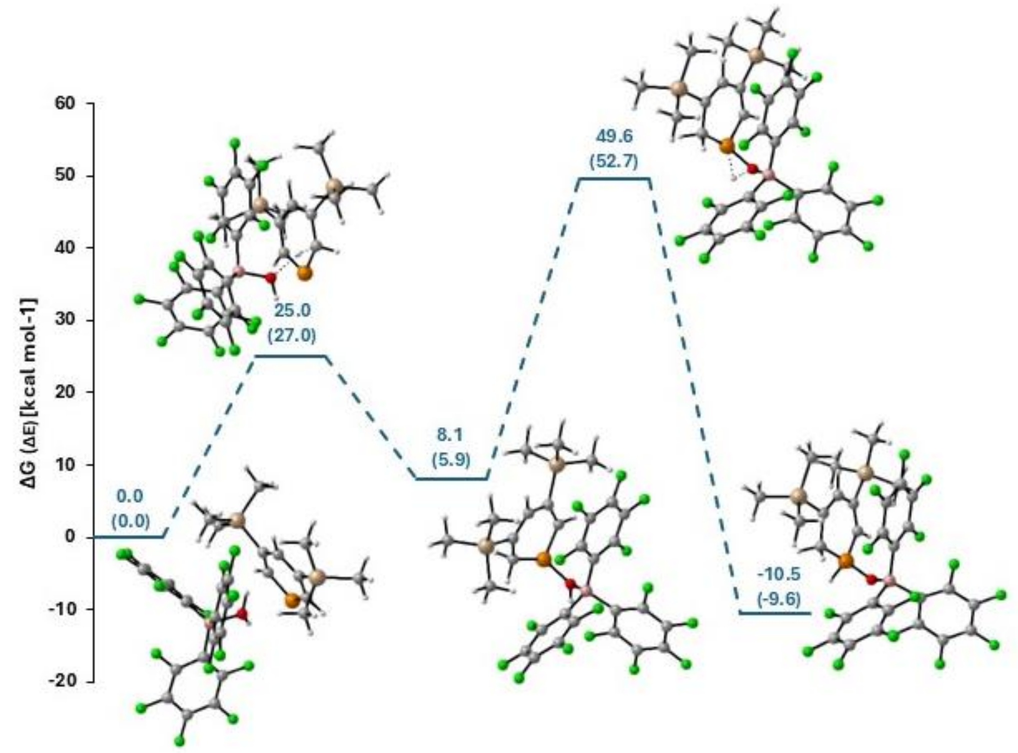

Figure S42 1,2-addition of [$H_2O \rightarrow B(C_6F_5)_3$] to **1**.

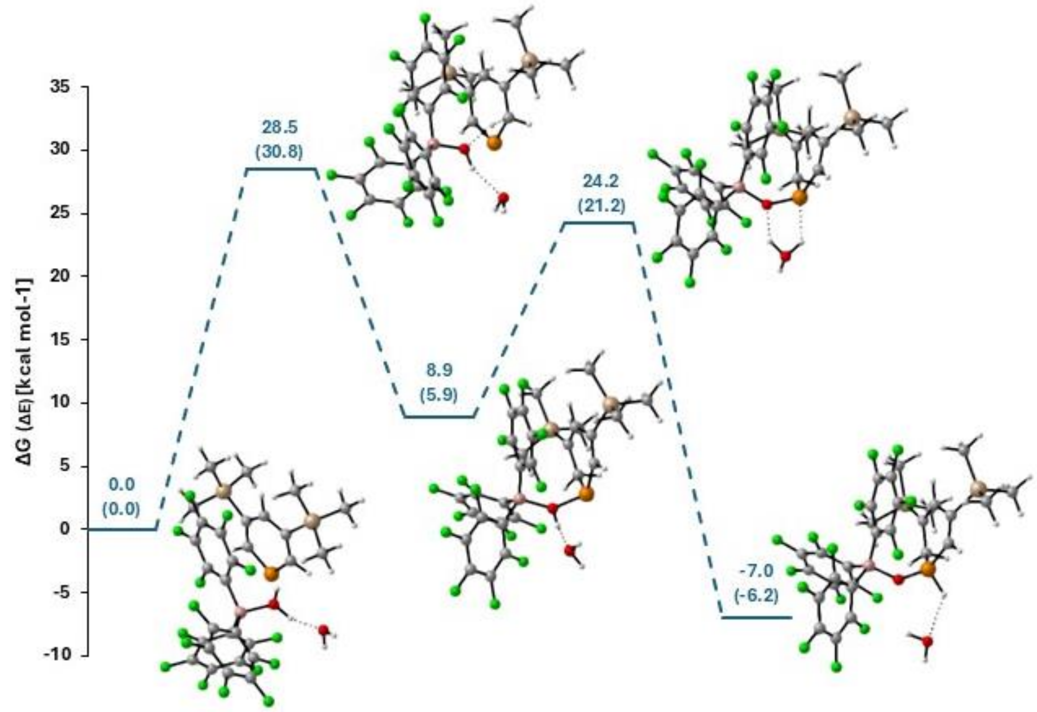

Figure S43 1,2-addition of [$H_2O \rightarrow B(C_6F_5)_3$]·($H_2O$) to **1**.

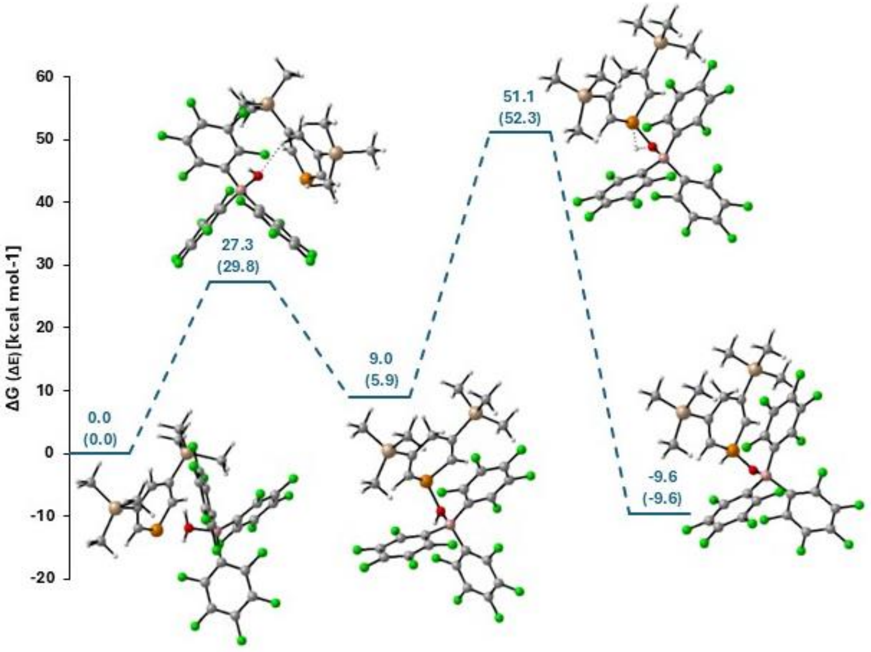

Figure S44 1,4-addition of $[H_2O \rightarrow B(C_6F_5)_3]$ to **1**.

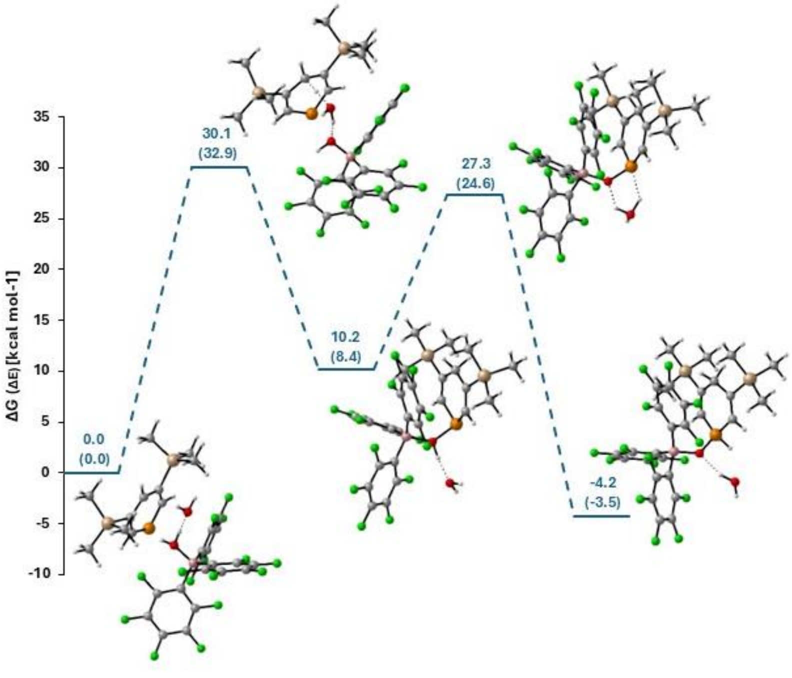

Figure S45 1,4-addition of $[H_2O \rightarrow B(C_6F_5)_3] \cdot (H_2O)$ to **1**.

Additions onto Lewis pair 2

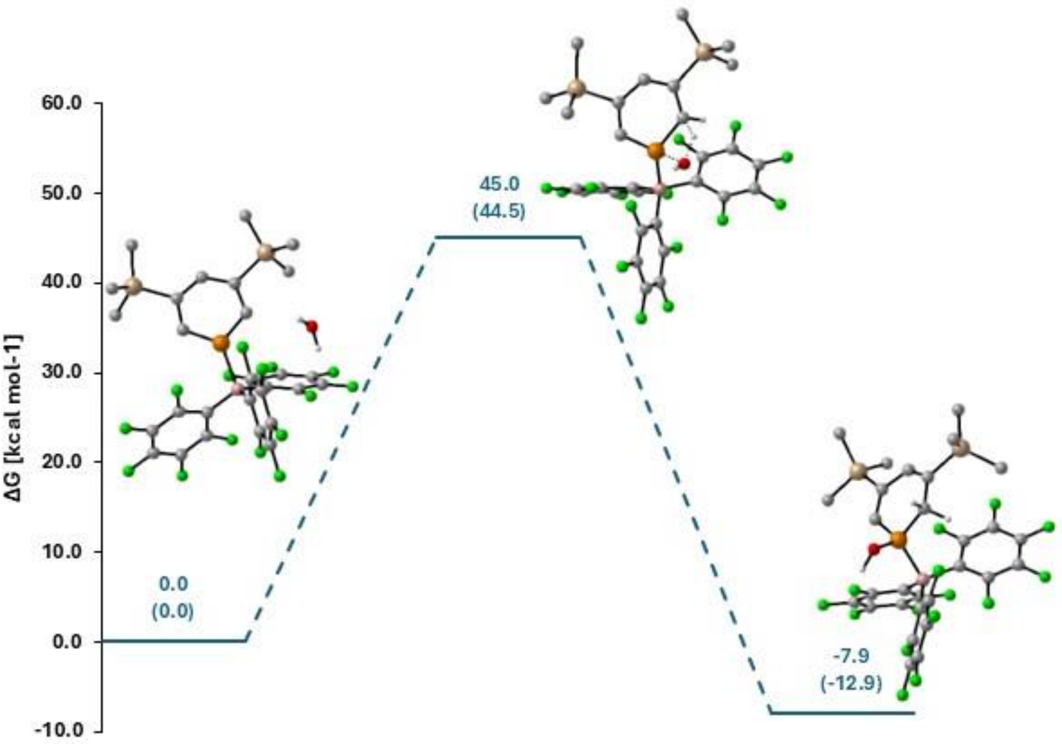

Figure S46 1,2-addition of (H_2O) to **2**.

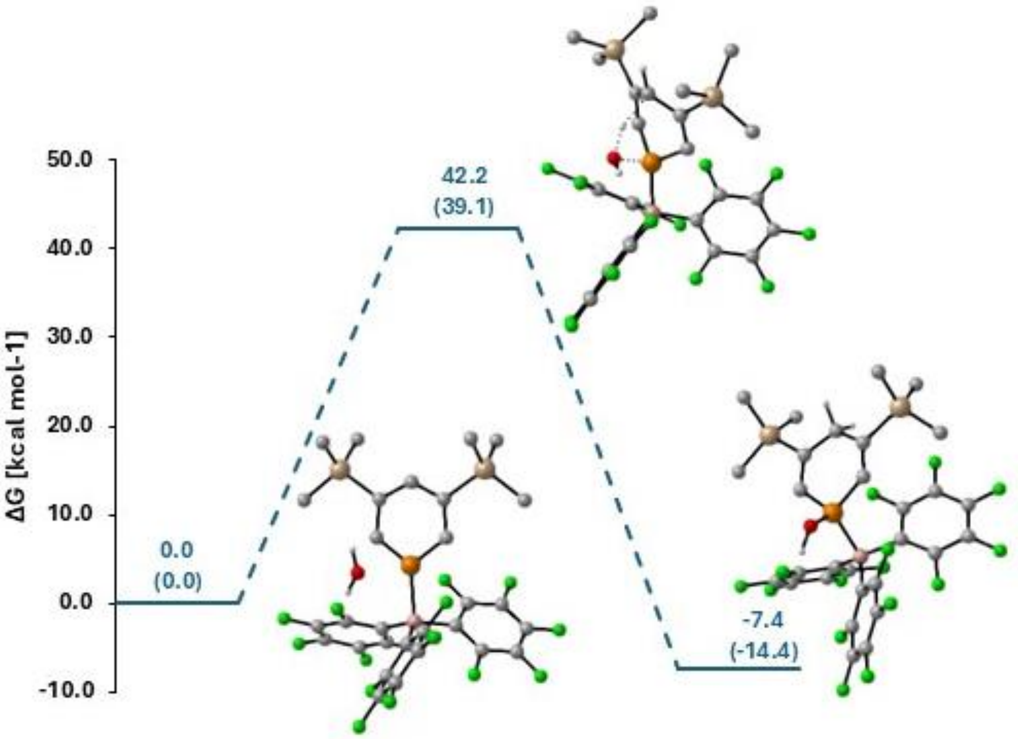

Figure S46 1,4-addition of (H_2O) to **2**.

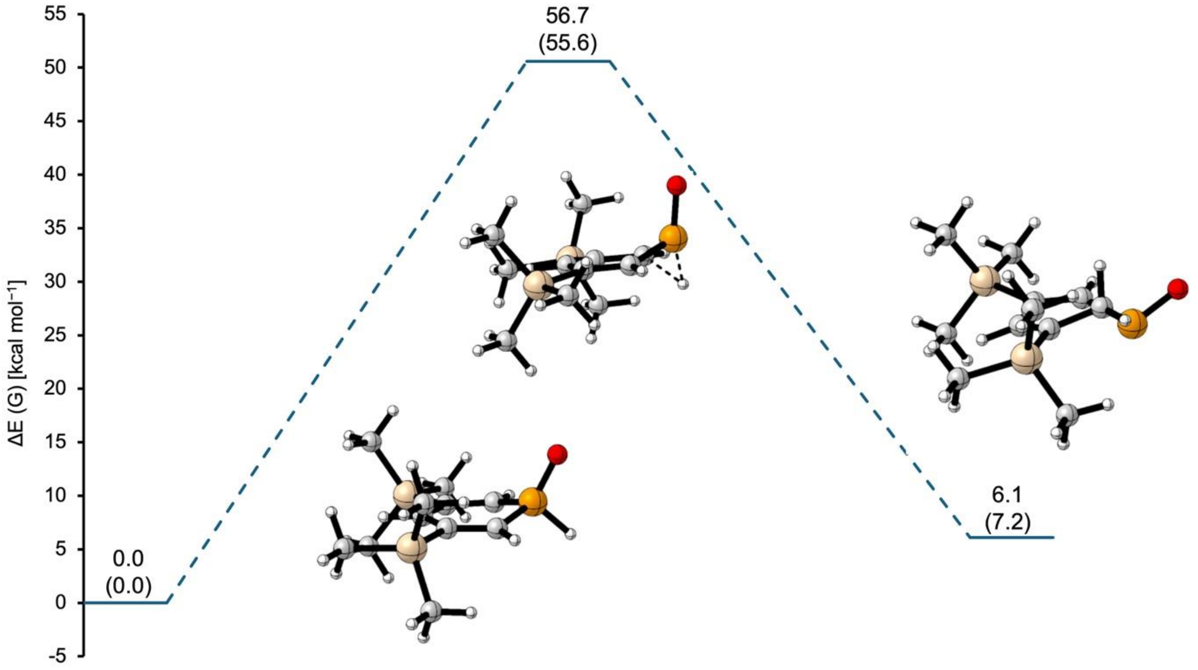

Figure S47 Isomerization of **7** to **11**.

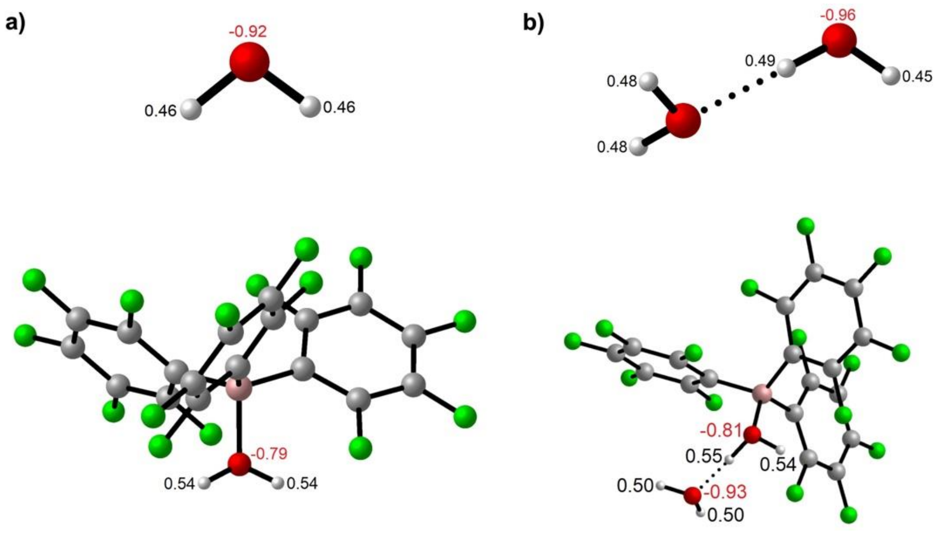

Figure S48 The calculated NPA atomic charges for a) water monomer and $[H_2O\rightarrow B(C_6F_5)_3]$ adduct and b) water dimer and $[H_2O\rightarrow B(C_6F_5)_3]\cdot(H_2O)$ adduct.

3.4 Investigation on the hydrophosphination reaction

Reaction profiles

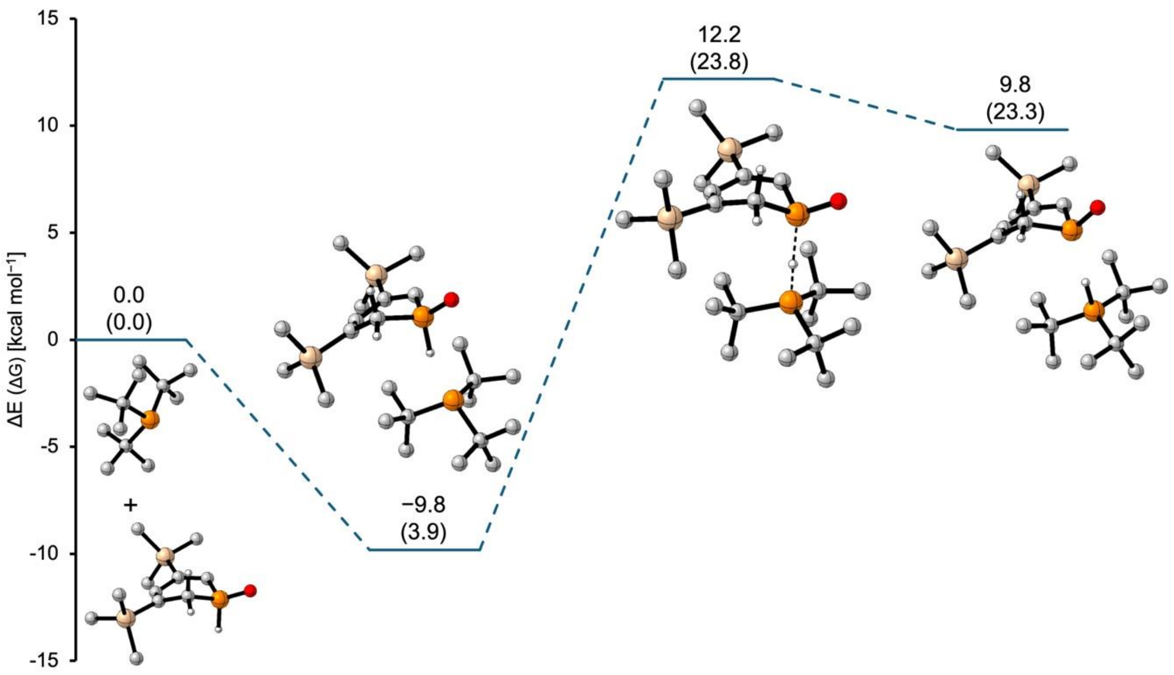

Figure S49 P(O)H-deprotonation of **5** by P^{t}Bu$_3$.

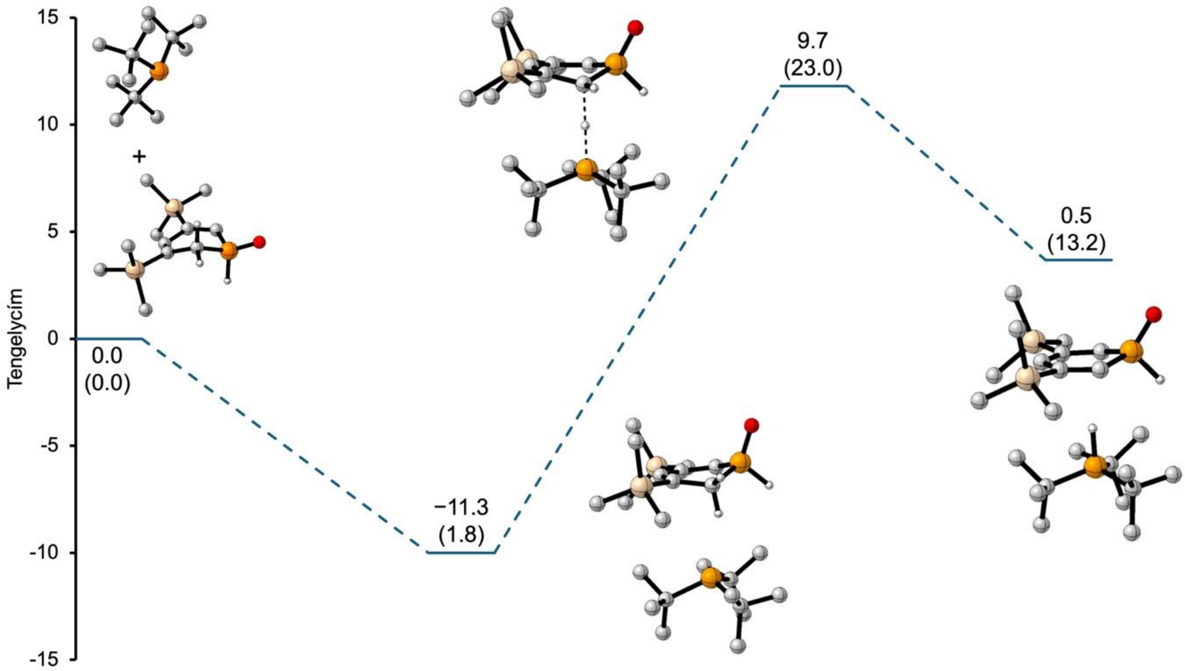

Figure S50 Ortho C(sp^3)-H deprotonation of **5** by P^{t}Bu$_3$.

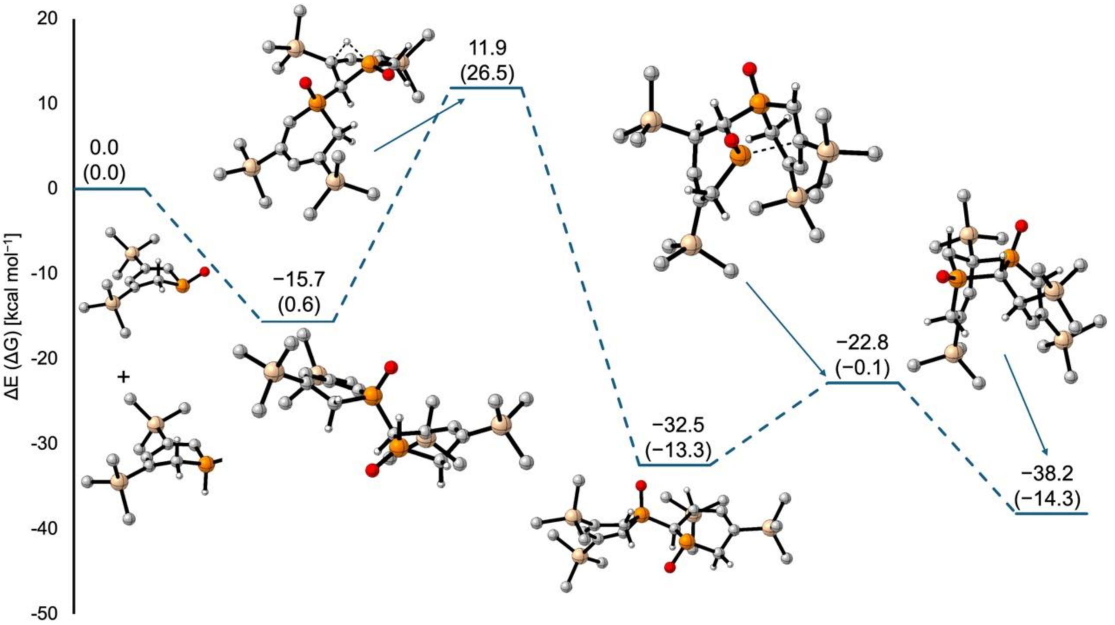

Figure S51 Reaction of **5** and the **11** anion.

3.5 Optimized geometries (in xyz format) and electronic energies (in a. u.)

3.5.1 General

3,5-bis(SiMe$_3$) phosphinine **1**:
$E_{\omega B97X\text{-}D/6\text{-}311+G(d,p)}$= -1352.22351527

C -3.834788 -0.182955 1.536384
Si -2.856266 -0.636870 0.000001
C -3.835022 -0.182652 -1.536143
C -2.459672 -2.470497 -0.000213
C -1.246052 0.368330 -0.000015
C -0.000000 -0.272043 -0.000004
C 1.246051 0.368330 0.000007
Si 2.856265 -0.636870 0.000001
C 2.459672 -2.470497 0.000049
C -1.330963 1.762934 -0.000019
P -0.000000 2.869453 -0.000004
C 1.330963 1.762934 0.000012
C 3.834875 -0.182843 -1.536292
C 3.834938 -0.182765 1.536231
H 2.319299 2.221029 0.000012
H -0.000000 -1.358646 -0.000006
H -2.319299 2.221030 -0.000021
H -4.787435 -0.721037 1.561590
H -3.277847 -0.434717 2.443584
H -4.053828 0.888691 1.562045
H -4.787692 -0.720697 -1.561281
H -4.054032 0.889005 -1.561590
H -3.278240 -0.434280 -2.443478
H -3.387397 -3.050852 -0.000274
H -1.886320 -2.758577 -0.886371
H -1.886306 -2.758787 0.885867
H 3.387396 -3.050852 0.000083
H 1.886295 -2.758658 0.886165
H 1.886329 -2.758707 -0.886073
H 4.787619 -0.720790 1.561371
H 4.053922 0.888896 1.561775

H 3.278120 -0.434479 2.443520
H 4.787514 -0.720939 -1.561490
H 3.277979 -0.434522 -2.443543
H 4.053935 0.888802 -1.561857

tris(pentafluorophenyl) borane:
$E_{\omega B97X\text{-}D/6\text{-}311+G(d,p)}$= -2208.2750957

C -1.743649 1.751783 0.771720
C -0.626816 1.434601 0.000688
C -0.102566 2.470545 -0.770350
C -0.651734 3.739628 -0.790946
C -1.756457 4.012734 0.000135
C -2.304491 3.015755 0.791685
B 0.000969 -0.000002 0.000673
C 1.556928 -0.174354 0.000534
C 2.390566 0.631858 0.773493
C 3.765512 0.484317 0.793426
C 4.354255 -0.487241 -0.000479
C 3.564848 -1.304649 -0.793896
C 2.191293 -1.144931 -0.772790
F 1.869851 1.575257 1.561303
F 4.523484 1.260260 1.560300
F 5.668796 -0.634049 -0.001218
F 4.132126 -2.228454 -1.561658
F 1.474607 -1.949849 -1.560340
F 0.954310 2.253679 -1.556341
F -0.134237 4.694373 -1.556050
F -2.287679 5.224111 -0.000567
F -3.357368 3.282638 1.556483
F -2.301600 0.827886 1.557192
C -0.928072 -1.260567 -0.000193
C -2.085257 -1.324597 -0.774100
C -2.911217 -2.433623 -0.794660
C -2.598946 -3.525701 -0.000455
C -1.463551 -3.501585 0.794027
C -0.647599 -2.385103 0.773627
F -2.422968 -0.301416 -1.562079
F 0.429312 -2.405501 1.562109
F -1.171815 -4.545575 1.561919
F -3.384275 -4.590093 -0.000250
F -3.995113 -2.462514 -1.562155

Lewis pair **2**:
$E_{\omega B97X\text{-}D/6\text{-}311+G(d,p)}$= -3560.52547579

C -1.615526 -0.048121 2.594790
C -1.979406 -0.296526 1.280627
C -3.319468 -0.626747 1.102155
C -4.236654 -0.707623 2.133090
C -3.823102 -0.447923 3.428988
C -2.501899 -0.116054 3.659827
B -1.046140 -0.184809 -0.052622
C -0.703354 -1.609334 -0.781152
C -0.966781 -2.862773 -0.239874
C -0.563593 -4.051255 -0.835658
C 0.148049 -4.020766 -2.018931
C 0.447592 -2.796244 -2.593260
C 0.024607 -1.638427 -1.970902
F -1.620230 -2.998150 0.921234
F -0.850417 -5.220391 -0.266175
F 0.545945 -5.148144 -2.595546
F 1.146394 -2.747006 -3.725123
F 0.387478 -0.484768 -2.552093
F -3.770384 -0.914264 -0.128098
F -5.504840 -1.038114 1.894876

F -4.686045 -0.519039 4.437073
F -2.088406 0.136689 4.901092
F -0.350238 0.278532 2.911124
P 0.874963 0.363551 0.380625
C 2.069795 -0.835736 0.533399
C 3.427969 -0.528693 0.599677
C 3.874632 0.799032 0.541856
C 3.074208 1.941584 0.414619
Si 3.849061 3.683196 0.343954
C 3.294526 4.481122 -1.258996
C 1.681369 1.859005 0.321022
Si 4.644030 -1.991935 0.715345
C 4.140587 -3.220834 -0.610022
C 6.386368 -1.368237 0.431675
C 4.463439 -2.754720 2.417456
C 3.197990 4.646314 1.814045
C 5.714370 3.533830 0.410132
C -1.685131 0.989804 -0.992963
C -2.300872 0.796952 -2.224622
C -2.896402 1.822002 -2.944219
C -2.910967 3.106500 -2.431772
C -2.337401 3.344602 -1.195552
C -1.756987 2.292126 -0.510736
F -2.374147 -0.418716 -2.778632
F -3.465570 1.576435 -4.123126
F -3.477299 4.096415 -3.112527
F -2.355838 4.570527 -0.675511
F -1.243214 2.578443 0.700411
H 1.737561 -1.868866 0.567089
H 4.948029 0.956285 0.587363
H 1.083946 2.757137 0.214947
H 3.697071 5.495406 -1.339472
H 3.640973 3.908056 -2.123611
H 2.203985 4.550032 -1.312852
H 3.591647 5.667246 1.808138
H 2.105862 4.708156 1.794272
H 3.493072 4.173325 2.754926
H 6.163690 4.530139 0.358582
H 6.049779 3.066023 1.340331
H 6.103177 2.949476 -0.428867
H 7.088042 -2.207346 0.455035
H 6.482298 -0.878368 -0.541660
H 6.693542 -0.658014 1.204812
H 4.808637 -4.087221 -0.601511
H 3.121748 -3.587598 -0.450882
H 4.184930 -2.766984 -1.604124
H 5.116332 -3.626774 2.520095
H 4.727898 -2.037735 3.199813
H 3.434331 -3.082445 2.592115

H_2O:
$E_{\omega B97X\text{-}D/6\text{-}311+G(d,p)}$= -76.4391277271

O -0.000000 0.117119 0.000000
H 0.759626 -0.468477 -0.000000
H -0.759626 -0.468477 -0.000000

$(H_2O)_2$:
$E_{\omega B97X\text{-}D/6\text{-}311+G(d,p)}$= -152.886311214

O 1.486207 -0.000824 0.117150
O -1.330877 0.000568 -0.092014
H 1.806906 0.005385 -0.785845
H 0.518631 0.000683 0.039377
H -1.785017 0.761465 0.276945
H -1.783159 -0.765490 0.268440

$(H_2O)_3$:
$E_{\omega B97X\text{-}D/6\text{-}311+G(d,p)}$=

O -1.455465 -0.690994 -0.092017
O 0.124273 1.601227 0.113752
O 1.329841 -0.906541 -0.100739
H -0.665575 1.040991 0.024495
H 1.234913 0.055593 0.002815
H -0.576533 -1.104345 -0.049300
H 0.085067 2.231731 -0.607666
H -1.955891 -1.056233 0.639782
H 1.888822 -1.197271 0.621906

$[H_2O\rightarrow B(C_6F_5)_3]$:
$E_{\omega B97X\text{-}D/6\text{-}311+G(d,p)}$= -2284.73827176

C -2.008013 -0.894371 -0.767038
C -1.143864 -1.058151 0.308248
C -1.301818 -2.248288 1.001689
C -2.248962 -3.208602 0.697500
C -3.095617 -2.993055 -0.375825
C -2.970213 -1.829261 -1.114349
B 0.015327 0.012951 0.695121
O -0.018182 0.049156 2.333720
F -0.483314 -2.523073 2.047625
F -2.348776 -4.324584 1.414121
F -4.014454 -3.895290 -0.696296
F -3.770403 -1.621159 -2.156753
F -1.923178 0.185480 -1.553666
C -0.345011 1.546612 0.311627
C -1.484214 2.147204 0.841945
C -1.870025 3.444116 0.553056
C -1.103992 4.200913 -0.317045
C 0.028227 3.643817 -0.882079
C 0.384652 2.341988 -0.563365
F -2.288196 1.454816 1.660478
F 1.479844 1.872923 -1.175387
F 0.765899 4.359573 -1.728630
F -1.456546 5.446616 -0.609593
F -2.970318 3.965228 1.092348
C 1.515425 -0.472907 0.290616
C 1.799992 -1.374341 -0.727071
C 3.091046 -1.754046 -1.064209
C 4.165569 -1.223736 -0.373683
C 3.934594 -0.313225 0.644017
C 2.631712 0.037295 0.940772
F 0.820672 -1.911745 -1.464787
F 3.304029 -2.622070 -2.049901
F 5.405348 -1.580103 -0.684151
F 4.956718 0.214370 1.311858
F 2.465854 0.955740 1.921665
H 0.043227 -0.823877 2.749359
H 0.660051 0.623588 2.716063

$[H_2O\rightarrow B(C_6F_5)_3]\cdot(H_2O)$:
$E_{\omega B97X\text{-}D/6\text{-}311+G(d,p)}$= -2361.19712619

C 1.787311 1.826938 -0.986460
C 0.644482 1.435569 -0.303707
C 0.252054 2.298602 0.710666
C 0.931923 3.468196 1.014942
C 2.059678 3.816591 0.293061
C 2.496061 2.984870 -0.723458
B -0.061431 0.006152 -0.668144
O -0.181597 -0.005834 -2.245971
F -0.821030 2.031461 1.464983

F 0.508928 4.260130 1.998504
F 2.717612 4.935903 0.573438
F 3.581605 3.300837 -1.426209
F 2.265297 1.041405 -1.980743
C 0.937787 -1.186628 -0.161006
C 1.524167 -2.161225 -0.952298
C 2.401302 -3.120520 -0.467628
C 2.724901 -3.129120 0.875589
C 2.162382 -2.178083 1.711098
C 1.294520 -1.241259 1.181955
F 1.259095 -2.229489 -2.273882
F 0.757733 -0.364328 2.043092
F 2.456071 -2.180517 3.009834
F 3.561995 -4.039490 1.361029
F 2.931177 -4.029249 -1.285121
C -1.619530 -0.147441 -0.221940
C -2.533486 0.843753 -0.571425
C -3.881584 0.788334 -0.267981
C -4.376971 -0.309738 0.415135
C -3.514072 -1.328608 0.771269
C -2.168283 -1.235353 0.444433
F -2.117641 1.932904 -1.232450
F -4.703520 1.774527 -0.622591
F -5.667909 -0.385932 0.716295
F -3.981309 -2.397331 1.415268
F -1.414009 -2.281963 0.807841
O -1.625103 -1.928183 -3.142275
H 0.654368 0.060939 -2.723750
H -0.728287 -0.768313 -2.614365
H -1.340566 -2.826687 -2.954726
H -2.567468 -1.893628 -2.957556

$[H_2O \rightarrow B(C_6F_5)_3]\cdot(H_2O)_2$:
$E_{\omega B97X\text{-}D/6\text{-}311+G(d,p)}$= -2437.65291434

C -0.198758 2.630916 0.887333
C 0.338391 1.583112 0.152588
C 1.160291 1.975847 -0.895842
C 1.461256 3.299295 -1.177272
C 0.919104 4.304377 -0.397390
C 0.074923 3.966002 0.644914
B -0.008944 0.023419 0.500077
O -0.064140 -0.066784 2.072617
F 1.691216 1.067482 -1.729338
F 2.260225 3.611998 -2.197497
F 1.195918 5.579372 -0.652835
F -0.474186 4.923070 1.393083
F -1.069612 2.379722 1.883296
C -1.482908 -0.447961 -0.030736
C -2.271430 0.240104 -0.942835
C -3.529694 -0.191674 -1.340974
C -4.048014 -1.363927 -0.824716
C -3.295099 -2.093282 0.080287
C -2.046501 -1.626850 0.445287
F -1.845473 1.375911 -1.512718
F -1.352964 -2.396503 1.305458
F -3.774753 -3.232242 0.579189
F -5.251439 -1.789149 -1.193569
F -4.240930 0.515246 -2.218282
C 1.212743 -0.975382 0.070185
C 1.144914 -1.951934 -0.914736
C 2.215141 -2.765451 -1.256500
C 3.425141 -2.616230 -0.604492
C 3.547709 -1.647168 0.375739
C 2.456181 -0.853598 0.681716
F 0.023190 -2.145230 -1.622875
F 2.088391 -3.686239 -2.211491

F 4.458954 -3.389858 -0.918000
F 4.709763 -1.484646 1.007877
F 2.648623 0.082809 1.625422
O -2.408340 0.228123 3.227194
O 0.941445 -2.178737 3.240920
H -0.955060 0.089299 2.486619
H 0.329102 -0.886176 2.478736
H 0.971067 -3.001263 2.745425
H 1.791127 -2.097244 3.681678
H -2.960642 0.950024 2.916316
H -2.985220 -0.535418 3.310188

ortho product **3**:

$E_{\omega B97X\text{-}D/6\text{-}311+G(d,p)}$= -3637.00088902

C 0.668566 -1.562589 0.956556
C -0.008779 -0.350756 0.854204
C 0.474689 0.646260 1.688995
C 1.532117 0.467019 2.570243
C 2.165911 -0.757509 2.634713
C 1.724951 -1.785802 1.818607
B -1.209125 -0.194382 -0.258244
C -2.378180 -1.347980 -0.166543
C -2.639985 -2.168333 0.922903
C -3.671097 -3.095513 0.953283
C -4.505703 -3.227420 -0.140389
C -4.298863 -2.419752 -1.244049
C -3.259846 -1.503900 -1.232012
F -1.895010 -2.100869 2.037108
F -3.865855 -3.859719 2.029693
F -5.499263 -4.112504 -0.128817
F -5.106981 -2.522244 -2.301273
F -3.150120 -0.728819 -2.321923
F -0.065993 1.874037 1.683203
F 1.945560 1.470995 3.345662
F 3.185885 -0.948774 3.465023
F 2.316952 -2.980060 1.877156
F 0.301480 -2.600339 0.191107
O -0.554534 -0.400256 -1.620528
P 0.804587 0.008472 -2.245265
C 2.051252 -1.232360 -2.063340
C 3.153598 -1.021908 -1.314408
Si 4.478976 -2.397538 -1.207072
C 5.685826 -2.066026 -2.603059
C 3.433132 0.250838 -0.629758
C 2.754142 1.404746 -0.741184
Si 3.459216 3.000731 0.015987
C 4.262973 3.924667 -1.407671
C 1.532780 1.546813 -1.619247
C 5.356410 -2.280360 0.444605
C 3.641595 -4.058367 -1.410893
C -2.004230 1.246826 -0.218235
C -2.767995 1.559889 0.901684
C -3.537880 2.700683 1.022557
C -3.575042 3.605550 -0.025620
C -2.840475 3.341429 -1.164337
C -2.083263 2.179561 -1.241268
F -2.762533 0.727557 1.955192
F -4.237890 2.941305 2.131125
F -4.306320 4.712869 0.065687
F -2.865541 4.201200 -2.183989
F -1.407475 2.019075 -2.397248
C 2.091442 4.052782 0.743552
C 4.731284 2.558151 1.316784
H 1.857122 -2.182296 -2.550778
H 4.311432 0.244931 0.008240

H 0.747468 2.105555 -1.102146
H 0.523942 0.175436 -3.604664
H 1.783147 2.146423 -2.502905
H 5.108036 3.475036 1.780279
H 4.302690 1.937869 2.108160
H 5.587406 2.029705 0.888344
H 4.704102 4.861023 -1.052374
H 5.056296 3.326546 -1.864898
H 3.534140 4.175161 -2.184443
H 2.504763 5.025128 1.029597
H 1.289374 4.237010 0.022597
H 1.652084 3.598650 1.632967
H 6.078854 -3.098498 0.525532
H 5.910914 -1.343427 0.547384
H 4.664639 -2.365545 1.285352
H 6.495692 -2.801786 -2.591789
H 5.186032 -2.124555 -3.574067
H 6.131932 -1.071682 -2.508108
H 4.378820 -4.858637 -1.295807
H 2.863814 -4.203215 -0.656737
H 3.187659 -4.169876 -2.399523

para product **4**:
$E_{\omega B97X\text{-}D/6\text{-}311+G(d,p)}$= -3637.00092726

C -1.897593 2.269131 -1.045824
C -1.800385 1.256413 -0.102712
C -2.423138 1.544401 1.107615
C -3.046985 2.743221 1.395727
C -3.086585 3.734489 0.429417
C -2.509454 3.491595 -0.800967
B -1.149652 -0.240517 -0.330430
O -0.529214 -0.363908 -1.715998
P 0.844793 0.115668 -2.269512
C 1.457035 1.600665 -1.527031
C 2.578625 1.624645 -0.793144
Si 3.119411 3.275688 0.001739
C 3.724472 2.918628 1.736665
F -2.424387 0.622329 2.084113
F -3.606806 2.955621 2.587000
F -3.677673 4.899315 0.681617
F -2.549849 4.429502 -1.749028
F -1.390379 2.130154 -2.286827
C 0.023061 -0.657838 0.751755
C 0.532098 -1.952962 0.717453
C 1.490491 -2.433612 1.589788
C 1.998160 -1.594530 2.566798
C 1.551481 -0.288834 2.626230
C 0.601148 0.156223 1.716286
F 0.082066 -2.822428 -0.199262
F 0.270731 1.452076 1.825887
F 2.037268 0.532839 3.558290
F 2.908649 -2.036499 3.428620
F 1.921320 -3.693631 1.508460
C -2.438814 -1.267515 -0.335229
C -2.793105 -2.150632 0.676932
C -3.923133 -2.954119 0.633649
C -4.767932 -2.890360 -0.457863
C -4.470703 -2.012706 -1.484690
C -3.335002 -1.224430 -1.399723
F -2.045073 -2.276968 1.784466
F -4.201591 -3.787788 1.637791
F -5.855467 -3.655023 -0.516624
F -5.285759 -1.924355 -2.537932
F -3.142616 -0.367767 -2.413898
C 2.132515 -1.085312 -2.076040

C 3.214288 -0.859123 -1.315744
C 3.457333 0.426506 -0.572162
Si 4.580586 -2.187800 -1.186300
C 3.900971 -3.823691 -1.784880
C 5.992293 -1.595539 -2.269182
C 5.141436 -2.292551 0.598172
C 4.514354 3.949394 -1.053983
C 1.646242 4.430282 0.021092
H 1.978451 -2.032235 -2.584786
H 3.435849 0.194259 0.503092
H 0.599966 0.314618 -3.632406
H 0.853287 2.489436 -1.665189
H 4.091926 3.841373 2.195815
H 2.923508 2.526876 2.367544
H 4.547317 2.197662 1.739120
H 4.884061 4.891680 -0.638446
H 5.354689 3.250098 -1.095556
H 4.176609 4.138313 -2.076704
H 1.903109 5.347004 0.560072
H 1.336359 4.717809 -0.987618
H 0.793073 3.969062 0.527054
H 6.016047 -2.946266 0.669099
H 5.428822 -1.313141 0.991408
H 4.366709 -2.709534 1.245116
H 6.812283 -2.319811 -2.248971
H 5.668932 -1.477787 -3.307094
H 6.387506 -0.635938 -1.923324
H 4.655171 -4.604801 -1.650510
H 3.011137 -4.115809 -1.220745
H 3.642832 -3.793871 -2.847143
H 4.499552 0.731962 -0.737648

ortho product without borane:
$E_{\omega B97X-D/6-311+G(d,p)}$= -1428.6668305

P	7.244355	17.128795	4.483595
Si	5.080442	16.849905	8.463754
Si	9.003289	20.520737	6.749156
O	6.775591	16.776932	3.099713
C	6.132428	16.682302	5.826550
H	5.408296	15.895370	5.635640
C	6.226940	17.299177	7.018477
C	7.224736	18.367900	7.251400
H	7.434616	18.596455	8.294005
C	7.828862	19.110299	6.306125
C	7.537577	18.879534	4.831784
H	8.344327	19.236965	4.187280
H	6.630461	19.425495	4.540113
C	3.862485	15.538974	7.912457
H	4.372555	14.623141	7.600120
H	3.246040	15.889132	7.079536
H	3.193122	15.282627	8.739181
C	4.192012	18.418086	8.985590
H	4.903970	19.195861	9.277706
H	3.537093	18.224149	9.840496
H	3.578397	18.810454	8.169562
C	6.150160	16.215922	9.869885
H	6.697417	15.317139	9.571578
H	5.530515	15.963909	10.736004
H	6.879161	16.966551	10.189080
C	8.352028	22.080544	5.932370
H	9.022376	22.924164	6.122981
H	7.361250	22.340237	6.316400
H	8.272734	21.956222	4.848149
C	9.081584	20.726586	8.610422
H	8.098369	20.958442	9.030054
H	9.756405	21.549749	8.864238

H	9.459491	19.824019	9.099511
C	10.701370	20.105447	6.062949
H	11.406717	20.916963	6.267109
H	10.670174	19.957460	4.979332
H	11.094580	19.191834	6.518147
H	8.482545	16.526192	4.823478

3.5.2 NMR chemical shift calculations

2,3,5,6-tetra-pyridyl-1,2-dihydro-phosphinine oxide

SCF Done: E(RB3LYP) = -1600.29414165
Sum of electronic and thermal Energies= -1599.884533
Sum of electronic and thermal Enthalpies= -1599.883589
Sum of electronic and thermal Free Energies= -1599.967556
Lowest frequency: 15.8855 cm^{-1}
Second lowest frequency: 26.8970 cm^{-1}

C	-1.26148600	0.69950800	-0.07654100
C	-1.43835700	-0.63902600	-0.07493200
C	0.05387600	1.33267500	-0.15856200
C	1.23682100	0.75457800	0.11629300
C	1.36241200	-0.71647700	0.47912000
P	0.01854600	-1.68394300	-0.31753300
C	-2.41279400	1.64000900	0.02741200
C	-3.42247900	1.45351200	0.97405500
H	-3.37740800	0.61872900	1.65775000
C	-4.46789500	2.36153700	1.02071100
H	-5.26050300	2.24370800	1.74825100
C	-4.47835400	3.42222200	0.12495200
H	-5.27618700	4.15249300	0.12515600
C	-3.42216100	3.53567200	-0.77044500
H	-3.38549200	4.35662200	-1.47853900
N	-2.40681600	2.67686400	-0.81640400
C	-2.75639800	-1.31945200	-0.05859500
N	-3.07425200	-1.99573000	1.04915600
C	-4.24166300	-2.63452400	1.07934900
H	-4.46678900	-3.17155300	1.99453300
C	-5.14317900	-2.63548000	0.02154800
H	-6.07715200	-3.17536700	0.09961500
C	-4.80857300	-1.93353300	-1.12807200
H	-5.47992300	-1.90778200	-1.97676800
C	-3.59477100	-1.26544000	-1.17258300
H	-3.29408000	-0.70390500	-2.04651000
C	2.44714300	1.60837600	0.17560900
N	2.49697400	2.65659200	-0.65724700
C	3.55181800	3.46292900	-0.60433200
H	3.55068200	4.29447900	-1.30147300
C	4.61390100	3.28244600	0.27380400
H	5.45109900	3.96707500	0.27118500
C	4.56122600	2.20321100	1.14472300
H	5.35916200	2.02404400	1.85377000
C	3.47025400	1.35138100	1.09546100
H	3.41008900	0.50426200	1.76260900
C	2.67528000	-1.36604900	0.10586000
C	3.20594100	-1.23600300	-1.17802800
H	2.70170800	-0.62834900	-1.91711700
C	4.39497900	-1.87951200	-1.47925700
H	4.82947200	-1.79202700	-2.46668000
C	5.01796500	-2.63317000	-0.49332400
H	5.94654800	-3.15367600	-0.68412600
C	4.41781700	-2.70015400	0.75679700
H	4.87339300	-3.27316400	1.55712700
N	3.27657300	-2.08169100	1.05745500
O	-0.09771200	-3.10690800	0.05456800
H	0.30932900	-1.45873000	-1.68767000

H	1.22500400	-0.86076300	1.55665000
H	0.04926200	2.39064000	-0.37817400

ortho 1,2-dihydrophosphinine oxide 3

SCF Done: E(RB3LYP) = -3638.39182064
Sum of electronic and thermal Energies= -3637.872460
Sum of electronic and thermal Enthalpies= -3637.871516
Sum of electronic and thermal Free Energies= -3638.010716
Lowest frequency: 11.4080 cm^{-1}
Second lowest frequency: 23.2530 cm^{-1}

P	0.76360700	0.12575100	-2.23107200
Si	4.15387000	-2.66918500	-1.16126700
Si	3.63645300	2.87757900	-0.13550000
F	-1.30731000	1.97290200	-2.42610700
F	0.52804000	-2.33137000	0.47140800
F	-0.30200000	2.20083500	1.60042100
F	2.55536500	-2.37135100	2.20435100
F	-1.79091700	-1.90521500	2.23750600
F	-2.92901400	4.05774300	-2.45542500
F	-2.96715600	-0.99279300	-2.26785200
F	-2.98226000	0.76636600	1.84648200
F	-4.74322500	-2.98172200	-2.18801400
F	3.17555000	-0.14520800	3.64716500
F	1.70418600	2.12372000	3.31828600
F	-5.07672200	-4.44255700	0.08937100
F	-4.61674300	4.55578700	-0.36980800
F	-4.62170700	2.87829700	1.78010300
F	-3.57914200	-3.86637000	2.28980500
O	-0.44359300	-0.43550100	-1.48782700
C	3.13094000	-1.09242200	-1.38072800
C	-0.03032600	-0.08397200	0.97396300
C	-2.08625400	1.23472300	-0.28897200
C	-3.06043400	-1.69761600	-1.12291600
C	2.14217000	-0.13104000	2.80282500
C	0.76463600	-1.20846700	1.17606300
C	-2.25292300	-1.37541600	-0.03519100
C	1.82222200	-1.25835400	2.06622200
C	-2.96145700	1.54398700	0.74948900
C	0.33452500	1.02252000	1.72962900
C	3.41245000	0.03710000	-0.70533900
H	4.22124000	0.00448600	0.01346600
C	-2.12191600	2.12634100	-1.34773700
C	-3.80975600	3.49441400	-0.34341500
C	-2.47871200	-2.13601300	1.10505900
C	2.75807800	1.34015600	-0.84761400
C	1.58709700	1.50476800	-1.50487700
H	1.09277200	2.46513200	-1.56089800
C	-4.17683000	-3.45809000	0.04987300
C	-2.95334900	3.23707200	-1.39874800
C	-3.81147000	2.63583900	0.74654100
C	-4.00124700	-2.71530800	-1.10676100
C	1.38993700	1.01804700	2.63122900
C	2.05844400	-1.10721500	-2.44539200
H	1.58393300	-2.08603800	-2.53792700
H	2.52072200	-0.89100600	-3.41720300
C	-3.41182700	-3.16195700	1.16549300
C	3.02119700	-4.11095800	-0.78638500
H	3.60579900	-5.03256000	-0.73100700
H	2.26635500	-4.25128100	-1.56295300
H	2.50393200	-3.97830100	0.16278900
C	5.40646100	-2.41185300	0.20439200
H	5.99702600	-3.32123000	0.33734200
H	4.92344600	-2.19232300	1.15746100
H	6.10115100	-1.60200700	-0.02724900

C	5.02042100	-2.96358800	-2.80357400
H	5.67306400	-2.12803300	-3.06489500
H	4.30523700	-3.10093900	-3.61787100
H	5.63477700	-3.86566600	-2.75482700
C	2.35940200	4.17859100	0.27678200
H	1.81091600	4.51094600	-0.60669200
H	2.85301900	5.05625200	0.70051800
H	1.63624300	3.81747900	1.00874700
C	4.63723100	2.40536100	1.37453200
H	4.01937400	2.02648200	2.18750600
H	5.15724000	3.29116100	1.74768800
H	5.39825400	1.65698000	1.14617100
B	-1.19855900	-0.13459200	-0.17125500
C	4.78780600	3.47194700	-1.49376600
H	5.50709600	2.69823600	-1.77063300
H	5.35106300	4.34826200	-1.16444800
H	4.22930100	3.74847400	-2.39017800
H	0.34936200	0.47552000	-3.51849000

para 1,4-dihydrophosphinine oxide 4

SCF Done: E(RB3LYP) = -3638.38856357
Sum of electronic and thermal Energies= -3637.869151
Sum of electronic and thermal Enthalpies= -3637.868207
Sum of electronic and thermal Free Energies= -3638.008143
Lowest frequency: 8.9763 cm^{-1}
Second lowest frequency: 21.6347 cm^{-1}

C	-1.90273100	2.25911400	-1.07135300
C	-1.82877300	1.26183900	-0.10907400
C	-2.46802600	1.57159500	1.08876400
C	-3.10029200	2.77568900	1.34234800
C	-3.12304500	3.75001800	0.35476600
C	-2.51956900	3.48633500	-0.86142500
B	-1.18617700	-0.23457700	-0.28793000
O	-0.53462100	-0.37670800	-1.67496800
P	0.81805200	0.09686300	-2.21697400
C	1.46281300	1.57116200	-1.49621300
C	2.61086200	1.59976600	-0.80576600
Si	3.17085500	3.24596400	-0.02737200
C	3.92839100	2.89216400	1.64653100
F	-2.47357200	0.66589200	2.08324400
F	-3.68073800	3.01275900	2.52194500
F	-3.72042200	4.92223400	0.57464600
F	-2.53386400	4.41279900	-1.82737100
F	-1.35820300	2.10117800	-2.30311000
C	-0.00556700	-0.62085100	0.79010200
C	0.52668800	-1.90788300	0.76417700
C	1.52331600	-2.35177900	1.61440900
C	2.04767700	-1.48283900	2.55796300
C	1.57419500	-0.18436800	2.60833100
C	0.58309600	0.22294000	1.72320200
F	0.07858800	-2.80051900	-0.13605500
F	0.23756300	1.52096600	1.81339200
F	2.08628400	0.67255600	3.50141000
F	3.01054400	-1.88657700	3.38912800
F	1.99487500	-3.60295300	1.52973200
C	-2.45217800	-1.27032400	-0.29482600
C	-2.80369100	-2.13709900	0.73317000
C	-3.92027600	-2.96097200	0.69015500
C	-4.74999400	-2.93234000	-0.41768800
C	-4.45280800	-2.07070200	-1.46144800
C	-3.33034000	-1.26199400	-1.37642200
F	-2.06153800	-2.22351300	1.85255200

F	-4.20076800	-3.78235100	1.70769500
F	-5.82627700	-3.71894400	-0.47701600
F	-5.25474400	-2.02076200	-2.53130400
F	-3.12731600	-0.41977700	-2.40671000
C	2.09815000	-1.10678400	-2.04580800
C	3.20453000	-0.89443700	-1.31904500
C	3.49410100	0.40209900	-0.61742700
Si	4.52219100	-2.26186500	-1.17372400
C	3.75066800	-3.90646500	-1.61159400
C	5.88271400	-1.80700100	-2.38513500
C	5.20438100	-2.27502800	0.56999000
C	4.46201600	3.97017500	-1.18183400
C	1.68573600	4.37115300	0.12483800
H	1.90822400	-2.06872800	-2.50874500
H	3.53211500	0.18944400	0.45871400
H	0.58657800	0.30736400	-3.58040500
H	0.84829100	2.45475900	-1.59419600
H	4.29094500	3.82207100	2.09099500
H	3.20444200	2.45671900	2.33530200
H	4.78058200	2.21302600	1.57485600
H	4.83959400	4.91629200	-0.78692400
H	5.31632000	3.30036100	-1.30271300
H	4.04315000	4.16343600	-2.17125500
H	1.97059700	5.29777300	0.62818000
H	1.26928500	4.64490600	-0.84643300
H	0.89782000	3.89860500	0.71376500
H	6.02632000	-2.99215700	0.63394900
H	5.60098200	-1.30124400	0.86396800
H	4.45571100	-2.57290200	1.30349100
H	6.68637000	-2.54635400	-2.35146200
H	5.50433500	-1.76945100	-3.40844800
H	6.32180600	-0.83421400	-2.15312300
H	4.48413800	-4.70530900	-1.48045600
H	2.89876300	-4.12907300	-0.96717100
H	3.41521400	-3.94143700	-2.64992500
H	4.52688600	0.69633000	-0.84429500

3.5.3 Formal insertion of the P atom into the H-O bond of H_2O

Without borane

vdW_insertion_H_2O:
$E_{\omega B97X\text{-}D/6\text{-}311+G(d,p)}$= -1428.66654719

C -4.333867 0.000228 -0.049174
Si -2.810690 -1.090906 0.002314
C -1.249486 -0.009277 -0.009698
C -1.334418 1.384151 -0.017467
P 0.005988 2.475071 -0.023410
C 1.340525 1.377015 -0.017347
C 1.248113 -0.015952 -0.009649
Si 2.803502 -1.105893 0.002315
C 4.332451 -0.022877 -0.049354
C -2.757655 -2.214939 -1.500451
C -2.794837 -2.122773 1.570444
C -0.002381 -0.648123 -0.005957
C 2.782162 -2.137636 1.570460
C 2.744321 -2.229730 -1.500361
O 0.024799 5.979927 0.124391
H 0.017765 5.014782 0.095435
H 0.028072 6.248682 -0.796175
H -0.005296 -1.737930 0.000206
H -3.630363 -2.875040 -1.519325
H -2.752597 -1.631639 -2.425767

H -1.862271 -2.843850 -1.493406
H -5.234928 -0.620606 -0.049369
H -4.385494 0.661507 0.820770
H -4.355204 0.619101 -0.951052
H -3.665955 -2.784178 1.607490
H -1.897332 -2.746387 1.623377
H -2.814371 -1.484545 2.458561
H 3.649587 -2.803876 1.607459
H 2.805278 -1.499529 2.458579
H 1.881202 -2.756250 1.623434
H 3.613121 -2.894978 -1.518879
H 1.845237 -2.853336 -1.493489
H 2.742978 -1.646548 -2.425764
H 5.230216 -0.648466 -0.050022
H 4.356762 0.596157 -0.951047
H 4.387847 0.637860 0.820773
H 2.327907 1.832897 -0.019508
H -2.319319 1.845369 -0.019747

TS_insertion_H_2O:
$E_{\omega B97X\text{-}D/6\text{-}311+G(d,p)}$= -1428.57147847

C -4.320545 0.001499 -0.264918
Si -2.776177 -1.009799 0.050277
C -2.642516 -2.420792 -1.178259
C -2.757772 -1.670850 1.804907
C -1.240800 0.098060 -0.162735
C 0.023225 -0.494527 -0.023388
C 1.262136 0.146223 -0.151659
C 1.360580 1.513796 -0.427295
P -0.034751 2.466657 -0.585026
O -0.350923 4.276970 0.930718
C -1.393941 1.457639 -0.439977
Si 2.838319 -0.902508 0.059947
C 2.851810 -1.563809 1.814579
C 2.756426 -2.318219 -1.167478
C 4.342387 0.167253 -0.259920
H -0.048396 3.901507 -0.634081
H 0.350651 4.588606 1.511396
H 0.042553 -1.560253 0.195734
H -3.498069 -3.095874 -1.079129
H -2.622563 -2.046364 -2.205683
H -1.733187 -3.006291 -1.013537
H -5.205445 -0.632073 -0.152035
H -4.412613 0.829338 0.444171
H -4.333435 0.413490 -1.278278
H -3.613228 -2.331382 1.976330
H -1.846246 -2.242913 2.001696
H -2.807527 -0.853775 2.530179
H 3.733869 -2.189230 1.983028
H 2.870450 -0.746187 2.540716
H 1.964881 -2.172610 2.013545
H 3.638778 -2.958414 -1.072083
H 1.872206 -2.939761 -0.998242
H 2.716457 -1.945552 -2.194947
H 5.250869 -0.433803 -0.157125
H 4.332622 0.585072 -1.270958
H 4.409796 0.993919 0.453397
H 2.326000 1.997194 -0.525847
H -2.375698 1.904384 -0.544883

PRO_insertion_H_2O:
$E_{\omega B97X\text{-}D/6\text{-}311+G(d,p)}$= -1428.65018934

C -4.329573 0.125412 -0.305145
Si -2.797057 -0.906049 0.024786
C -1.241056 0.169288 -0.114652

C -1.369828 1.533403 -0.377438
P -0.000023 2.574766 -0.370026
C 1.369791 1.533343 -0.377452
C 1.241064 0.169270 -0.114679
Si 2.797098 -0.906006 0.024791
C 4.329501 0.125299 -0.306133
C -2.678084 -2.300064 -1.228672
C -2.864851 -1.622876 1.760135
C -0.000026 -0.468859 0.033860
C 2.865410 -1.621955 1.760482
C 2.677723 -2.300665 -1.227915
O -0.000078 3.698999 0.843738
H 0.000140 3.558966 -1.371018
H -0.000459 3.291283 1.716844
H -0.000012 -1.538298 0.232168
H -3.552060 -2.955773 -1.163578
H -2.623877 -1.905517 -2.247557
H -1.785916 -2.909297 -1.056293
H -5.223485 -0.501607 -0.231645
H -4.431673 0.937948 0.420302
H -4.310261 0.563464 -1.307498
H -3.739966 -2.269191 1.880655
H -1.972610 -2.219595 1.971958
H -2.923846 -0.827750 2.509246
H 3.740542 -2.268237 1.881060
H 2.924657 -0.826457 2.509178
H 1.973214 -2.218542 1.972863
H 3.551825 -2.956213 -1.162902
H 1.785727 -2.909942 -1.054802
H 2.622974 -1.906647 -2.246976
H 5.223365 -0.501882 -0.233436
H 4.309496 0.563536 -1.308391
H 4.432274 0.937684 0.419386
H 2.346539 1.983907 -0.518823
H -2.346627 1.983877 -0.518699

With borane

vdW_insertion_H$_2$O_B:
$E_{\omega B97X\text{-}D/6\text{-}311+G(d,p)}$= -3636.98749739

P 1.740608 0.151536 2.373716
H -0.522791 -0.324586 2.451127
O -1.417377 -0.598545 2.141212
C 2.995313 -0.924165 1.872694
C 3.948847 -0.646127 0.891787
C 3.904350 0.555285 0.170181
H 4.655192 0.691440 -0.604862
C 2.976267 1.590320 0.343926
C 1.985809 1.495711 1.320474
H 1.289920 2.321748 1.437552
Si 3.063785 3.130782 -0.766800
H 3.040438 -1.878238 2.392627
Si 5.369508 -1.869819 0.571657
C 5.864568 -1.785270 -1.235562
C 4.817523 -3.601507 1.034580
C 6.813017 -1.351224 1.653430
C 1.395278 3.985987 -0.727654
C 4.407208 4.260800 -0.104058
C 3.469590 2.595730 -2.517171
H 6.635965 -2.533364 -1.442929
H 5.016749 -1.982357 -1.895801
H 6.278426 -0.806291 -1.494301
H 7.667448 -2.019270 1.506911
H 6.538134 -1.378466 2.711952

H 7.133644 -0.333108 1.413286
H 5.624020 -4.310998 0.825274
H 4.581385 -3.679306 2.099790
H 3.940339 -3.918172 0.465340
H 3.525789 3.471463 -3.171158
H 4.430969 2.077141 -2.574077
H 2.699986 1.925607 -2.907692
H 1.382145 4.819819 -1.435706
H 0.602207 3.288570 -1.011331
H 1.164273 4.390539 0.261989
H 4.493363 5.163543 -0.716672
H 4.189521 4.566351 0.923494
H 5.377659 3.755387 -0.108315
B -1.648505 -0.370321 0.556090
H -1.534250 -1.527424 2.384609
C -3.106899 -1.050739 0.289401
C -0.386803 -1.123559 -0.153997
C -1.760916 1.235281 0.313005
C 0.494606 -0.575429 -1.076548
C 1.549277 -1.286183 -1.631922
C 1.762150 -2.600037 -1.262928
C 0.923801 -3.187701 -0.331241
C -0.116016 -2.443712 0.187704
F 0.368813 0.689454 -1.492978
F 2.356405 -0.714144 -2.521654
F 2.762920 -3.293598 -1.794445
F 1.121822 -4.449899 0.042445
F -0.911291 -3.068197 1.086138
C -1.518782 2.237099 1.240079
C -1.710409 3.585527 0.977184
C -2.162231 3.981041 -0.266726
C -2.416320 3.018301 -1.229215
C -2.216032 1.685168 -0.921901
F -1.065368 1.944161 2.472306
F -1.447992 4.499644 1.910045
F -2.336285 5.268527 -0.541789
F -2.842259 3.383521 -2.436601
F -2.470922 0.798160 -1.894586
C -4.199425 -0.665840 1.062530
C -5.482299 -1.151190 0.873029
C -5.719228 -2.059075 -0.143872
C -4.670214 -2.458643 -0.951803
C -3.399080 -1.953289 -0.726764
F -4.042812 0.229948 2.047317
F -6.485507 -0.748852 1.651072
F -6.941445 -2.536672 -0.345669
F -4.890148 -3.322371 -1.941483
F -2.449478 -2.373231 -1.571989

TS_insertion_H_2O_B:
$E_{\omega B97X\text{-}D/6\text{-}311+G(d,p)}$= -3636.95740857

P -1.696851 0.307891 -2.341513
H -0.644869 0.145973 -3.238859
O 1.227333 -0.533461 -2.120004
C -2.844405 -0.877526 -1.989621
C -3.778891 -0.621785 -0.981291
C -3.749481 0.564423 -0.230687
H -4.495972 0.652662 0.553156
C -2.865313 1.644968 -0.366277
C -1.859599 1.648870 -1.333917
H -1.179660 2.482017 -1.451292
Si -3.017311 3.156128 0.799841
H -2.843661 -1.801230 -2.556448
Si -5.187299 -1.884031 -0.679773
C -5.725102 -1.764300 1.108374

C -4.562587 -3.596175 -1.103417
C -6.583429 -1.388806 -1.826425
C -1.362877 4.030744 0.803931
C -4.379390 4.243305 0.114375
C -3.443687 2.535308 2.511275
H -6.468117 -2.541032 1.313142
H -4.891261 -1.908764 1.798784
H -6.191495 -0.799809 1.327737
H -7.434622 -2.065611 -1.704933
H -6.267160 -1.426618 -2.872849
H -6.924945 -0.372539 -1.609608
H -5.354752 -4.325671 -0.909434
H -4.293622 -3.682713 -2.159936
H -3.696194 -3.882046 -0.503314
H -3.520824 3.384269 3.197479
H -4.399701 2.004964 2.533414
H -2.670056 1.861391 2.885475
H -1.371069 4.837082 1.542924
H -0.562966 3.335599 1.073511
H -1.124622 4.478868 -0.164976
H -4.511808 5.131410 0.739605
H -4.145311 4.574511 -0.901294
H -5.331148 3.704503 0.086972
B 1.526377 -0.379553 -0.683741
H 1.388628 -1.437877 -2.388617
C 3.009793 -1.056724 -0.341011
C 0.296775 -1.130799 0.159736
C 1.685110 1.226211 -0.325847
C -0.566311 -0.584908 1.097768
C -1.590903 -1.296417 1.710525
C -1.793590 -2.622293 1.386132
C -0.974588 -3.212828 0.439022
C 0.029601 -2.462704 -0.141288
F -0.460171 0.699274 1.483610
F -2.383301 -0.710156 2.610661
F -2.762559 -3.324908 1.972356
F -1.156384 -4.494160 0.111925
F 0.801922 -3.113028 -1.037532
C 1.508372 2.275481 -1.216191
C 1.764122 3.604290 -0.901696
C 2.219363 3.936347 0.358336
C 2.408434 2.927613 1.287229
C 2.142752 1.618624 0.927893
F 1.045824 2.073528 -2.465456
F 1.556109 4.566614 -1.805099
F 2.454714 5.206217 0.681378
F 2.836715 3.228850 2.514690
F 2.343502 0.693643 1.881185
C 4.105370 -0.688688 -1.117408
C 5.387473 -1.180222 -0.930185
C 5.626987 -2.084921 0.087982
C 4.577752 -2.469987 0.900670
C 3.310499 -1.951786 0.677452
F 3.967569 0.209616 -2.106242
F 6.393815 -0.784905 -1.714709
F 6.850902 -2.571513 0.286523
F 4.795155 -3.333603 1.896306
F 2.366015 -2.368080 1.538985

λ⁵-P_insertion_H_2O_B:
$E_{\omega B97X\text{-}D/6\text{-}311+G(d,p)}$= -3636.96708810

P 1.105735 0.241246 2.456513
H 0.554732 0.316267 3.736905
O -0.629162 -0.389594 1.859451
C 2.306770 -0.921107 2.177564

C 3.276457 -0.686687 1.197784
C 3.346667 0.512771 0.474476
H 4.115654 0.590375 -0.287155
C 2.549824 1.642669 0.697155
C 1.536821 1.670844 1.658983
H 0.961182 2.566863 1.849574
Si 2.875367 3.251471 -0.270218
H 2.265645 -1.852110 2.730641
Si 4.680940 -1.958250 1.000537
C 5.431545 -1.813282 -0.710509
C 4.033399 -3.688239 1.313664
C 5.968881 -1.523860 2.294963
C 1.235631 4.125068 -0.535259
C 4.014943 4.318589 0.770744
C 3.678649 2.835007 -1.909531
H 6.214935 -2.568451 -0.827935
H 4.697186 -1.971297 -1.502977
H 5.893402 -0.834022 -0.864459
H 6.817558 -2.212867 2.242247
H 5.546154 -1.582759 3.302213
H 6.345734 -0.508057 2.144177
H 4.855836 -4.404922 1.224885
H 3.620659 -3.788453 2.321761
H 3.260167 -3.978462 0.599690
H 3.829696 3.751511 -2.488224
H 4.656786 2.364196 -1.775887
H 3.051434 2.160685 -2.496915
H 1.374297 4.993566 -1.186197
H 0.508587 3.458508 -1.006125
H 0.812224 4.487272 0.406159
H 4.220836 5.269058 0.268823
H 3.562716 4.539716 1.742103
H 4.969410 3.814312 0.947892
B -1.186421 -0.285148 0.410099
H -1.311205 -0.239285 2.527481
C -2.588928 -1.153073 0.361110
C 0.005194 -0.937493 -0.497270
C -1.621630 1.261243 0.059046
C 0.776156 -0.296532 -1.455257
C 1.722038 -0.946450 -2.236888
C 1.936108 -2.298795 -2.067564
C 1.217829 -2.977887 -1.098010
C 0.295818 -2.290118 -0.334408
F 0.640593 1.014892 -1.694955
F 2.424764 -0.273716 -3.148526
F 2.821640 -2.943781 -2.822097
F 1.409563 -4.287258 -0.926634
F -0.363996 -3.010871 0.584913
C -1.721786 2.304651 0.967448
C -2.148183 3.581777 0.632912
C -2.522092 3.856520 -0.667453
C -2.477087 2.840261 -1.607335
C -2.048883 1.582400 -1.225195
F -1.404573 2.132126 2.268986
F -2.195504 4.542739 1.555592
F -2.925018 5.075341 -1.011546
F -2.848831 3.082590 -2.863366
F -2.054509 0.635374 -2.174610
C -3.530763 -1.017332 1.372174
C -4.764887 -1.640796 1.387812
C -5.116081 -2.452795 0.324234
C -4.223283 -2.616055 -0.719039
C -2.994125 -1.973457 -0.685958
F -3.256428 -0.221083 2.434336
F -5.609871 -1.464424 2.401950
F -6.295093 -3.065242 0.305147
F -4.551018 -3.393466 -1.750039

F -2.200891 -2.194331 -1.742407

preTS_Isomertisation_of_**λ⁵-P**_to_**3**_H2O_B:
$E_{\omega B97X-D/6-311+G(d,p)}$= -3636.95433387

C -1.528794 -1.675141 -2.098806
C -2.049596 -0.701225 -1.262655
C -3.387991 -0.405301 -1.497052
C -4.158720 -1.030772 -2.460652
C -3.589341 -2.009115 -3.258055
C -2.257778 -2.332054 -3.076835
B -1.263277 0.079546 -0.052276
C -2.120576 -0.167229 1.323365
C -2.894493 0.780503 1.982435
C -3.639525 0.503230 3.117704
C -3.644216 -0.778095 3.638376
C -2.910405 -1.764501 3.005204
C -2.182983 -1.446127 1.869929
F -2.972457 2.039203 1.531433
F -4.356033 1.458904 3.708720
F -4.351959 -1.059676 4.727284
F -2.917535 -3.008706 3.483497
F -1.525492 -2.464426 1.290714
F -3.988891 0.559313 -0.785175
F -5.435991 -0.696713 -2.633665
F -4.310928 -2.623029 -4.189495
F -1.687162 -3.260905 -3.841536
F -0.221867 -2.031273 -2.014994
C -0.881394 1.635376 -0.403523
C -0.335216 2.460814 0.572728
C 0.106382 3.750692 0.350081
C 0.013080 4.282441 -0.925450
C -0.513350 3.504930 -1.938414
C -0.940822 2.211293 -1.666007
F -0.195123 1.998945 1.832090
F -1.413744 1.531712 -2.720497
F -0.596800 3.997175 -3.173308
F 0.434132 5.517724 -1.171104
F 0.626444 4.477119 1.338550
O 0.115035 -0.630765 0.177125
P 1.512475 -0.555124 1.458354
C 2.366254 -2.003518 1.206077
C 3.594256 -1.956827 0.545485
Si 4.484945 -3.584508 0.114731
C 3.490611 -5.031351 0.768742
C 2.552400 0.746945 1.181607
C 3.747928 0.527918 0.481892
C 4.216159 -0.751471 0.172759
Si 4.674186 2.083810 -0.101988
C 4.830074 3.268525 1.343060
C 6.361432 1.609665 -0.762382
C 3.612878 2.845717 -1.450582
C 4.624266 -3.667873 -1.753811
C 6.188878 -3.540780 0.896175
H 2.231933 1.744041 1.454531
H 5.158863 -0.822439 -0.361785
H 0.439686 -1.097763 -0.604372
H 1.901059 -2.939708 1.490423
H 6.743999 -4.452786 0.656070
H 6.769448 -2.688196 0.531977
H 6.118818 -3.462282 1.984830
H 4.001778 -5.968561 0.528879
H 3.375849 -4.984265 1.855672
H 2.494150 -5.072695 0.319086
H 5.143205 -4.581216 -2.060428
H 3.634855 -3.666521 -2.220194

H 5.185016 -2.814506 -2.146136
H 6.903352 2.507520 -1.074412
H 6.961187 1.103183 -0.000640
H 6.283064 0.950377 -1.631614
H 5.360972 4.173255 1.031485
H 3.851161 3.575167 1.722955
H 5.387886 2.814685 2.167013
H 4.035745 3.792404 -1.799691
H 3.527766 2.172540 -2.308521
H 2.603038 3.043394 -1.078213
H 0.572602 -0.458948 2.482770

TS_Isomertisation_of_**λ⁵-P**_to_**3**_H_2O_B:
$E_{\omega B97X\text{-}D/6\text{-}311+G(d,p)}$= -3636.91595342

C -0.021691 -1.581494 1.958328
C -0.456463 -1.459674 0.645025
C -0.207158 -2.576563 -0.143965
C 0.389223 -3.736622 0.309482
C 0.787120 -3.815147 1.633586
C 0.579923 -2.728465 2.460927
B -1.111647 -0.111151 -0.014126
O 0.010199 0.475436 -0.932337
P 1.083738 0.017299 -2.139647
C 2.276057 -1.111661 -1.650250
C 3.326323 -0.634340 -0.890972
Si 4.536873 -1.878447 -0.100963
C 4.168699 -3.598525 -0.742040
F -0.532034 -2.550828 -1.451929
F 0.592460 -4.768360 -0.510152
F 1.370395 -4.914894 2.096765
F 0.968401 -2.784457 3.734487
F -0.151493 -0.575191 2.833373
C -1.516126 1.038844 1.080125
C -2.616320 0.803158 1.898878
C -3.093497 1.701022 2.835796
C -2.455059 2.919131 2.992959
C -1.351974 3.201343 2.211300
C -0.908567 2.268606 1.284616
F -3.257615 -0.373705 1.822803
F 0.185243 2.637407 0.590122
F -0.719061 4.366303 2.352244
F -2.893902 3.800746 3.886159
F -4.150557 1.403293 3.590532
C -2.422537 -0.343052 -0.975848
C -2.813664 0.675230 -1.839941
C -3.919415 0.600689 -2.670679
C -4.709653 -0.534051 -2.645826
C -4.378887 -1.565026 -1.785552
C -3.261409 -1.450581 -0.973145
F -2.115673 1.822844 -1.893321
F -4.232631 1.611249 -3.482499
F -5.777239 -0.629000 -3.432567
F -5.140279 -2.658639 -1.741807
F -3.032459 -2.487240 -0.155245
C 1.735553 1.648534 -2.052749
C 2.927448 1.807209 -1.280075
C 3.570995 0.751849 -0.661052
Si 3.623329 3.562968 -1.021047
C 2.484604 4.800911 -1.845310
C 3.702082 3.865667 0.827182
C 5.338443 3.622629 -1.776118
C 6.285072 -1.368015 -0.543199
C 4.259126 -1.779876 1.750130
H 2.159901 -2.158903 -1.898076
H 4.417779 0.991641 -0.022893
H 0.613698 1.514063 -1.063293

H 1.443553 2.388741 -2.790056
H 5.785368 4.612071 -1.639026
H 5.997537 2.885953 -1.307526
H 5.303012 3.411522 -2.848718
H 2.847103 5.816870 -1.662218
H 2.442256 4.655423 -2.928747
H 1.467563 4.731507 -1.448944
H 4.102337 4.863284 1.032433
H 2.706754 3.798645 1.274338
H 4.348235 3.136228 1.323843
H 7.005806 -2.058430 -0.094542
H 6.434142 -1.379021 -1.626596
H 6.513896 -0.362502 -0.179203
H 4.873957 -4.311600 -0.304920
H 3.161540 -3.928959 -0.475718
H 4.270212 -3.653455 -1.829802
H 4.914503 -2.478953 2.278057
H 4.460865 -0.773579 2.127954
H 3.224534 -2.031169 1.997926
H 0.271686 -0.386032 -3.202444

postTS_Isomertisation_of_**λ⁵-P**_to_**3**_H_2O_B:
$E_{\omega B97X\text{-}D/6\text{-}311+G(d,p)}$= -3636.99668009

C -0.191685 -1.618109 1.528057
C 0.869842 -0.765426 1.268421
C 1.768106 -0.628292 2.320663
C 1.646979 -1.279641 3.534127
C 0.571907 -2.127044 3.741645
C -0.354030 -2.292846 2.730535
B 1.164564 0.015288 -0.144757
C 2.648188 -0.489013 -0.651396
C 3.816914 0.260443 -0.655834
C 5.044455 -0.235710 -1.068206
C 5.144699 -1.547442 -1.492176
C 4.013673 -2.343488 -1.485081
C 2.807278 -1.808869 -1.063257
F 3.822305 1.536467 -0.239639
F 6.129904 0.540531 -1.056523
F 6.314336 -2.040429 -1.892141
F 4.097418 -3.617684 -1.873825
F 1.766605 -2.656474 -1.046107
F 2.813860 0.206447 2.193884
F 2.544203 -1.095404 4.504228
F 0.430829 -2.766645 4.900443
F -1.401719 -3.099812 2.916307
F -1.155511 -1.831709 0.621103
C 0.982472 1.645995 -0.051120
C 1.291988 2.438686 -1.152460
C 1.078553 3.803830 -1.213451
C 0.522336 4.448360 -0.121148
C 0.191560 3.708382 0.998501
C 0.416912 2.338474 1.011760
F 1.806937 1.871361 -2.258384
F 0.022375 1.708723 2.126669
F -0.355772 4.313866 2.052067
F 0.304249 5.757989 -0.152370
F 1.397608 4.499663 -2.303282
O 0.190955 -0.477263 -1.220096
P -0.978182 0.096278 -2.054882
C -2.123059 -1.213283 -2.498434
C -3.219578 -1.431251 -1.477979
Si -3.996378 -3.158063 -1.416417
C -2.666138 -4.382341 -0.919352
C -1.979948 1.318757 -1.255565
C -3.130760 0.946937 -0.655539

C -3.640181 -0.433915 -0.681611
Si -4.119145 2.225638 0.357552
C -3.510189 3.944355 -0.063490
C -5.932825 2.033693 -0.070933
C -3.795777 1.807903 2.155249
C -5.399664 -3.160146 -0.177435
C -4.615103 -3.548543 -3.144808
H -1.618036 2.340519 -1.225953
H -4.454423 -0.641954 0.007001
H -1.544063 -2.119236 -2.693973
H -2.571671 -0.912170 -3.454909
H -5.057602 -4.548763 -3.175368
H -5.376443 -2.830688 -3.463266
H -3.799005 -3.524813 -3.873591
H -3.070721 -5.399169 -0.930082
H -1.814251 -4.357064 -1.605293
H -2.295767 -4.169817 0.086077
H -5.862480 -4.151056 -0.146226
H -5.042721 -2.927998 0.830167
H -6.175742 -2.437069 -0.444213
H -6.531019 2.751739 0.498028
H -6.107355 2.216510 -1.134983
H -6.300346 1.031610 0.167347
H -4.100538 4.687798 0.480063
H -2.465043 4.086751 0.221707
H -3.607362 4.154460 -1.132341
H -4.353964 2.481335 2.812536
H -4.101205 0.782863 2.384648
H -2.732186 1.906035 2.389343
H -0.458666 0.646909 -3.234786

preTS_Isomertisation_of_**λ⁵-P**_to_**4**_H_2O_B:
$E_{\omega B97X-D/6-311+G(d,p)}$= -3636.95433366

C 1.010308 2.233257 1.661013
C 0.913433 1.649549 0.404428
C 0.355943 2.475602 -0.564916
C -0.061209 3.773461 -0.341660
C 0.068067 4.312163 0.927824
C 0.606622 3.534321 1.934036
B 1.267944 0.087367 0.054422
C 2.099472 -0.178109 -1.333469
C 2.872731 0.755942 -2.012459
C 3.595780 0.461660 -3.157648
C 3.577582 -0.823496 -3.668485
C 2.843191 -1.796820 -3.015961
C 2.138399 -1.461617 -1.871359
F 2.972520 2.016932 -1.572306
F 4.313229 1.404613 -3.767554
F 4.264136 -1.121114 -4.766606
F 2.828179 -3.044454 -3.484882
F 1.478025 -2.467571 -1.273959
F 0.181448 2.007333 -1.817611
F -0.591244 4.501704 -1.323702
F -0.329252 5.555196 1.173956
F 0.725997 4.033977 3.163040
F 1.499320 1.554793 2.708878
C 2.061354 -0.699612 1.255990
C 3.409002 -0.427377 1.464694
C 4.184554 -1.060368 2.419479
C 3.610875 -2.021863 3.234121
C 2.270476 -2.320781 3.078642
C 1.536943 -1.657134 2.108615
F 4.015311 0.520890 0.735603
F 0.222111 -1.988406 2.050560
F 1.695946 -3.232980 3.860261
F 4.337020 -2.642576 4.157486

F 5.470730 -0.749410 2.567791
O -0.124246 -0.604134 -0.150596
P -1.525941 -0.538297 -1.425483
C -2.372822 -1.990145 -1.169432
C -3.600578 -1.948694 -0.508393
Si -4.481100 -3.580135 -0.071274
C -6.187329 -3.548010 -0.848339
C -2.571423 0.759474 -1.148596
C -3.768440 0.535149 -0.452758
C -4.229961 -0.745728 -0.140393
Si -4.719483 2.088536 0.097182
C -3.623040 2.966232 1.343024
C -4.994082 3.177017 -1.404627
C -6.351936 1.594150 0.870644
C -3.480588 -5.023233 -0.724151
C -4.615034 -3.659337 1.797836
H -1.902577 -2.924523 -1.451433
H -5.172866 -0.820203 0.393241
H -2.254214 1.758359 -1.420119
H -4.077155 3.904246 1.675837
H -3.450793 2.339335 2.222741
H -2.649296 3.200959 0.902670
H -5.510823 4.097782 -1.117369
H -4.047375 3.460118 -1.873930
H -5.603999 2.664755 -2.154104
H -6.903338 2.488798 1.175221
H -6.977330 1.039411 0.165141
H -6.205522 0.974681 1.760154
H -3.986496 -5.962551 -0.481381
H -3.368079 -4.977809 -1.811388
H -2.483088 -5.058506 -0.276299
H -6.736366 -4.462848 -0.605061
H -6.772113 -2.698248 -0.484297
H -6.120530 -3.471210 -1.937323
H -5.129772 -4.573762 2.108286
H -3.624342 -3.653114 2.261450
H -5.177738 -2.806921 2.189427
H -0.446085 -1.059475 0.638916
H -0.589599 -0.440470 -2.452952

TS_Isomertisation_of_**λ⁵-P**_to_**4**_H_2O_B:
$E_{\omega B97X\text{-}D/6\text{-}311+G(d,p)}$= -3636.91196219

C 0.478426 -2.161498 1.784617
C 0.216523 -0.864565 1.357283
C -0.863961 -0.259838 1.995017
C -1.619330 -0.863356 2.983608
C -1.307969 -2.151018 3.381327
C -0.249957 -2.802444 2.776857
B 0.962619 -0.140709 0.088409
O -0.154274 -0.330234 -1.003340
P -0.645812 0.005574 -2.571711
C -1.507413 1.492385 -2.341840
C -2.442898 1.322426 -1.350336
Si -3.228599 2.871577 -0.571177
C -4.217175 3.730269 -1.912930
F -1.231136 0.988697 1.661719
F -2.644167 -0.222359 3.544772
F -2.023886 -2.756554 4.321742
F 0.052455 -4.048601 3.139188
F 1.466204 -2.886712 1.242212
C 2.332449 -0.884926 -0.404431
C 3.506749 -0.690865 0.315017
C 4.722685 -1.259998 -0.020918
C 4.803107 -2.082552 -1.130603
C 3.660836 -2.322543 -1.870638
C 2.463532 -1.733542 -1.494194

F 3.491601 0.058227 1.428316
F 1.401246 -2.060234 -2.256089
F 3.716496 -3.123126 -2.935036
F 5.959722 -2.639555 -1.474983
F 5.808971 -1.031753 0.715742
C 1.274651 1.461895 0.276433
C 1.532052 2.250464 -0.834525
C 1.797681 3.607676 -0.790633
C 1.850407 4.239594 0.437327
C 1.650973 3.492775 1.584631
C 1.381692 2.136682 1.487447
F 1.548376 1.687471 -2.062014
F 2.005675 4.300452 -1.910076
F 2.098090 5.542639 0.515464
F 1.722052 4.084577 2.776418
F 1.243344 1.490699 2.652111
C -1.831292 -1.251582 -2.742146
C -2.734761 -1.161116 -1.714554
C -2.738570 -0.002495 -0.838998
Si -3.854561 -2.618873 -1.242068
C -3.635853 -3.993589 -2.492885
C -3.279405 -3.150623 0.463268
C -5.627639 -2.016377 -1.187742
C -4.333006 2.359587 0.850679
C -1.826900 3.967549 0.019798
H -1.739101 -2.075429 -3.435088
H -3.422090 -0.043634 0.002102
H -1.216130 2.434703 -2.784457
H -2.212016 4.878617 0.487316
H -1.209772 3.440947 0.752392
H -1.184912 4.262254 -0.815460
H -4.672631 4.646727 -1.525306
H -3.579818 4.002702 -2.759090
H -5.017194 3.083832 -2.284620
H -4.781028 3.245956 1.309924
H -5.146203 1.711538 0.510954
H -3.769015 1.830027 1.622729
H -4.277303 -4.841308 -2.234347
H -3.907023 -3.664554 -3.500281
H -2.602187 -4.350457 -2.514583
H -6.298522 -2.820948 -0.871741
H -5.738238 -1.188789 -0.480890
H -5.954693 -1.668414 -2.171658
H -3.832798 -4.029357 0.807250
H -2.214094 -3.400088 0.452849
H -3.433524 -2.350454 1.192763
H -1.260985 -0.259796 -0.568264
H 0.472609 -0.025186 -3.397681

postTS_Isomertisation_of_**λ⁵-P**_to_**4**_H_2O_B:
$E_{\omega B97X\text{-}D/6\text{-}311+G(d,p)}$= -3636.99773573

C -0.739295 -1.298770 1.203861
C 0.480460 -1.476150 0.561788
C 0.962932 -2.778514 0.610587
C 0.325199 -3.810819 1.284696
C -0.865102 -3.565902 1.942317
C -1.404484 -2.292652 1.897612
B 1.200417 -0.265353 -0.290870
O 0.508769 -0.247985 -1.643110
P -0.895160 0.019782 -2.237225
C -1.592746 1.565563 -1.739131
C -2.746643 1.642367 -1.060225
Si -3.255692 3.304892 -0.275222
C -2.834135 3.142792 1.543739
F 2.099171 -3.120634 -0.015478

F 0.847952 -5.037516 1.298345
F -1.490101 -4.540351 2.596559
F -2.560519 -2.038509 2.514466
F -1.353927 -0.103364 1.167008
C 1.120064 1.234027 0.406429
C 0.951469 1.504279 1.760299
C 0.939540 2.785130 2.293007
C 1.121510 3.876598 1.464734
C 1.331607 3.661549 0.115284
C 1.340208 2.365385 -0.373471
F 0.786997 0.517812 2.651741
F 1.583678 2.245880 -1.690592
F 1.533409 4.698986 -0.699838
F 1.103584 5.111173 1.958023
F 0.752170 2.972940 3.600017
C 2.799927 -0.519456 -0.577858
C 3.675172 -0.596715 0.499443
C 5.044326 -0.750242 0.382995
C 5.607868 -0.831630 -0.878037
C 4.783330 -0.760962 -1.983733
C 3.411700 -0.608143 -1.822392
F 3.188231 -0.541339 1.751062
F 5.821146 -0.822958 1.465449
F 6.923261 -0.977712 -1.022410
F 5.313887 -0.841105 -3.206692
F 2.712197 -0.564389 -2.965341
C -2.101456 -1.214610 -1.844660
C -3.258483 -0.919238 -1.233317
C -3.644221 0.466351 -0.796465
Si -4.538837 -2.309227 -0.945613
C -5.475839 -1.938178 0.632106
C -5.700580 -2.279859 -2.416742
C -3.629602 -3.942660 -0.840997
C -2.272584 4.686355 -1.065910
C -5.096739 3.541803 -0.526947
H -1.847056 -2.231614 -2.129741
H -4.624847 0.700721 -1.235136
H -0.986989 2.444263 -1.935560
H -2.586436 5.645866 -0.644283
H -1.200619 4.582888 -0.878180
H -2.429635 4.726894 -2.147348
H -5.419652 4.488897 -0.084668
H -5.353101 3.561566 -1.590151
H -5.670524 2.740994 -0.051344
H -3.052158 4.074323 2.074357
H -3.404859 2.340924 2.020197
H -1.771112 2.919293 1.668265
H -4.330593 -4.740443 -0.578598
H -3.165985 -4.211664 -1.794242
H -2.847496 -3.917615 -0.077597
H -6.468496 -3.051868 -2.308345
H -6.206930 -1.314274 -2.505576
H -5.158378 -2.468187 -3.347564
H -6.204291 -2.731304 0.825475
H -4.806193 -1.877974 1.493099
H -6.025681 -0.995462 0.556497
H -3.853525 0.426314 0.281408
H -0.657500 0.005650 -3.616619

vdW_insertion_$(H_2O)_2$_B:
$E_{\omega B97X-D/6-311+G(d,p)}$= -3713.44609046

C -3.367171 -1.749312 -0.955456
C -3.080383 -0.907040 0.111814
C -4.183387 -0.549154 0.881333
C -5.469057 -1.005445 0.645429

C -5.699930 -1.855336 -0.421563
C -4.640707 -2.224919 -1.229892
B -1.610209 -0.271073 0.456506
C -1.691083 1.347845 0.239893
C -1.434815 2.319604 1.196156
C -1.600573 3.678519 0.968201
C -2.033100 4.118107 -0.267300
C -2.294051 3.187512 -1.258914
C -2.122162 1.843086 -0.985922
F -0.987706 1.993431 2.421596
F -1.328106 4.562795 1.928134
F -2.181407 5.417014 -0.507399
F -2.699906 3.594797 -2.460988
F -2.378002 0.990306 -1.990339
F -4.033323 0.285528 1.922074
F -6.480249 -0.632764 1.429494
F -6.924870 -2.305943 -0.669522
F -4.852792 -3.032817 -2.268046
F -2.409061 -2.140432 -1.805616
O -1.423137 -0.535823 1.996066
C -0.351607 -1.015200 -0.279685
C 0.570032 -0.430004 -1.138547
C 1.621863 -1.126400 -1.717000
C 1.792843 -2.467374 -1.436653
C 0.913304 -3.094691 -0.572448
C -0.124588 -2.364206 -0.028245
F 0.490800 0.864976 -1.470006
F 2.465515 -0.514707 -2.545971
F 2.790091 -3.149630 -1.991624
F 1.067508 -4.387690 -0.288032
F -0.960870 -3.039248 0.784398
P 1.809776 -0.035803 2.358205
C 2.093602 1.376686 1.407151
C 3.100422 1.526336 0.454508
Si 3.238434 3.141885 -0.537163
C 3.627670 2.729582 -2.324492
C 3.056616 -1.091095 1.796688
C 4.026940 -0.760590 0.848696
Si 5.423708 -1.987289 0.450371
C 6.873333 -1.579377 1.571247
C 4.014189 0.491023 0.217573
C 1.602232 4.051909 -0.434716
C 4.621606 4.170866 0.205165
C 5.930571 -1.788964 -1.344803
C 4.831876 -3.735006 0.787598
H -0.511583 -0.434772 2.332978
H 4.778474 0.670556 -0.535377
H 1.406304 2.201621 1.572487
H 3.079571 -2.080978 2.246495
H 6.693626 -2.531395 -1.598453
H 5.082764 -1.931028 -2.019030
H 6.357384 -0.800230 -1.536609
H 7.713680 -2.254103 1.380403
H 6.592288 -1.676295 2.624081
H 7.217671 -0.554046 1.406324
H 5.618168 -4.447257 0.519033
H 4.600611 -3.886124 1.845969
H 3.942627 -3.984491 0.203813
H 3.719745 3.650675 -2.908452
H 4.567831 2.179164 -2.422216
H 2.833014 2.122480 -2.764625
H 1.626903 4.942870 -1.069177
H 0.787646 3.410911 -0.783030
H 1.375235 4.378729 0.584172
H 4.744294 5.110818 -0.341777
H 4.412432 4.410882 1.251740
H 5.572256 3.630288 0.165849

H -1.864295 -1.343167 2.398613
O -2.604413 -2.532162 3.108942
H -2.785598 -3.301982 2.563280
H -3.411750 -2.343503 3.594636

TS_insertion_(H_2O)$_2$_B:
$E_{\omega B97X\text{-}D/6\text{-}311+G(d,p)}$= -3713.40546926

C -3.307228 -1.715480 -0.907048
C -2.979042 -0.878960 0.151409
C -4.064935 -0.507838 0.939579
C -5.362028 -0.946980 0.730399
C -5.629383 -1.797495 -0.327185
C -4.591337 -2.180035 -1.154942
B -1.472930 -0.277357 0.537794
C -1.578152 1.351355 0.239825
C -1.394903 2.358227 1.176710
C -1.598975 3.706221 0.908119
C -2.004933 4.102581 -0.350109
C -2.199698 3.137813 -1.323552
C -1.987805 1.808086 -1.008334
F -0.976662 2.094871 2.430363
F -1.388258 4.625801 1.855071
F -2.190488 5.391845 -0.628415
F -2.582644 3.501128 -2.549841
F -2.190586 0.927043 -2.003355
F -3.897733 0.332671 1.974485
F -6.355753 -0.555948 1.533462
F -6.868359 -2.234008 -0.547975
F -4.834724 -2.990343 -2.188578
F -2.375717 -2.126135 -1.784585
O -1.180820 -0.519502 1.952769
C -0.259177 -1.016990 -0.343575
C 0.658808 -0.435411 -1.205030
C 1.676826 -1.136599 -1.840717
C 1.816322 -2.490922 -1.619542
C 0.939931 -3.120235 -0.752353
C -0.055910 -2.379374 -0.145526
F 0.619802 0.879930 -1.488058
F 2.523920 -0.512606 -2.663413
F 2.777647 -3.184417 -2.230246
F 1.057034 -4.432080 -0.529365
F -0.885157 -3.072118 0.658784
P 1.696766 0.145074 2.325766
C 1.955032 1.543577 1.421747
C 2.996464 1.564420 0.492760
Si 3.255480 3.149948 -0.547568
C 3.726597 2.645870 -2.285563
C 2.806281 -1.061570 1.927900
C 3.786948 -0.779075 0.971889
Si 5.144660 -2.084293 0.628017
C 6.504018 -1.762994 1.876405
C 3.838383 0.457585 0.308778
C 1.640537 4.096406 -0.545982
C 4.629001 4.127006 0.269343
C 5.775396 -1.844344 -1.117413
C 4.420811 -3.792639 0.872798
H 0.598493 -0.032503 3.162683
H 4.616762 0.566988 -0.440759
H 1.303994 2.394131 1.573093
H 2.745283 -2.021321 2.427290
H 6.482026 -2.644010 -1.359427
H 4.968037 -1.877827 -1.852400
H 6.305141 -0.894151 -1.229660
H 7.327416 -2.469616 1.734700
H 6.133018 -1.875596 2.899171

H 6.903336 -0.750529 1.767890
H 5.199092 -4.540544 0.692904
H 4.056953 -3.942519 1.893171
H 3.599990 -3.997501 0.182565
H 3.875042 3.541402 -2.896727
H 4.655453 2.069596 -2.312653
H 2.938354 2.044858 -2.743870
H 1.713044 4.954002 -1.221126
H 0.821558 3.461215 -0.894710
H 1.385257 4.482520 0.445101
H 4.818192 5.053705 -0.280836
H 4.366122 4.390275 1.297792
H 5.559009 3.551340 0.291626
H -1.638805 -1.298830 2.291022
O -2.675875 -2.757120 2.995949
H -2.876907 -3.283094 2.217913
H -3.529102 -2.501459 3.354027

λ⁵-P_insertion_$(H_2O)_2$_B:
$E_{\omega B97X\text{-}D/6\text{-}311+G(d,p)}$= -3713.42132950

C 2.873215 -1.944338 0.881231
C 2.511464 -1.116559 -0.176586
C 3.501013 -0.960249 -1.142949
C 4.732561 -1.590394 -1.104227
C 5.036036 -2.417197 -0.037717
C 4.098893 -2.589431 0.963376
B 1.118730 -0.239423 -0.275877
C 1.583034 1.312321 -0.023245
C 1.719804 2.304733 -0.984367
C 2.185999 3.581929 -0.704065
C 2.564342 3.908910 0.582640
C 2.486875 2.941398 1.569941
C 2.020731 1.681375 1.244945
F 1.403570 2.088658 -2.273428
F 2.266849 4.497466 -1.670971
F 3.004778 5.130617 0.868406
F 2.866600 3.230225 2.814480
F 2.004953 0.777135 2.237062
F 3.287616 -0.152855 -2.196083
F 5.625265 -1.403137 -2.076561
F 6.212513 -3.031927 0.025466
F 4.380532 -3.374600 2.002770
F 2.043367 -2.165886 1.909200
O 0.582266 -0.406595 -1.733689
C -0.086727 -0.809930 0.677157
C -0.836814 -0.105936 1.606612
C -1.802900 -0.692774 2.413589
C -2.063567 -2.042437 2.299032
C -1.372895 -2.782543 1.354360
C -0.430484 -2.154030 0.566656
F -0.662421 1.209711 1.790813
F -2.481668 0.039357 3.297564
F -2.968700 -2.626508 3.080223
F -1.609949 -4.090261 1.231232
F 0.199700 -2.930129 -0.335035
P -1.034804 0.115194 -2.379557
C -1.479210 1.594255 -1.670494
C -2.526051 1.634493 -0.748150
Si -2.866022 3.307234 0.092173
C -3.714488 3.019378 1.737592
C -2.293483 -0.993287 -2.090101
C -3.281831 -0.697721 -1.147533
Si -4.715721 -1.929089 -0.938533
C -5.972170 -1.522288 -2.273637
C -3.348535 0.532999 -0.478118

C -1.233582 4.201718 0.337724
C -3.977182 4.304111 -1.046573
C -5.502881 -1.715512 0.749691
C -4.099033 -3.683958 -1.173768
H -0.508041 0.115290 -3.674110
H -4.135541 0.663825 0.257262
H -0.896990 2.475497 -1.900364
H -2.271855 -1.943218 -2.612101
H -6.309169 -2.445587 0.871309
H -4.789891 -1.869559 1.562298
H -5.940404 -0.719917 0.865470
H -6.833841 -2.194524 -2.214117
H -5.530394 -1.623952 -3.269293
H -6.333920 -0.495365 -2.167259
H -4.933822 -4.383367 -1.064555
H -3.677532 -3.831324 -2.172488
H -3.337271 -3.955133 -0.439880
H -3.881170 3.977759 2.239149
H -4.688709 2.537942 1.612754
H -3.105132 2.393412 2.393310
H -1.391954 5.123946 0.905033
H -0.525119 3.578398 0.889293
H -0.775909 4.479166 -0.616457
H -4.189915 5.288397 -0.617868
H -3.502625 4.454354 -2.020833
H -4.929754 3.791311 -1.209323
H 0.980672 -1.151010 -2.265624
O 1.415228 -2.405593 -3.190347
H 1.502006 -3.243507 -2.728010
H 2.189282 -2.325156 -3.753844

TS_Isomertisation_of_**λ⁵-P**_to_**3**_(H_2O)$_2$_B:
$E_{\omega B97X\text{-}D/6\text{-}311+G(d,p)}$= -3713.39200184

C -2.359765 2.195264 -1.467520
C -1.630150 1.713080 -0.389576
C -1.048745 2.705191 0.395447
C -1.166822 4.060471 0.156382
C -1.911852 4.488986 -0.930462
C -2.510743 3.548996 -1.745589
B -1.311226 0.133965 -0.041568
O 0.218299 0.050126 -0.145023
P 1.447722 -0.078087 0.909721
C 2.586596 1.197714 0.614399
C 3.934110 0.916050 0.233798
Si 5.094763 2.407409 0.047027
C 4.294404 3.623898 -1.141159
F -0.321593 2.356071 1.474563
F -0.582638 4.952984 0.955464
F -2.043341 5.786571 -1.185351
F -3.223693 3.946414 -2.799249
F -2.970631 1.366054 -2.326164
C -1.949243 -0.916740 -1.133560
C -3.298788 -1.246843 -1.071632
C -3.910148 -2.169025 -1.901187
C -3.158416 -2.810154 -2.870481
C -1.816996 -2.502774 -2.989480
C -1.245390 -1.569265 -2.135883
F -4.096905 -0.629692 -0.183676
F 0.056469 -1.314376 -2.361501
F -1.082245 -3.099992 -3.929841
F -3.722543 -3.700664 -3.682271
F -5.211285 -2.437651 -1.785181
C -1.811431 -0.355181 1.450357
C -1.467543 -1.627605 1.894007
C -1.869252 -2.167242 3.103126

C -2.679880 -1.418154 3.937146
C -3.068873 -0.153064 3.537106
C -2.638482 0.347275 2.317168
F -0.684369 -2.409254 1.127335
F -1.487591 -3.391127 3.467206
F -3.083018 -1.909784 5.104334
F -3.862677 0.572456 4.324782
F -3.099197 1.565903 2.000785
C 2.340517 -1.550908 0.686131
C 3.631842 -1.547606 0.212793
C 4.392110 -0.361091 -0.012146
Si 4.488297 -3.207745 -0.147260
C 6.054693 -3.293189 0.882884
C 3.338880 -4.624312 0.281598
C 4.912035 -3.235468 -1.975331
C 6.756562 1.848903 -0.613178
C 5.285533 3.203953 1.734812
O 1.526632 1.314551 -1.895006
H 2.112688 1.449589 -1.025852
H 0.794891 0.749801 -1.471934
H 5.729365 2.503864 2.448386
H 2.322578 2.183040 0.989259
H 5.414609 -0.482289 -0.355973
H 1.819824 -2.478948 0.890854
H 6.593909 -4.225011 0.686368
H 6.726195 -2.461071 0.652186
H 5.822739 -3.254034 1.951111
H 3.830642 -5.577892 0.066607
H 3.070281 -4.619635 1.342064
H 2.416615 -4.584843 -0.305351
H 5.426496 -4.164821 -2.238519
H 4.007324 -3.163074 -2.585931
H 5.567159 -2.400452 -2.240413
H 7.416908 2.713526 -0.730686
H 7.244263 1.146678 0.068882
H 6.658956 1.366103 -1.589780
H 5.933030 4.084089 1.674459
H 4.318563 3.525042 2.133102
H 4.928241 4.506597 -1.268911
H 4.145083 3.175040 -2.128029
H 3.325269 3.968001 -0.766240
H 0.754146 -0.018390 2.128704
H 1.166964 2.155655 -2.209782

λ⁵-P_from_TS_isomerisation_**3**_$(H_2O)_2$_B:
$E_{\omega B97X\text{-}D/6\text{-}311+G(d,p)}$= -3713.41191204

C -0.308625 2.526486 0.384366
C -0.864524 1.630564 -0.522645
C -0.879888 2.084492 -1.834891
C -0.408700 3.333641 -2.219209
C 0.120471 4.188642 -1.271968
C 0.173853 3.777281 0.049561
B -1.268938 0.114929 -0.041788
O 0.127706 -0.579530 0.145131
P 1.380283 -0.606379 1.444462
C 2.463582 0.692540 1.300069
C 3.684469 0.497557 0.643839
Si 4.646872 2.065660 0.163882
C 6.425302 1.629994 -0.232363
F -1.342164 1.314076 -2.830800
F -0.451543 3.707699 -3.497067
F 0.583365 5.382593 -1.623719
F 0.695610 4.579993 0.975753
F -0.200850 2.180380 1.681451
C -2.095247 -0.008877 1.367474

C -2.876702 0.993865 1.931042
C -3.645142 0.811904 3.070804
C -3.663880 -0.420291 3.698101
C -2.918428 -1.456934 3.166294
C -2.171066 -1.235013 2.022079
F -2.936697 2.213959 1.383571
F -1.492074 -2.292811 1.546834
F -2.932361 -2.654568 3.749856
F -4.392520 -0.607741 4.793019
F -4.368087 1.815311 3.566257
C -2.125849 -0.753306 -1.135297
C -3.386750 -0.291227 -1.497351
C -4.241538 -0.959956 -2.352478
C -3.844255 -2.172259 -2.891020
C -2.603758 -2.679828 -2.558211
C -1.775395 -1.974629 -1.694526
F -3.821506 0.882195 -1.010077
F -5.434704 -0.453384 -2.663174
F -4.647178 -2.837638 -3.716353
F -2.209248 -3.847813 -3.067130
F -0.593617 -2.553491 -1.434983
C 2.255701 -2.048313 1.188774
C 3.506897 -1.985046 0.580353
C 4.153642 -0.769806 0.277487
Si 4.398250 -3.600015 0.109839
C 3.366093 -5.066509 0.650508
C 4.610126 -3.591122 -1.754414
C 6.071343 -3.613077 0.956638
C 4.560478 3.294430 1.576922
C 3.776753 2.764267 -1.348143
O 1.718839 -0.567022 -1.970284
H 2.618449 -0.670520 -1.632924
H 0.634507 -0.611774 -0.714815
H 4.972901 2.870418 2.496730
H 2.141656 1.676808 1.613106
H 5.120603 -0.825779 -0.213979
H 1.773135 -2.992810 1.408993
H 6.627643 -4.518259 0.694517
H 6.672554 -2.749849 0.656588
H 5.959599 -3.585873 2.044301
H 3.876557 -5.996557 0.382943
H 3.209673 -5.072484 1.733205
H 2.387401 -5.071667 0.161771
H 5.129134 -4.494934 -2.087942
H 3.639328 -3.552317 -2.257210
H 5.197180 -2.727497 -2.081082
H 6.984913 2.537734 -0.477592
H 6.916115 1.151329 0.619886
H 6.496178 0.955531 -1.090490
H 5.138680 4.189263 1.327063
H 3.533792 3.613046 1.777928
H 4.181391 3.743180 -1.621585
H 3.891466 2.099677 -2.209869
H 2.706784 2.883364 -1.150277
H 0.427998 -0.550614 2.462070
H 1.737139 0.219376 -2.521915

TS_Isomertisation_of_**λ⁵-P**_to_**4**_$(H_2O)_2$_B:
$E_{\omega B97X\text{-}D/6\text{-}311+G(d,p)}$= -3713.40094416

C -0.641035 2.087153 -1.759899
C -0.764347 1.638515 -0.452031
C -0.337365 2.552298 0.504907
C 0.146287 3.814459 0.217891
C 0.233886 4.219160 -1.103538
C -0.161830 3.346434 -2.098356

B -1.171476 0.103236 -0.014288
C -1.915983 -0.753399 -1.207199
C -1.469369 -1.927448 -1.795157
C -2.195060 -2.637729 -2.741863
C -3.434059 -2.178128 -3.142394
C -3.925109 -1.007783 -2.588958
C -3.166705 -0.334594 -1.649566
F -0.264106 -2.454715 -1.494789
F -1.701654 -3.759270 -3.269707
F -4.142712 -2.845669 -4.048614
F -5.115267 -0.542895 -2.968412
F -3.691251 0.801776 -1.163738
F -0.361547 2.213461 1.808698
F 0.535230 4.637403 1.192537
F 0.703615 5.424329 -1.408705
F -0.067409 3.713676 -3.376322
F -0.968771 1.301326 -2.798095
O 0.172569 -0.566676 0.274821
P 1.177950 -0.571414 1.541442
C 2.138799 -2.013907 1.429801
C 3.316896 -1.961483 0.721506
Si 4.302189 -3.557135 0.402285
C 3.410387 -5.012212 1.173102
C 2.310862 0.736114 1.428255
C 3.449465 0.545169 0.675157
Si 4.414468 2.088824 0.121528
C 3.730115 2.542210 -1.567588
C 3.846209 -0.739865 0.184698
C 4.107803 3.480495 1.337658
C 6.242839 1.690498 0.015553
C 6.003819 -3.339902 1.159987
C 4.442331 -3.779957 -1.456417
C -2.168675 -0.032753 1.290844
C -2.296473 -1.264005 1.927020
C -3.156726 -1.505018 2.985230
C -3.966626 -0.482470 3.444317
C -3.898270 0.754736 2.830647
C -3.018016 0.954273 1.778040
F -1.561388 -2.313533 1.518078
F -3.216948 -2.708730 3.555570
F -4.803730 -0.687944 4.456522
F -4.682968 1.745565 3.254897
F -3.039861 2.178779 1.235271
O 1.912757 -0.625109 -1.672306
H 2.808954 -0.685843 -1.033449
H 1.134260 -0.631362 -1.027809
H 0.333919 -0.508778 2.656746
H 2.037041 1.708935 1.814088
H 4.819236 -0.798227 -0.296493
H 1.738125 -2.941012 1.821504
H 3.973674 -5.932012 0.988903
H 3.310265 -4.894389 2.255963
H 2.410763 -5.141876 0.748254
H 5.063351 -4.650148 -1.689932
H 3.458867 -3.940265 -1.908229
H 4.900174 -2.906619 -1.930236
H 6.614535 -4.233117 0.996667
H 6.528076 -2.487713 0.717917
H 5.932007 -3.170205 2.238118
H 6.807024 2.584868 -0.265814
H 6.625943 1.337481 0.977298
H 6.445988 0.921195 -0.734839
H 4.707045 4.351727 1.056097
H 3.060323 3.793305 1.346938
H 4.388157 3.193112 2.354917
H 4.172355 3.473991 -1.932762
H 3.934003 1.758697 -2.303064

H 2.645631 2.678559 -1.513214
H 1.847202 -1.411853 -2.229889

λ[5]-P_from_TS_isomerisation_**4**_(H_2O)$_2$_B:
$E_{\omega B97X\text{-}D/6\text{-}311+G(d,p)}$= -3713.41297056

C -3.389702 -0.320857 -1.466987
C -2.091600 -0.719236 -1.164782
C -1.678672 -1.872222 -1.815970
C -2.480500 -2.577646 -2.703087
C -3.761975 -2.137132 -2.969529
C -4.222367 -0.991316 -2.342762
B -1.263518 0.136877 -0.038094
C -0.921769 1.682067 -0.463379
C -0.982303 2.188684 -1.754563
C -0.567367 3.470073 -2.093664
C -0.051633 4.304392 -1.121173
C 0.046185 3.840768 0.180383
C -0.378604 2.558309 0.469618
F -1.440511 1.445331 -2.771665
F -0.651924 3.896336 -3.353142
F 0.356645 5.530054 -1.430183
F 0.556764 4.624437 1.129853
F -0.224683 2.158525 1.747596
F -0.445879 -2.379069 -1.633000
F -2.020622 -3.678207 -3.299632
F -4.542326 -2.801496 -3.816601
F -5.454273 -0.548066 -2.590872
F -3.886128 0.789553 -0.898881
C -2.077044 -0.078122 1.368326
C -2.878454 0.872560 1.990376
C -3.627212 0.611970 3.128037
C -3.604097 -0.650300 3.692211
C -2.836677 -1.637542 3.100802
C -2.109599 -1.337194 1.961532
F -2.980382 2.115573 1.504653
F -1.405064 -2.349734 1.427638
F -2.810076 -2.862822 3.623530
F -4.313745 -0.913022 4.784139
F -4.372048 1.568168 3.681087
O 0.155821 -0.511940 0.128524
P 1.402128 -0.542163 1.426870
C 2.264079 -1.997518 1.198913
C 3.506188 -1.959056 0.572164
Si 4.357210 -3.592086 0.093884
C 6.099698 -3.581084 0.785892
C 2.496064 0.743041 1.248025
C 3.712030 0.521945 0.588692
Si 4.703006 2.075555 0.116599
C 4.901029 3.144174 1.644257
C 4.163403 -0.756404 0.240213
C 4.396317 -3.654059 -1.781632
C 3.378869 -5.035946 0.777621
C 6.372534 1.579592 -0.573449
C 3.684534 2.977961 -1.177238
O 1.712751 -0.424050 -1.982470
H 2.622341 -0.426924 -1.656034
H 0.668732 -0.515941 -0.732090
H 0.454623 -0.463206 2.447548
H 2.184027 1.738307 1.537651
H 5.123269 -0.831218 -0.262261
H 1.775842 -2.932326 1.446639
H 3.877397 -5.975233 0.519859
H 3.299633 -4.988817 1.867671
H 2.367984 -5.072057 0.360995
H 4.908782 -4.555933 -2.130140

H 3.381320 -3.663405 -2.189971
H 4.922529 -2.787603 -2.193250
H 6.625645 -4.501118 0.513155
H 6.676496 -2.736837 0.397334
H 6.086577 -3.508024 1.877095
H 6.949453 2.473974 -0.827427
H 6.950770 1.002621 0.154019
H 6.270938 0.980634 -1.483159
H 5.449869 4.058442 1.398707
H 3.931488 3.439714 2.055620
H 5.454175 2.615100 2.425378
H 4.154390 3.924372 -1.460767
H 3.567077 2.371634 -2.080147
H 2.684703 3.198895 -0.791945
H 1.646157 -1.177129 -2.576004

PRO_Isomertisation_of_**λ⁵-P**_to_**3**_$(H_2O)_2$_B:
$E_{\omega B97X-D/6-311+G(d,p)}$= -3713.44497026

C 1.157475 2.272167 1.574042
C 1.102305 1.678990 0.322340
C 0.892525 2.572371 -0.721495
C 0.795485 3.941534 -0.569535
C 0.881286 4.484890 0.701006
C 1.056918 3.641457 1.780336
B 1.116202 0.068917 0.000751
O -0.339241 -0.204440 -0.388043
P -1.206241 -0.410990 -1.656208
C -2.348836 0.952930 -1.858994
C -3.435957 0.885332 -0.804094
Si -4.082289 2.524611 -0.108352
C -4.973864 3.426945 -1.491101
F 0.761134 2.103542 -1.980645
F 0.596568 4.736457 -1.621308
F 0.771472 5.797155 0.879728
F 1.112528 4.145827 3.012285
F 1.291356 1.531976 2.689127
C 1.488170 -0.894741 1.274605
C 0.700337 -1.909592 1.802042
C 1.119204 -2.733681 2.839648
C 2.376384 -2.568513 3.386520
C 3.203829 -1.577719 2.884422
C 2.747896 -0.780318 1.852558
F -0.530857 -2.164797 1.335009
F 3.590940 0.165957 1.407228
F 4.421333 -1.404386 3.399300
F 2.789592 -3.351230 4.379537
F 0.315514 -3.690479 3.306960
C 2.134538 -0.390914 -1.210571
C 3.228286 0.330073 -1.676005
C 4.106535 -0.150313 -2.635866
C 3.915904 -1.409633 -3.175008
C 2.849397 -2.173822 -2.737279
C 2.002959 -1.656202 -1.772312
F 3.504080 1.552312 -1.202940
F 5.135408 0.591547 -3.043586
F 4.747643 -1.880226 -4.098235
F 2.651003 -3.392271 -3.238554
F 0.994627 -2.460636 -1.378616
C -2.257735 -1.819452 -1.456789
C -3.440601 -1.634924 -0.833621
C -3.896641 -0.305705 -0.381737
Si -4.553129 -3.125732 -0.414323
C -4.627034 -3.227024 1.455350
C -6.246769 -2.777663 -1.136627
C -3.800037 -4.672627 -1.148561

C -2.583856 3.509359 0.441706
C -5.229652 2.171101 1.326232
O -1.684990 0.610959 2.267454
H -1.797174 1.894050 -1.847649
H -1.432874 0.239418 1.414877
H -5.845713 2.862201 -1.833318
H -2.786092 0.844450 -2.861173
H -4.692304 -0.312664 0.358187
H -1.885165 -2.795974 -1.742510
H -6.937456 -3.591704 -0.897197
H -6.667273 -1.852041 -0.732962
H -6.198471 -2.683278 -2.225139
H -4.441872 -5.532545 -0.935348
H -3.697459 -4.594476 -2.234782
H -2.814449 -4.880736 -0.722326
H -5.260988 -4.064041 1.763417
H -3.629914 -3.381857 1.876439
H -5.041682 -2.314553 1.892967
H -5.593765 3.112849 1.747726
H -6.100970 1.586537 1.017165
H -4.707817 1.626451 2.117779
H -5.317066 4.408217 -1.149270
H -4.312723 3.586053 -2.348520
H -2.894320 4.387786 1.015410
H -1.933088 2.897362 1.073376
H -2.009536 3.867284 -0.417318
H -0.399062 -0.531310 -2.796063
H -0.882200 0.573764 2.792864

PRO_Isomertisation_of_**λ⁵-P**_to_**4**_$(H_2O)_2$_B:
$E_{\omega B97X\text{-}D/6\text{-}311+G(d,p)}$= -3713.44008011
C 2.996266 1.271806 -1.660291
C 2.183372 0.202057 -1.303305
C 2.322428 -0.919822 -2.114039
C 3.160905 -0.983918 -3.214345
C 3.935738 0.115099 -3.536809
C 3.854374 1.249041 -2.749099
B 1.213947 0.125202 0.025579
O -0.129272 -0.511444 -0.338170
P -1.152992 -0.517352 -1.515416
C -2.313969 0.806546 -1.387701
C -3.489920 0.613278 -0.768679
C -3.969932 -0.735341 -0.312229
C -3.299480 -1.990399 -0.802139
Si -4.225987 -3.625702 -0.474614
C -4.576586 -3.678799 1.365528
F 1.620337 -2.036730 -1.844486
F 3.233877 -2.090613 -3.954208
F 4.751849 0.080153 -4.585616
F 4.605386 2.310550 -3.042671
F 3.001499 2.404792 -0.945960
C 0.779183 1.566796 0.684447
C 0.694994 1.833319 2.044239
C 0.173285 3.012001 2.561935
C -0.301364 3.987293 1.706629
C -0.246737 3.767069 0.340650
C 0.279281 2.578219 -0.127218
F 1.112184 0.943056 2.955299
F 0.275741 2.417640 -1.466990
F -0.703098 4.694932 -0.502401
F -0.806771 5.119700 2.185688
F 0.118344 3.205876 3.879383
C 1.970758 -0.894489 1.066302
C 1.521842 -2.139754 1.483924
C 2.257463 -2.974086 2.315868
C 3.501224 -2.576885 2.765816

C 3.992566 -1.342395 2.376843
C 3.227312 -0.544195 1.547750
F 0.322163 -2.621386 1.115951
F 1.769309 -4.160533 2.684281
F 4.215976 -3.366876 3.563061
F 5.188455 -0.937761 2.806211
F 3.749095 0.649214 1.218774
C -2.092839 -2.004949 -1.390405
C -3.144416 -5.057730 -1.001877
C -5.822463 -3.585424 -1.456447
Si -4.535235 2.138644 -0.291971
C -6.350471 1.698484 -0.434740
C -4.063350 2.522061 1.480945
C -4.093366 3.560275 -1.425859
O -1.820962 -0.595489 2.106305
H -3.885930 -0.746156 0.786317
H -1.188042 -0.635017 1.378975
H -0.442436 -0.442485 -2.722250
H -1.992073 1.785902 -1.720925
H -5.045379 -0.806884 -0.509609
H -1.609279 -2.922898 -1.707455
H -3.658926 -6.000951 -0.795354
H -2.924836 -5.027931 -2.072968
H -2.197176 -5.067506 -0.455162
H -5.095633 -4.605688 1.627098
H -3.648157 -3.635462 1.941939
H -5.209436 -2.842242 1.675608
H -6.385611 -4.509707 -1.295362
H -6.461655 -2.750792 -1.154417
H -5.623753 -3.493386 -2.527884
H -6.966819 2.572678 -0.205057
H -6.601639 1.366679 -1.446469
H -6.628105 0.904988 0.264986
H -4.736699 4.416224 -1.200340
H -3.058717 3.886780 -1.293278
H -4.241186 3.296645 -2.476838
H -4.582707 3.417125 1.835945
H -4.315598 1.693393 2.148346
H -2.986041 2.696873 1.558777
H -1.635735 -1.380726 2.624829

3.5.4 1,2- and 1,4-additions

With borane:

vdW_add_1,2_H_2O_B:
$E_{\omega B97X\text{-}D/6\text{-}311+G(d,p)}$= -3636.98561464

C -2.567106 0.023792 2.169733
C -2.440943 -0.037005 0.792729
C -3.620177 -0.323191 0.113784
C -4.836525 -0.520712 0.739532
C -4.909399 -0.441797 2.121138
C -3.763214 -0.168630 2.843487
B -1.075997 0.112481 -0.078711
O 0.138874 0.328246 0.955947
F -3.606483 -0.394022 -1.224769
F -5.933429 -0.779574 0.032266
F -6.067152 -0.629549 2.743334
F -3.813023 -0.097903 4.171276
F -1.487204 0.267908 2.947579
C -0.950429 1.454321 -0.988907
C 0.221766 1.675774 -1.701806
C 0.470295 2.797969 -2.466544
C -0.492333 3.792271 -2.524675

C -1.669694 3.634270 -1.818315
C -1.877135 2.484611 -1.067058
F 1.206084 0.756738 -1.641168
F -3.037652 2.429592 -0.402977
F -2.595486 4.589785 -1.857075
F -0.281495 4.887058 -3.244124
F 1.613241 2.935339 -3.134669
C -0.792815 -1.306792 -0.832169
C -0.868952 -1.529319 -2.200955
C -0.673989 -2.775444 -2.777592
C -0.397610 -3.867610 -1.974423
C -0.331496 -3.699992 -0.601897
C -0.529781 -2.438881 -0.068731
F -1.143895 -0.528608 -3.044989
F -0.744609 -2.928591 -4.097660
F -0.201829 -5.064422 -2.513102
F -0.089389 -4.745580 0.186181
F -0.471048 -2.338120 1.272021
C 2.553562 0.869278 3.053723
C 3.005833 1.204423 1.771393
C 3.337202 0.201185 0.850910
C 3.237910 -1.184061 1.076001
Si 3.819236 -2.370194 -0.295484
C 2.933652 -1.942073 -1.890827
P 2.322699 -0.727143 3.680806
C 2.774789 -1.673218 2.297762
C 5.665758 -2.115846 -0.506995
C 3.452295 -4.134202 0.215183
Si 3.104249 3.044212 1.294011
C 1.354281 3.724604 1.285505
C 4.133589 3.932049 2.584864
C 3.887631 3.203802 -0.400257
H 1.039103 0.174028 0.619975
H 0.064294 -0.049121 1.844400
H 2.312933 1.678354 3.741554
H 3.701733 0.520737 -0.122820
H 2.698011 -2.750455 2.425425
H 3.184722 -2.669168 -2.669028
H 1.850292 -1.948659 -1.755015
H 3.216562 -0.950438 -2.253780
H 6.055447 -2.763795 -1.298148
H 5.889498 -1.080263 -0.780339
H 6.203306 -2.346280 0.417204
H 2.389854 -4.292361 0.417180
H 3.743856 -4.817354 -0.588265
H 4.013330 -4.416783 1.110786
H 3.938634 4.258473 -0.687058
H 4.906953 2.806364 -0.405799
H 3.314629 2.680530 -1.170091
H 5.152255 3.535059 2.613976
H 4.191521 5.000737 2.356953
H 3.700237 3.826746 3.583694
H 0.726894 3.206959 0.555126
H 0.884926 3.611584 2.267354
H 1.359242 4.789721 1.034526

TS_add_1,2_H_2O_B:
$E_{\omega B97X\text{-}D/6\text{-}311+G(d,p)}$= -3636.94263174

C 3.098945 -1.767031 -0.918732
C 2.700637 -1.054761 0.204766
C 3.628022 -1.065133 1.238525
C 4.840641 -1.731186 1.198860
C 5.182791 -2.435116 0.058398
C 4.303636 -2.449354 -1.008448
B 1.305654 -0.159652 0.391049

O 0.667559 -0.443821 1.694513
F 3.372661 -0.377380 2.375044
F 5.679204 -1.696501 2.236743
F 6.342400 -3.086120 -0.011582
F 4.623402 -3.121835 -2.116610
F 2.318165 -1.837744 -2.009688
C 1.819724 1.411971 0.242064
C 2.388942 1.857202 -0.945776
C 2.881759 3.133309 -1.144197
C 2.829411 4.047133 -0.105156
C 2.288477 3.654362 1.102895
C 1.806841 2.360131 1.253898
F 2.459014 1.024717 -1.999112
F 1.321620 2.074390 2.477495
F 2.239239 4.520239 2.119159
F 3.296128 5.283954 -0.268880
F 3.400067 3.495307 -2.320040
C 0.130090 -0.577847 -0.686819
C -0.249321 -1.912647 -0.783080
C -1.223607 -2.381346 -1.647401
C -1.890394 -1.487318 -2.467100
C -1.568982 -0.147186 -2.391971
C -0.592138 0.279069 -1.501760
F 0.371483 -2.843671 -0.041613
F -1.508850 -3.684255 -1.720092
F -2.827477 -1.914148 -3.310792
F -2.202442 0.729972 -3.176093
F -0.380517 1.605122 -1.480952
C -2.127936 -0.666034 2.469685
C -3.043157 -1.047633 1.404772
C -3.492598 -0.117988 0.492881
C -3.278069 1.277376 0.583004
Si -4.227550 2.478046 -0.585998
C -2.996856 3.616676 -1.410709
C -2.505861 1.857224 1.600973
P -1.748718 1.038865 2.894014
C -5.220413 1.482380 -1.817476
C -5.359377 3.439304 0.558049
Si -3.723542 -2.837726 1.399217
C -4.636371 -3.145942 -0.202684
C -2.311391 -4.031464 1.667414
C -4.905471 -2.883867 2.853971
H -1.064571 -0.780529 2.026649
H 1.257271 -0.306996 2.436864
H -2.093519 -1.337918 3.329079
H -4.135057 -0.465122 -0.309791
H -2.407293 2.940199 1.618507
H -5.768008 2.167291 -2.471946
H -4.587277 0.857608 -2.451313
H -5.955721 0.843761 -1.319680
H -5.969212 4.135324 -0.025945
H -6.033200 2.771377 1.101526
H -4.791321 4.022247 1.288232
H -2.347157 3.076530 -2.100574
H -3.538421 4.381807 -1.975404
H -2.367886 4.126036 -0.675557
H -4.999097 -4.178101 -0.214398
H -5.506000 -2.490092 -0.300903
H -4.002887 -3.012005 -1.081935
H -5.706738 -2.149192 2.736728
H -5.362460 -3.875512 2.926756
H -4.390336 -2.683354 3.797517
H -1.622328 -4.055127 0.822244
H -1.740283 -3.781472 2.565811
H -2.715899 -5.039506 1.800929

3T_add_1,2_H_2O_B:

$E_{\omega B97X\text{-}D/6\text{-}311+G(d,p)}$= -3636.97608712

C -0.768254 -0.075738 -1.562580
C -0.122091 -0.783937 -0.559304
C -0.525969 -2.110309 -0.440190
C -1.424757 -2.723027 -1.294834
C -1.999769 -1.985158 -2.313024
C -1.681826 -0.647145 -2.435047
B 1.093944 -0.252828 0.380494
C 1.675998 1.250977 0.079611
C 2.143921 1.557031 -1.194853
C 2.668742 2.784500 -1.553860
C 2.779629 3.780638 -0.598392
C 2.378577 3.513167 0.695545
C 1.857882 2.265665 1.008689
F 2.090890 0.626914 -2.159283
F 3.070817 3.015183 -2.802379
F 3.277511 4.969842 -0.919332
F 2.498050 4.450079 1.635404
F 1.534017 2.091583 2.304795
F 0.008741 -2.891087 0.511843
F -1.711139 -4.019519 -1.173642
F -2.848922 -2.556263 -3.161183
F -2.246249 0.079019 -3.399308
F -0.534098 1.229122 -1.754922
C 2.408867 -1.250149 0.348395
C 2.764745 -2.067515 -0.720141
C 3.935677 -2.811071 -0.753521
C 4.817385 -2.761985 0.310448
C 4.513744 -1.961313 1.396978
C 3.338175 -1.234383 1.380122
F 1.981805 -2.184343 -1.799730
F 3.105658 -0.460079 2.469675
F 5.345972 -1.894661 2.433184
F 5.939313 -3.471926 0.289902
F 4.217051 -3.576153 -1.806207
O 0.515708 -0.349808 1.857375
C -2.225940 -0.838884 2.387150
P -0.902617 0.387040 2.728095
C -1.355755 1.698978 1.599740
C -2.392996 1.669061 0.734615
Si -2.798092 3.286391 -0.193632
C -4.043398 4.218106 0.856604
C -3.180625 -0.657919 1.237269
Si -4.533018 -1.975344 1.051023
C -5.847293 -1.539207 2.322310
C -3.229369 0.482688 0.525804
C -1.214518 4.275715 -0.382249
C -3.541253 2.899746 -1.868943
C -5.286257 -1.927207 -0.663174
C -3.837593 -3.667791 1.459921
H -1.758583 -1.830148 2.367742
H 1.234746 -0.385730 2.502781
H -2.806505 -0.838407 3.321496
H -3.971077 0.563627 -0.264525
H -0.782737 2.612137 1.719020
H -3.780794 3.836329 -2.382220
H -2.848693 2.338282 -2.498683
H -4.468867 2.326739 -1.782139
H -4.321778 5.162066 0.377969
H -4.954903 3.629618 0.997890
H -3.629255 4.445388 1.843134
H -0.430486 3.673014 -0.849151
H -1.395856 5.148301 -1.016989
H -0.841543 4.641799 0.578588
H -6.125151 -2.628900 -0.706162
H -5.676539 -0.934601 -0.905153

H -4.576433 -2.213578 -1.441002
H -6.280341 -0.556771 2.112995
H -6.655226 -2.277464 2.308013
H -5.432131 -1.517968 3.334429
H -3.017323 -3.959195 0.801397
H -3.474673 -3.701221 2.491675
H -4.625279 -4.421569 1.364642

preTS_ isomerisation_1,2_H_2O_B:

$E_{\omega B97X\text{-}D/6\text{-}311+G(d,p)}$= -3636.97628993
C -1.870032 2.249021 -0.858412
C -1.641553 1.210971 0.033834
C -2.088919 1.464213 1.327825
C -2.654671 2.658815 1.732028
C -2.830305 3.674410 0.806774
C -2.443211 3.461132 -0.500569
B -1.090486 -0.286041 -0.345788
O -0.505400 -0.286701 -1.824994
P 1.022919 0.090082 -2.726977
C 1.598370 1.696757 -2.051214
C 2.436218 1.739417 -0.807122
Si 2.567488 3.377239 0.134581
C 3.477339 3.102369 1.748161
F -1.988756 0.506900 2.260609
F -3.039105 2.838366 2.994191
F -3.367507 4.832985 1.172358
F -2.609233 4.422333 -1.408142
F -1.543547 2.137676 -2.161550
C 0.107633 -0.911954 0.551768
C 0.445699 -2.251322 0.376976
C 1.390557 -2.911488 1.136568
C 2.080871 -2.213100 2.112262
C 1.814766 -0.870139 2.296326
C 0.845287 -0.250849 1.521374
F -0.172749 -2.982056 -0.561971
F 0.655925 1.050949 1.770198
F 2.489238 -0.180682 3.216147
F 2.985691 -2.830685 2.864881
F 1.639039 -4.207887 0.947154
C -2.438972 -1.237637 -0.382786
C -2.837250 -2.085298 0.646374
C -4.029716 -2.793906 0.632043
C -4.891524 -2.675761 -0.442931
C -4.545791 -1.842151 -1.491272
C -3.349705 -1.151861 -1.426969
F -2.072180 -2.270656 1.729300
F -4.350730 -3.592556 1.647898
F -6.034399 -3.351481 -0.468649
F -5.358673 -1.708265 -2.536419
F -3.079950 -0.339042 -2.479603
C 2.231334 -1.011101 -1.999827
C 3.218442 -0.613102 -1.160766
C 3.198241 0.681583 -0.476400
Si 4.754820 -1.717641 -0.931511
C 4.317124 -3.508254 -1.259426
C 5.996073 -1.109448 -2.201650
C 5.472825 -1.494338 0.785981
C 3.522121 4.561067 -0.969219
C 0.855735 4.080503 0.439627
H 2.241032 -2.013597 -2.418871
H 3.857296 0.766661 0.383514
H 0.758403 2.392997 -2.032655
H -1.220068 -0.211572 -2.472181
H 2.223914 2.045313 -2.889275
H 3.534720 4.043916 2.302836

H 2.961821 2.370853 2.375038
H 4.500822 2.753874 1.582240
H 3.615678 5.541957 -0.492901
H 4.528676 4.183812 -1.171886
H 3.013213 4.702587 -1.927699
H 0.936453 5.015414 1.002702
H 0.347464 4.310134 -0.501241
H 0.232707 3.390731 1.012683
H 6.358436 -2.129256 0.889102
H 5.789095 -0.461442 0.956903
H 4.775073 -1.772831 1.577773
H 6.931769 -1.671885 -2.125837
H 5.606420 -1.230625 -3.216434
H 6.224990 -0.050489 -2.049303
H 5.208836 -4.128513 -1.126219
H 3.548387 -3.878059 -0.577394
H 3.964917 -3.656163 -2.284101

TS_isomerisation_1,2_H_2O_B:
$E_{\omega B97X\text{-}D/6\text{-}311+G(d,p)}$= -3636.90163822

C 0.623817 -1.612290 0.910515
C -0.026856 -0.384566 0.822605
C 0.494554 0.601120 1.649059
C 1.557500 0.394379 2.516821
C 2.160809 -0.845714 2.571607
C 1.685196 -1.861615 1.758972
B -1.248688 -0.194958 -0.247415
C -2.426931 -1.333795 -0.175512
C -2.702960 -2.160819 0.905121
C -3.745553 -3.075375 0.919772
C -4.574185 -3.187590 -0.181069
C -4.350532 -2.373953 -1.277443
C -3.300695 -1.471746 -1.248823
F -1.961476 -2.111513 2.021801
F -3.956800 -3.846841 1.987174
F -5.577708 -4.060747 -0.183690
F -5.150035 -2.459480 -2.341806
F -3.164710 -0.690331 -2.334014
F -0.016562 1.840585 1.651647
F 2.006560 1.387488 3.285721
F 3.186134 -1.062862 3.388354
F 2.251362 -3.068704 1.806289
F 0.225634 -2.637724 0.145608
O -0.562641 -0.419765 -1.619904
P 0.894857 0.006768 -2.363021
C 2.111280 -1.240464 -2.047691
C 3.203271 -1.013827 -1.282077
Si 4.525779 -2.388689 -1.130855
C 5.774237 -2.052904 -2.488991
C 3.469198 0.263430 -0.609633
C 2.786189 1.413178 -0.748352
Si 3.467172 3.022843 0.003477
C 4.272572 3.945697 -1.419929
C 1.587783 1.535549 -1.654080
C 5.353599 -2.270746 0.546189
C 3.702036 -4.052826 -1.362317
C -2.015503 1.255650 -0.191361
C -2.749083 1.574850 0.947065
C -3.492177 2.730497 1.091946
C -3.533241 3.643621 0.050909
C -2.829719 3.372821 -1.105831
C -2.098247 2.196699 -1.205369
F -2.737202 0.734497 1.993582
F -4.162654 2.977320 2.216747
F -4.239774 4.764192 0.165466

F -2.860158 4.238410 -2.119564
F -1.451980 2.025558 -2.377054
C 2.080881 4.063598 0.712231
C 4.733235 2.602329 1.317530
H 1.935717 -2.200804 -2.520628
H 4.333747 0.267292 0.047183
H 0.770995 2.067861 -1.156590
H -0.553503 -0.089220 -2.891347
H 1.846355 2.164330 -2.516274
H 5.092791 3.525390 1.782448
H 4.305496 1.977460 2.105779
H 5.600283 2.084974 0.897534
H 4.704149 4.887128 -1.066299
H 5.073377 3.351703 -1.869385
H 3.546709 4.186805 -2.202359
H 2.477194 5.045650 0.988933
H 1.279678 4.225788 -0.014990
H 1.644586 3.611609 1.604291
H 6.083340 -3.080746 0.642407
H 5.893648 -1.327926 0.669539
H 4.640681 -2.369292 1.367421
H 6.581038 -2.791486 -2.457668
H 5.302830 -2.104664 -3.474424
H 6.220370 -1.060537 -2.375686
H 4.437967 -4.849277 -1.216399
H 2.896533 -4.199910 -0.638378
H 3.287879 -4.168652 -2.367714

3_add_1,2_H_2O_B:
$E_{\omega B97X\text{-}D/6\text{-}311+G(d,p)}$= -3637.00089845

C -2.118550 2.143722 -1.298225
C -2.044309 1.230558 -0.257474
C -2.848022 1.541705 0.834717
C -3.653690 2.661453 0.912203
C -3.686311 3.546179 -0.153120
C -2.910855 3.283957 -1.264856
B -1.206567 -0.186421 -0.247943
O -0.538350 -0.413335 -1.600224
P 0.808611 -0.009860 -2.251309
C 1.553561 1.531226 -1.656346
C 2.752850 1.387522 -0.748756
Si 3.452043 2.978160 0.023679
C 4.668290 2.523886 1.373370
F -2.847543 0.730555 1.904617
F -4.391929 2.901315 1.995877
F -4.451720 4.632895 -0.103461
F -2.930021 4.124918 -2.300227
F -1.403154 1.984126 -2.430505
C -0.010634 -0.272617 0.876407
C 0.716276 -1.453179 1.003304
C 1.776191 -1.617410 1.874542
C 2.163606 -0.560388 2.680644
C 1.474978 0.633011 2.596685
C 0.419280 0.755234 1.703237
F 0.394402 -2.519733 0.257166
F -0.178859 1.955880 1.683405
F 1.830819 1.660381 3.370225
F 3.183888 -0.694366 3.521748
F 2.420555 -2.782918 1.953117
C -2.336981 -1.375833 -0.129358
C -2.574419 -2.174741 0.981319
C -3.570484 -3.138434 1.032909
C -4.393209 -3.331704 -0.060675
C -4.210229 -2.547847 -1.185504
C -3.206021 -1.593770 -1.194219

F -1.839278 -2.047683 2.096860
F -3.743315 -3.878894 2.129504
F -5.353132 -4.252734 -0.029056
F -5.007670 -2.710265 -2.243344
F -3.118671 -0.846134 -2.305262
C 2.058813 -1.247781 -2.076208
C 3.158191 -1.037077 -1.322679
C 3.427003 0.231483 -0.626582
Si 4.483070 -2.414809 -1.225843
C 3.625855 -4.074988 -1.328707
C 5.624381 -2.144126 -2.688485
C 5.438388 -2.246295 0.377231
C 4.325254 3.881756 -1.371766
C 2.074447 4.058782 0.688202
H 1.869639 -2.196074 -2.568824
H 4.292179 0.220299 0.029052
H 0.769231 2.126123 -1.180645
H 0.508814 0.144987 -3.608011
H 1.840672 2.090962 -2.555333
H 5.031853 3.436682 1.855005
H 4.208336 1.902163 2.145563
H 5.537452 1.992535 0.975895
H 4.768036 4.812375 -1.003648
H 5.125387 3.268221 -1.795430
H 3.630950 4.140288 -2.176996
H 2.495037 5.024094 0.987286
H 1.309583 4.256198 -0.068819
H 1.585969 3.621647 1.560153
H 6.147986 -3.075374 0.460198
H 6.015113 -1.317742 0.412265
H 4.784484 -2.280879 1.251488
H 6.426373 -2.888552 -2.691409
H 5.077844 -2.228766 -3.631961
H 6.084471 -1.152384 -2.648408
H 4.362445 -4.877872 -1.228987
H 2.890352 -4.187081 -0.528009
H 3.115441 -4.213869 -2.285797

vdW_add_1,4_H_2O_B:
$E_{\omega B97X\text{-}D/6\text{-}311+G(d,p)}$= -3636.98555670

C 3.641975 0.076443 -0.044403
C 2.449086 -0.290084 -0.657656
C 2.586119 -0.773615 -1.948044
C 3.805713 -0.905750 -2.594189
C 4.963982 -0.537376 -1.936346
C 4.880782 -0.038985 -0.646094
B 1.065313 -0.046570 0.159703
C 0.904496 1.550208 0.450475
C 0.741546 2.422325 -0.619205
C 0.636539 3.794936 -0.488036
C 0.713113 4.355464 0.774940
C 0.895032 3.532794 1.872091
C 0.987681 2.160593 1.695181
F 0.686032 1.931515 -1.872546
F 0.460347 4.573289 -1.553358
F 0.605921 5.669182 0.932356
F 0.969759 4.064704 3.090130
F 1.171222 1.435501 2.805071
F 1.499543 -1.136544 -2.665711
F 3.866869 -1.376991 -3.837315
F 6.143367 -0.655649 -2.534711
F 5.988619 0.317592 -0.000962
F 3.616938 0.550297 1.209223
C 0.785343 -1.034752 1.419599
C 1.604223 -2.063621 1.862633

C 1.257101 -2.908433 2.908900
C 0.041609 -2.750743 3.548243
C -0.817446 -1.747680 3.131137
C -0.427028 -0.932444 2.088971
F 2.789297 -2.305933 1.290022
F -1.314977 0.003026 1.688360
F -2.003070 -1.594366 3.716017
F -0.304652 -3.558827 4.542284
F 2.083683 -3.878097 3.294054
O -0.147211 -0.461220 -0.817528
C -3.311137 -0.541340 -0.943043
C -2.842566 -1.455435 -1.902106
Si -2.845640 -3.306558 -1.460242
C -1.982287 -4.267461 -2.816006
C -3.311668 0.854279 -1.075765
C -2.822823 1.457466 -2.240858
P -2.222621 0.645452 -3.646125
C -2.352182 -1.006523 -3.129791
Si -4.011487 1.972132 0.297873
C -2.646615 3.131435 0.852219
C -5.427588 2.953747 -0.442925
C -4.620493 0.917475 1.721314
C -1.932334 -3.506505 0.168418
C -4.626032 -3.861265 -1.265127
H -2.003536 -1.746851 -3.846306
H -3.696131 -0.950134 -0.011183
H -2.827373 2.544822 -2.298612
H -5.851870 3.634351 0.301585
H -5.091968 3.554090 -1.293649
H -6.224828 2.290394 -0.790349
H -5.033338 1.561888 2.503419
H -5.413138 0.234090 1.402339
H -3.814730 0.328093 2.165715
H -3.004456 3.802679 1.638556
H -1.796189 2.569456 1.246080
H -2.295803 3.748469 0.019963
H -1.952745 -5.329591 -2.554997
H -2.502707 -4.175330 -3.773686
H -0.951058 -3.928168 -2.952145
H -4.672481 -4.916267 -0.978357
H -5.134379 -3.279637 -0.490229
H -5.180631 -3.737753 -2.199640
H -1.902710 -4.559972 0.462509
H -0.902381 -3.145582 0.093746
H -2.433096 -2.953997 0.968888
H -1.040125 -0.197193 -0.534877
H -0.059025 -0.284292 -1.765396

TS_add_1,4_H_2O_B:
$E_{\omega B97X\text{-}D/6\text{-}311+G(d,p)}$=

C -0.521905 -0.896140 2.065758
C 0.692763 -1.021124 1.401611
C 1.464390 -2.087238 1.837646
C 1.075041 -2.958393 2.848723
C -0.142528 -2.781354 3.474994
C -0.952479 -1.730644 3.078138
B 0.973746 -0.064679 0.072410
O -0.096013 -0.391677 -0.887572
F 2.659761 -2.357206 1.283059
F 1.864942 -3.971011 3.214005
F -0.536039 -3.609817 4.440552
F -2.139753 -1.548234 3.662705
F -1.376691 0.090965 1.720101
C 2.491642 -0.294928 -0.555848
C 2.766396 -0.958652 -1.741407

C 4.038057 -1.090500 -2.279596
C 5.118447 -0.545925 -1.612493
C 4.903582 0.112616 -0.414066
C 3.615636 0.216601 0.080957
F 1.781510 -1.558895 -2.449098
F 3.487810 0.832123 1.269343
F 5.938784 0.629275 0.251786
F 6.348783 -0.660228 -2.109755
F 4.228398 -1.742885 -3.429244
C 0.835745 1.557556 0.367652
C 0.850649 2.182959 1.607266
C 0.743780 3.554752 1.780327
C 0.619132 4.375774 0.675597
C 0.618144 3.805977 -0.583511
C 0.737029 2.430913 -0.710304
F 0.983879 1.471186 2.738552
F 0.749598 4.089999 3.003889
F 0.497361 5.694200 0.822067
F 0.507493 4.585311 -1.663696
F 0.792044 1.972814 -1.973673
C -3.123932 -0.524413 -0.914503
C -3.173364 0.924231 -1.150353
Si -4.039646 2.068476 0.125891
C -2.744802 3.272072 0.729221
C -2.510229 1.480479 -2.212130
P -1.655389 0.584628 -3.432381
C -2.105612 -1.068060 -3.099887
C -2.725670 -1.486843 -1.956120
Si -2.896558 -3.346561 -1.521328
C -2.011087 -4.345343 -2.827355
C -2.096650 -3.536709 0.163142
C -4.724940 -3.740323 -1.458369
C -5.408912 2.932812 -0.813994
C -4.713259 1.020147 1.519137
H -1.764002 -1.799122 -3.827135
H -3.918650 -0.890731 -0.262110
H -2.465338 2.561917 -2.314428
H -5.937006 3.623322 -0.149443
H -5.011051 3.510509 -1.652801
H -6.135771 2.215828 -1.205027
H -5.169554 1.670324 2.271527
H -5.484525 0.326441 1.172018
H -3.922604 0.447281 2.010217
H -3.165376 3.929404 1.495649
H -1.899079 2.734784 1.164838
H -2.373327 3.899128 -0.085543
H -2.077785 -5.409298 -2.581831
H -2.455736 -4.205253 -3.816612
H -0.951214 -4.081443 -2.883461
H -4.869459 -4.791451 -1.191332
H -5.242056 -3.131930 -0.711243
H -5.199002 -3.569780 -2.428596
H -2.100527 -4.588575 0.463320
H -1.059145 -3.189109 0.155561
H -2.640175 -2.972080 0.926012
H -2.199119 -0.558963 -0.274469
H 0.096272 -1.210796 -1.341496

4T_add_1,4_H_2O_B:
$E_{\omega B97X\text{-}D/6\text{-}311+G(d,p)}$= -3636.96320416

C 3.547622 0.941269 -0.613525
C 2.528023 0.004270 -0.712871
C 2.777915 -1.033576 -1.603262
C 3.922786 -1.124804 -2.375680
C 4.898966 -0.151500 -2.258116

C 4.711691 0.886382 -1.363640
B 1.144738 0.055735 0.146418
C 0.726194 1.558899 0.640377
C 0.571873 2.574071 -0.295886
C 0.170155 3.861451 0.006029
C -0.086802 4.194495 1.324687
C 0.084230 3.233803 2.303035
C 0.488039 1.952698 1.950976
F 0.843534 2.327072 -1.590408
F 0.038254 4.779445 -0.951471
F -0.481981 5.421371 1.644447
F -0.142771 3.543035 3.578897
F 0.652506 1.105466 2.976708
F 1.901353 -2.046328 -1.729497
F 4.099665 -2.144362 -3.216032
F 6.008512 -0.220295 -2.986857
F 5.652941 1.820174 -1.227037
F 3.460102 1.959591 0.256107
O -0.015391 -0.411984 -0.846058
P -0.694625 0.000000 -2.504815
C -1.700467 -1.506360 -2.470470
C -2.789009 -1.659868 -1.696570
C -3.407703 -0.530351 -0.919176
C -3.010602 0.887074 -1.219046
Si -4.038003 2.266551 -0.406967
C -3.594013 3.915874 -1.173265
C -1.921998 1.197746 -1.938504
C 1.062714 -1.080323 1.323633
C -0.154599 -1.401520 1.920389
C -0.310560 -2.361440 2.902496
C 0.801773 -3.055273 3.346859
C 2.038506 -2.764360 2.802885
C 2.148610 -1.794049 1.816309
F -1.269187 -0.735855 1.562752
F -1.505868 -2.608639 3.433972
F 0.682307 -3.981840 4.290082
F 3.117501 -3.417175 3.230587
F 3.390776 -1.565748 1.371402
C -5.862595 1.896104 -0.633614
C -3.579010 2.236207 1.412581
Si -3.517392 -3.394283 -1.404219
C -2.806040 -4.593230 -2.652987
C -2.985008 -3.847965 0.338737
C -5.385539 -3.314886 -1.532469
H -1.300527 -2.343694 -3.035867
H -4.497470 -0.610315 -1.017198
H -1.685867 2.234223 -2.160273
H -6.466429 2.700098 -0.202035
H -6.122096 1.811641 -1.692924
H -6.149047 0.964673 -0.136376
H -4.083119 3.042577 1.953591
H -3.860641 1.288407 1.880428
H -2.499982 2.364992 1.536934
H -4.206641 4.703680 -0.724653
H -2.546694 4.178877 -1.004684
H -3.777405 3.920369 -2.251423
H -3.244724 -5.583993 -2.501143
H -3.026514 -4.282518 -3.678185
H -1.721845 -4.691954 -2.549269
H -5.815497 -4.311206 -1.392691
H -5.813814 -2.659400 -0.768725
H -5.699494 -2.947004 -2.513565
H -3.327000 -4.853534 0.601190
H -1.893916 -3.829461 0.426422
H -3.395696 -3.152123 1.075766
H -3.231687 -0.716181 0.150783
H -0.379249 -1.270945 -0.603283

preTS_isomerisation_1,4_H_2O_B:
$E_{\omega B97X\text{-}D/6\text{-}311+G(d,p)}$= -3636.97616453
C -3.427603 -1.129247 -1.420865
C -2.533848 -1.181062 -0.360157
C -2.971732 -1.951915 0.712358
C -4.187463 -2.619320 0.725700
C -5.032597 -2.537339 -0.365890
C -4.646180 -1.781807 -1.458160
B -1.152878 -0.281305 -0.352955
C 0.028517 -0.922745 0.562650
C 0.307626 -2.280882 0.436333
C 1.249347 -2.947846 1.195129
C 2.000250 -2.239493 2.116353
C 1.796394 -0.879280 2.246867
C 0.826090 -0.252271 1.478104
F -0.364568 -3.025629 -0.453619
F 1.436231 -4.260629 1.056023
F 2.900317 -2.862229 2.870385
F 2.525966 -0.181692 3.117898
F 0.700143 1.066112 1.678352
F -2.221847 -2.098882 1.811750
F -4.546609 -3.344480 1.783103
F -6.197901 -3.174275 -0.365903
F -5.441665 -1.686209 -2.520850
F -3.117345 -0.396154 -2.519061
C -1.635367 1.250038 -0.018516
C -1.835760 2.260171 -0.949580
C -2.311656 3.522728 -0.626920
C -2.637938 3.815085 0.682603
C -2.496122 2.829609 1.644790
C -2.021211 1.584292 1.275819
F -1.568452 2.067114 -2.256572
F -1.944052 0.664583 2.247687
F -2.823342 3.086339 2.909915
F -3.087946 5.020372 1.013730
F -2.452309 4.452897 -1.570335
O -0.552510 -0.365033 -1.821550
P 1.002067 0.183929 -2.583621
C 2.168680 -1.022839 -1.937647
C 3.256914 -0.714535 -1.214374
Si 4.662167 -1.982877 -1.017628
C 5.430515 -1.801217 0.683170
C 1.394019 1.601280 -1.551525
C 2.484942 1.730495 -0.784572
C 3.514187 0.648861 -0.630698
Si 2.777052 3.378579 0.123477
C 1.125266 4.233436 0.360484
C 3.585049 3.016048 1.775411
C 3.923132 4.418421 -0.938039
C 4.018738 -3.716677 -1.306156
C 5.941852 -1.540858 -2.318951
H 2.040740 -2.028779 -2.330716
H 3.727153 0.521444 0.439644
H -1.243715 -0.312594 -2.495347
H 0.711307 2.437181 -1.658918
H 3.739504 3.947605 2.328263
H 2.963884 2.357677 2.387262
H 4.562665 2.541760 1.646184
H 4.140072 5.373061 -0.448944
H 4.874480 3.906390 -1.111107
H 3.469328 4.629938 -1.910458
H 1.256011 5.134711 0.966810
H 0.681133 4.539608 -0.590955
H 0.420174 3.575902 0.876300
H 6.260625 -2.506027 0.791174

H 5.832401 -0.793759 0.826872
H 4.720594 -2.001162 1.488198
H 6.801030 -2.215723 -2.255438
H 5.519966 -1.620801 -3.324802
H 6.309174 -0.519009 -2.184469
H 4.839349 -4.430834 -1.186952
H 3.233613 -3.991000 -0.598429
H 3.623969 -3.836541 -2.319007
H 4.467083 1.027066 -1.030428

TS_isomerisation_1,4_H_2O_B:
$E_{\omega B97X-D/6-311+G(d,p)}$= -3636.90215584

C -1.794275 2.276386 -0.945269
C -1.674234 1.260755 -0.007405
C -2.100985 1.611392 1.269340
C -2.546886 2.872349 1.619351
C -2.611301 3.861368 0.652183
C -2.237771 3.556221 -0.641547
B -1.219206 -0.288553 -0.307578
O -0.559129 -0.445251 -1.704526
P 0.929534 0.068334 -2.336659
C 1.443084 1.558493 -1.521065
C 2.569403 1.667633 -0.801355
Si 2.937573 3.333082 0.059289
C 3.699995 2.960225 1.728326
F -2.086260 0.691817 2.246070
F -2.917446 3.143391 2.870377
F -3.033493 5.082980 0.963838
F -2.303655 4.491645 -1.589424
F -1.466778 2.080787 -2.237641
C -0.058110 -0.909382 0.669321
C 0.241005 -2.267032 0.574699
C 1.221659 -2.896024 1.317554
C 1.987818 -2.151363 2.197067
C 1.759267 -0.793196 2.301546
C 0.758115 -0.203137 1.541897
F -0.439690 -3.048886 -0.275388
F 0.626263 1.122174 1.702249
F 2.501373 -0.060541 3.133521
F 2.927603 -2.736453 2.933545
F 1.436333 -4.206795 1.198478
C -2.611766 -1.154828 -0.346862
C -3.078657 -1.968899 0.678023
C -4.294160 -2.634979 0.632670
C -5.109579 -2.498208 -0.475156
C -4.696065 -1.688067 -1.517139
C -3.477400 -1.036053 -1.428667
F -2.356593 -2.161731 1.792256
F -4.682152 -3.408747 1.647486
F -6.277849 -3.131487 -0.535797
F -5.476115 -1.534509 -2.588295
F -3.161070 -0.239756 -2.462796
C 2.201867 -1.113001 -1.948393
C 3.305605 -0.797938 -1.249219
C 3.552917 0.548708 -0.629345
Si 4.710785 -2.080896 -1.076521
C 4.016421 -3.800214 -1.321142
C 5.958389 -1.663494 -2.413173
C 5.495233 -1.886119 0.613291
C 4.143172 4.276264 -1.023088
C 1.318895 4.257615 0.249865
H 2.061195 -2.104844 -2.367387
H 3.707030 0.391366 0.447746
H -0.487239 -0.051448 -2.954568
H 0.778386 2.404834 -1.641610
H 3.915081 3.893970 2.256436

H 3.023703 2.367916 2.349366
H 4.641561 2.412262 1.626514
H 4.396053 5.236741 -0.563820
H 5.072885 3.717102 -1.163614
H 3.711900 4.474183 -2.008373
H 1.476762 5.161880 0.845004
H 0.904905 4.567992 -0.713737
H 0.576884 3.639202 0.763261
H 6.320387 -2.597028 0.718668
H 5.906036 -0.881550 0.750152
H 4.787968 -2.078893 1.422951
H 6.810386 -2.348424 -2.364408
H 5.509931 -1.748871 -3.406841
H 6.340844 -0.645106 -2.297954
H 4.813481 -4.536858 -1.182104
H 3.224550 -4.025455 -0.602732
H 3.615085 -3.937353 -2.329099
H 4.537088 0.909651 -0.962768

4_isomerisation_1,4_H_2O_B:
$E_{\omega B97X\text{-}D/6\text{-}311+G(d,p)}$= -3637.00092887

C -3.337027 -1.222337 -1.400553
C -2.442525 -1.265874 -0.334669
C -2.798924 -2.148546 0.677100
C -3.929403 -2.951298 0.632199
C -4.772461 -2.887186 -0.460652
C -4.473111 -2.009882 -1.487162
B -1.152812 -0.239817 -0.327952
C 0.018569 -0.656950 0.755607
C 0.527479 -1.952141 0.722787
C 1.488375 -2.430774 1.593463
C 1.998934 -1.589406 2.566993
C 1.551979 -0.283769 2.625081
C 0.598566 0.159010 1.717343
F 0.075880 -2.823365 -0.191570
F 1.919742 -3.690716 1.513086
F 2.912317 -2.029227 3.426908
F 2.040582 0.540206 3.553691
F 0.267605 1.454797 1.825628
F -2.052672 -2.274916 1.785801
F -4.210016 -3.784589 1.636061
F -5.860415 -3.651140 -0.520939
F -5.286497 -1.921140 -2.541657
F -3.142572 -0.365880 -2.414531
C -1.802659 1.257692 -0.101566
C -1.896883 2.270733 -1.044621
C -2.508217 3.493665 -0.800816
C -3.087747 3.736785 0.428406
C -3.050890 2.745341 1.394633
C -2.427398 1.546069 1.107618
F -1.386989 2.131651 -2.284555
F -2.430824 0.624024 2.084140
F -3.612852 2.958053 2.584856
F -3.678434 4.902041 0.679573
F -2.545821 4.431806 -1.748755
O -0.530099 -0.363727 -1.712430
P 0.845615 0.113278 -2.263733
C 2.130273 -1.090671 -2.068373
C 3.216554 -0.863869 -1.314777
Si 4.577958 -2.197475 -1.183566
C 5.146334 -2.294654 0.598978
C 1.460075 1.597352 -1.521176
C 2.586574 1.622158 -0.794877
C 3.468932 0.425508 -0.580910
Si 3.131493 3.273694 -0.003782

C 1.658172 4.427895 0.023527
C 3.748576 2.917376 1.727102
C 4.520050 3.947532 -1.067823
C 3.889041 -3.833872 -1.770029
C 5.987617 -1.616571 -2.275206
H 1.970314 -2.040211 -2.570395
H 3.464687 0.198968 0.495583
H 0.602925 0.312477 -3.627050
H 0.854209 2.485471 -1.654302
H 4.114922 3.841163 2.185041
H 2.953935 2.520920 2.362960
H 4.574816 2.200293 1.722768
H 4.892125 4.889739 -0.654214
H 5.360197 3.248300 -1.114237
H 4.176529 4.136627 -2.088571
H 1.918155 5.345389 0.559680
H 1.342250 4.714013 -0.983717
H 0.808092 3.967195 0.535089
H 6.016577 -2.954192 0.669855
H 5.442893 -1.314798 0.984202
H 4.371945 -2.701628 1.252655
H 6.805495 -2.343171 -2.253832
H 5.660899 -1.503560 -3.312591
H 6.386844 -0.656207 -1.936089
H 4.640678 -4.617231 -1.634350
H 3.000407 -4.119320 -1.200587
H 3.626662 -3.808973 -2.831378
H 4.508328 0.731054 -0.763867

vdW_add_1,2_$(H_2O)_2$_B:
$E_{\omega B97X\text{-}D/6\text{-}311+G(d,p)}$= -3713.43862012

C 2.196319 2.113535 -0.989662
C 1.740854 1.485878 0.164432
C 1.688650 2.302007 1.283122
C 2.024823 3.648227 1.265394
C 2.453924 4.228127 0.087329
C 2.543567 3.449316 -1.055004
B 1.410773 -0.113287 0.108725
C 0.163115 -0.557474 -0.846641
C -0.142820 -1.912503 -0.959285
C -1.156447 -2.410693 -1.754455
C -1.933889 -1.531325 -2.487581
C -1.689970 -0.175973 -2.390035
C -0.669413 0.285590 -1.569915
F 0.591734 -2.822082 -0.300675
F -1.375991 -3.722756 -1.842935
F -2.896733 -1.991382 -3.278829
F -2.432762 0.681990 -3.089105
F -0.522240 1.615193 -1.530177
F 1.310528 1.815703 2.480649
F 1.947596 4.379964 2.376581
F 2.782498 5.515615 0.050064
F 2.958819 3.993626 -2.197869
F 2.296569 1.406895 -2.126165
C 2.819230 -0.886818 -0.201650
C 3.837683 -0.860750 0.741617
C 5.087149 -1.424591 0.555549
C 5.370046 -2.044841 -0.648345
C 4.398703 -2.080358 -1.632251
C 3.157747 -1.506611 -1.398444
F 3.628796 -0.244105 1.920557
F 2.288117 -1.574442 -2.414683
F 4.663937 -2.665579 -2.799432
F 6.561704 -2.593470 -0.858855
F 6.013137 -1.368421 1.512118

O 0.894506 -0.635280 1.505379
O 1.626104 -0.609492 3.971990
C -1.990027 0.285244 2.361753
P -1.961993 1.966734 1.938075
C -3.125775 1.985452 0.659505
C -3.849355 0.891859 0.181122
Si -5.306474 1.199203 -1.004233
C -6.807241 1.482303 0.089813
C -2.748371 -0.714297 1.738236
Si -2.733944 -2.480289 2.447107
C -3.815303 -2.461728 3.980678
C -3.617844 -0.394842 0.687704
C -4.975089 2.728774 -2.036076
C -5.608795 -0.292536 -2.097499
C -3.423614 -3.675434 1.178986
C -0.984195 -2.965186 2.919230
H -0.037739 -0.405321 1.657175
H 1.369224 -0.553054 2.385546
H -1.387230 0.011448 3.229746
H -4.189685 -1.210372 0.251846
H -3.315780 2.962754 0.220874
H -6.524605 -0.134134 -2.675854
H -4.792652 -0.452823 -2.803767
H -5.743926 -1.208567 -1.515300
H -7.696236 1.686133 -0.515251
H -7.011488 0.602146 0.707022
H -6.648081 2.333838 0.757979
H -4.927770 3.629846 -1.417860
H -4.037748 2.643000 -2.590612
H -5.785587 2.868493 -2.758077
H -3.373379 -4.695562 1.571879
H -4.471219 -3.463344 0.947083
H -2.855037 -3.650757 0.246072
H -4.839487 -2.167798 3.733147
H -3.847788 -3.452478 4.444424
H -3.430213 -1.753829 4.720765
H -0.999798 -3.952270 3.391864
H -0.327314 -3.020937 2.048049
H -0.544893 -2.266237 3.637466
H 2.146682 -1.353109 4.287429
H 2.045394 0.178890 4.327466

TS_add_1,2_(H_2O)$_2$_B:
$E_{\omega B97X\text{-}D/6\text{-}311+G(d,p)}$= -3713.38951924

C 0.484208 1.653006 -1.025702
C -0.043819 0.374713 -0.877816
C 0.574003 -0.588753 -1.658821
C 1.595313 -0.310347 -2.558891
C 2.069902 0.980460 -2.680443
C 1.506150 1.975034 -1.900853
B -1.249928 0.141872 0.227202
C -1.913165 -1.378990 0.166688
C -1.952788 -2.286340 1.213836
C -2.568087 -3.529179 1.133392
C -3.192266 -3.910402 -0.037614
C -3.190302 -3.036927 -1.112028
C -2.564307 -1.811225 -0.983311
F -1.387351 -2.011323 2.405150
F -2.565490 -4.357449 2.182319
F -3.787932 -5.098293 -0.132886
F -3.784427 -3.389821 -2.254490
F -2.584974 -1.018833 -2.069702
F 0.205127 -1.879272 -1.599001
F 2.114466 -1.279663 -3.318858
F 3.051567 1.264115 -3.534244

F 1.937108 3.234346 -2.019419
F -0.027409 2.676511 -0.323220
C -2.553495 1.155118 -0.026071
C -2.852236 1.876659 -1.174605
C -3.981393 2.670905 -1.312770
C -4.887639 2.764524 -0.273107
C -4.649034 2.051123 0.887215
C -3.508691 1.271112 0.975266
F -2.046391 1.840553 -2.249255
F -3.361532 0.576875 2.123680
F -5.518360 2.114294 1.899229
F -5.975818 3.523773 -0.389507
F -4.204015 3.345939 -2.443425
O -0.572852 0.441076 1.499592
C 2.121727 0.769491 2.388494
P 1.660081 -0.903351 2.852061
C 2.441064 -1.793720 1.619235
C 3.262691 -1.276363 0.609054
Si 4.213124 -2.541505 -0.484191
C 5.138126 -1.633351 -1.831831
C 3.091637 1.079938 1.351978
Si 3.835426 2.843437 1.313461
C 2.447991 4.091814 1.404893
C 3.534134 0.106519 0.483787
C 5.415089 -3.360502 0.698769
C 2.998796 -3.790534 -1.161266
C 4.905730 2.931708 2.850643
C 4.873748 3.038249 -0.228926
O -1.300080 0.478828 4.249195
H 1.079801 0.873898 1.878119
H -1.108877 0.441576 2.303852
H 2.056056 1.479669 3.213716
H 4.213487 0.404772 -0.308020
H 2.304299 -2.871464 1.671273
H 5.664215 -2.362302 -2.455793
H 4.476847 -1.060587 -2.485201
H 5.888153 -0.955273 -1.414964
H 6.018284 -4.100333 0.163757
H 6.093224 -2.626589 1.142834
H 4.889387 -3.875147 1.507770
H 2.455961 -4.296341 -0.358048
H 2.269796 -3.330498 -1.829684
H 3.544772 -4.553112 -1.725110
H 5.299420 4.046055 -0.250005
H 5.705652 2.328553 -0.245624
H 4.288532 2.910181 -1.142272
H 5.690065 2.170040 2.831250
H 5.387144 3.912621 2.910130
H 4.315618 2.793669 3.760976
H 2.870835 5.093797 1.525924
H 1.837884 4.087312 0.500388
H 1.792342 3.900775 2.259138
H -2.128179 0.914735 4.463722
H -1.452173 -0.449595 4.442362

3T H_2O_add_1,2_(H_2O)$_2$_B:
$E_{\omega B97X\text{-}D/6\text{-}311+G(d,p)}$= -3713.43113011

C -2.117924 1.653002 1.205473
C -1.626614 1.296121 -0.047171
C -1.823732 2.257620 -1.028251
C -2.379235 3.505317 -0.779327
C -2.801640 3.826784 0.495139
C -2.677945 2.881805 1.499419
B -1.032254 -0.219103 -0.247083
C 0.181736 -0.662137 0.754206

C 0.637452 -1.974628 0.696211
C 1.570016 -2.512463 1.563847
C 2.132004 -1.703791 2.534486
C 1.763794 -0.374855 2.594639
C 0.816879 0.118418 1.708870
F 0.117601 -2.823055 -0.210210
F 1.904340 -3.802440 1.501278
F 3.015735 -2.199327 3.396238
F 2.310888 0.418449 3.516205
F 0.543093 1.424575 1.838037
F -1.483532 2.033479 -2.308793
F -2.512352 4.393091 -1.765579
F -3.333075 5.018261 0.751660
F -3.103041 3.161823 2.731186
F -2.060579 0.769088 2.214438
C -2.359941 -1.196708 -0.148784
C -3.352175 -1.112815 -1.122559
C -4.536428 -1.828024 -1.091070
C -4.789412 -2.671048 -0.024117
C -3.850652 -2.772921 0.984784
C -2.672339 -2.043883 0.909656
F -3.190159 -0.294398 -2.175401
F -1.841511 -2.201712 1.948599
F -4.084776 -3.571210 2.025905
F -5.920179 -3.366469 0.032730
F -5.432548 -1.706369 -2.070368
O -0.452729 -0.432674 -1.690448
P 0.843942 0.294187 -2.678513
C 1.334976 1.659742 -1.621129
C 2.397455 1.663628 -0.787875
Si 2.846036 3.324440 0.033891
C 4.075526 4.178025 -1.099093
C 3.229060 0.481156 -0.537327
C 3.186170 -0.673078 -1.226682
Si 4.516716 -2.002989 -1.004160
C 5.814860 -1.664869 -2.321650
C 2.245727 -0.860428 -2.386935
C 1.283089 4.348918 0.208083
C 3.629219 3.028328 1.709813
C 5.310622 -1.889723 0.688891
C 3.776260 -3.698799 -1.313177
O -1.228088 -2.490146 -3.091704
H 1.842145 -1.878859 -2.418695
H -0.849348 -1.192791 -2.196075
H 2.825955 -0.773358 -3.318221
H 3.961093 0.579220 0.260214
H 0.768186 2.571882 -1.767609
H 3.899343 3.989976 2.157523
H 2.944607 2.520964 2.391975
H 4.543782 2.433034 1.634290
H 4.378078 5.144380 -0.683942
H 4.975612 3.569624 -1.229193
H 3.638584 4.353119 -2.086469
H 0.882620 4.657370 -0.761837
H 0.507384 3.789236 0.738034
H 1.497089 5.257857 0.778514
H 6.142496 -2.598839 0.744343
H 5.716752 -0.891863 0.877546
H 4.616190 -2.132548 1.494797
H 6.276268 -0.684497 -2.171264
H 6.604795 -2.421732 -2.286220
H 5.378797 -1.683857 -3.324963
H 4.536678 -4.469575 -1.153987
H 2.935892 -3.918115 -0.651812
H 3.429160 -3.788555 -2.347030
H -1.418305 -3.299475 -2.610126
H -1.913690 -2.403904 -3.759238

preTS_isomerisation_1,2_(H_2O)$_2$_B
$E_{\omega B97X\text{-}D/6\text{-}311+G(d,p)}$= -3713.42918092

C 0.918990 -0.164506 1.626107
C 0.181220 -0.852573 0.676753
C 0.555316 -2.182457 0.509230
C 1.527174 -2.813655 1.257711
C 2.213606 -2.089712 2.217422
C 1.916146 -0.752497 2.391789
B -1.031895 -0.275386 -0.247727
C -2.403892 -1.193536 -0.187114
C -3.413042 -1.002940 -1.127133
C -4.627539 -1.664792 -1.111192
C -4.896053 -2.561132 -0.093060
C -3.941861 -2.767520 0.884833
C -2.733664 -2.088239 0.826232
F -3.237321 -0.124863 -2.127784
F -5.537994 -1.441761 -2.059388
F -6.056523 -3.207995 -0.051626
F -4.190928 -3.617444 1.881585
F -1.889610 -2.346162 1.835058
F -0.061960 -2.937379 -0.415654
F 1.805561 -4.105700 1.073767
F 3.145202 -2.677773 2.962994
F 2.589506 -0.036219 3.292908
F 0.708555 1.137283 1.862069
C -1.569114 1.242508 0.058280
C -1.827142 2.235655 -0.876670
C -2.425586 3.448150 -0.560905
C -2.809636 3.708064 0.738523
C -2.607581 2.735600 1.703429
C -2.017612 1.539005 1.343137
F -1.512432 2.082556 -2.173847
F -1.908143 0.615584 2.310762
F -2.993893 2.956311 2.959607
F -3.369887 4.870205 1.059394
F -2.619689 4.369203 -1.505840
O -0.480737 -0.385140 -1.722201
P 0.989776 0.028931 -2.646916
C 1.535960 1.663640 -2.009895
C 2.411238 1.754986 -0.792125
Si 2.550058 3.421260 0.095089
C 0.846510 4.132046 0.424146
C 2.278823 -1.011235 -1.942184
C 3.264541 -0.576023 -1.123452
C 3.215184 0.729866 -0.460200
Si 4.834258 -1.629390 -0.899268
C 5.566818 -1.364980 0.806632
C 4.445185 -3.437542 -1.195452
C 6.048665 -1.008457 -2.189941
C 3.505846 3.206787 1.691958
C 3.469782 4.576081 -1.068837
O -1.597141 -2.191091 -3.256129
H -0.985157 -1.023619 -2.296082
H 2.319523 -2.017334 -2.352282
H 3.892806 0.854979 0.380203
H 0.684114 2.341994 -1.979281
H 2.130117 2.010954 -2.870592
H 3.565613 4.165834 2.215605
H 3.016401 2.488926 2.354455
H 4.529095 2.865707 1.509931
H 3.568342 5.572298 -0.626380
H 4.473635 4.198410 -1.284057
H 2.936944 4.683953 -2.018722
H 0.940494 5.078109 0.966241
H 0.319310 4.342916 -0.510501

H 0.235385 3.454411 1.023777
H 4.889285 -1.662106 1.609132
H 6.476764 -1.965577 0.903870
H 5.846281 -0.319528 0.965181
H 6.999146 -1.545993 -2.116945
H 5.652097 -1.151996 -3.199178
H 6.252425 0.057888 -2.053740
H 5.354342 -4.031754 -1.061417
H 3.692877 -3.815219 -0.499298
H 4.086950 -3.611220 -2.214081
H -1.816902 -1.955000 -4.160912
H -1.108142 -3.016554 -3.301575

TS_isomerisation_1,2_$(H_2O)_2$_B
$E_{\omega B97X\text{-}D/6\text{-}311+G(d,p)}$= -3713.40484382

C 3.410062 -1.082831 1.177772
C 2.403601 -1.250460 0.235647
C 2.733704 -2.152611 -0.771956
C 3.932924 -2.849238 -0.819900
C 4.884556 -2.656755 0.163502
C 4.619126 -1.755451 1.177363
B 1.029174 -0.309415 0.301093
C -0.173853 -0.925621 -0.630133
C -0.536397 -2.255963 -0.439864
C -1.512478 -2.904607 -1.168708
C -2.216118 -2.201276 -2.130605
C -1.927145 -0.866105 -2.330722
C -0.923958 -0.261576 -1.586280
F 0.091943 -2.996698 0.488546
F -1.782924 -4.195885 -0.959962
F -3.154611 -2.807247 -2.855320
F -2.613217 -0.168721 -3.238788
F -0.719380 1.037082 -1.853740
F 1.895843 -2.407330 -1.787567
F 4.176495 -3.706094 -1.812106
F 6.037328 -3.318956 0.130557
F 5.524909 -1.536007 2.131204
F 3.250375 -0.187777 2.181694
C 1.598188 1.184418 -0.121107
C 2.026314 1.420483 -1.424673
C 2.605674 2.600045 -1.853446
C 2.818797 3.620838 -0.942451
C 2.455352 3.424229 0.373822
C 1.867401 2.225910 0.756368
F 1.898618 0.455491 -2.348981
F 1.574326 2.142797 2.070534
F 2.660752 4.392019 1.270002
F 3.370155 4.767994 -1.329658
F 2.969741 2.760755 -3.126097
O 0.526340 -0.316446 1.729902
P -0.906752 0.075667 2.470403
C -1.531095 1.714735 1.918067
C -2.441178 1.776448 0.721321
Si -2.571108 3.407473 -0.225102
C -3.476038 4.622731 0.888886
C -2.236130 -0.988473 1.886721
C -3.261954 -0.562624 1.116431
C -3.244739 0.738236 0.433687
Si -4.828012 -1.627900 0.957547
C -6.033170 -0.969429 2.239392
C -5.587113 -1.432830 -0.746789
C -4.424969 -3.422192 1.315019
C -0.856908 4.083778 -0.579643
C -3.527452 3.144293 -1.814723
O 1.339092 -0.451510 4.102984
H 1.474002 -0.430623 3.095028

H -2.241907 -1.991860 2.304329
H -3.941997 0.838764 -0.394342
H -0.691306 2.406513 1.849578
H -2.103655 2.044436 2.797435
H -3.581126 4.083334 -2.374116
H -3.041797 2.398287 -2.448401
H -4.552757 2.815795 -1.621012
H -3.557772 5.602642 0.408127
H -4.486330 4.269593 1.115560
H -2.944210 4.758530 1.835842
H -0.936092 5.006045 -1.163570
H -0.327935 4.328192 0.346092
H -0.252700 3.371761 -1.145647
H -4.916170 -1.752731 -1.546178
H -6.493064 -2.043877 -0.810821
H -5.877128 -0.395969 -0.939503
H -6.980993 -1.515001 2.197048
H -5.624428 -1.073860 3.248658
H -6.245667 0.090109 2.067486
H -5.331293 -4.027134 1.213773
H -3.677731 -3.820120 0.624436
H -4.052953 -3.555436 2.334911
H 1.871504 0.235264 4.529537
H 0.339960 -0.192379 4.096058

3 H_2O_isomerisation_1,2_(H_2O)$_2$_B
$E_{\omega B97X\text{-}D/6\text{-}311+G(d,p)}$= -3713.44857495

C 1.831341 2.266750 0.683380
C 1.605063 1.218263 -0.197350
C 2.039270 1.464131 -1.496782
C 2.592617 2.658030 -1.919122
C 2.768132 3.683991 -1.005422
C 2.392271 3.479795 0.306334
B 1.060954 -0.284883 0.200269
O 0.527482 -0.303516 1.633290
P -0.843855 0.005094 2.304310
C -2.187542 -1.088358 1.916282
C -3.216426 -0.671003 1.146551
Si -4.772632 -1.766803 0.984229
C -4.311469 -3.549438 1.312583
F 1.933707 0.497901 -2.422332
F 2.965183 2.828630 -3.187514
F 3.294733 4.844097 -1.386650
F 2.557215 4.453977 1.202832
F 1.507557 2.178157 1.987127
C 2.425625 -1.217068 0.194980
C 3.382968 -1.049403 1.188404
C 4.580057 -1.741577 1.246738
C 4.882068 -2.652583 0.251266
C 3.979448 -2.839493 -0.778412
C 2.789205 -2.126873 -0.791815
F 3.192268 -0.139901 2.166111
F 1.990708 -2.373198 -1.841164
F 4.260318 -3.705473 -1.752739
F 6.026564 -3.329236 0.278911
F 5.444680 -1.528492 2.239324
C -0.140895 -0.892620 -0.740807
C -0.502282 -2.228700 -0.587876
C -1.487505 -2.853396 -1.326696
C -2.197177 -2.121127 -2.262753
C -1.912645 -0.779203 -2.421865
C -0.905100 -0.197443 -1.665344
F 0.125269 -2.992735 0.319761
F -1.760545 -4.147493 -1.151530
F -3.140171 -2.703990 -2.997852

F -2.607677 -0.053330 -3.298482
F -0.713551 1.112141 -1.881790
C -1.543483 1.641719 2.004786
C -2.483299 1.712642 0.824893
C -3.232503 0.647123 0.491872
Si -2.685779 3.381153 -0.053359
C -3.677392 3.131703 -1.620685
C -3.587990 4.518227 1.137439
C -0.991747 4.093435 -0.426224
C -5.957359 -1.129697 2.291164
C -5.533666 -1.552346 -0.713570
O 1.526983 -0.450795 4.587391
H 1.936138 -0.354274 3.720902
H -2.155485 -2.081436 2.352294
H -3.937078 0.750345 -0.328191
H -0.741822 2.377846 1.964223
H -2.109852 1.852229 2.923746
H -3.775691 4.085613 -2.147605
H -3.187281 2.423344 -2.293089
H -4.686274 2.765945 -1.408971
H -3.712437 5.512602 0.697688
H -4.580267 4.126461 1.378751
H -3.031286 4.634708 2.072358
H -1.101477 5.025487 -0.989106
H -0.447549 4.331597 0.492282
H -0.386622 3.405609 -1.020774
H -4.861441 -1.854512 -1.518551
H -6.431402 -2.174613 -0.782740
H -5.838799 -0.517397 -0.891507
H -6.898193 -1.687545 2.260235
H -5.529772 -1.237579 3.291939
H -6.186569 -0.072165 2.130103
H -5.197965 -4.181196 1.201919
H -3.553381 -3.910672 0.613571
H -3.935012 -3.687760 2.330051
H 2.159331 -0.073615 5.201784
H -0.558518 -0.099826 3.667260

vdW_add_1,4_$(H_2O)_2$_B:
$E_{\omega B97X\text{-}D/6\text{-}311+G(d,p)}$= -3713.44320740

C 0.286346 -0.872178 2.542134
C 0.245553 -1.054189 1.166437
C -0.685541 -1.993611 0.738308
C -1.498669 -2.723535 1.581072
C -1.415795 -2.510318 2.946663
C -0.517795 -1.577861 3.428144
B 1.119110 -0.234986 0.056781
O 0.054469 0.516238 -0.832251
F -0.816461 -2.243990 -0.577784
F -2.353835 -3.624260 1.096911
F -2.196741 -3.188634 3.779941
F -0.438735 -1.351789 4.738174
F 1.112503 0.020024 3.103219
C 2.143279 0.876560 0.671929
C 3.329872 0.452109 1.258822
C 4.288433 1.310312 1.765056
C 4.076626 2.677399 1.699369
C 2.907864 3.150259 1.134160
C 1.974567 2.250950 0.639863
F 3.575683 -0.861831 1.381422
F 0.858737 2.797616 0.125261
F 2.683509 4.462874 1.069678
F 4.983947 3.522373 2.179457
F 5.404371 0.838217 2.319595
C 1.947475 -1.153438 -1.014096

C 2.265582 -2.495641 -0.850989
C 3.005103 -3.221023 -1.772113
C 3.470622 -2.601325 -2.917289
C 3.196530 -1.260415 -3.116947
C 2.458196 -0.571359 -2.169967
F 1.877894 -3.169499 0.240342
F 3.274747 -4.509342 -1.561458
F 4.178610 -3.283791 -3.811367
F 3.647015 -0.645564 -4.210721
F 2.248475 0.731279 -2.417972
O -0.818043 -0.677871 -2.921465
C -2.150398 2.414937 0.955803
P -2.522246 1.311005 2.239193
C -3.465013 0.138780 1.380550
C -3.780293 0.164273 0.020229
Si -4.922236 -1.149323 -0.745161
C -6.361220 -0.251468 -1.548079
C -2.558075 2.304866 -0.378319
Si -2.171694 3.679956 -1.639735
C -1.023687 2.996065 -2.958735
C -3.323704 1.206201 -0.798118
C -3.797333 4.188182 -2.427602
C -1.393135 5.138799 -0.762390
C -5.540776 -2.305254 0.590892
C -3.970152 -2.093783 -2.061538
H -3.855831 -0.678381 1.983093
H -3.610671 1.175290 -1.848829
H -1.557896 3.280595 1.240835
H -1.220489 5.946597 -1.480046
H -2.049340 5.525091 0.023061
H -0.431779 4.880812 -0.312139
H -0.775902 3.776137 -3.684994
H -0.090519 2.621136 -2.531305
H -1.509726 2.181871 -3.505195
H -3.631617 4.973983 -3.171025
H -4.279184 3.346174 -2.933084
H -4.492393 4.572946 -1.675883
H -7.040610 -0.964497 -2.025134
H -6.930256 0.315582 -0.805877
H -6.017237 0.448545 -2.315384
H -6.223649 -3.039640 0.153098
H -4.725833 -2.853388 1.069576
H -6.089753 -1.759104 1.363627
H -4.594715 -2.878494 -2.498385
H -3.679319 -1.421329 -2.875878
H -3.080447 -2.569715 -1.640017
H -0.335925 0.032932 -1.623555
H -1.018053 -0.178085 -3.716658
H -1.510457 -1.337595 -2.820960
H -0.630872 1.027668 -0.374223

TS_add_1,4_$(H_2O)_2$_B:
$E_{\omega B97X\text{-}D/6\text{-}311+G(d,p)}$= -3713.39077714

C -0.042479 2.288973 0.329897
C 0.683999 1.468201 -0.525679
C 0.751165 1.925697 -1.834092
C 0.179894 3.117404 -2.262687
C -0.502953 3.911144 -1.361954
C -0.616073 3.489511 -0.047991
B 1.245488 0.019341 0.021531
O -0.010806 -0.765428 0.231866
F 1.379163 1.220933 -2.789011
F 0.285144 3.501387 -3.535939
F -1.048338 5.061615 -1.749987
F -1.275597 4.243626 0.834668

F -0.212658 1.937482 1.614821
C 2.091473 0.123863 1.447727
C 2.706565 1.252074 1.974063
C 3.429150 1.249543 3.158252
C 3.570457 0.075023 3.873946
C 2.989309 -1.081554 3.386215
C 2.280369 -1.029741 2.198305
F 2.629328 2.437700 1.349641
F 1.768720 -2.204392 1.768922
F 3.123970 -2.228040 4.056521
F 4.258854 0.056144 5.013316
F 3.989220 2.371312 3.615621
C 2.284237 -0.736259 -1.020922
C 2.122144 -2.007905 -1.547805
C 3.048616 -2.627573 -2.374844
C 4.216196 -1.967420 -2.703216
C 4.435429 -0.698266 -2.193776
C 3.481933 -0.124503 -1.373693
F 1.021869 -2.744646 -1.270824
F 2.823653 -3.854945 -2.848168
F 5.119371 -2.542612 -3.493894
F 5.557367 -0.045901 -2.502172
F 3.750048 1.111634 -0.922314
P -1.721718 -0.737892 -2.008292
C -2.685537 0.555154 -1.364679
C -3.666215 0.421031 -0.404628
Si -4.644892 1.937444 0.232871
C -4.043241 2.283660 1.970316
C -2.470752 -2.138669 -1.297483
C -3.440186 -2.111164 -0.321930
C -3.915640 -0.857072 0.217154
Si -4.112252 -3.721415 0.458305
C -3.281086 -5.185066 -0.353578
C -3.709712 -3.637646 2.287800
C -5.964149 -3.737440 0.180960
C -6.459362 1.468758 0.226115
C -4.334555 3.374831 -0.921819
O -1.933517 -0.521381 2.029449
H -2.470004 1.541446 -1.767798
H -4.819744 -0.919013 0.823708
H -2.095784 -3.096363 -1.647136
H -3.662972 -6.112895 0.082381
H -3.480289 -5.216282 -1.428509
H -2.197805 -5.168033 -0.204136
H -4.124873 -4.508153 2.804164
H -2.627989 -3.640353 2.450679
H -4.133074 -2.743587 2.755409
H -6.402894 -4.638542 0.619757
H -6.446572 -2.871422 0.643101
H -6.201923 -3.730412 -0.886337
H -7.064671 2.326297 0.535187
H -6.786621 1.168100 -0.773135
H -6.672000 0.648037 0.916981
H -4.942525 4.229278 -0.610344
H -3.291426 3.696820 -0.911796
H -4.607962 3.126410 -1.951135
H -4.547099 3.167229 2.373549
H -4.253795 1.439658 2.632908
H -2.966215 2.464579 1.987437
H -1.132509 -0.531239 1.438526
H -1.819597 -1.229057 2.670244
H -3.032569 -0.733465 1.109589
H 0.168843 -1.706484 0.275812

4T H_2O_add_1,4_(H_2O)$_2$_B:
$E_{\omega B97X\text{-}D/6\text{-}311+G(d,p)}$= -3713.42661053

C 1.131020 2.364781 -1.566978
C 0.836350 1.688297 -0.390780
C 0.041297 2.404562 0.498239
C -0.393993 3.698031 0.285061
C -0.057386 4.335581 -0.897644
C 0.705435 3.660451 -1.831154
B 1.197219 0.120155 -0.067885
O -0.173968 -0.606793 -0.205471
P -1.199587 -0.497591 -1.663444
C -2.363053 0.769257 -1.111246
C -3.522290 0.552094 -0.473370
Si -4.553709 2.028655 0.136357
C -6.373337 1.636598 -0.099176
F -0.362571 1.831396 1.647465
F -1.138200 4.329390 1.193461
F -0.470271 5.576030 -1.132768
F 1.022206 4.255385 -2.980145
F 1.844524 1.784306 -2.540825
C 2.301510 -0.574245 -1.057185
C 3.570122 -0.007305 -1.127502
C 4.618541 -0.537440 -1.854523
C 4.425435 -1.715330 -2.556461
C 3.190258 -2.330424 -2.505309
C 2.164235 -1.762853 -1.760585
F 3.820122 1.130196 -0.458729
F 1.012656 -2.452951 -1.761742
F 2.991617 -3.471523 -3.167053
F 5.417682 -2.248633 -3.263764
F 5.806256 0.067388 -1.884735
C 1.734655 -0.174893 1.456492
C 2.335637 0.758563 2.293420
C 2.853449 0.444529 3.541316
C 2.790455 -0.856758 4.003552
C 2.216683 -1.826863 3.201704
C 1.718037 -1.472586 1.959694
F 2.451913 2.042473 1.929826
F 3.410969 1.388241 4.299666
F 3.278698 -1.172604 5.198703
F 2.155089 -3.089390 3.626076
F 1.200604 -2.472322 1.227703
C -2.148455 -1.991384 -1.304160
C -3.321185 -2.038048 -0.656189
Si -4.156524 -3.710937 -0.313692
C -4.335540 -3.883518 1.547736
C -4.038853 -0.819613 -0.145137
C -5.850590 -3.701941 -1.121956
C -3.102186 -5.097100 -1.002093
C -4.084892 3.565458 -0.827261
C -4.164420 2.237879 1.960401
O -1.556691 -0.860472 1.999770
H -2.058979 1.786049 -1.348899
H -5.086286 -0.875449 -0.470101
H -1.679794 -2.907196 -1.653608
H -3.578245 -6.059844 -0.792669
H -2.982496 -5.013180 -2.086008
H -2.107337 -5.110655 -0.547693
H -4.820952 -4.831987 1.797264
H -3.358647 -3.866303 2.039658
H -4.944342 -3.077683 1.968258
H -6.355576 -4.658599 -0.956513
H -6.488352 -2.914304 -0.709996
H -5.770326 -3.544425 -2.201395
H -6.986317 2.489795 0.206569
H -6.600194 1.417148 -1.146714
H -6.678978 0.776005 0.503233
H -4.701444 4.408380 -0.501014
H -3.040575 3.845914 -0.671371

H -4.244197 3.425209 -1.900019
H -4.743902 3.061124 2.389154
H -4.405123 1.329074 2.520208
H -3.103456 2.457687 2.105704
H -1.442833 -0.128906 2.612418
H -1.472917 -1.665093 2.517509
H -4.097565 -0.901620 0.950226
H -0.688201 -0.719108 0.643504

preTS_isomerisation_1,4_(H_2O)$_2$_B
$E_{\omega B97X\text{-}D/6\text{-}311+G(d,p)}$= -3713.42975208

C -2.017883 1.624954 1.297629
C -1.597526 1.271875 0.018907
C -1.811329 2.251573 -0.941748
C -2.329969 3.506398 -0.652767
C -2.685811 3.821322 0.643430
C -2.533940 2.863237 1.631246
B -1.091688 -0.265464 -0.244886
O -0.518645 -0.422752 -1.702060
P 0.977613 0.105346 -2.509748
C 2.214063 -1.041821 -1.864025
C 3.307163 -0.688459 -1.171486
Si 4.745429 -1.914978 -0.971342
C 6.013288 -1.453237 -2.278411
F -1.518081 2.043307 -2.236521
F -2.482345 4.412100 -1.619927
F -3.175701 5.021946 0.937860
F -2.893764 3.138630 2.884708
F -1.941302 0.724617 2.290416
C 0.112891 -0.826196 0.706987
C 0.878221 -0.105060 1.610223
C 1.872033 -0.674983 2.394857
C 2.138486 -2.025908 2.289077
C 1.428267 -2.781795 1.372984
C 0.461363 -2.169121 0.601782
F 0.698261 1.211679 1.784169
F -0.171028 -2.954688 -0.286100
F 1.678553 -4.086842 1.252206
F 3.061242 -2.594752 3.060164
F 2.564273 0.069697 3.258464
C -2.473785 -1.161777 -0.173342
C -3.465060 -0.991478 -1.135376
C -4.687341 -1.639180 -1.116963
C -4.982587 -2.496402 -0.072844
C -4.046992 -2.679037 0.927742
C -2.830038 -2.016127 0.865229
F -3.264054 -0.146152 -2.159626
F -5.580217 -1.438076 -2.086518
F -6.151176 -3.128309 -0.028133
F -4.322502 -3.490520 1.949159
F -2.003894 -2.246625 1.895209
C 1.368587 1.566675 -1.534988
C 2.475917 1.746399 -0.803101
C 3.534253 0.695712 -0.624684
Si 2.756368 3.435316 0.027335
C 3.883751 4.441136 -1.087139
C 1.101430 4.289878 0.243203
C 3.582648 3.158179 1.687391
C 5.517034 -1.710673 0.725721
C 4.142687 -3.666316 -1.247546
O -1.608879 -2.162321 -3.312252
H -1.004509 -1.048271 -2.305951
H 2.110514 -2.062272 -2.227583
H 3.750006 0.600822 0.448543
H 0.674133 2.388812 -1.658640

H 3.738437 4.116658 2.191904
H 2.970202 2.528695 2.337291
H 4.560962 2.681699 1.572079
H 4.093388 5.418658 -0.641934
H 4.839695 3.932891 -1.245546
H 3.420445 4.605131 -2.064320
H 1.234770 5.222437 0.799742
H 0.641991 4.543197 -0.716483
H 0.407738 3.655960 0.802177
H 4.826237 -1.963606 1.532425
H 6.388044 -2.366879 0.817447
H 5.860374 -0.683434 0.881177
H 6.886451 -2.109925 -2.214325
H 5.590560 -1.546378 -3.282846
H 6.360099 -0.423430 -2.150332
H 4.973546 -4.364093 -1.104971
H 3.350533 -3.943244 -0.548265
H 3.766848 -3.806332 -2.265185
H -1.946485 -1.857917 -4.158455
H -1.084844 -2.946538 -3.493095
H 4.476837 1.087623 -1.035284

TS_isomerisation_1,4_$(H_2O)_2$_B
$E_{\omega B97X\text{-}D/6\text{-}311+G(d,p)}$= -3713.40403705

C 1.845174 2.259638 0.813188
C 1.636559 1.224988 -0.088261
C 2.040541 1.520510 -1.386417
C 2.536127 2.747287 -1.788283
C 2.683478 3.758626 -0.854315
C 2.341835 3.506514 0.459071
B 1.106136 -0.296710 0.292299
O 0.576795 -0.356340 1.708587
P -0.889872 0.127901 2.325326
C -2.160778 -1.042141 1.813666
C -3.293774 -0.706368 1.180030
Si -4.714666 -1.959257 1.045464
C -4.080257 -3.693376 1.357785
F 1.956883 0.576815 -2.337199
F 2.878404 2.963859 -3.058658
F 3.155393 4.948980 -1.215333
F 2.489615 4.462250 1.378732
F 1.562945 2.123211 2.127664
C 2.493372 -1.209200 0.226957
C 3.462777 -1.080006 1.212289
C 4.677487 -1.742756 1.221082
C 4.986753 -2.590779 0.174175
C 4.071974 -2.743157 -0.850567
C 2.865204 -2.059575 -0.810251
F 3.262926 -0.234958 2.251710
F 2.060184 -2.274089 -1.861234
F 4.357790 -3.549757 -1.873289
F 6.146863 -3.240817 0.149563
F 5.548101 -1.563427 2.215286
C -0.080580 -0.899592 -0.675189
C -0.413098 -2.245082 -0.552065
C -1.387812 -2.874971 -1.300544
C -2.122139 -2.137434 -2.211594
C -1.865414 -0.786940 -2.341553
C -0.861991 -0.200701 -1.582193
F 0.237575 -3.020719 0.331285
F -1.625447 -4.181633 -1.159775
F -3.055018 -2.723970 -2.959188
F -2.574612 -0.060566 -3.208822
F -0.688364 1.114294 -1.787778
C -1.381642 1.594108 1.408252
C -2.524053 1.742390 0.727548

C -3.574364 0.669946 0.629087
Si -2.842315 3.391149 -0.162291
C -3.637680 3.031971 -1.821999
C -4.011382 4.413909 0.893397
C -1.204298 4.277439 -0.391342
C -5.970701 -1.486052 2.360854
C -5.521628 -1.819046 -0.642235
O 1.281137 -0.139811 4.124247
H 1.492398 -0.275446 3.142966
H -2.014964 -2.057965 2.174398
H -3.852231 0.554766 -0.427593
H -0.679785 2.418439 1.462567
H -3.795021 3.964007 -2.373386
H -3.007060 2.379064 -2.429958
H -4.612476 2.549553 -1.701185
H -4.238194 5.367147 0.405866
H -4.957093 3.888026 1.056369
H -3.569781 4.628867 1.870793
H -1.348476 5.177039 -0.997237
H -0.769274 4.589883 0.562496
H -0.483846 3.633486 -0.903948
H -4.833374 -2.061969 -1.454326
H -6.368634 -2.509143 -0.706801
H -5.905446 -0.808678 -0.812647
H -6.831253 -2.161584 2.329838
H -5.530368 -1.542331 3.360524
H -6.340819 -0.467486 2.209702
H -4.903834 -4.406571 1.254325
H -3.299957 -3.979376 0.648822
H -3.678836 -3.798280 2.369814
H 1.778851 0.616257 4.468360
H 0.279508 0.101332 4.008382
H -4.495663 1.052555 1.093267

4 H_2O_isomerisation_1,4_(H_2O)$_2$_B
$E_{\omega B97X-D/6-311+G(d,p)}$= -3713.44878746

C -2.363933 1.743773 1.097997
C -1.756509 1.354416 -0.091629
C -1.846544 2.292587 -1.109235
C -2.437964 3.539497 -0.955412
C -3.000270 3.885243 0.257245
C -2.966929 2.971007 1.297097
B -1.135707 -0.164631 -0.206125
O -0.528621 -0.410450 -1.592388
P 0.849131 0.036073 -2.193963
C 2.108355 -1.182142 -1.953591
C 3.207225 -0.936034 -1.224716
Si 4.540938 -2.290859 -1.032419
C 5.918865 -1.836863 -2.220070
F -1.350548 2.048937 -2.338913
F -2.472346 4.401474 -1.972668
F -3.571549 5.074690 0.423340
F -3.513246 3.282930 2.472158
F -2.371690 0.898364 2.140949
C 0.043430 -0.525119 0.885045
C 0.644175 0.341562 1.787784
C 1.599241 -0.058295 2.713331
C 2.026628 -1.371914 2.733388
C 1.495552 -2.265327 1.818892
C 0.533161 -1.827395 0.928613
F 0.333465 1.646232 1.815222
F 0.057635 -2.746027 0.075342
F 1.907889 -3.533469 1.813949
F 2.942041 -1.770195 3.610789
F 2.107911 0.814105 3.584739

C -2.440275 -1.168742 -0.127537
C -3.348398 -1.189874 -1.182297
C -4.493595 -1.968038 -1.202255
C -4.789722 -2.766918 -0.113064
C -3.934007 -2.763669 0.972276
C -2.793997 -1.974058 0.948420
F -3.149378 -0.416266 -2.262777
F -5.314641 -1.946202 -2.253238
F -5.885246 -3.520846 -0.107004
F -4.211678 -3.520484 2.034626
F -2.035881 -2.030848 2.053606
C 1.486135 1.544272 -1.528953
C 2.629146 1.586118 -0.829122
C 3.494497 0.386843 -0.568007
Si 3.220921 3.269296 -0.142894
C 4.604376 3.847975 -1.267217
C 1.771987 4.453803 -0.162194
C 3.857506 2.998843 1.595892
C 5.171528 -2.250038 0.731256
C 3.788546 -3.949384 -1.452939
O -0.926248 -2.799826 -3.163092
H -0.998097 -2.098255 -2.504113
H 1.914815 -2.153014 -2.400116
H 3.507225 0.219389 0.519296
H 0.894324 2.434971 -1.700096
H 4.261160 3.936362 1.990149
H 3.061896 2.668221 2.267070
H 4.660163 2.255930 1.622551
H 4.996621 4.808997 -0.921140
H 5.433015 3.133801 -1.278585
H 4.249165 3.975726 -2.293443
H 2.058883 5.394189 0.317602
H 1.448281 4.689575 -1.179934
H 0.919714 4.041775 0.385765
H 4.399844 -2.540074 1.447878
H 6.002289 -2.953827 0.839723
H 5.541652 -1.257976 1.005819
H 6.730098 -2.568319 -2.155527
H 5.557501 -1.820707 -3.252004
H 6.337730 -0.852929 -1.989155
H 4.526958 -4.739567 -1.287574
H 2.920276 -4.163529 -0.824155
H 3.477510 -4.002501 -2.499946
H -1.601517 -2.592200 -3.811816
H 0.575706 0.173174 -3.558721
H 4.535364 0.662612 -0.786308

3.5.5 Deprotonation

tBuO^{-}
$E_{\omega B97X\text{-}D/6\text{-}311+G(d,p)}$= -233.1524523

C	0.051282	0.425816	-3.340025
C	0.289536	0.224473	-1.821676
C	1.431850	-0.812047	-1.665726
C	0.800675	1.570328	-1.247410
O	-0.849207	-0.187250	-1.187077
H	0.951521	0.755761	-3.876164
H	-0.289157	-0.515299	-3.785790
H	-0.733327	1.174857	-3.492451
H	2.361751	-0.504557	-2.162839
H	1.116680	-1.771599	-2.089846
H	1.645443	-0.966450	-0.602392
H	1.714319	1.928983	-1.740211
H	0.024426	2.335308	-1.361622
H	1.008126	1.457262	-0.177488

HO⁻
$E_{\omega B97X\text{-}D/6\text{-}311+G(d,p)}$= -75.9149776

O	-1.573761	-0.966870	-1.725082
H	-2.487245	-1.220289	-1.576016

tBuOH
$E_{\omega B97X\text{-}D/6\text{-}311+G(d,p)}$= -233.6810006

O	-0.787639	-0.418386	-1.305099
C	0.364414	0.173250	-1.926922
C	0.129280	0.286256	-3.433371
C	1.516798	-0.777328	-1.628533
C	0.623320	1.550187	-1.314914
H	1.007624	0.704098	-3.932703
H	-0.078306	-0.698078	-3.860340
H	-0.721317	0.942327	-3.644542
H	2.443960	-0.401721	-2.068225
H	1.311755	-1.767006	-2.044364
H	1.659706	-0.875791	-0.549500
H	1.516729	2.006275	-1.749906
H	-0.221342	2.221452	-1.500534
H	0.766898	1.464832	-0.234911
H	-1.544235	0.149004	-1.469993

[(tBuO)→B(C_6F_5)$_3$]⁻
$E_{\omega B97X\text{-}D/6\text{-}311+G(d,p)}$= -2441.5343464

C	-2.646164	2.066653	-0.716187
C	-2.292018	1.170902	0.284114
C	-2.867764	1.459024	1.518181
C	-3.677890	2.549818	1.774016
C	-3.975480	3.426954	0.746107
C	-3.456785	3.176946	-0.507600
B	-1.442014	-0.260089	0.056088
O	-0.861369	-0.437764	-1.263967
C	0.354144	0.054859	-1.810684
F	-2.652094	0.638852	2.563753
F	-4.180565	2.763629	2.995000
F	-4.755685	4.487499	0.962244
F	-3.746588	4.004644	-1.518355
F	-2.228660	1.917305	-1.983182
C	-0.284206	-0.523060	1.224382
C	0.271437	-1.792722	1.346122
C	1.288826	-2.116501	2.224897
C	1.821948	-1.132687	3.039519
C	1.321583	0.150500	2.948082
C	0.295414	0.424528	2.053495
F	-0.172721	-2.801342	0.576828
F	-0.096489	1.713086	2.020376
F	1.833392	1.117873	3.716739
F	2.807182	-1.418807	3.891602
F	1.767859	-3.363213	2.293491
C	-2.692170	-1.375556	0.166899
C	-3.008564	-2.171913	1.261570
C	-4.087786	-3.043524	1.303312
C	-4.929444	-3.145241	0.213940
C	-4.675697	-2.359772	-0.894590
C	-3.588447	-1.499494	-0.892913
F	-2.267095	-2.151894	2.384290
F	-4.320428	-3.787533	2.390598
F	-5.972514	-3.976937	0.234448
F	-5.493505	-2.426052	-1.951548
F	-3.466300	-0.739914	-1.991730

C	0.108828	0.145900	-3.320454
C	1.495139	-0.944251	-1.561328
C	0.771933	1.433205	-1.283624
H	1.010857	0.476147	-3.844680
H	-0.178826	-0.834758	-3.708844
H	-0.697709	0.850064	-3.529034
H	2.367283	-0.682934	-2.168524
H	1.173420	-1.953178	-1.830721
H	1.807732	-0.946449	-0.515446
H	1.690864	1.747362	-1.787533
H	0.006978	2.184081	-1.479463
H	0.976572	1.411551	-0.213423

$HMDS^{-}$

$E_{\omega B97X\text{-}D/6\text{-}311+G(d,p)}$= -873.4346271

N	6.206074	17.011902	3.689573
Si	6.020046	17.243803	5.335145
Si	6.547806	15.636970	2.800961
C	7.268298	16.301694	6.429180
C	4.319189	16.720495	6.016924
C	6.204282	19.069328	5.825119
C	8.296905	14.928200	3.074523
C	6.431323	15.953634	0.932470
C	5.392059	14.155958	3.134469
H	7.136405	16.529737	7.493836
H	7.152735	15.218647	6.302588
H	8.298470	16.554106	6.153419
H	7.202640	19.442953	5.571146
H	5.474364	19.690343	5.293649
H	6.052369	19.218151	6.900799
H	4.238192	16.886822	7.098116
H	3.513842	17.281787	5.529601
H	4.140553	15.656076	5.825195
H	5.455348	13.846627	4.184616
H	4.347905	14.422323	2.934341
H	5.643532	13.287027	2.514220
H	6.669867	15.053938	0.352852
H	7.128792	16.742878	0.629784
H	5.423151	16.277837	0.651243
H	8.476631	14.031354	2.468980
H	9.063293	15.667681	2.815606
H	8.441764	14.659444	4.126941

HMDS_H (protonated)

$E_{\omega B97X\text{-}D/6\text{-}311+G(d,p)}$= - 873.9671429

C	7.163265	16.142127	6.389794
Si	5.999533	17.235682	5.400566
C	6.285734	19.041935	5.815751
N	6.309697	17.022726	3.698173
Si	6.635809	15.574423	2.784036
C	5.504746	14.195228	3.373314
C	4.233760	16.766767	5.841186
C	8.415181	15.010303	3.002094
C	6.320207	15.952883	0.974612
H	6.980061	16.258525	7.462743
H	7.026646	15.083641	6.146347
H	8.207785	16.400173	6.191766
H	7.321074	19.333885	5.616592
H	5.630406	19.689379	5.224364
H	6.076841	19.234174	6.872653
H	4.044985	16.899419	6.911649
H	3.517367	17.384991	5.291253
H	4.034350	15.720171	5.591195
H	5.658514	13.972923	4.434103

H	4.453263	14.463936	3.235485
H	5.698352	13.274618	2.813558
H	6.539325	15.079611	0.352489
H	6.954936	16.774992	0.628744
H	5.276840	16.235467	0.806220
H	8.614113	14.098928	2.428573
H	9.113311	15.782665	2.664577
H	8.631209	14.800073	4.054130
H	6.299071	17.875335	3.154334

7

$E_{\omega B97X\text{-}D/6\text{-}311+G(d,p)}$= -1428.171185

C	7.201110	17.575960	4.567641
Si	6.318668	17.075257	6.151265
C	6.778585	18.284399	7.520465
C	4.448849	17.067010	5.910044
C	3.652397	16.825582	7.051497
C	2.248225	16.907778	7.042745
C	1.505886	17.161213	5.890115
P	2.211939	17.082759	4.287180
C	3.915900	17.321642	4.652825
O	1.849055	15.885070	3.409013
C	6.890183	15.353918	6.656957
Si	1.286900	16.702698	8.651602
C	0.239903	18.237180	8.959424
C	0.146965	15.210425	8.515967
C	2.450059	16.454043	10.110102
H	4.577193	17.542307	3.817918
H	4.152552	16.636253	7.997838
H	0.422803	17.257603	5.953699
H	6.895694	18.573599	4.238053
H	6.993339	16.873389	3.755180
H	8.283775	17.591645	4.729222
H	6.403813	15.040847	7.585918
H	7.973293	15.327807	6.816046
H	6.641030	14.619496	5.885119
H	6.467414	19.301496	7.263206
H	7.860069	18.291859	7.691761
H	6.289269	18.013359	8.460888
H	-0.443180	15.078314	9.428809
H	0.723793	14.295980	8.347388
H	-0.545855	15.326227	7.677126
H	3.062702	15.556292	9.984071
H	1.875660	16.341657	11.035342
H	3.122893	17.308051	10.233219
H	-0.360764	18.129007	9.868433
H	-0.441800	18.419716	8.123269
H	0.874516	19.121369	9.073118
H	1.767537	18.223099	3.559440

11

$E_{\omega B97X\text{-}D/6\text{-}311+G(d,p)}$= -1428.161228

C	7.192343	17.177540	4.448095
Si	6.320156	16.993268	6.101144
C	6.995734	18.264183	7.317046
C	4.470338	17.240920	5.898151
C	3.612291	17.110109	7.099128
C	2.295678	16.829018	7.075651
C	1.590622	16.624867	5.743222
P	2.101832	17.924346	4.506831
C	3.896596	17.531686	4.710979
O	1.667046	17.416423	3.089909
C	6.636729	15.274248	6.801930
Si	1.302130	16.623603	8.650955

C	-0.057253	17.924534	8.695280
C	0.508034	14.916914	8.645259
C	2.399412	16.809448	10.166354
H	4.517408	17.649671	3.820758
H	4.107221	17.215426	8.064554
H	0.502108	16.652313	5.855337
H	1.846738	15.632686	5.335729
H	7.045892	18.177508	4.029165
H	6.823759	16.448846	3.720095
H	8.268456	17.017920	4.569207
H	6.113771	15.140370	7.754319
H	7.704396	15.108457	6.978324
H	6.280667	14.501634	6.113761
H	6.836733	19.281615	6.947368
H	8.070177	18.123053	7.473495
H	6.503184	18.180647	8.290954
H	-0.134870	14.779543	9.520571
H	1.269242	14.130916	8.655142
H	-0.107529	14.776772	7.751324
H	3.193664	16.056961	10.179171
H	1.806846	16.687584	11.078601
H	2.867263	17.797935	10.201403
H	-0.698100	17.794406	9.573402
H	-0.689848	17.861163	7.804601
H	0.370092	18.931071	8.730123

TS_**7**_to_**11**

$E_{\omega B97X\text{-}D/6\text{-}311+G(d,p)}$= -1428.080567

C	4.353189	0.139456	-0.441999
Si	2.823878	-0.852192	0.013257
C	2.983695	-1.476904	1.781618
C	1.270302	0.207108	-0.125526
C	0.001071	-0.455259	0.086492
C	-1.220557	0.128920	-0.131769
Si	-2.811708	-0.860449	0.035365
C	-3.763333	-0.771882	-1.586635
C	1.347948	1.523872	-0.473172
P	-0.019622	2.721606	-0.277417
O	0.105354	3.392971	1.108578
C	-1.306819	1.510698	-0.529157
C	2.670909	-2.334266	-1.138715
C	-3.869687	-0.113360	1.400193
C	-2.443390	-2.658963	0.445753
H	-0.646539	2.394220	-1.654555
H	3.866672	-2.114232	1.895557
H	3.073446	-0.641484	2.482253
H	2.105773	-2.063128	2.070263
H	4.294783	0.517252	-1.467135
H	4.488018	0.994580	0.226795
H	5.245145	-0.490580	-0.366029
H	2.582845	-2.010753	-2.180349
H	3.546170	-2.986910	-1.057617
H	1.784633	-2.929246	-0.897373
H	2.328334	1.953160	-0.676345
H	-1.834442	-3.129172	-0.332352
H	-1.908694	-2.750202	1.396058
H	-3.376242	-3.225456	0.530928
H	-3.190062	-1.226324	-2.400348
H	-4.720556	-1.297524	-1.508781
H	-3.971000	0.266392	-1.862794
H	-3.353931	-0.163591	2.363876
H	-4.823383	-0.642646	1.495117
H	-4.085404	0.939034	1.192280
H	0.031306	-1.505178	0.372255
H	-2.295724	1.924370	-0.732291

preTS_from_**7**_to_**8**

$E_{\omega B97X\text{-}D/6\text{-}311+G(d,p)}$= -1661.8685054

O	-1.573693	-1.768452	-0.072389
P	-0.559823	-1.127697	0.888572
C	1.108535	-1.634481	0.728126
C	2.080221	-0.815071	0.160326
Si	3.753441	-1.600449	-0.217036
C	3.537342	-2.952519	-1.508688
C	-0.400575	0.615208	0.831566
C	0.698217	1.240144	0.248748
C	1.874362	0.552717	-0.110113
Si	0.569508	3.100126	-0.034382
C	-0.825572	3.444452	-1.250202
C	0.173854	3.959804	1.593271
C	2.178248	3.791760	-0.721962
C	4.968226	-0.318459	-0.866116
C	4.454041	-2.370055	1.352204
H	-1.263651	1.206822	1.131794
H	2.687519	1.118827	-0.552392
H	1.337474	-2.674657	0.953700
H	-1.017746	-1.490887	2.184061
H	-3.251947	-1.981240	0.161501
H	4.490286	-3.444285	-1.729922
H	2.834915	-3.714785	-1.158138
H	3.141905	-2.539252	-2.441478
H	5.937128	-0.786352	-1.068306
H	4.613004	0.133281	-1.797197
H	5.126799	0.484824	-0.140398
H	5.415902	-2.854753	1.155370
H	4.607163	-1.609339	2.123755
H	3.773015	-3.124784	1.756982
H	3.013465	3.612523	-0.038263
H	2.427996	3.337942	-1.685706
H	2.091436	4.872709	-0.872042
H	0.062379	5.039527	1.449682
H	-0.758720	3.578733	2.020377
H	0.969605	3.793512	2.325722
H	-0.950383	4.518967	-1.419327
H	-0.627019	2.967574	-2.214779
H	-1.772481	3.047121	-0.871663
O	-4.224408	-2.096332	0.272656
C	-4.873820	-0.942993	-0.250812
C	-6.371863	-1.188127	-0.100954
C	-4.503606	-0.766692	-1.726715
C	-4.452538	0.296477	0.545838
H	-4.992440	0.114758	-2.153305
H	-3.421339	-0.649400	-1.826504
H	-4.810433	-1.646042	-2.300391
H	-6.631776	-1.328214	0.952242
H	-6.944707	-0.341069	-0.489135
H	-6.665262	-2.086523	-0.651228
H	-4.691522	0.164702	1.605081
H	-3.375119	0.452509	0.449956
H	-4.965022	1.192522	0.181980

TS_from_**7**_to_**8**

$E_{\omega B97X\text{-}D/6\text{-}311+G(d,p)}$= -1661.8169037

O	-1.562730	-0.960329	-1.730539
P	-1.000005	-0.723664	-0.155957
C	0.480271	-1.612753	0.088814
C	1.737117	-1.015614	0.021355
Si	3.237854	-2.158255	0.112051
C	3.215205	-3.336241	-1.354700
C	-0.471575	0.925487	0.052871
C	0.866468	1.304442	-0.011515

C	1.922839	0.376229	-0.069436
Si	1.239671	3.154657	0.025370
C	0.466946	3.971865	-1.482646
C	0.489450	3.909770	1.577381
C	3.094841	3.463481	0.017330
C	4.835262	-1.165651	0.085642
C	3.158021	-3.162527	1.701754
H	-1.248799	1.681107	0.146979
H	2.938080	0.756612	-0.113837
H	0.395261	-2.689848	0.214917
H	-2.395956	-1.164711	0.354125
H	-2.496506	-1.218403	-1.579680
H	4.062259	-4.028949	-1.317381
H	2.294116	-3.926758	-1.364341
H	3.267237	-2.784284	-2.298071
H	5.696707	-1.838865	0.141938
H	4.926923	-0.579993	-0.834023
H	4.891596	-0.475622	0.932863
H	4.007351	-3.849559	1.774725
H	3.173664	-2.506596	2.577414
H	2.239783	-3.755947	1.744687
H	3.580038	3.006939	0.885216
H	3.563235	3.057279	-0.884238
H	3.297358	4.538958	0.045072
H	0.662966	4.990248	1.611188
H	-0.591075	3.740556	1.611713
H	0.926280	3.465828	2.476934
H	0.645484	5.052008	-1.483347
H	0.882620	3.556342	-2.405596
H	-0.614615	3.806505	-1.500040
O	-3.705859	-1.573828	-0.088574
C	-4.750051	-0.703458	0.217217
C	-5.382019	-1.089430	1.566498
C	-5.823062	-0.783875	-0.881361
C	-4.246779	0.753073	0.306894
H	-6.676480	-0.127923	-0.672594
H	-5.392472	-0.493454	-1.845133
H	-6.189763	-1.811371	-0.968385
H	-4.627495	-1.038813	2.358141
H	-6.213017	-0.428160	1.839338
H	-5.757892	-2.116315	1.520316
H	-3.510112	0.856420	1.110753
H	-3.765830	1.042824	-0.633268
H	-5.065112	1.453736	0.505391

8_tBuOH

$E_{\omega B97X\text{-}D/6\text{-}311+G(d,p)}$= -1661.8492403

H	-3.637530	-1.129219	2.483946
H	-3.665962	0.199054	-1.032507
H	-5.171432	-0.608310	-1.506146
H	-5.213873	0.763209	-0.378628
H	-3.637530	-1.129219	2.483946
H	-3.665962	0.199054	-1.032507
H	-5.171432	-0.608310	-1.506146
H	-5.213873	0.763209	-0.378628

8

$E_{\omega B97X\text{-}D/6\text{-}311+G(d,p)}$= -1428.1557089

C	3.169468	-3.377165	-1.309059
Si	3.211822	-2.158124	0.127056
C	3.177601	-3.138050	1.736060
C	1.718036	-1.016218	0.041817
C	1.904947	0.373889	-0.092972

C	0.845123	1.297704	0.001047
Si	1.211974	3.143465	0.035890
C	3.065316	3.470409	-0.037494
C	0.457723	-1.596709	0.153271
P	-1.077768	-0.751839	-0.117751
C	-0.485882	0.904507	0.108707
O	-1.253405	-0.850031	-1.880846
C	4.820289	-1.181960	0.042857
C	0.391006	3.988517	-1.434537
C	0.521612	3.903198	1.615927
H	-1.247655	1.670454	0.258350
H	2.918705	0.754885	-0.174070
H	0.389769	-2.669842	0.335290
H	-2.134001	-1.184853	-2.065949
H	4.021565	-4.064054	-1.273341
H	2.251628	-3.972540	-1.282674
H	3.195905	-2.849506	-2.267498
H	5.678444	-1.859938	0.094841
H	4.894670	-0.617560	-0.891740
H	4.900586	-0.472122	0.871710
H	4.013636	-3.843406	1.787365
H	3.241986	-2.469276	2.599851
H	2.247864	-3.708365	1.824468
H	3.585028	3.015462	0.811175
H	3.504264	3.068072	-0.955643
H	3.261540	4.547347	-0.015429
H	0.683163	4.986041	1.637570
H	-0.554110	3.719870	1.697820
H	1.001992	3.468905	2.498064
H	0.576207	5.067783	-1.427551
H	0.770485	3.582843	-2.377481
H	-0.691544	3.829336	-1.415970

TS_dissoc_**8**

$E_{\omega B97X\text{-}D/6\text{-}311+G(d,p)}$= -1428.1335124

C	3.918763	0.061227	1.305798
Si	2.850170	-0.880528	0.082661
C	3.758101	-1.024470	-1.557407
C	1.242434	0.076663	-0.202746
C	0.000000	-0.529452	0.024403
C	-1.242434	0.076663	-0.202745
Si	-2.850170	-0.880528	0.082661
C	-2.470653	-2.594723	0.750686
C	1.327649	1.392537	-0.663695
P	-0.000000	2.463775	-0.959964
C	-1.327649	1.392536	-0.663695
O	0.000000	3.953398	1.650956
C	2.470652	-2.594729	0.750671
C	-3.918769	0.061235	1.305786
C	-3.758093	-1.024483	-1.557409
H	-2.316876	1.820097	-0.822993
H	0.000000	-1.552723	0.390030
H	2.316876	1.820098	-0.822993
H	-0.000000	4.724335	1.079190
H	4.866685	-0.459713	1.474288
H	4.146899	1.065816	0.937789
H	3.410168	0.166789	2.268275
H	3.402081	-3.142841	0.923671
H	1.931220	-2.543167	1.701176
H	1.866441	-3.176308	0.047854
H	4.710211	-1.549829	-1.432019
H	3.159003	-1.574788	-2.289044
H	3.971759	-0.035385	-1.973905
H	-1.866441	-3.176309	0.047874

H	-1.931222	-2.543154	1.701190
H	-3.402082	-3.142835	0.923689
H	-4.710204	-1.549844	-1.432022
H	-3.971753	-0.035401	-1.973915
H	-3.158992	-1.574804	-2.289041
H	-4.866691	-0.459705	1.474276
H	-3.410179	0.166805	2.268265
H	-4.146904	1.065821	0.937769

3.5.6 Hydrophosphination reaction

$P^{t}Bu_{3}$:
$E_{\omega B97X\text{-}D/6\text{-}31+G(d)}$= -814.753034
$G_{\omega B97X\text{-}D/6\text{-}31+G(d)}$= -814.419132
$E_{\omega B97X\text{-}D/6\text{-}311+G(d,p)}$=-814.8918427

P	-0.020277	-0.035512	-0.014080
C	-0.039246	0.051726	1.904287
C	1.816189	-0.009601	-0.575293
C	-0.845794	1.569296	-0.670713
C	-1.441602	-0.396098	2.367326
C	0.926771	-1.002540	2.473695
C	0.294374	1.404727	2.548508
H	0.748848	-1.088468	3.551202
H	0.757560	-1.989117	2.033709
H	1.975057	-0.735429	2.342755
H	-1.444745	-0.474190	3.460610
H	-2.232207	0.297594	2.089895
H	-1.694133	-1.378948	1.960099
H	0.305085	1.289280	3.639013
H	1.273706	1.777863	2.246824
H	-0.445952	2.170694	2.315259
C	1.884883	0.303683	-2.080609
C	2.761342	0.952860	0.157780
C	2.355903	-1.448960	-0.439114
C	-1.223486	1.318023	-2.145796
C	-2.185346	1.787741	0.055042
C	-0.031675	2.867337	-0.574414
H	-2.738346	2.575017	-0.468844
H	-2.805836	0.887506	0.043014
H	-2.062753	2.115025	1.087180
H	-1.807313	2.170575	-2.511424
H	-0.362587	1.209894	-2.801877
H	-1.841040	0.421236	-2.245559
H	-0.647109	3.703229	-0.928181
H	0.272469	3.091749	0.448564
H	0.863959	2.843852	-1.196027
H	3.362853	-1.491165	-0.869972
H	2.430271	-1.788744	0.591508
H	1.727358	-2.157406	-0.985571
H	3.753662	0.903157	-0.306409
H	2.423554	1.988370	0.103542
H	2.886769	0.692705	1.209298
H	2.905401	0.109453	-2.428245
H	1.213710	-0.336063	-2.660104
H	1.661156	1.345167	-2.310105

$[HP^{t}Bu_{3}]^{+}$:
$E_{\omega B97X\text{-}D/6\text{-}31+G(d)}$= -815.2026695
$G_{\omega B97X\text{-}D/6\text{-}31+G(d)}$= -814.857098
$E_{\omega B97X\text{-}D/6\text{-}311+G(d,p)}$= -815.34237

C	-1.275599	0.334318	2.568758
C	0.167060	0.314398	2.028728
C	0.910151	1.541000	2.589055
P	0.129846	0.247235	0.140972
C	-1.043431	1.503753	-0.653320

C	-0.815163	2.915461	-0.094375
C	1.847160	-0.068987	-0.613989
C	2.051573	-1.599112	-0.573994
C	0.835725	-0.975829	2.542755
C	1.952982	0.368382	-2.083559
C	2.968975	0.643387	0.159216
C	-0.895613	1.536652	-2.185619
C	-2.487944	1.032249	-0.374442
H	0.755058	-0.978908	3.634890
H	0.337348	-1.877969	2.173265
H	1.898710	-1.032495	2.303380
H	-1.222423	0.177750	3.651330
H	-1.764167	1.298164	2.411991
H	-1.898477	-0.463039	2.152619
H	0.781430	1.531954	3.676920
H	1.980737	1.521576	2.389629
H	0.508158	2.486663	2.222179
H	-3.163079	1.732555	-0.877627
H	-2.676510	0.037071	-0.789758
H	-2.759818	1.028412	0.678509
H	-1.738617	2.116682	-2.575620
H	0.015517	2.033736	-2.514032
H	-0.953641	0.542119	-2.637329
H	-1.438135	3.613374	-0.664093
H	-1.111781	3.000723	0.953653
H	0.224722	3.241340	-0.198514
H	3.059569	-1.812355	-0.945786
H	1.976499	-2.027291	0.426557
H	1.339438	-2.114955	-1.226243
H	3.905120	0.465181	-0.380355
H	2.821307	1.726376	0.206542
H	3.107199	0.255414	1.169364
H	2.920390	0.011295	-2.453112
H	1.183007	-0.071985	-2.718978
H	1.944995	1.454569	-2.193606
H	-0.535077	-0.961559	-0.112602

TS_deprotonation_P_**5**_P^{t}Bu$_3$:
$E_{\omega B97X\text{-}D/6\text{-}31+G(d)}$= -2243.2049157
$G_{\omega B97X\text{-}D/6\text{-}31+G(d)}$= -2242.58951
$E_{\omega B97X\text{-}D/6\text{-}311+G(d,p)}$= -2243.5389977

P	0.34014700	0.15494200	2.20099100
Si	2.59249300	-2.95463200	-0.28192600
Si	3.07304800	2.65871000	-0.33791800
O	0.04195500	0.05625800	3.71941600
C	1.16369400	-1.35911600	1.58987700
H	0.87223800	-2.30184300	2.05715500
C	1.95697000	-1.35836300	0.49410600
C	2.36526000	-0.07339400	-0.12064200
H	2.79051100	-0.13828400	-1.12354100
C	2.32058900	1.13709700	0.47643800
C	1.77537600	1.28471300	1.89112100
H	1.46184200	2.31543200	2.09350000
H	2.56925800	1.05184200	2.62201800
C	1.95598500	-4.43559300	0.68893800
H	0.85972000	-4.47015200	0.68997200
H	2.29545400	-4.41363100	1.73105200
H	2.31937700	-5.36684600	0.23782500
C	4.47776600	-2.92428200	-0.28626600
H	4.85558700	-2.05493400	-0.83832500
H	4.88592400	-3.82548400	-0.75986200
H	4.87157300	-2.86853800	0.73544900
C	1.98428900	-3.06651300	-2.06689900
H	0.89870900	-3.21401900	-2.11006400
H	2.45729100	-3.91325000	-2.57958700
H	2.22135500	-2.15866400	-2.63486200

C	4.51275800	3.22836800	0.73894100
H	4.97419700	4.13566600	0.33029400
H	5.28547900	2.45347100	0.80631900
H	4.17438700	3.45430100	1.75750500
C	3.67212100	2.24682100	-2.07573400
H	4.46127900	1.48588800	-2.06447700
H	4.08057500	3.14460800	-2.55546500
H	2.85238200	1.87684200	-2.70356600
C	1.80647900	4.05575300	-0.42818500
H	2.29991000	4.99120300	-0.71991600
H	1.31945500	4.22639200	0.53937900
H	1.02464800	3.84638500	-1.16634400
H	-1.12530400	0.22648600	0.92641500
P	-2.34434600	0.11781200	-0.12408500
C	-3.61268400	1.39872100	0.44902100
C	-3.03100500	-1.64663000	-0.04235500
C	-1.63668000	0.52430200	-1.83133300
C	-3.10905800	2.81607900	0.11719500
C	-3.69353000	1.32737900	1.99026800
C	-5.00788900	1.21686200	-0.16761300
C	-2.69715600	0.84675700	-2.89430700
C	-0.67240100	1.71914900	-1.67317200
C	-0.76903000	-0.65225200	-2.31495100
C	-3.91646700	-2.01543000	-1.24282000
C	-1.84366200	-2.62824400	0.06058900
C	-3.84169400	-1.84308600	1.25294700
H	-2.24753400	-3.64225500	0.16747200
H	-1.19166100	-2.62828900	-0.81146500
H	-1.22995300	-2.41999200	0.94095600
H	-4.32707800	-3.01958200	-1.07982500
H	-3.35699700	-2.04411300	-2.18084200
H	-4.75889000	-1.32889900	-1.36607600
H	-4.09689800	-2.90648100	1.32920400
H	-3.26363000	-1.58165800	2.14467000
H	-4.78162100	-1.28836700	1.26002000
H	-5.65681000	2.02710600	0.18735700
H	-4.98706600	1.26360600	-1.26011700
H	-5.47758900	0.27496700	0.12445700
H	-4.31084600	2.16366100	2.33926100
H	-4.15442200	0.41260800	2.35964500
H	-2.70605200	1.42610300	2.45344400
H	-3.78065100	3.53342400	0.60297900
H	-2.10060000	2.99861400	0.50221900
H	-3.12965700	3.03346000	-0.95247600
H	-2.18972700	1.02788300	-3.85012800
H	-3.40509200	0.02751900	-3.04461800
H	-3.26445800	1.74947300	-2.65368900
H	-0.09186600	1.81774400	-2.59868700
H	-1.18170000	2.66807200	-1.51260300
H	0.03920100	1.55915700	-0.85823300
H	-0.24717500	-0.33785800	-3.22631600
H	-1.35190200	-1.53926100	-2.57122000
H	-0.00560800	-0.92257700	-1.57834400

IonPair_deprotonation_**5**_P^{t}Bu$_3$:
$E_{\omega B97X\text{-}D/6\text{-}31+G(d)}$= -2243.2088009
$G_{\omega B97X\text{-}D/6\text{-}31+G(d)}$= -2242.590316
$E_{\omega B97X\text{-}D/6\text{-}311+G(d,p)}$= -2243.5427795

P	-0.62124800	-0.26754700	2.39346500
Si	-2.27489500	3.05556500	-0.33317800
Si	-3.20218200	-2.49933500	-0.46830400
O	-0.59596000	-0.09133400	3.94946300
C	-1.16429200	1.32653100	1.63995900
H	-0.82629800	2.23732800	2.14133100
C	-1.82414800	1.41007300	0.46071800
C	-2.28287500	0.16248300	-0.19547400
H	-2.52366400	0.23448700	-1.25804000

C	-2.47529900	-1.01855000	0.43102500
C	-2.20037300	-1.14388300	1.92430000
H	-2.12891100	-2.19394200	2.23219400
H	-3.03936200	-0.70488900	2.49352200
C	-1.51902300	4.47710100	0.64348100
H	-0.42506700	4.40683800	0.67524300
H	-1.88605200	4.49767900	1.67620200
H	-1.77951000	5.43497000	0.17689000
C	-4.15500200	3.21126400	-0.36118100
H	-4.60771300	2.37913100	-0.91452000
H	-4.46900400	4.14551400	-0.84278500
H	-4.56401900	3.19738100	0.65596500
C	-1.66009300	3.13204600	-2.12004600
H	-0.56866600	3.21725900	-2.17428100
H	-2.08557500	4.00814100	-2.62527500
H	-1.95625700	2.24414900	-2.69199800
C	-4.84567500	-2.93631100	0.34806500
H	-5.28971800	-3.82726300	-0.11268300
H	-5.56269600	-2.11186900	0.25626900
H	-4.70882600	-3.14411100	1.41623700
C	-3.46832800	-2.10281700	-2.29204100
H	-4.18623600	-1.28508000	-2.42657000
H	-3.86079000	-2.98219900	-2.81714400
H	-2.53063900	-1.81297000	-2.78147000
C	-2.04744100	-3.98550600	-0.31145600
H	-2.53107200	-4.88702200	-0.70783600
H	-1.78739400	-4.18068700	0.73581000
H	-1.11444000	-3.83491300	-0.86588500
H	1.30831800	-0.24320300	0.76309400
P	2.41077700	-0.20679900	-0.13877200
C	3.57805800	-1.51530200	0.54606800
C	3.09155400	1.54782400	-0.03551900
C	1.66643100	-0.63949300	-1.81289000
C	3.03282600	-2.92247300	0.23891100
C	3.60078100	-1.38342100	2.08486700
C	4.99540100	-1.39151700	-0.03428400
C	2.73428100	-1.02976000	-2.84574700
C	0.66057500	-1.79285900	-1.61509900
C	0.84988800	0.55553900	-2.33649200
C	4.01849000	1.87072100	-1.21833400
C	1.90857500	2.53741900	0.00956100
C	3.86529300	1.74546000	1.28158400
H	2.31597800	3.53761000	0.19531600
H	1.34349000	2.58594600	-0.91929700
H	1.21391600	2.30144200	0.82036300
H	4.42512400	2.87742400	-1.06981800
H	3.49208100	1.87310300	-2.17554300
H	4.86432600	1.18063700	-1.28936000
H	4.14588700	2.80268000	1.34099900
H	3.25186700	1.52004200	2.15871500
H	4.78964000	1.16757400	1.32809600
H	5.60666200	-2.20224400	0.37821100
H	5.00999500	-1.48919700	-1.12377200
H	5.48200800	-0.45117400	0.23396300
H	4.13836500	-2.24967600	2.48591900
H	4.11920600	-0.49352700	2.43711300
H	2.59096100	-1.39108800	2.50865900
H	3.67329400	-3.64324300	0.75886400
H	2.01338500	-3.06193800	0.61091500
H	3.07005100	-3.17076700	-0.82326400
H	2.23125100	-1.22701200	-3.79934100
H	3.46628600	-0.23579500	-3.01684100
H	3.27091100	-1.94041000	-2.56854600
H	0.09211400	-1.90306000	-2.54575400
H	1.13356400	-2.75241700	-1.41437800
H	-0.05474000	-1.57393300	-0.81652600
H	0.31945200	0.22828100	-3.23747500

H	1.46930900	1.40748500	-2.62306800
H	0.09540200	0.87976600	-1.61362000

vdW_deprotonation_**5**_P^{t}Bu$_3$:
$E_{\omega B97X\text{-}D/6\text{-}31+G(d)}$= -2243.2405629
$G_{\omega B97X\text{-}D/6\text{-}31+G(d)}$= -2242.621841
$E_{\omega B97X\text{-}D/6\text{-}311+G(d,p)}$= -2243.5740656

P	-1.10049900	-0.17851600	2.52635300
Si	-2.18996500	3.07955300	-0.45364100
Si	-3.37743800	-2.43924900	-0.56136800
O	-0.95912900	-0.07275400	4.02613400
C	-1.30767900	1.38808000	1.65677800
H	-0.93421500	2.28349000	2.15017600
C	-1.89110500	1.42988500	0.44123900
C	-2.37802200	0.19142200	-0.21142900
H	-2.53320000	0.25971200	-1.28753100
C	-2.67742700	-0.97213400	0.40159300
C	-2.52720000	-1.11539700	1.90951900
H	-2.42382900	-2.16077300	2.21816700
H	-3.42308400	-0.72392000	2.41465000
C	-1.35881400	4.46897700	0.49913900
H	-0.27835800	4.30470200	0.58681400
H	-1.77189600	4.57475900	1.50886600
H	-1.51066500	5.42091300	-0.02355400
C	-4.05542700	3.32555200	-0.52223900
H	-4.54151500	2.50173700	-1.05868800
H	-4.30585000	4.25847300	-1.04142500
H	-4.48418400	3.37115200	0.48552000
C	-1.51259700	2.99640700	-2.20976500
H	-0.41764700	2.99130000	-2.22652000
H	-1.85007700	3.87549400	-2.77257000
H	-1.86062500	2.10621700	-2.74656300
C	-5.12165800	-2.76286600	0.07185700
H	-5.56202000	-3.63372400	-0.42854200
H	-5.77433000	-1.90083200	-0.10883900
H	-5.11884700	-2.96376900	1.15001500
C	-3.39159800	-2.03997800	-2.39923300
H	-4.04751900	-1.19202300	-2.62826800
H	-3.75561900	-2.90515500	-2.96623700
H	-2.38523400	-1.80155900	-2.76377900
C	-2.31110000	-3.95866700	-0.23781800
H	-2.73391000	-4.82863000	-0.75521200
H	-2.26285700	-4.20276400	0.82994700
H	-1.28659300	-3.81763400	-0.59927200
H	0.00179100	-0.79815400	1.89553800
P	2.47769500	-0.26996800	0.12854700
C	3.95802400	-1.43458800	0.50457200
C	3.17261900	1.50618500	-0.10473700
C	1.71634800	-0.82175600	-1.54838500
C	3.50892400	-2.89940600	0.33902200
C	4.28718600	-1.28516800	2.00670600
C	5.24201000	-1.23010100	-0.31440700
C	2.68642900	-1.21433800	-2.67295300
C	0.77471000	-2.00783800	-1.24666700
C	0.79107700	0.28833800	-2.07815700
C	3.91256600	1.80703300	-1.41759000
C	1.98275100	2.47962800	0.04514500
C	4.11597900	1.84894700	1.06420800
H	2.35656200	3.51209000	0.01427600
H	1.23787300	2.38093600	-0.74399800
H	1.47247100	2.33299800	1.00338500
H	4.30349700	2.83349700	-1.38509700
H	3.25967500	1.74071300	-2.29113400
H	4.76045000	1.13615600	-1.57855100
H	4.33182400	2.92487100	1.03431300
H	3.65797300	1.63384200	2.03586400

H	5.07495500	1.32985100	1.00516100
H	5.97598800	-1.99834000	-0.03390100
H	5.06781500	-1.31680100	-1.38984900
H	5.70659200	-0.25927500	-0.12488500
H	5.03699000	-2.03825900	2.28397600
H	4.69937500	-0.31030100	2.26712500
H	3.39805700	-1.45301500	2.62470100
H	4.29019700	-3.55251300	0.74889100
H	2.58556800	-3.10762800	0.89115500
H	3.36633400	-3.18914500	-0.70402100
H	2.11154100	-1.44388900	-3.58093800
H	3.38384900	-0.40922800	-2.91870000
H	3.27076500	-2.10520300	-2.43028800
H	0.23784400	-2.28205300	-2.16538800
H	1.29099100	-2.90175900	-0.89570000
H	0.02644900	-1.72900900	-0.49734700
H	0.18827900	-0.11564800	-2.90227400
H	1.33492200	1.14929600	-2.47269300
H	0.09620500	0.63453300	-1.30760800

5:

$E_{\omega B97X\text{-}D/6\text{-}31+G(d)}$=
$G_{\omega B97X\text{-}D/6\text{-}31+G(d)}$=
$E_{\omega B97X\text{-}D/6\text{-}311+G(d,p)}$=

P	7.228149	17.127580	4.468942
Si	5.080815	16.844015	8.466485
Si	9.005983	20.524673	6.746698
O	6.746694	16.790357	3.079892
C	6.116363	16.688675	5.816421
H	5.388164	15.902041	5.627943
C	6.219005	17.301183	7.014722
C	7.219813	18.369355	7.247369
H	7.424167	18.601498	8.292119
C	7.834894	19.109176	6.301068
C	7.550265	18.876260	4.823858
H	8.368664	19.222560	4.183747
H	6.655805	19.442706	4.523234
C	3.853311	15.532167	7.918116
H	4.358368	14.613130	7.598881
H	3.227723	15.884269	7.089766
H	3.188907	15.274303	8.751464
C	4.192021	18.412364	9.007942
H	4.904574	19.192186	9.301974
H	3.542182	18.211084	9.868005
H	3.569422	18.812004	8.199096
C	6.161193	16.197950	9.866884
H	6.708448	15.298944	9.560595
H	5.544101	15.938488	10.735591
H	6.894102	16.945910	10.191484
C	8.363094	22.083332	5.908024
H	9.032100	22.930297	6.102053
H	7.365467	22.348141	6.277396
H	8.297148	21.953849	4.821137
C	9.064926	20.748947	8.612611
H	8.076090	20.984383	9.023168
H	9.737312	21.577501	8.865593
H	9.440331	19.851301	9.117678
C	10.719387	20.107191	6.083995
H	11.421327	20.923516	6.293255
H	10.706485	19.951195	4.998748
H	11.111106	19.196228	6.551422
H	8.456563	16.504911	4.796550

P_anion_from_**5**:
$E_{\omega B97X\text{-}D/6\text{-}31+G(d)}$= -1428.472835
$G_{\omega B97X\text{-}D/6\text{-}31+G(d)}$= -1428.209809
$E_{\omega B97X\text{-}D/6\text{-}311+G(d,p)}$= -1428.6665631

C	7.204281	17.175525	4.443173
Si	6.324180	16.997947	6.099458
C	6.994151	18.285530	7.309927
C	4.471422	17.233531	5.890481
C	3.611725	17.103948	7.092045
C	2.288968	16.831011	7.072231
C	1.579701	16.628474	5.739347
P	2.101601	17.913217	4.487177
C	3.896413	17.518855	4.697818
O	1.663251	17.388599	3.071706
C	6.654453	15.281848	6.816240
Si	1.297754	16.622328	8.651320
C	-0.052717	17.940110	8.715974
C	0.481798	14.919731	8.634903
C	2.403008	16.782378	10.171197
H	4.520552	17.634658	3.807151
H	4.110850	17.203369	8.058079
H	0.489222	16.666185	5.853391
H	1.821619	15.626480	5.339781
H	7.054718	18.172826	4.012830
H	6.845229	16.437166	3.716547
H	8.283143	17.023752	4.571217
H	6.130859	15.148600	7.771124
H	7.725447	15.125948	6.996189
H	6.306510	14.497867	6.132934
H	6.832565	19.302287	6.932394
H	8.071363	18.149716	7.468707
H	6.501602	18.209450	8.287137
H	-0.159163	14.781936	9.514561
H	1.234131	14.121845	8.634544
H	-0.142501	14.791671	7.742279
H	3.191481	16.020254	10.177573
H	1.809040	16.657472	11.084998
H	2.883215	17.767110	10.217823
H	-0.693286	17.805205	9.596532
H	-0.691697	17.896004	7.825764
H	0.384069	18.944769	8.762787

Dimer_anion_1

$E_{\omega B97X\text{-}D/6\text{-}31+G(d)}$= -2856.4638126

$G_{\omega B97X\text{-}D/6\text{-}31+G(d)}$= -2855.923913

$E_{\omega B97X\text{-}D/6\text{-}311+G(d,p)}$= -2856.8524984

P	-1.710412	-1.664819	1.305538
P	0.395542	-0.370991	-0.364470
Si	-1.586433	2.837649	0.655809
Si	-5.364554	-0.985201	-0.884675
Si	3.852414	2.581574	-0.446184
Si	3.984451	-3.073195	-0.048738
O	-1.110882	-2.326261	2.533345
O	-0.095302	-0.789451	-1.733759
C	-0.939887	-0.036763	0.882586
H	-0.401484	0.247145	1.800241
C	-1.904143	1.041281	0.404410
C	-3.136354	0.654743	-0.166762
H	-3.607673	1.389184	-0.832028
C	-3.855464	-0.518335	0.053507
C	-3.482589	-1.305449	1.294407
H	-4.019857	-2.256786	1.394643
H	-3.669926	-0.725081	2.216136
C	1.448882	1.088500	-0.395905
H	0.958271	2.051272	-0.491070
C	2.797622	1.007042	-0.389739
C	3.505011	-0.288518	-0.343267
H	4.565380	-0.251766	-0.592849
C	2.966715	-1.483972	-0.027096
C	1.520947	-1.593556	0.409158

H	1.468669	-1.452685	1.498970
H	1.116891	-2.594525	0.216983
C	-0.260305	3.099409	1.989795
H	-0.618711	2.706777	2.950457
H	-0.056836	4.169897	2.123393
H	0.691934	2.603990	1.768663
C	-3.149179	3.718878	1.278646
H	-3.975320	3.597981	0.565938
H	-2.978578	4.795468	1.413799
H	-3.478000	3.299735	2.237526
C	-1.120052	3.831029	-0.903566
H	-0.268190	3.421763	-1.456877
H	-0.888358	4.874846	-0.651541
H	-1.975053	3.840132	-1.593057
C	-6.881083	-1.128606	0.252454
H	-6.697759	-1.846633	1.062733
H	-7.768375	-1.469460	-0.297458
H	-7.117615	-0.162062	0.715244
C	-5.753274	0.294761	-2.223097
H	-5.922819	1.288643	-1.790332
H	-6.659753	0.009920	-2.772317
H	-4.933745	0.379951	-2.946861
C	-5.193150	-2.681488	-1.719095
H	-4.934795	-3.454457	-0.983123
H	-4.397885	-2.666427	-2.474399
H	-6.126467	-2.988268	-2.209983
C	2.741206	4.092656	-0.574975
H	2.167008	4.091412	-1.508642
H	3.351901	5.003653	-0.560262
H	2.030699	4.153348	0.257567
C	4.866931	2.640910	1.140450
H	4.214985	2.690107	2.020647
H	5.521075	3.521163	1.152847
H	5.499583	1.750654	1.239857
C	5.003501	2.492140	-1.935076
H	5.669242	1.622628	-1.881550
H	5.631882	3.389729	-1.987061
H	4.433661	2.423425	-2.868973
C	5.791426	-2.683855	-0.405794
H	6.217111	-2.015716	0.352107
H	5.916349	-2.210381	-1.386683
H	6.381013	-3.608670	-0.405772
C	3.290824	-4.205918	-1.385606
H	2.223679	-4.404008	-1.231312
H	3.813192	-5.170566	-1.384442
H	3.407887	-3.757017	-2.378619
C	3.810129	-3.901678	1.634571
H	4.214772	-3.265754	2.430942
H	4.348205	-4.857089	1.657125
H	2.758538	-4.105054	1.869930
H	-1.540785	-2.448394	0.139727

TS_intramolecular_H^{+}:

$E_{\omega B97X\text{-}D/6\text{-}31+G(d)}$= -2856.419286
$G_{\omega B97X\text{-}D/6\text{-}31+G(d)}$= -2855.881915
$E_{\omega B97X\text{-}D/6\text{-}311+G(d,p)}$= -2856.8086475

P	1.782859	-1.795849	1.441898
P	-0.760932	-0.161459	0.992704
Si	1.945370	2.343644	1.529413
Si	5.129089	-0.931169	-1.624104
Si	-3.383385	2.746546	-1.321573
Si	-4.292748	-2.699208	-0.057265
O	1.121470	-3.168143	1.645509
O	-1.015506	-0.111611	2.483497
C	0.966418	-0.376515	0.491564

H	0.951975	-0.602624	-0.582620
C	2.060099	0.617478	0.852531
C	3.251110	0.427313	0.008945
H	3.774856	1.324649	-0.337665
C	3.751524	-0.774750	-0.380851
C	3.180953	-2.043733	0.233925
H	3.958632	-2.592739	0.783200
H	2.807218	-2.749347	-0.528295
C	-1.427930	1.254862	0.090573
H	-0.824904	2.158717	0.044551
C	-2.664036	1.222496	-0.454985
C	-3.515742	0.015956	-0.382259
H	-4.571202	0.171772	-0.606553
C	-3.103491	-1.236228	-0.093648
C	-1.637880	-1.526602	0.155694
H	-1.145408	-1.707732	-0.810957
H	-1.479233	-2.431749	0.751972
C	1.787265	3.659911	0.162681
H	2.613260	3.577365	-0.555754
H	1.816077	4.672404	0.587542
H	0.852670	3.564251	-0.403480
C	3.585465	2.718780	2.402263
H	3.726135	2.054609	3.264201
H	3.610561	3.755363	2.762972
H	4.441948	2.575718	1.731700
C	0.578771	2.678825	2.788630
H	-0.421600	2.726088	2.346308
H	0.781381	3.648406	3.263487
H	0.551631	1.908710	3.566169
C	4.511523	-1.817979	-3.178233
H	4.095221	-2.802610	-2.930780
H	5.325083	-1.971822	-3.898603
H	3.723680	-1.237806	-3.674269
C	5.797372	0.766728	-2.113111
H	5.025804	1.382407	-2.590738
H	6.626544	0.657888	-2.823490
H	6.173593	1.315228	-1.240927
C	6.544496	-1.963110	-0.908790
H	6.199046	-2.966693	-0.630650
H	6.960666	-1.492760	-0.009366
H	7.356780	-2.080559	-1.637313
C	-2.105774	4.126166	-1.342724
H	-1.801974	4.414508	-0.329406
H	-2.522257	5.015386	-1.831100
H	-1.206823	3.832199	-1.897326
C	-3.853250	2.257137	-3.079283
H	-2.972885	1.931959	-3.646009
H	-4.305871	3.102744	-3.611258
H	-4.577462	1.433613	-3.081363
C	-4.923511	3.289994	-0.382030
H	-5.676604	2.493666	-0.349897
H	-5.381111	4.162761	-0.863650
H	-4.677903	3.562085	0.651014
C	-6.016436	-2.151955	-0.581308
H	-6.017102	-1.733656	-1.594819
H	-6.424494	-1.395964	0.099901
H	-6.699349	-3.010194	-0.573168
C	-4.338043	-3.396177	1.692004
H	-3.339948	-3.699244	2.029406
H	-4.989457	-4.277468	1.740113
H	-4.718533	-2.652065	2.401369
C	-3.634170	-4.010826	-1.240074
H	-3.598080	-3.633797	-2.269115
H	-4.273117	-4.902226	-1.227632
H	-2.621058	-4.323170	-0.959671
H	2.352056	-0.444607	2.101197

Dimer_anion_2

$E_{\omega B97X\text{-}D/6\text{-}31+G(d)}$= -2856.48904

$G_{\omega B97X\text{-}D/6\text{-}31+G(d)}$= -2855.944423

$E_{\omega B97X\text{-}D/6\text{-}311+G(d,p)}$= -2856.8793281

P	-1.438699	-1.699503	0.881441
P	0.718134	-0.115269	-0.769521
Si	-2.072608	2.461325	-0.924857
Si	-5.734462	-1.069453	0.353037
Si	3.985626	2.554898	0.943924
Si	4.189881	-2.911777	-0.531074
O	-0.502088	-1.764811	2.139728
O	0.661436	-0.051570	-2.285343
C	-0.934879	-0.045362	0.035432
H	-0.798122	0.606166	0.910109
C	-1.990930	0.542760	-0.944554
C	-3.399042	0.041493	-0.758836
H	-4.050833	0.263487	-1.605181
C	-3.929288	-0.573278	0.314014
C	-3.048618	-0.943354	1.486038
H	-3.536504	-1.670382	2.148481
H	-2.800257	-0.070893	2.109729
C	1.678796	1.227020	-0.025087
H	1.163704	2.163674	0.181073
C	2.999399	1.104036	0.230769
C	3.722514	-0.155193	-0.036901
H	4.810398	-0.085184	-0.044863
C	3.155665	-1.364454	-0.232590
C	1.656323	-1.546454	-0.151275
H	1.353571	-1.697897	0.898497
H	1.314673	-2.436780	-0.692551
C	-1.950819	3.079099	0.854070
H	-2.664993	2.545954	1.494320
H	-2.188128	4.149102	0.903260
H	-0.951769	2.939711	1.283137
C	-3.722732	3.042298	-1.637762
H	-3.890718	2.638479	-2.643760
H	-3.733115	4.137064	-1.711434
H	-4.565558	2.737138	-1.007031
C	-0.720424	3.225918	-1.996114
H	0.290471	2.905203	-1.728885
H	-0.760922	4.320640	-1.926313
H	-0.877700	2.948542	-3.045419
C	-6.541373	-0.316813	1.886648
H	-6.016124	-0.628601	2.797830
H	-7.588537	-0.630933	1.979281
H	-6.517749	0.779100	1.846093
C	-6.639708	-0.467361	-1.189404
H	-6.584566	0.623570	-1.288430
H	-7.699606	-0.746081	-1.139544
H	-6.220950	-0.910480	-2.100874
C	-5.871659	-2.949377	0.465605
H	-5.325201	-3.336601	1.334178
H	-5.452292	-3.424998	-0.428972
H	-6.918370	-3.264510	0.560582
C	2.837014	4.014820	1.241414
H	2.364571	4.353700	0.311701
H	3.401719	4.858928	1.655367
H	2.043527	3.762592	1.954754
C	4.777601	1.997897	2.559646
H	4.014478	1.719173	3.295542
H	5.390355	2.798721	2.991199
H	5.425227	1.126963	2.402541
C	5.330275	3.023049	-0.291391
H	6.007528	2.182561	-0.484484
H	5.933080	3.856166	0.090425
H	4.894587	3.330134	-1.249293
C	6.024553	-2.515879	-0.375120

H	6.269540	-2.130570	0.621707
H	6.342552	-1.772668	-1.115849
H	6.618425	-3.423504	-0.538339
C	3.815820	-3.557352	-2.262018
H	2.745976	-3.760450	-2.389708
H	4.359225	-4.490620	-2.454722
H	4.110264	-2.827448	-3.025140
C	3.687014	-4.203904	0.744466
H	3.905204	-3.859100	1.762046
H	4.224528	-5.145961	0.581089
H	2.612789	-4.416377	0.688375
H	-1.691832	0.288342	-1.971147

TS_ring_closing:

$E_{\omega B97X\text{-}D/6\text{-}31+G(d)}$= -2856.474159

$G_{\omega B97X\text{-}D/6\text{-}31+G(d)}$= -2855.923842

$E_{\omega B97X\text{-}D/6\text{-}311+G(d,p)}$= -2856.8638862

P	0.269065	-1.266685	-1.133610
P	0.808952	-1.573986	1.888924
Si	-3.324717	-2.119555	0.677477
Si	-2.442467	2.366993	-1.607737
Si	3.712767	-1.180376	-1.405076
Si	1.447898	2.929283	1.612470
O	0.279562	-2.571783	-1.971184
O	0.472192	-2.486815	3.057652
C	-0.482327	-1.672611	0.547572
H	-0.621132	-2.760873	0.481131
C	-1.811777	-0.986529	0.957177
C	-2.094975	0.350835	0.336742
H	-2.688296	1.016935	0.965691
C	-1.781993	0.756479	-0.906485
C	-1.033101	-0.143732	-1.868221
H	-0.530059	0.445633	-2.647072
H	-1.738560	-0.798130	-2.399994
C	2.296186	-1.829787	0.989751
H	2.593655	-2.860980	0.802025
C	2.459844	-0.872758	-0.018309
C	2.164184	0.560135	0.255941
H	2.538545	1.255687	-0.496555
C	1.541237	1.076316	1.333755
C	0.903350	0.183080	2.381770
H	-0.110320	0.535738	2.614362
H	1.461707	0.216928	3.328548
C	-3.089965	-3.690157	1.692894
H	-2.259436	-4.298413	1.316828
H	-3.996592	-4.307301	1.661374
H	-2.877273	-3.451521	2.741863
C	-3.514978	-2.549272	-1.144046
H	-3.790928	-1.663021	-1.727952
H	-4.301625	-3.301750	-1.280867
H	-2.581043	-2.950402	-1.555098
C	-4.863656	-1.209871	1.281561
H	-4.758592	-0.904295	2.329956
H	-5.749565	-1.852424	1.206993
H	-5.050449	-0.308890	0.684965
C	-3.347688	1.955425	-3.213418
H	-2.671755	1.490141	-3.940978
H	-3.764339	2.860117	-3.673320
H	-4.173087	1.256479	-3.030270
C	-3.654693	3.190282	-0.419315
H	-4.502964	2.529927	-0.202121
H	-4.052358	4.108191	-0.869937
H	-3.188802	3.464450	0.534132
C	-1.032916	3.557008	-2.009574
H	-0.236242	3.053056	-2.570763
H	-0.588753	3.970470	-1.097389

H	-1.392290	4.395588	-2.619004
C	3.899724	-3.025385	-1.709155
H	4.308853	-3.540862	-0.832223
H	4.577135	-3.208619	-2.552474
H	2.923780	-3.463964	-1.945326
C	3.190988	-0.307945	-2.993525
H	2.308313	-0.790221	-3.428001
H	4.001406	-0.341524	-3.732924
H	2.944955	0.745998	-2.815229
C	5.364083	-0.448424	-0.844486
H	5.273310	0.627628	-0.649998
H	6.136382	-0.587091	-1.611627
H	5.711677	-0.926659	0.079053
C	2.201454	3.852633	0.151975
H	1.732724	3.573112	-0.798089
H	3.275977	3.648462	0.071242
H	2.075790	4.934573	0.282066
C	2.412090	3.357580	3.178747
H	2.014257	2.818265	4.047153
H	2.349737	4.431253	3.396213
H	3.471151	3.092773	3.075217
C	-0.351248	3.434265	1.872284
H	-0.983216	3.027416	1.077106
H	-0.465095	4.525236	1.881211
H	-0.733255	3.049953	2.826188
H	-1.829280	-0.852467	2.050760

Final_dimer_anion:
$E_{\omega B97X\text{-}D/6\text{-}31+G(d)}$= -2856.4976302
$G_{\omega B97X\text{-}D/6\text{-}31+G(d)}$= -2855.945585
$E_{\omega B97X\text{-}D/6\text{-}311+G(d,p)}$= -2856.888392

P	0.001142	-1.510172	-0.838733
P	0.573924	-1.717033	2.007336
Si	-3.716142	-1.384180	0.620892
Si	-1.703614	2.656850	-1.648778
Si	3.056417	-1.698266	-1.554657
Si	2.041959	2.592173	1.627729
O	-0.169101	-2.748316	-1.705197
O	0.176066	-2.503923	3.249870
C	-0.856256	-1.624702	0.778723
H	-1.221872	-2.660008	0.814881
C	-2.008162	-0.633903	1.053849
C	-1.931698	0.698941	0.368119
H	-2.464000	1.492021	0.895386
C	-1.394718	0.995184	-0.828434
C	-0.677048	-0.030277	-1.688771
H	0.145546	0.436000	-2.246979
H	-1.363610	-0.428179	-2.448870
C	1.904357	-2.183816	0.987814
H	1.870240	-3.245472	0.718394
C	1.751040	-1.252246	-0.240437
C	1.917734	0.215345	0.111478
H	2.334087	0.851519	-0.672561
C	1.614432	0.795297	1.285323
C	0.929715	0.034138	2.406645
H	-0.000796	0.545879	2.690641
H	1.556845	0.016602	3.308653
C	-3.970616	-2.951696	1.635431
H	-3.261367	-3.739973	1.358201
H	-4.982407	-3.346190	1.480040
H	-3.846498	-2.752005	2.706506
C	-3.781111	-1.775384	-1.218582
H	-3.704243	-0.857482	-1.813887
H	-4.728254	-2.265486	-1.475550
H	-2.962338	-2.441642	-1.515471
C	-5.054596	-0.129302	1.057572

H	-4.980704	0.181722	2.106940
H	-6.052535	-0.558075	0.904908
H	-4.976146	0.768661	0.433255
C	-2.561845	2.309482	-3.294981
H	-1.923717	1.711270	-3.956824
H	-2.799911	3.244374	-3.817249
H	-3.497684	1.757661	-3.143566
C	-2.821516	3.742210	-0.587906
H	-3.798875	3.269595	-0.433105
H	-2.991041	4.702533	-1.090303
H	-2.391061	3.955031	0.397343
C	-0.085672	3.556681	-2.015903
H	0.648606	2.887137	-2.481071
H	0.364690	3.967212	-1.106197
H	-0.260091	4.389502	-2.708415
C	3.196130	-3.556382	-1.804654
H	3.567726	-4.047814	-0.898378
H	3.894592	-3.778268	-2.621441
H	2.219783	-3.984624	-2.053201
C	2.617623	-0.867133	-3.197382
H	1.672971	-1.252181	-3.599548
H	3.401507	-1.052135	-3.942670
H	2.521308	0.220814	-3.086407
C	4.723437	-1.018808	-0.987006
H	4.727511	0.077349	-0.957992
H	5.519535	-1.340298	-1.670637
H	4.965652	-1.382191	0.018020
C	3.046507	3.321280	0.208480
H	2.534329	3.244995	-0.756367
H	4.010225	2.805214	0.118918
H	3.250762	4.382877	0.395132
C	3.082352	2.670441	3.201270
H	2.526110	2.292579	4.067556
H	3.377753	3.704172	3.420364
H	3.994926	2.070673	3.100640
C	0.460141	3.579046	1.924408
H	-0.231061	3.482023	1.081324
H	0.675085	4.644648	2.071426
H	-0.059229	3.215309	2.819719
H	-2.066135	-0.457799	2.139108

Final_dimer:

$E_{\omega B97X\text{-}D/6\text{-}31+G(d)}$= -2857.0462887

$G_{\omega B97X\text{-}D/6\text{-}31+G(d)}$= -2856.480247

$E_{\omega B97X\text{-}D/6\text{-}311+G(d,p)}$= -2857.4355514

P	0.089003	-1.524464	0.808126
P	-0.419818	-1.585084	-2.094061
Si	3.786697	-1.253506	-0.615846
Si	1.603939	2.707810	1.639824
Si	-2.934121	-1.886724	1.583651
Si	-2.222578	2.575248	-1.515242
O	0.311213	-2.775314	1.625133
O	-0.086656	-2.339100	-3.356291
C	0.926714	-1.579627	-0.832032
H	1.313458	-2.605823	-0.897025
C	2.057017	-0.554073	-1.082017
H	2.138089	-0.378766	-2.165129
C	1.910207	0.773126	-0.395772
H	2.416295	1.587319	-0.914833
C	1.348064	1.043101	0.794710
C	0.669663	-0.014284	1.648879
H	-0.178720	0.408974	2.200988
H	1.365608	-0.384632	2.413615
C	-1.765235	-2.183013	-1.020412
H	-2.734458	-2.067847	-1.517204
H	-1.615108	-3.245127	-0.797245
C	-1.666532	-1.301774	0.254013

C	-1.930447	0.158844	-0.072915
H	-2.402790	0.734844	0.722854
C	-1.664871	0.794090	-1.225757
C	-0.925627	0.139629	-2.378892
H	-0.032796	0.726126	-2.633122
H	-1.543863	0.126485	-3.286475
C	4.078457	-2.822344	-1.614225
H	3.412963	-3.637783	-1.308309
H	5.109181	-3.170474	-1.475348
H	3.924633	-2.645019	-2.685215
C	3.838538	-1.618471	1.227580
H	3.723019	-0.698071	1.812315
H	4.803164	-2.065774	1.496536
H	3.049915	-2.318178	1.528724
C	5.070843	0.048443	-1.059455
H	4.992511	0.344708	-2.112435
H	6.082909	-0.340410	-0.895436
H	4.954461	0.948370	-0.444359
C	2.659425	3.842694	0.571022
H	3.653193	3.412560	0.398921
H	2.797119	4.803858	1.081074
H	2.206861	4.048766	-0.405580
C	-0.042909	3.528395	2.050981
H	-0.524321	3.945673	1.160731
H	0.112176	4.349479	2.761573
H	-0.742567	2.820840	2.513085
C	2.505740	2.353520	3.258814
H	1.901740	1.724918	3.924377
H	2.724969	3.286408	3.792368
H	3.455631	1.836118	3.077666
C	-2.904250	-3.755662	1.741766
H	-3.258723	-4.242536	0.825720
H	-3.562812	-4.070514	2.560427
H	-1.890380	-4.109190	1.953887
C	-2.526618	-1.053470	3.221786
H	-1.555361	-1.380982	3.609809
H	-3.287925	-1.310560	3.968169
H	-2.512417	0.039924	3.133994
C	-4.631652	-1.325663	0.991165
H	-4.711969	-0.233928	0.941119
H	-5.404166	-1.686847	1.681032
H	-4.861642	-1.726443	-0.003240
C	-3.306466	2.599118	-3.057119
H	-2.752798	2.274700	-3.946214
H	-3.674440	3.614363	-3.249494
H	-4.176431	1.941952	-2.940658
C	-0.697356	3.636687	-1.826487
H	0.050676	3.497066	-1.039944
H	-0.955813	4.701520	-1.867757
H	-0.227630	3.370532	-2.781210
C	-3.227489	3.185768	-0.046555
H	-4.151218	2.603789	0.056821
H	-3.509883	4.234465	-0.199227
H	-2.680998	3.123886	0.900034

4. References

[47] R. K. Harris, E. D. Becker, S. M. Cabral de Menezes, R. Goodfellow, P. Granger, *Pure Appl. Chem.* **2001**, *73*, 1795–1818.

[48] G. R. Fulmer, A. J. M. Miller, N. H. Sherden, H. E. Gottlieb, A. Nudelman, B. M. Stoltz, J. E. Bercaw, K. I. Goldberg, *Organometallics* **2010**, *29*, 2176–2179.

[49] N. Avarvari, P. Le Floch, F. Mathey, *J. Am. Chem. Soc*. **1996**, *118*, 11978–11979.

[50] Bruker, *APEX III.* Bruker AXS Inc., Madison, Wisconsin, USA: **2019**.

[51] Bruker, *Apex 4.* Bruker AXS Inc.; Madison, WI, USA: **2021**.

[52] Sheldrick, G. M. *SADABS*; *University of Göttingen: Germany* **1996**.

[53] Coppens, P. The Evaluation of Absorption and Extinction in Single-Crystal Structure Analysis. *Crystallographic Computing, Copenhagen, Muksgaard* **1979**.

[54] O. V. Dolomanov, L. J. Bourhis, R. J. Gildea, J. a. K. Howard, H. Puschmann, *J. Appl. Cryst.* **2009**, *42*, 339–341.

[55] Sheldrick, G. M. A short history of SHELX. *Acta Crystallogr., Sect. A: Found. Crystallogr.* **2008**, *A64*, 112.

[56] Sheldrick, G. M. Crystal structure refinement with SHELXL. *Acta Crystallogr., Sect. C: Struct. Chem.* **2015**, *C71*, 3.

[57] The facility “CheckCIF,” can be found at http://checkcif.iucr.org.

[58] Gaussian 16, Revision C.02, M. J. Frisch, G. W. Trucks, H. B. Schlegel, G. E. Scuseria, M. A. Robb, J. R. Cheeseman, G. Scalmani, V. Barone, G. A. Petersson, H. Nakatsuji, X. Li, M. Caricato, A. V. Marenich, J. Bloino, B. G. Janesko, R. Gomperts, B. Mennucci, H. P. Hratchian, J. V. Ortiz, A. F. Izmaylov, J. L. Sonnenberg, D. Williams-Young, F. Ding, F. Lipparini, F. Egidi, J. Goings, B. Peng, A. Petrone, T. Henderson, D. Ranasinghe, V. G. Zakrzewski, J. Gao, N. Rega, G. Zheng, W. Liang, M. Hada, M. Ehara, K. Toyota, R. Fukuda, J. Hasegawa, M. Ishida, T. Nakajima, Y. Honda, O. Kitao, H. Nakai, T. Vreven, K. Throssell, J. A. Montgomery, Jr., J. E. Peralta, F. Ogliaro, M. J. Bearpark, J. J. Heyd, E. N. Brothers, K. N. Kudin, V. N. Staroverov, T. A. Keith, R. Kobayashi, J. Normand, K. Raghavachari, A. P. Rendell, J. C. Burant, S. S. Iyengar, J. Tomasi, M. Cossi, J. M. Millam, M. Klene, C. Adamo, R. Cammi, J. W. Ochterski, R. L. Martin, K. Morokuma, O. Farkas, J. B. Foresman, and D. J. Fox, Gaussian, Inc., Wallingford CT, **2019**.

[59] F. Weigend, R. Ahlrichs, *Phys. Chem. Chem. Phys.*, **2005**, *7*, 3297.

[60] A. D. Becke, *J. Chem. Phys.*, **1993**, *98*, 5648–5652.

[61] Lee, C.; Yang, W.; Parr, R. G. *Phys. Rev. B* **1988**, *37*, 785–789.

[62] Grimme, S.; Ehrlich, S.; Goerigk, L. *J. Comput. Chem.* **2011**, *32*, 1456–1465.

[63] L. Bennett, B. Melchers, B. Proppe, *Curta: A General-purpose High-Performance Computer at ZEDAT, Freie Universität Berlin*, Freie Universität Berlin, **2020**.

[64] T. Helgaker, M. Jaszuński, K. Ruud, *Chem. Rev.*, **1999**, *99*, 293–352.

[65] R. Ditchfield, *J. Chem. Phys.*, **1972**, *56*, 5688–5691.

[66] K. Wolinski, J. F. Hinton, P. Pulay, *J. Am. Chem. Soc.*, **1990**, *112*, 8251–8260.
